# Synthetic Biology Open Language (SBOL) Version 2.3

**DOI:** 10.1515/jib-2019-0025

**Published:** 2019-06-13

**Authors:** Curtis Madsen, Angel Goñi Moreno, Umesh P, Zachary Palchick, Nicholas Roehner, Christian Atallah, Bryan Bartley, Kiri Choi, Robert Sidney Cox, Thomas Gorochowski, Raik Grünberg, Chris Macklin, James McLaughlin, Xianwei Meng, Tramy Nguyen, Matthew Pocock, Meher Samineni, James Scott-Brown, Ysis Tarter, Michael Zhang, Zhen Zhang, Zach Zundel, Jacob Beal, Michael Bissell, Kevin Clancy, John H. Gennari, Goksel Misirli, Chris Myers, Ernst Oberortner, Herbert Sauro, Anil Wipat

**Affiliations:** Boston University, Boston, MA, USA; Newcastle University, Newcastle, UK; Kerala Technological University, Thiruvananthapuram, India; Zymergen, Emeryville, CA, USA; Raytheon BBN Technologies, Cambridge, MA, USA; University of Washington, Seattle, WA, USA; Prospect Bio, Brisbane, CA, USA; University of Bristol, Bristol, UK; KAUST, Thuwal, Saudi Arabia; Amyris, Inc., Emeryville, CA, USA; DOE Joint Genome Institute, Walnut Creek, CA, USA; University of Utah, Salt Lake City, UT, USA; Turing Ate My Hamster, Ltd., Newcastle, UK; Imperial College, London, UK; Utah State University, Logan, UT, USA; BioCoder Consulting, Carlsbad, CA, USA; Keele University, Keele, Staffordshire, UK

**Keywords:** Synthetic Biology, Synthetic Biology Open Language, Standards

## Abstract

Synthetic biology builds upon the techniques and successes of genetics, molecular biology, and metabolic engineering by applying engineering principles to the design of biological systems. The field still faces substantial challenges, including long development times, high rates of failure, and poor reproducibility. One method to ameliorate these problems is to improve the exchange of information about designed systems between laboratories. The synthetic biology open language (SBOL) has been developed as a standard to support the specification and exchange of biological design information in synthetic biology, filling a need not satisfied by other pre-existing standards. This document details version 2.3.0 of SBOL, which builds upon version 2.2.0 published in last year’s JIB Standards in Systems Biology special issue. In particular, SBOL 2.3.0 includes means of succinctly representing sequence modifications, such as insertion, deletion, and replacement, an extension to support organization and attachment of experimental data derived from designs, and an extension for describing numerical parameters of design elements. The new version also includes specifying types of synthetic biology activities, unambiguous locations for sequences with multiple encodings, refinement of a number of validation rules, improved figures and examples, and clarification on a number of issues related to the use of external ontology terms.

Automatically generated rough PDF by ProofCheck from River Valley Technologies Ltd

**Jacob Beal** is the corresponding author.
CC BY © 2019, Curtis Madsen et al., published by Walter de Gruyter GmbH, Berlin/Boston.
This work is licensed under the Creative Commons Attribution 4.0 Public License.

## Synthetic Biology Open Language (SBOL) Version 2.3

**Editors:**

Curtis Madsen — *Boston University, USA*
Angel Goñi Moreno — *Newcastle University, UK*
Umesh P — *Kerala Technological University, India*
Zach Palchick — *Zymergen, USA*
Nicholas Roehner — *Raytheon BBN Technologies, USA*

sbol-editors@googlegroups.com

**Chair:**

Anil Wipat — *Newcastle University, UK*

**Steering Committee:**

Jacob Beal — *Raytheon BBN Technologies, USA*
Michael Bissell — *Amyris, Inc., USA*
John H. Gennari, Herbert Sauro — *University of Washington, USA*
Kevin Clancy — *BioCoder Consulting, USA*
Goksel Misirli — *Keele University, UK*
Chris Myers — *University of Utah, USA*
Ernst Oberortner — *DOE Joint Genome Institute, USA*

**Additional authors, by institution:**

Chris Macklin, Ysis Tarter (*Amyris, USA*),
John Meng (*DOE Joint Genome Institute, USA*) Raik Grünberg (*KAUST, SA*),
Christian Atallah, James McLaughlin (*Newcastle, UK*)
Robert Sidney Cox (*Prospect Bio, USA*), Bryan Bartley (*Raytheon BBN, USA*)
Matthew Pocock (*Turing Ate My Hamster, UK*)
Thomas Gorochowski (*U. of Bristol, UK*), James Scott-Brown (*U. of Oxford, UK*)
Tramy Nguyen, Meher Samineni, Michael Zhang, Zach Zundel (*U. of Utah, USA*)
Kiri Choi (*U. of Washington, USA*), Zhen Zhang (*Utah State U., USA*)

**Non-author contributors, by institution:**

Douglas Densmore (*Boston University, USA*), Paolo Missier (*Newcastle University, UK*),
Jacqueline Quinn (*Google, USA*), Guy-Bart Stan (*Imperial College London, UK*)

Version 2.3

April 1, 2019

### Contents

1 Purpose 4
2 Relation to other BBF RFCs 7
3 Copyright and License Statement 8
4 A Brief History of SBOL 9
5 SBOL Specification Vocabulary 11
5.1 Term Conventions . . . 11
5.2 SBOL Class Names . . . 11
6 Overview of SBOL 13
7 SBOL Data Model 16
7.1 Understanding the UML Diagrams . . . 16
7.2 Naming and Font Conventions . . . 16
7.3 Data Types . . . 17
7.4 Identified . . . 17
7.5 TopLevel . . . 20
7.6 Sequence . . . 21
7.7 ComponentDefinition . . . 22
7.7.1 ComponentInstance . . . 27
7.7.2 Component . . . 28
7.7.3 MapsTo . . . 30
7.7.4 SequenceAnnotation . . . 32
7.7.5 Location . . . 34
7.7.6 SequenceConstraint . . . 36
7.8 Model . . . 38
7.9 ModuleDefinition . . . 39
7.9.1 Module . . . 42
7.9.2 FunctionalComponent . . . 43
7.9.3 Interaction . . . 44
7.9.4 Participation . . . 46
7.10 Collection . . . 47
7.11 CombinatorialDerivation . . . 48
7.11.1 VariableComponent . . . 50
7.12 Implementation . . . 52
7.13 Attachment . . . 53
7.14 ExperimentalData . . . 54
7.15 Experiment . . . 55
7.16 Annotation and Extension of SBOL . . . 56
7.16.1 Annotating SBOL objects . . . 56
7.16.2 GenericTopLevel . . . 58
8 Mapping Between SBOL 1.1 and SBOL 2.x 60
9 Data Model Examples 61
10 SBOL RDF Serialization 67
11 SBOL Compliance 69
12 Recommended Best Practices 70
12.1 SBOL Namespaces . . . 70
12.2 Use of the Version Property . . . 70
12.3 Compliant SBOL Objects . . . 70
12.4 Annotations: Embedded Objects vs. External References . . . 71
12.5 Completeness and Validation . . . 71
12.6 Recommended Ontologies for External Terms . . . 71
12.7 Annotating Entities with Date & Time . . . 72
12.8 Annotating Entities with Authorship information . . . 72
13 Complementary Standards 73
13.1 Adding Provenance with PROV-O . . . 73
13.1.1 Activity . . . 75
13.1.2 Usage . . . 76
13.1.3 Association . . . 76
13.1.4 Plan . . . 78
13.1.5 Agent . . . 78
13.2 Adding Measures/Parameters with OM . . . 93
13.2.1 Measure . . . 93

13.2.2 Unit . . . 95
13.2.3 SingularUnit . . . 95
13.2.4 CompoundUnit . . . 96
13.2.5 UnitMultiplication . . . 96
13.2.6 UnitDivision . . . 97
13.2.7 UnitExponentiation . . . 97
13.2.8 PrefixedUnit . . . 98
13.2.9 Prefix . . . 99
13.2.10 SIPrefix . . . 99
13.2.11 BinaryPrefix . . . 100

**References** **102**

**A Validation Rules** **103**

**B Examples of Serialization** **132**

B.1 Simple Examples . . . 132
B.1.1 Serializing Sequence Objects . . . 132
B.1.2 Serializing ComponentDefinition Objects . . . 132
B.1.3 Serializing SequenceConstraint Objects . . . 132
B.1.4 Serializing Cut Location Objects . . . 133
B.1.5 Serializing Model Objects . . . 134
B.1.6 Serializing ModuleDefinition Objects . . . 134
B.1.7 Serializing Application Specific Data Within SBOL Objects . . . 135
B.1.8 Serializing Application-Specific Data Outside SBOL Objects . . . 135
B.1.9 Serializing Collection Objects . . . 136
B.2 Complex Examples . . . 136
B.2.1 PoPS Receiver . . . 136
B.2.2 Toggle Switch . . . 140

## 1 Purpose

Synthetic biology builds upon the techniques and successes of genetics, molecular biology, and metabolic engineering by applying engineering principles to the design of biological systems. These principles include standardization, modularity, and design abstraction. The field still faces substantial challenges, including long development times, high rates of failure, and poor reproducibility. A common factor of these challenges is the exchange of information about designed systems between laboratories. When designing a synthetic system, synthetic biologists need to exchange information about multiple types of molecules and their expected behavior in the design. Furthermore, there are often multiple degrees of separation between a specified nucleic acid sequence (e.g., a sequence that encodes an enzyme or transcription factor) and the molecular interactions that a designer intends to result from said sequence (e.g., chemical modification of metabolites or regulation of gene expression), yet these different perspectives need to be connected together in the engineering of biological systems.

The *Synthetic Biology Open Language* (SBOL) has been developed as a standard to support the specification and exchange of biological design information in synthetic biology, filling a need not satisfied by other pre-existing standards. Previous nucleic acid sequence description formats lack key capabilities. For example, simple sequence encoding formats such as FASTA encode almost nothing about design rationale. More sophisticated formats such as GenBank and Swiss-Prot support a flat annotation of sequence features that is well suited to the description of natural systems, but is unable to represent the multi-layered design structure common to engineered systems. Figure 1 shows the relationship of selected prior sequence description formats to SBOL 1.x and SBOL 2.x. Modeling languages, such as the Systems Biology Markup Language (SBML) Hucka et al. (2003) can be used represent biological processes, but are not sufficient to represent the associated nucleotide or amino acid sequences. Synthetic biology needs a structured standard that defines how to represent relevant molecules and their functional roles within a designed system, standardized rules on how such information is encoded in a file format, and software libraries to enable the exchange of such data between participating laboratories and as part of the publication process.

To help address these challenges, SBOL introduces a standardized format for the electronic exchange of information on the structural and functional aspects of biological designs. The standard has been designed to support the explicit and unambiguous description of biological designs by means of a well defined data model. The standard further describes the rules and best practices on how to use this data model and populate it with relevant design details. SBOL uses existing Semantic Web practices and resources, such as *Uniform Resource Identifiers* (`URI`s) and ontologies, to unambiguously identify and define genetic design elements. The definition of the data model and associated format, the rules on the addition of data within the format and the representation of this in electronic data files are intended to make the SBOL standard a useful means of promoting global data exchange between laboratories and between software programs.

2.3.0 This document details version 2.3 of SBOL that builds upon the previous version 2.2. In particular, SBOL 2.3 includes means of succinctly representing sequence modifications (such as insertion, deletion, and replacement), an extension to support organization and attachment of experimental data derived from designs, and an extension for describing numerical parameters of design elements. The new version also includes typing of activities, unambiguous locations for sequences with multiple encodings, refinement of a number of validation rules, improved figures and examples, and clarification on a number of issues related to ontology terms.

2.2.0 Version 2.2 of SBOL in turn builds upon SBOL version 2.1. SBOL 2.2 included improved description and validation rules for genetic design provenance, an extension to support combinatorial genetic designs, a new class to add non-SBOL data as attachments, a new class for genetic design implementations, and a description of a methodology to describe the entire design-build-test-learn cycle within the SBOL data model.

2.1.0 Version 2.1 included improved rules for what constitutes a valid SBOL document, new role fields to simplify the expression of sequence features and how components are used in context, and new best practices descriptions to improve the exchange of basic sequence topology information and the description of genetic design provenance.

SBOL 2.x builds upon SBOL 1.1, which focused on representing the structural aspects of genetic designs. Users of the standard were able to exchange information on DNA designs, but they could not represent molecules other than DNA or the functional aspects of designs beyond DNA sequence features. The SBOL 2 data model defined in this specification extends the version 1.1 data model to provide for the most pressing data exchange needs identified by the SBOL community. In particular, the extended data model can:

- Represent non-DNA structural components of a biological design, including RNA, proteins, small molecules and other physical components.
- Describe the behavioral aspects of a biological design, such as the intended or expected molecular interactions, and link to mathematical models written in other standards
- Associate structure and function so that a single design can be understood in terms of its structure, behavior, or both.
- Support rich annotation of biological designs, so that classes of design data that are not explicitly covered by this specification can be safely exchanged

Taken together, these extensions enable SBOL to support the description and exchange of hierarchical, modular representations of both the intended structure and function of designed biological systems.

2.2.0

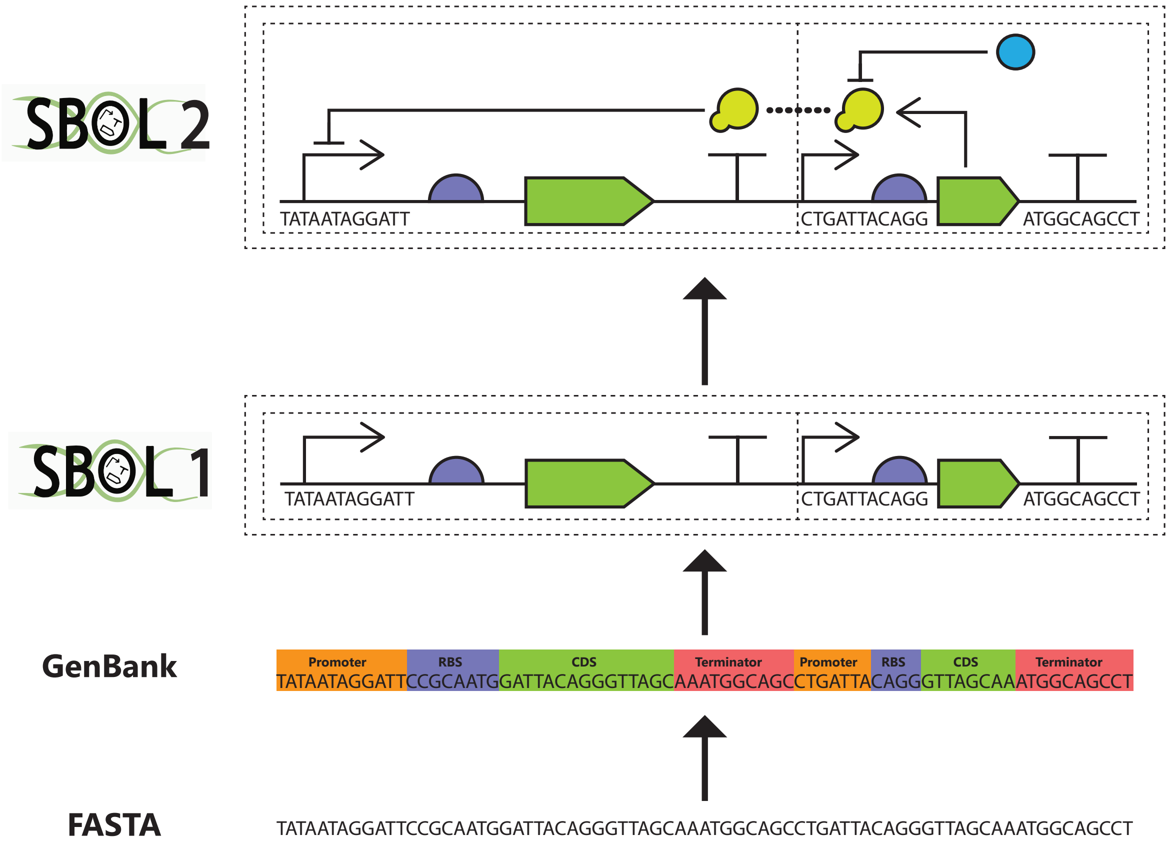

***Figure 1:*** *SBOL 2 extends prior sequence description formats to represent both the structure and function of a genetic design in a modular, hierarchical manner.*

While the ultimate goal of SBOL is to describe synthetic biological designs such that they can be reproduced in the lab with a high degree of fidelity, SBOL 2 does not yet provide a complete catalog of the different classes of data that are necessary to achieve this goal. For example, SBOL 2 does not yet include data on environmental and host context, or details on how the performance of a design is measured. To enable progress towards capturing these types of data, SBOL 2 provides an annotation mechanism that allows SBOL to be easily extended (see Section 7.16). Three scenarios are envisaged for extending SBOL:

- Critical data related to the reproducibility of designs. These include what growth media was used, what temperature the organisms were grown at, or where the recombinant DNA was integrated into the host genome or a plasmid.
- Tool specific data. These could include tool settings specific to the design that is being loaded, such as which windows are to be opened or which settings are to be initialized. Tool makers could also include encrypted proprietary information related to a company or client in an extension.
- Data that are non-essential for reproducibility but are nevertheless useful to many users. There are many cases where specific communities of users require data that cannot be explicitly represented using the SBOL data model. These include data on visualization, evolutionary stability, or other .

The extension mechanism is therefore a critical part of SBOL 2 and will allow others in the community to incorporate their own custom data into SBOL files and contribute to community efforts to expand the scope of SBOL.

The SBOL 2 specifications also add a number of measures to simplify adoption and validation of compatibility with the standard. First, unlike the SBOL 1.1 specification, the SBOL 2 specification explicitly incorporates the primary serialization format for its data model to better show how the standard can be used. Second, the specification includes a set of validation rules for determining the compatibility of a document with SBOL 2, most of which are machine-verifiable. Finally, the specification includes a set of recommended best practices that can allow software tools to take best advantage of the standard and effectively exchange data.

In addition, care has been taken to ensure that all SBOL 2.x specifications are backwards-compatible with previous versions. First, every SBOL 2.x file is also a valid SBOL 2.y file where y is greater than or equal to x. Second, while the changes made in SBOL 2 do mean that an SBOL 1.x file is not a valid SBOL 2.x file, there does exist a direct mapping from the SBOL 1.x data model to the SBOL 2 data model, making it possible to automatically convert any SBOL 1.x file to an SBOL 2 file. Since SBOL 2 can encode all data previously encoded in SBOL 1.1, developers are encouraged to upgrade their SBOL 1.1 compliant software tools to use SBOL 2 software libraries.

Lastly, the SBOL standard has been developed in collaboration between both “wet” bench scientists and “dry” scientific modelers and software developers that are active within the synthetic biology community. As before with SBOL version 1.1, this open community has met to discuss and agree upon the data exchange needs that each version of the SBOL 2.x standard is intended to address. These discussions have informed the efforts of developers within the community to produce a SBOL 2 specification after several rounds of proposal and revision. This specification has been evaluated by the community for its ability to represent a wide range of synthetic biology designs and share these designs between different laboratories. This specification has also informed the development of software libraries that implement the standard, and software tools that employ the standard by means of these libraries, thereby providing further testing of SBOL 2. The publication of this specification is intended to make these capabilities more widely accessible to potential developers and users in the synthetic biology community and beyond.

### 2 Relation to other BBF RFCs

BBF RFC 114 replaces BBF RFC 108 (the SBOL 2.0 standard), as well as the minor update SBOL 2.0.1.

BBF RFC 114 updates BBF RFC 30 (RDF-based framework for synthetic biology data), as it proposes a standard conforming to BBF RFC 30.

BBF RFC 114 also implicitly supersedes the previously replaced BBF RFC 87 (SBOL 1.1, replaced by BBF RFC 108), BBF RFC 84 (SBOL 1.0, replaced by BBF RFC 87) and BBF RFC 31 (PoBoL, replaced by BBF RFC 84).

## 3 Copyright and License Statement

Copyright (C) The BioBricks Foundation and all authors listed on this BBF RFC. This work is made available under the Creative Commons Attribution 4.0 International Public License. To view a copy of this license visit https://creativecommons.org/licenses/by/4.0/.

In addition to the listed authors, the following people are specifically recognized as additional contributors sharing in the copyright (alphabetically by institution): Douglas Densmore (Boston University, USA), Jacqueline Quinn (Google, USA), and Guy-Bart Stan (Imperial College London, UK).

The authors would also like to thank Michael Hucka for developing the LaTeX style file used to develop this document (Hucka, 2017).

## 4 A Brief History of SBOL

The SBOL effort was kickstarted in 2006 with the goal of developing a data exchange standard for genetic designs. Herbert Sauro (University of Washington) secured a modest grant from Microsoft in the field of computational synthetic biology, which was used to fund the initial meeting in Seattle on April 26-27, 2008. This workshop was organized by Herbert Sauro, Sean Sleight, and Deepak Chandran, and included talks by Raik Gruenberg, Kim de Mora, John Cumbers, Christopher Anderson, Mac Cowell, Jason Morrison, Jean Peccoud, Ralph Santos, Andrew Milar, Vincent Rouilly, Mike Hucka, Michael Blinov, Lucian Smith, Sarah Richardson, Guillermo Rodrigo, Jonathan Goler, and Michal Galdzicki.

Michal's early efforts were instrumental in making SBOL successful. As part of his doctoral work, Mike led the development of PoBol, as SBOL was originally known. He organized annual workshops from 2008 to 2011 and kept the idea of developing a genetic design standard alive. The original SBOL 1.0 was developed by a small group of dedicated researchers calling themselves the Synthetic Biology Data Exchange Working Group, meeting at Stanford in 2009 and Anaheim, CA in 2010. During the Anaheim meeting, the community decided to write a letter to Nature Biotechnology highlighting the issue of reproducibility in synthetic biology. This letter was initiated by Jean Peccoud and submitted by participants of the Anaheim meeting, including Deepak Chandran, Douglas Densmore, Dmytriv, Michal Galdzicki, Timothy Ham, Cesar Rodriquez, Jean Peccoud, Herbert Sauro, and Guy-Bart Stan. The overall pace of development quickened when several new members joined at the next workshop in Blacksburg, Virginia on January 7-10, 2011. This early work was also supported by an STTR grant from the National Institute of Health (NIH #1R41LM010745 and #9R42HG006737, from 2010-13) in collaboration with Clark & Parsia, LLC (Co-PIs: John Gennari and Evren Sirin). New members included Cesar Rodriguez, Mandy Wilson, Guy-Bart Stan, Chris Myers, and Nicholas Roehner.

The SBOL Developers Group was officially established at a meeting in San Diego in June 2011. Rules of governance were established, and the first SBOL editors were elected: Mike Galdzicki, Cesar Rodriguez, and Mandy Wilson. At our next meeting in Seattle in January 2012, Herbert Sauro was elected the SBOL chair, and two new editors were added: Matthew Pocock and Ernst Oberortner. New developers joining at these workshops included several representatives from industry, Kevin Clancy, Jacob Beal, Aaron Adler, and Fusun Yaman Sirin. New members hailing from Newcastle University included Anil Wipat, Matthew Pocock, and Goksel Misirli.

Development of the first software library (libSBOLj) based on the SBOL standard was initiated by Allan Kuchinsky, a research scientist from Agilent, at the 2011 meeting. By the time of the 2012 meeting, the first data exchange between software tools using SBOL was conducted when a design was passed from Newcastle University's VirtualParts Repository to Boston University's Eugene tool, and finally to University of Utah's iBioSim tool.

SBOL 1.0 was officially released in October 2011. In March 2012, SBOL 1.1 was released, the version that this document replaces. SBOL 1.1 did not make any major changes, but provided a number of small adjustments and clarifications, particularly around the annotation of sequences. Multi-institutional data exchange using SBOL 1.1 was later demonstrated in Nature Biotechnology Galdzicki et al. (2014).

While SBOL 1.1 had a number of significant advantages over the GenBank representation of DNA sequences, such as representing hierarchical organization of DNA components, it was still limited in other respects. The major topic of discussion at the 8th SBOL Workshop at Boston University in November 2012 was how to address these shortcomings through extensions. Several extensions were discussed at this meeting, such as a means to describe genetic regulation, which later became important classes in the current 2.x specification.

A general framework for SBOL 2.0 emerged at the 9th SBOL workshop at Newcastle University in April 2013. Subsequently, Nicholas Roehner, Matthew Pocock, and Ernst Oberortner drafted a proposal for SBOL 2.0, and Nicholas presented this proposal at the SEED conference in Los Angeles in July 2014 Roehner et al. (2015). The proposed 2.0 data model was discussed over the course of the 10th, 11th, and 12th workshops. The actual specification document you are now reading was drafted at the 13th workshop in Wittenberg, Germany by the authors. The SBOL 2.x data model presented here is essentially the result of these meetings and ongoing discussions conducted through the SBOL Developers mailing lists, plus minor adjustments and updates approved by the community through subsequence meetings and mailing list discussions.

The Computational Modeling in Biology Network (COMBINE) holds regular workshops where synthetic biologists and systems biologists can work toward a common goal of integrating biological knowledge through inter-operable and non-overlapping data standards. In April of 2014, several SBOL Developers attended a COMBINE workshop and then proposed that SBOL join this larger standards community. The proposal passed and SBOL workshops have been co-located with COMBINE meetings since the 11th workshop at the University of Southern California in August 2014.

Current development of this SBOL 2.x specification is funded in large part by a grant from the National Science Foundation (DBI-1355909 and DBI-1356041). This document and the supporting software libraries are due in no small part to this support. Any opinions, findings, and conclusions or recommendations expressed in this material are those of the author(s) and do not necessarily reflect the views of the National Science Foundation.

## 5 SBOL Specification Vocabulary

### 5.1 Term Conventions

This document indicates requirement levels using the controlled vocabulary specified in IETF RFC 2119 and reiterated in BBF RFC 0. In particular, the key words "MUST", "MUST NOT", "REQUIRED", "SHALL", "SHALL NOT", "SHOULD", "SHOULD NOT", "RECOMMENDED", "MAY", and "OPTIONAL" in this document are to be interpreted as described in RFC 2119.

- The words "MUST", "REQUIRED", or "SHALL" mean that the item is an absolute requirement.
- The phrases "MUST NOT" or "SHALL NOT" mean that the item is an absolute prohibition.
- The word "SHOULD" or the adjective "RECOMMENDED" mean that there might exist valid reasons in particular circumstances to ignore a particular item, but the full implications need to be understood and carefully weighed before choosing a different course.
- The phrases "SHOULD NOT" or "NOT RECOMMENDED" mean that there might exist valid reasons in particular circumstances when the particular behavior is acceptable or even useful, but the full implications needs to be understood and the case carefully weighed before implementing any behavior described with this label.
- The word "MAY" or the adjective "OPTIONAL" mean that an item is truly optional.

### 5.2 SBOL Class Names

SBOL defines the following "top-level" and dependent classes:

*Collection*: Represents a user-defined container for organizing a group of SBOL objects.

*ComponentDefinition*: Describes the structure of designed entities, such as DNA, RNA, and proteins, as well as other entities they interact with, such as small molecules or environmental properties.

- *Component*: Pointer class. Incorporates a child ComponentDefinition *by reference* into exactly one parent ComponentDefinition. Represents a specific occurrence or instance of an entity within the design of a more complex entity. Because the same definition might appear in multiple designs or multiple times in a single design, a single ComponentDefinition can have zero or more parent ComponentDefinitions, and each such parent-child link requires its own, distinct Component.
- *Location*: Specifies the base coordinates and orientation of a genetic feature on a DNA or RNA molecule or a residue or site on another sequential macromolecule such as a protein.
- *SequenceAnnotation*: Describes the Location of a notable sub-sequence found within the Sequence of a ComponentDefinition. Can also link to and effectively position a child Component.
- *SequenceConstraint*: Describes the relative spatial position and orientation of two Component objects that are contained within the same ComponentDefinition.

*GenericTopLevel*: Represents a data container that can contain custom data added by user applications.

*Model*: Links to quantitative or qualitative computational models that might be used to predict the functional behavior of a biological design.

*ModuleDefinition*: Describes a "system" design as a collection of biological components and their functional relationships.

- *FunctionalComponent*: Pointer class. Incorporates a child ComponentDefinition *by reference* into exactly one parent ModuleDefinition. Represents a specific occurrence or instance of an entity within the design of a system. Because the same definition might appear in multiple designs or multiple times in a single design, a single ComponentDefinition can have zero or more parent ModuleDefinitions, and each such parent-child link requires its own, distinct FunctionalComponent.

- *Interaction*: Describes a functional relationship between biological entities, such as regulatory activation or repression, or a biological process such as transcription or translation.
- *MapsTo*: When a design (ComponentDefinition or ModuleDefinition) includes another design as a sub-design, the parent design might need to refer to a ComponentInstance (either a Component or FunctionalComponent) in the sub-design. In this case, a MapsTo needs to be added to the instance for the sub-design, and this MapsTo needs to link between the ComponentInstance in the sub-design and a ComponentInstance in the parent design.
- *Module*: Pointer class. Incorporates a child ModuleDefinition *by reference* into exactly one parent ModuleDefinition. Represents a specific occurrence or instance of a subsystem within the design of a larger system. Because the same definition might appear in multiple designs or multiple times in a single design, a single ModuleDefinition can have zero or more parent ModuleDefinitions, and each such parent-child link requires its own, distinct Module.
- *Participation*: Describes the role that a FunctionalComponent plays in an Interaction. For example, a transcription factor might participate in an Interaction as a repressor or as an activator.

*Sequence*: Generally represents a contiguous series of monomers in a macromolecular polymer such as DNA, RNA, or protein. A Sequence can also encode the atoms and bonds of a molecule with non-linear structure (see Section 7.6).

## 6 Overview of SBOL

Synthetic biology designs can be described using:

- Structural terms, e.g., a set of annotated sequences or information about the chemical makeup of components.
- Functional terms, e.g., the way that components might interact with each other and the overall behavior of a design.

In broad strokes, the prior SBOL 1.1 standard focused on conveying physical, structural information, whereas SBOL 2 expands the scope to include functional aspects as well. The physical information about a designed genetic construct includes the order of its constituents and their descriptions. Specifying the exact locations of these constituents and their sequences allow genetic constructs to be defined unambiguously and reused in other designs. SBOL 2 extends SBOL 1.1 in several ways: it extends physical descriptions to include entities beyond DNA sequences, and it supports functional descriptions of designs.

As an example, consider the design of an expression cassette, such as the one found in the plasmid pUC18 Norrander et al. (1983). This device is designed to detect successful versus unsuccessful molecular cloning. As an overall system, the device is designed to grow either blue-colored (unsuccessful) or white-colored (successful) colonies in the presence of IPTG and the chemical X-gal. Internally, the device has a number of parts, including a promoter, the lac repressor binding site, and the lacZ coding sequence. These parts have specific component-level interactions with IPTG and X-gal, as well as native host gene products, transcriptional machinery and translational machinery that collectively cause the desired system-level behavior.

Knowledge of how such a device functions within the context of a host and how it might be adapted to new experimental applications has generally been passed on through working with fellow scientists or reading articles in papers and books. But there has been no systematic way to communicate the integration of sequences with functional designs, so users typically have had to look in many different places to develop an understanding of a system. The SBOL 2 standard allows designers to describe these functional characteristics and connect them to the physical parts and sequences that make up the design.

SBOL 2 includes two main classes that match the structural/functional distinction above:

- The `ComponentDefinition` object describes the physical aspects of the designed system, such as its DNA or RNA sequences, and the physical relationships among sub-components, as when one sequence contains another as a sub-sequence.
- The `ModuleDefinition` object describes interactions of the designed system, such as specific binding relationships and repression and activation relationships.

Figure 2 shows a simplified view of these classes, as well as other helper classes in SBOL. To continue with the pUC18 example, the description would begin with a top-level `ModuleDefinition`. The `ModuleDefinition` specifies the structural elements that make up the cassette by referencing a number of `ComponentDefinition` objects. These would include the DNA component for the promoter and the small molecule component for IPTG, for example. The `ComponentDefinition` objects can be organized hierarchically. For example, the plasmid `ComponentDefinition` might reference `ComponentDefinition`s for the promoter, coding sequence, etc. Each `ComponentDefinition` object can also include the actual `Sequence` information (if available), as well as `SequenceAnnotation` objects that identify the locations of the promoters, coding sequences, etc., on the `Sequence`. In order to specify functional information, the `ModuleDefinition` can specify `Interaction` objects that describe any qualitative relationships among components, such as how IPTG and X-gal interact with the gene products. Finally, a `ModuleDefinition` object can point to a `Model` object that provides a reference to a complete computational model using a language such as SBML Hucka et al. (2003), CellML Cuellar et al. (2003), MATLAB MathWorks (2015), etc. Finally, all the of elements of the genetic design can be grouped together within a `Collection`.

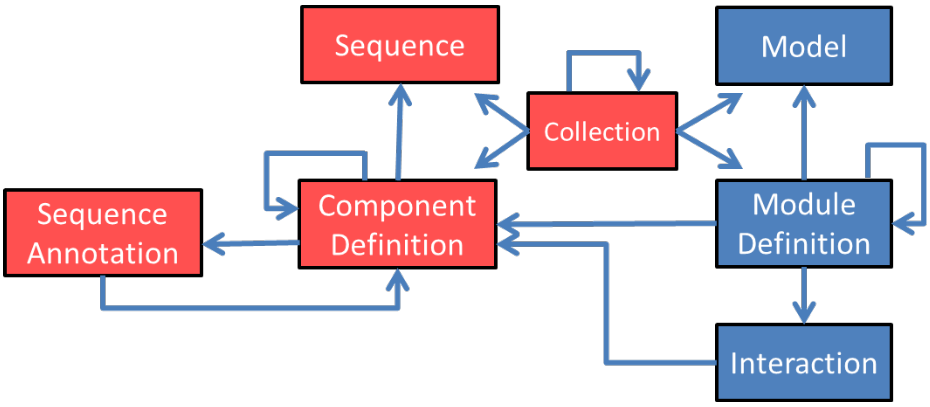

***Figure 2:*** *Main classes of information represented by the SBOL standard, and their relationships. Red boxes are classes from the SBOL 1.1 that focused on structure, whereas blue classes are some of the new classes that support the functional aspects of designs.*

Whereas Figure 2 provides a broad overview of SBOL, Figure 3 provides a detailed, implementation-level overview of the class structure for the SBOL 2.x data model. This figure relies on the semantics of the *Unified Modeling Language* (UML), which will be presented in more detail in the next section. Figure 3 distinguishes between *top level* classes, in green, and other supporting classes (note that Figure 2 also includes all of the top level classes). In Figure 3, dashed arcs represent "refersTo", whereas a solid arrow represents ownership. In UML, the meaning of ownership is that if a parent class is deleted, so are all of its owned children. Thus, a `Collection` does not own its `ComponentDefinition` objects, because these can stand on their own. All of the supporting classes (in orange) have to be owned by some top-level class, directly or indirectly.

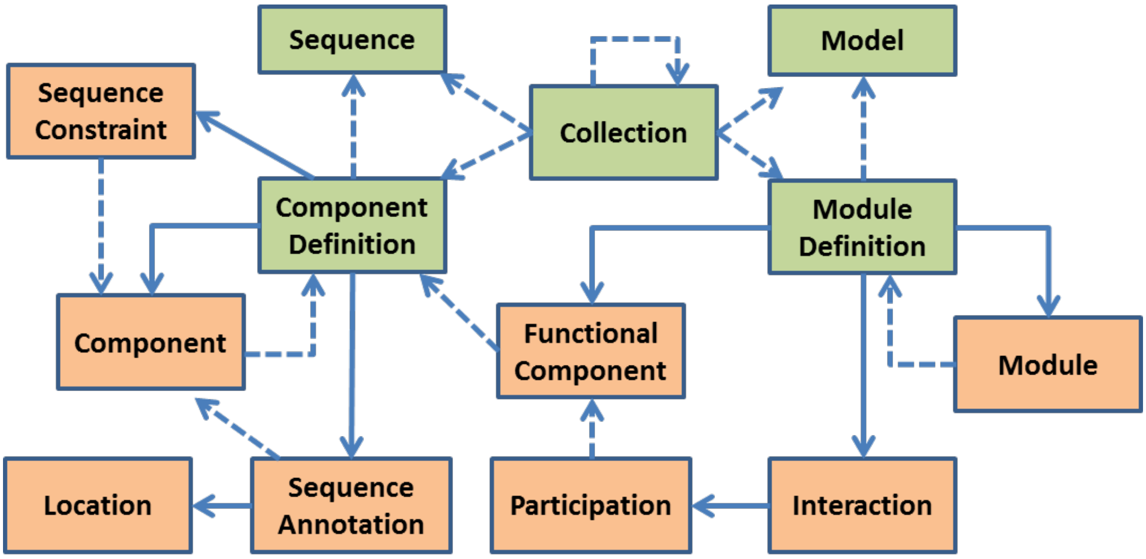

***Figure 3:*** *Main classes of information represented by the SBOL 2.x standard, and their relationships. Green boxes are "top level" classes, while the other classes are in support of these classes. Solid arrows indicates ownership, whereas a dashed arrow indicates that one class refers to an object of another class.*

Figure 3 additionally shows that when it is possible to incorporate a single object into multiple parents, we always incorporate that object by reference. We do not directly incorporate it by copy, because when an object is used many times, keeping many copies becomes spatially inefficient and difficult to maintain. Instead, each reference is handled by a pointer object. Pointers refer from a parent to a child. There are three distinct pointer classes: Component, Module, and FunctionalComponent. A Component points from a ComponentDefinition to a child ComponentDefinition, incorporating it by reference into the parent structure. A Module points from a ModuleDefinition to a child ModuleDefinition, likewise incorporating the child by reference into the parent system. Similarly, a parent ModuleDefinition on the functional side of a model might incorporate a child ComponentDefinition from the model's physical side by means of a FunctionalComponent reference. These three pointer classes allow the efficient reuse of definitions in multiple locations.

SBOL 2.x provides a few helper classes. Location generalizes the positioning information from SBOL 1.1 to allow discontinuous ranges and cuts to be annotated. SequenceConstraint generalizes the relative positioning information among Components. There are also Participations, which allow Interaction objects to specify the roles of their participants while referencing the FunctionalComponents, so that these can stand on their own. Additionally, there is the MapsTo class (not shown), which enables connections to be made between Components and FunctionalComponents across various levels of the design hierarchy. The next section provides complete definitions and details for all of these classes.

There is one final, critical element of SBOL 2: its extension mechanism. This extension mechanism enables the storage of application specific information within an SBOL document. It is also intended to support the prototyping of data representations whose format is not yet a matter of consensus within the community. In particular, each SBOL entity can be annotated using the *Resource Description Framework* (RDF). Moreover, application specific entities in the form of RDF documents can be included as GenericTopLevel entities. SBOL libraries make these annotations and entities available to tools as generic properties and objects that are preserved during subsequent read and write operations.

## 7 SBOL Data Model

In this section, we describe the types of biological design data that can belong to an SBOL document and the relationships between these data types. The SBOL data model is specified using Unified Modeling Language (UML) 2.0 diagrams (OMG 2005). Subsections Section 7.1, Section 7.2, Section 7.3 review the basics of UML diagrams and explain the naming conventions and generic data types used in this specification. The remaining sections then describe the SBOL data model in detail. Complete SBOL examples and best practices when using the standard can be found in Section 9 and Section 12, respectively.

### 7.1 Understanding the UML Diagrams

The types of biological design data modeled by SBOL are commonly referred to as *classes*, especially when discussing the details of software implementation. Each SBOL class can be instantiated by many SBOL objects. These objects MAY contain data that differ in content, but they MUST agree on the type and form of their data as dictated by their common class. Classes are represented in UML diagrams as rectangles labeled at the top with class names.

Classes can be connected to other classes by association properties, which are represented in UML diagrams as arrows. These arrows are labeled with data cardinalities in order to indicate how many values a given association property can possess (see below). The remaining (non-association) properties of a class are listed below its name. Each of the latter properties is labeled with its data type and cardinality.

In the case of an association property, the class from which the arrow originates is the owner of the association property. A diamond at the origin of the arrow indicates the type of association. Open-faced diamonds indicate shared aggregation, in which the owner of the association property exists independently of its value. In the SBOL data model, the value of an association property MUST be a `URI` or set of `URI`s that refer to SBOL objects belonging to the class at the tip of the arrow.

By contrast, filled diamonds indicate composite aggregation, also known as a part-whole relationship, in which the value of the association property MUST NOT exist independently of its owner. In addition, in the SBOL data model, it is REQUIRED that the value of each composite aggregation property is a unique SBOL object (that is, not the value for more than one such property). Note that in all cases, composite aggregation is used in such a way that there SHOULD NOT be duplication of such objects.

All SBOL properties are labeled with one of several restrictions on data cardinality. These are:

- 1 - REQUIRED, one: there MUST be exactly one value for this property.
- $0 \ldots 1$ - OPTIONAL: there MAY be a single value for this property, or it MAY be absent.
- $0 \ldots *$ - unbounded: there MAY be any number of values for this property, including none.
- $1 \ldots *$ - REQUIRED, unbounded: there MAY be any number of values for this property, as long as there is at least one.
- $n \ldots *$ - at least: there MUST be at least $n$ values for this property.

Finally, classes can inherit the properties of other classes. Inheritance relationships are represented in UML diagrams as open-faced, triangular arrows that point from the inheriting class to the inherited class. Some classes in the SBOL data model cannot be instantiated as objects and exist only to group common properties for inheritance. These classes have italicized names and are known as abstract classes.

### 7.2 Naming and Font Conventions

SBOL classes are named using upper "camel case," meaning that each word is capitalized and all words are run together without spaces, e.g. `Identified`, `SequenceAnnotation`. Properties, on the other hand, are named using lower camel case, meaning that they begin lowercase (e.g., `identity`) but if they consist of multiple words, all words after the first begin with an uppercase letter (e.g., `persistentIdentity`).

Within the SBOL data model, each property is given a singular or plural name in accordance with its data cardinalities. The forms of these names follow the usual rules of English grammar. For example, sequenceAnnotation is the singular form of `sequenceAnnotations`.

SBOL properties are always given singular names, however, when SBOL objects are serialized (using *Resource Description Framework* (RDF) as described in Section 10). This is because the SBOL data model does not contain classes that correspond directly to the RDF elements that group other elements into ordered or unordered sets. Consequently, if an SBOL property has multiple values, then it is serialized as multiple property entries, each with a singular name and a single value. For example, if an SBOL property has five values, then its serialization contains five RDF triples, each with a singular predicate name and one of the five values as its object.

### 7.3 Data Types

When SBOL use simple "primitive" data types such as `String`s or `Integer`s, these are defined as the following specific formal types:

- `String`: `http://www.w3.org/TR/xmlschema11-2/#string`
  *Example: "LacI coding sequence"*
- `Integer`: `http://www.w3.org/TR/xmlschema11-2/#integer`
  *Example: 3*
- 2.2.0 `Long`: `http://www.w3.org/TR/xmlschema11-2/#long`
  *Example: 9223372036854775806*
- 2.2.0 `Double`: `http://www.w3.org/TR/xmlschema11-2/#double`
  *Example: 3.14159*
- `Boolean`: `http://www.w3.org/TR/xmlschema11-2/#boolean`
  *Example:* `true`

The term `literal` is used to denote an object that can be any of the four types listed above. In addition to the simple types listed above, SBOL also uses objects with types *Uniform Resource Identifier* (`URI`) and *XML Qualified Name* (`QName`):

- `URI`: `http://www.w3.org/TR/xmlschema11-2/#anyURI`
  *Example:* `http://www.partsregistry.org/Part:BBa_J23119`
- `QName`: `http://www.w3.org/TR/xmlschema11-2/#QName`
  *Example:* `myapp:Datasheet` where `myapp="http://www.myapp.org/"`

Note that, in compliance with RDF standards, `URI`s are generally serialized using an `rdf:resource` property, e.g.: `rdf:resource="http://www.partsregistry.org/Part:BBa_J23119"`

It is important to realize that in RDF, a `URI` might or might not be a resolvable URL (web address). A `URI` is always a globally unique identifier within a structured namespace. In some cases, that name is also a reference to (or within) a document, and in some cases that document can also be retrieved (e.g., using a web browser).

### 7.4 Identified

All SBOL-defined classes are directly or indirectly derived from the `Identified` abstract class. This inheritance means that all SBOL objects are uniquely identified using `URI`s that uniquely refer to these objects within an SBOL document or at locations on the World Wide Web.

As shown in Figure 4, the Identified class includes the following properties: identity, persistentIdentity, version, wasDerivedFroms , name, description, and annotations. The latter property is described separately in
2.2.0 Section 7.16.

When an SBOL resource reference takes the form of a URI, that URI can either be the value of an identity property or the value of a persistentIdentity property. If the URI is equal to the value of an identity property, then it is guaranteed to be unique, and it refers to precisely one SBOL object with that URI. If the URI is equal to the value of a persistentIdentity property, then it MAY refer to multiple SBOL objects that are different "versions" of each other. These objects SHOULD be compared to one another to determine which single object the URI resolves to (normally the most recent version - see Section 7.4). Throughout this document, when a URI is used to refer to an SBOL object, it could fall into either of these cases.

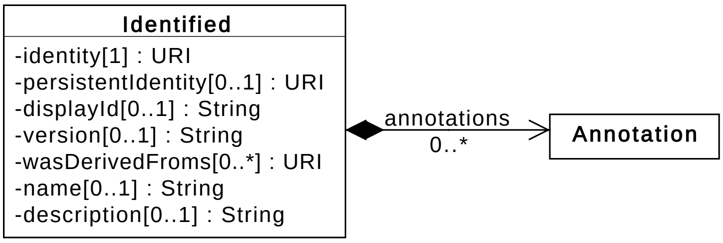

*Figure 4: Diagram of the Identified abstract class and its associated properties*

#### *The identity property*

The identity property is REQUIRED by all Identified objects and has a data type of URI. A given Identified object's identity URI MUST be globally unique among all other identity URIs. It is also highly RECOMMENDED that the URI structure follows the recommended best practices for compliant URIs specified in Section 12.3.

Although most SBOL properties are defined by SBOL and serialized with its namespace, the identity property is defined by the analogous RDF about property and is serialized with the RDF namespace as follows:

http://www.w3.org/1999/02/22-rdf-syntax-ns#about.

The use of about is expressly for the purpose of making SBOL compliant with pre-existing standards: when you see about in an SBOL document, you SHOULD interpret it as meaning identity.

#### *The persistentIdentity property*

The persistentIdentity property is OPTIONAL and has a data type of URI. This URI serves to uniquely refer to a set of SBOL objects of the same class that are different versions of each other.

2.0.1 An Identified object MUST be referred to using either its identity URI or its persistentIdentity URI.

#### *The displayId property*

The displayId property is an OPTIONAL identifier with a data type of String. This property is intended to be an intermediate between name and identity that is machine-readable, but more human-readable than the full URI of an identity.

If the displayId property is used, then its String value ~~SHOULD be locally unique (global uniqueness is not~~
2.0.1 ~~necessary) and~~ MUST be composed of only alphanumeric or underscore characters and MUST NOT begin with a digit.

#### The `version` property

The `version` property is OPTIONAL and has a data type of `String`. This property can be used to compare two SBOL objects with the same `persistentIdentity`.

If the `version` property is used, then it is RECOMMENDED that version numbering follow the conventions of semantic versioning (`http://semver.org/`), particularly as implemented by Maven (`http://maven.apache.org/`). This convention represents versions as sequences of numbers and qualifiers that are separated by the characters "`.`" and "`-`" and are compared in lexicographical order (for example, 1 < 1.3.1 < 2.0-beta). For a full explanation, see the linked resources.

#### The `wasDerivedFroms` property

2.2.0 The `wasDerivedFroms` property is OPTIONAL and MAY specify a set of `URI`s. An SBOL object with this property refers to one or more SBOL objects or non-SBOL resources from which this object was derived.

2.0.1 The `wasDerivedFroms` property of a `TopLevel` SBOL object is subject to the following rules. If any members of the `wasDerivedFroms` property of an SBOL object *A* that refers to an SBOL object *B* has an identical `persistentIdentity`, and both *A* and *B* have a `version`, then the `version` of *B* MUST precede that of *A*. In addition, an SBOL object MUST NOT refer to itself via its own `wasDerivedFroms` property or form a cyclical chain of references via its `wasDerivedFroms` property and those of other SBOL objects. For example, the reference chain "*A* was derived from *B* and *B* was derived from *A*" is cyclical.

#### The `name` property

The `name` property is OPTIONAL and has a data type of `String`. This property is intended to be displayed to a human when visualizing an `Identified` object.

If an `Identified` object lacks a name, then software tools SHOULD instead display the object's `displayId` or `identity`. It is RECOMMENDED that software tools give users the ability to switch perspectives between `name` properties that are human-readable and `displayId` properties that are less human-readable, but are more likely to be unique.

#### The `description` property

The `description` property is OPTIONAL and has a data type of `String`. This property is intended to contain a more thorough text description of an `Identified` object.

#### The `annotations` property

The `annotations` property is OPTIONAL and MAY specify a set of `Annotation` objects that are contained by the `Identified` object. The `Annotation` class is described in more detail in Section Section 7.16.1.

#### Serialization

No complete serialization is defined for `Identified`, since this class is only used indirectly through its child classes. Any such child class, however, has the following form for serializing properties inherited from `Identified`, where CLASS_NAME is replaced by the name of the class:

```
<?xml version="1.0" ?>
<rdf:RDF xmlns:pr="http://partsregistry.org" xmlns:rdf="http://www.w3.org/1999/02/22-rdf-syntax-ns#" xmlns:dcterms="http://purl.
     org/dc/terms/" xmlns:prov="http://www.w3.org/ns/prov#" xmlns:sbol="http://sbols.org/v2#">
  <sbol:CLASS_NAME rdf:about="...">
    zero or one <sbol:persistentIdentity rdf:resource="..."/> element
    zero or one <sbol:displayId>...</sbol:displayId> element
    zero or one <sbol:version>...</sbol:version> element
    zero or more <prov:wasDerivedFrom rdf:resource="..."/> element
    zero or one <dcterms:title>...</dcterms:title> element
    zero or one <dcterms:description>...</dcterms:description> element
```

```
            ...
  </sbol:CLASS_NAME>
  ...
</rdf:RDF>
```

Note that several of the properties are not in the sbol namespace, but are mapped to standardized terms defined elsewhere:

- identity is serialized as rdf:about
- 2.2.0 wasDerivedFroms are serialized as prov:wasDerivedFrom
- name is serialized as dcterms:title
- description is serialized as dcterms:description

### 7.5 TopLevel

TopLevel is an abstract class that is extended by any Identified class that can be found at the top level of an SBOL document or file. In other words, TopLevel objects are not nested inside any other object via a composite aggregation or black diamond arrow association property. Instead of nesting, composite TopLevel objects refer to subordinate TopLevel objects by their URIs using shared aggregation or white diamond arrow association properties. The TopLevel classes defined in this specification are Sequence, ComponentDefinition, Model, ModuleDefinition, Collection, GenericTopLevel, CombinatorialDerivation, and Implementation(Figure 5).

2.2.1

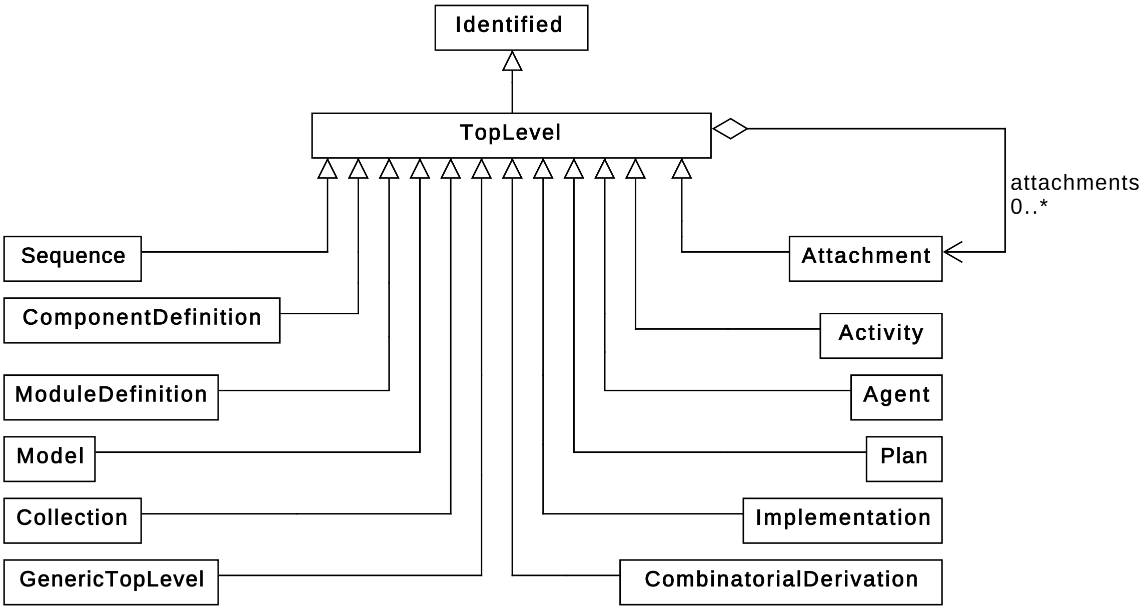

***Figure 5:*** *Classes that inherit from the TopLevel abstract class.*

#### *The attachments property*

The attachments property is OPTIONAL and MAY specify a set of Attachment objects that are referenced by the TopLevel object. The Attachment class is described in more detail in Section Section 7.13.

#### *Serialization*

No serialization is defined for TopLevel, since this class has no properties of its own and is only used indirectly through its child classes. All TopLevel classes are serialized one level beneath the RDF document root.

### 7.6 Sequence

The purpose of the Sequence class is to represent the primary structure of a ComponentDefinition object and the manner in which it is encoded. This representation is accomplished by means of the elements property and encoding property (Figure 6).

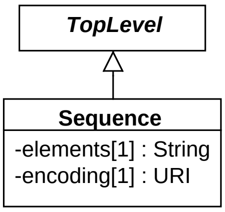

***Figure 6:*** *Diagram of the Sequence class and its associated properties.*

#### *The `elements` property*

The elements property is a REQUIRED String of characters that represents the constituents of a biological or chemical molecule. For example, these characters could represent the nucleotide bases of a molecule of DNA, the amino acid residues of a protein, or the atoms and chemical bonds of a small molecule.

#### *The `encoding` property*

The encoding property is REQUIRED and has a data type of URI. This property MUST indicate how the elements property of a Sequence MUST be formed and interpreted.

For example, the elements property of a Sequence with an `IUPAC DNA` encoding property MUST contain characters that represent nucleotide bases, such as `a`, `t`, `c`, and `g`. The elements property of a Sequence with a `Simplified Molecular-Input Line-Entry System (SMILES)` encoding, on the other hand, MUST contain characters that represent atoms and chemical bonds, such as `C`, `N`, `O`, and `=`.

Table 1 provides a list of possible URI values for the encoding property. The terms in Table 1 are organized by the type of ComponentDefinition (see Table 2) that typically refer to a Sequence with such an encoding. It is
2.0.1 RECOMMENDED that the encoding property of a Sequence contains a URI from Table 1. When the encoding of a
Sequence is well described by one of the URIs in Table 1, it MUST contain that URI.

More information on IUPAC encoding can be found at http://www.bioinformatics.org/sms2/iupac.html.

| Encoding | URI | ComponentDefinition Type |
|---|---|---|
| IUPAC DNA, RNA | http://www.chem.qmul.ac.uk/iubmb/misc/naseq.html | DNA, RNA |
| IUPAC Protein | http://www.chem.qmul.ac.uk/iupac/AminoAcid/ | Protein |
| SMILES | http://www.opensmiles.org/opensmiles.html | SmallMolecule |

***Table 1:*** *URIs for specifying the encoding property of a Sequence, organized by the type of ComponentDefinition (see Table 2) that typically refer to a Sequence with such an encoding.*

#### Serialization

The serialization of a Sequence MUST have the following form:

```
<sbol:Sequence rdf:about="...">
  ... properties inherited from identified ...
  one <sbol:elements>...</sbol:elements> element
  one <sbol:encoding rdf:resource="..."/> element
</sbol:Sequence>
```

The example below shows the serialization of the Sequence for a promoter. The nucleotide bases of the Sequence are serialized as the String value of its elements property, while its IUPAC DNA encoding is serialized as the URI value of its encoding property.

```
<?xml version="1.0" ?>
<rdf:RDF xmlns:rdf="http://www.w3.org/1999/02/22-rdf-syntax-ns#" xmlns:dcterms="http://purl.org/dc/terms/" xmlns:prov="http://www.
     w3.org/ns/prov#" xmlns:sbol="http://sbols.org/v2#">
  <sbol:Sequence rdf:about="http://partsregistry.org/seq/BBa_J23119">
    <sbol:persistentIdentity rdf:resource="http://partsregistry.org/seq/BBa_J23119"/>
    <sbol:displayId>BBa_J23119</sbol:displayId>
    <prov:wasDerivedFrom rdf:resource="http://parts.igem.org/Part:BBa_J23119:Design"/>
    <sbol:elements>ttgacagctagctcagtcctaggtataatgctagc</sbol:elements>
    <sbol:encoding rdf:resource="http://www.chem.qmul.ac.uk/iubmb/misc/naseq.html"/>
  </sbol:Sequence>
</rdf:RDF>
```

### 7.7 ComponentDefinition

The ComponentDefinition class represents the structural entities of a biological design. The primary usage of this class is to represent structural entities with designed sequences, such as DNA, RNA, and proteins, but it can also be used to represent any other entity that is part of a design, such as small molecules, molecular complexes, and light.

As shown in Figure 7, the ComponentDefinition class describes a structural design entity using the following properties: types, roles, and sequences. In addition, this class has properties for describing and organizing the substructure of said design entity, including components, sequenceAnnotations, and sequenceConstraints.

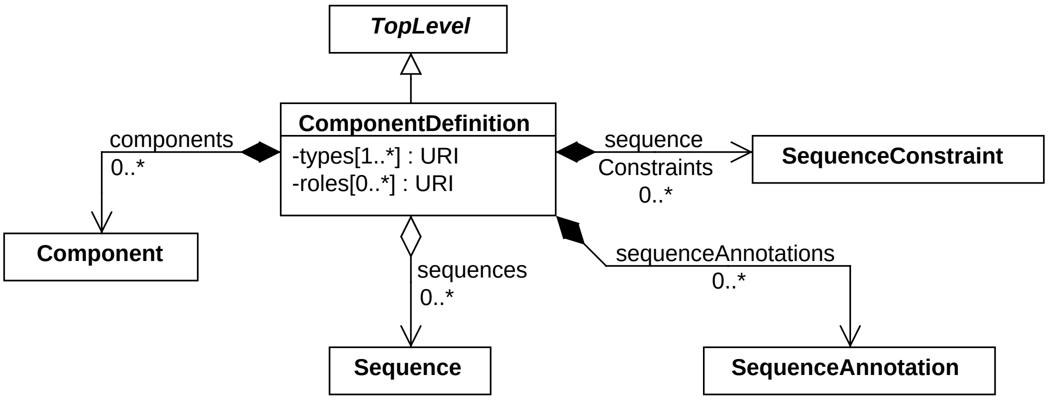

***Figure 7:*** *Diagram of the ComponentDefinition class and its associated properties.*

#### *The `types` property*

The `types` property is a REQUIRED set of `URI`s that specifies the category of biochemical or physical entity (for example DNA, protein, or small molecule) that a `ComponentDefinition` object abstracts for the purpose of engineering design. For DNA or RNA entities, additional `types` fields are used to describe nucleic acid topology (circular / linear) and strandedness (double- or single-stranded).

The `types` property of every `ComponentDefinition` MUST contain one or more `URI`s that MUST identify terms from appropriate ontologies, such as the Biological Pathway Exchange (BioPAX) ontology Demir et al. (2010) or the ontology of Chemical Entities of Biological Interest (ChEBI) Degtyarenko et al. (2008). Table 2 provides a list of possible ontology terms for the `types` property and their `URI`s. In order to maximize the compatibility of designs, the
2.0.1 `types` property of a `ComponentDefinition` SHOULD contain a `URI` from Table 2, and any `ComponentDefinition` that can be well-described by one of the terms in Table 2 MUST use the `URI` for that term as one of its `types`. Finally, if the `types` property contains multiple `URI`s, then they MUST identify non-conflicting terms (otherwise, it might not be clear how to interpret them). For example, the BioPAX terms provided by Table 2 would conflict because they specify classes of biochemical entities with different molecular structures.

| ComponentDefinition Type | URI for BioPAX Term |
|---|---|
| DNA Molecule | `http://www.biopax.org/release/biopax-level3.owl#Dna` |
| DNA Region | `http://www.biopax.org/release/biopax-level3.owl#DnaRegion` |
| RNA Molecule | `http://www.biopax.org/release/biopax-level3.owl#Rna` |
| RNA Region | `http://www.biopax.org/release/biopax-level3.owl#RnaRegion` |
| Protein | `http://www.biopax.org/release/biopax-level3.owl#Protein` |
| Small Molecule | `http://www.biopax.org/release/biopax-level3.owl#SmallMolecule` |
| Complex | `http://www.biopax.org/release/biopax-level3.owl#Complex` |

***Table 2:*** *BioPAX terms to specify the molecule type using the `types` property of a `ComponentDefinition`.*

2.1.0 *Nucleic Acid Topology types*

[New in 2.1.0; see SEP 011: `https://github.com/SynBioDex/SEPs/blob/master/sep_011.md`]

Any `ComponentDefinition` classified as DNA (see Table 2) is RECOMMENDED to encode circular/linear topology information in an additional type field. This (topology) type field SHOULD specify a URI from the Topology Attribute branch of the SO (this is currently just 'linear' or 'circular' as given in Table 3). Topology information SHOULD be specified for DNA `ComponentDefinition` records with a fully specified sequence, except in three scenarios: if the DNA record doesn't have sequence information, or if the DNA record has incomplete sequence information, or if topology is genuinely unknown. For any `ComponentDefinition` classified as RNA (see Table 2), a topology type field is OPTIONAL. The default assumption in this case is linear topology. In any case, no more than one topology should be specified.

Any `ComponentDefinition` classified as DNA or RNA MAY also have strand information encoded in an additional (third) type field using a URI from the Strand Attribute branch of the SO (currently there are only two possible terms for single or double-stranded nucleic acids given in Table 3). In absence of this field, the default strand information assumed for DNA is 'double-stranded' and for RNA is 'single-stranded'.

Any other type of `ComponentDefinition` record (protein, small molecule, etc) SHOULD NOT have any type field pointing to SO terms from the topology or strand attribute branches of SO.

Note that a *circular* topology instructs software to interpret the beginning / end position of a given sequence (be it DNA or RNA) as arbitrary so that sequence features may be mapped or identified across this junction. *Double stranded* instructs software to apply sequence searches to both strands (i.e. sequence and reverse complement of sequence).

| Nucleic Acid Topology | URI for Nucleic Acid Topology Term in SO |
|---|---|
| linear | http://identifiers.org/so/SO:0000987 |
| circular | http://identifiers.org/so/SO:0000988 |
| single-stranded | http://identifiers.org/so/SO:0000984 |
| double-stranded | http://identifiers.org/so/SO:0000985 |

***Table 3:*** *Sequence Ontology terms to encode DNA or RNA topology information in the `types` properties of a `ComponentDefinition`.*

#### *The `roles` property*

The `roles` property is an OPTIONAL set of `URI`s that clarifies the potential function of the entity represented by a `ComponentDefinition` in a biochemical or physical context.

The `roles` property of a `ComponentDefinition` MAY contain one or more `URI`s that MUST identify terms from ontologies that are consistent with the `types` property of the `ComponentDefinition`. For example, the `roles` property of a DNA or RNA `ComponentDefinition` could contain URIs identifying terms from the Sequence Ontology (SO). As a best practice, a DNA or RNA `ComponentDefinition` SHOULD contain exactly one `URI` that refers to a
2.0.1 term from the sequence feature branch of the SO. Similarly, the roles property of a Protein and SmallMolecule `ComponentDefinition` SHOULD respectively contain `URI`s identifying terms from the `MolecularFunction` branch
2.2.1 (`GO:0003674`) of the Gene Ontology (GO) and the `role` branch (`CHEBI:50906`) of the CHEBI ontology. Table 4 contains a list of possible ontology terms for the `roles` property and their `URI`s. These terms are organized by the type of `ComponentDefinition` to which they SHOULD apply (see Table 2). Any `ComponentDefinition` that can be well-described by one of the terms in Table 4 MUST use the `URI` for that term as one of its `roles`.

| ComponentDefinition Role | URI for Ontology Term | ComponentDefinition Type |
|---|---|---|
| Promoter | http://identifiers.org/so/SO:0000167 | DNA |
| RBS | http://identifiers.org/so/SO:0000139 | DNA |
| CDS | http://identifiers.org/so/SO:0000316 | DNA |
| Terminator | http://identifiers.org/so/SO:0000141 | DNA |
| Gene | http://identifiers.org/so/SO:0000704 | DNA |
| Operator | http://identifiers.org/so/SO:0000057 | DNA |
| Engineered Gene | http://identifiers.org/so/SO:0000280 | DNA |
| mRNA | http://identifiers.org/so/SO:0000234 | RNA |
| Effector | http://identifiers.org/chebi/CHEBI:35224 | Small Molecule |
| Transcription Factor | http://identifiers.org/go/GO:0003700 | Protein |

***Table 4:*** *Ontology terms to specify the `roles` property of a `ComponentDefinition`, organized by the type of `ComponentDefinition` to which they are intended to apply (see Table 2).*

#### *The `sequences` property*

The `sequences` property is OPTIONAL and MAY include a set of `URI`s that refer to `Sequence` objects. These objects define the primary structure of the `ComponentDefinition`.

Many `ComponentDefinition` objects will refer to precisely one `Sequence` object. For certain use cases, however, it can be appropriate to refer to multiple `Sequence` objects. For example, a user might wish to provide two different representations of the structure of a DNA `ComponentDefinition`, one that represents its structure at the level of nucleotide bases and one that represents its structure at the level of atoms and bonds.

If a `ComponentDefinition` refers to more than one `Sequence` object, then these objects MUST be consistent with each other, such that well-defined mappings exist between their `elements` properties in accordance with their `encoding` properties. Furthermore, these objects MUST NOT have conflicting `encoding` properties. For example, the IUPAC encoding properties provided by Table 1 conflict with each other because they do not specify how to encode the same class of biochemical entity. The SMILES encoding, however, does not conflict with them because it specifies how to encode biochemical entities in general, which includes DNA, RNA, and proteins. If a ComponentDefinition refers to more than one Sequence with the same encoding, then the elements of these Sequence objects SHOULD have equal lengths. These requirements and best practices are intended to make it easier for software tools to locate any regions specified by the SequenceAnnotation objects of a ComponentDefinition on its associated Sequence objects, as well as validate whether its Sequence objects are consistent with those associated with any ComponentDefinition objects that it composes via its Component objects.

Finally, if a ComponentDefinition refers to one or more Sequence objects and its types property refers to a term from Table 2, then one of these Sequence objects MUST have the encoding that is cross-listed with this term in Table 1. Conversely, if a ComponentDefinition refers to a Sequence with an encoding from Table 1, then its types property MUST refer to the term from Table 2 that is cross-listed with this encoding in Table 1. For example, if the types property of a ComponentDefinition refers to the BioPAX term for DNA, then one of the Sequence objects to which it refers (if any) MUST have an IUPAC DNA encoding, and if a ComponentDefinition refers to a Sequence with an IUPAC DNA encoding, then its types property MUST refer to the BioPAX term for DNA. These requirements are meant to provide for some degree of consistency between the types property of a ComponentDefinition and the encoding properties of the Sequence objects to which the ComponentDefinition refers.

#### *The components property*

The components property is OPTIONAL and MAY specify a set of Component objects that are contained by the ComponentDefinition. The set of relations between Component and ComponentDefinition objects is strictly acyclic (see Section 7.7.1).

While the ComponentDefinition class is analogous to a blueprint or specification sheet for a biological part, the Component class represents the specific occurrence of a part within a design. Hence, this class allows a biological design to include multiple instances of a particular part (defined by reference to the same ComponentDefinition). For example, the ComponentDefinition of a polycistronic gene could contain two Component objects that refer to the same ComponentDefinition of a CDS.

The components properties of ComponentDefinition objects can be used to construct a hierarchy of Component and ComponentDefinition objects. If a ComponentDefinition in such a hierarchy refers to one or more Sequence objects, and there exist ComponentDefinition objects lower in the hierarchy that refer to Sequence objects with the same encoding, then the elements properties of these Sequence objects SHOULD be consistent with each other, such that well-defined mappings exist from the "lower level" elements to the "higher level" elements in accordance with their shared encoding properties. This mapping is also subject to any restrictions on the positions of the Component objects in the hierarchy that are imposed by the SequenceAnnotation or SequenceConstraint objects contained by the ComponentDefinition objects in the hierarchy.

A DNA ComponentDefinition, for example, could refer to a Sequence with an IUPAC DNA encoding and an elements String of "gattaca." In turn, this ComponentDefinition could contain a Component that refers to a "lower level" ComponentDefinition that also refers to a Sequence with an IUPAC DNA encoding. Consequently, a consistent elements String of this "lower level" Sequence could be "gatta," or perhaps "tgta" if the Component is positioned by a SequenceAnnotation that contains a Location with an orientation of "reverse complement" (see Section 7.7.5).

#### *The sequenceAnnotations property*

The sequenceAnnotations property is OPTIONAL and MAY contain a set of SequenceAnnotation objects. Each SequenceAnnotation specifies and describes a potentially discontiguous region on the Sequence objects referred to by the ComponentDefinition.

In addition, each SequenceAnnotation can position a Component of the ComponentDefinition at the region specified by its Location objects (see Section 7.7.5). The sequenceAnnotations property MUST NOT contain two or more SequenceAnnotation objects that refer to the same Component in this way.

Finally, as a best practice, if a ComponentDefinition refers to a Sequence with an IUPAC encoding from Table 1, then each of its SequenceAnnotation objects that contains a Range or Cut SHOULD specify a region on the elements of this Sequence. For example, the ComponentDefinition of a eukaryotic gene could refer to a Sequence with an IUPAC DNA encoding. In order to specify the discontiguous region occupied by its CDS, this gene ComponentDefinition would need a SequenceAnnotation that contains one or more Range objects, each one specifying start and end positions that correspond to indices of the elements of its DNA Sequence.

#### *The sequenceConstraints property*

The sequenceConstraints property is OPTIONAL and MAY contain a set of SequenceConstraint objects. These objects describe any restrictions on the relative, sequence-based positions and/or orientations of the Component objects contained by the ComponentDefinition. For example, the ComponentDefinition of a gene might specify that the position of its promoter Component precedes that of its CDS Component. This is particularly useful when a ComponentDefinition lacks a Sequence and therefore cannot specify the precise, sequence-based positions of its Component objects using SequenceAnnotation objects.

#### *Serialization*

The serialization of a ComponentDefinition MUST have the form below. The components, sequenceConstraints, sequenceAnnotations, and sequences properties of a ComponentDefinition contain or reference objects belonging to the appropriate SBOL classes as their values, while the types and roles properties contain URIs that identify ontology terms as their values.

As shown below, each of these objects and URIs are serialized as part of an implicit set of SBOL properties with singular rather then plural names. In particular, each object is serialized as an RDF/XML node nested within a property, while each URI (except the identity) is serialized as an rdf:resource on a property.

```
<sbol:ComponentDefinition rdf:about="...">
  ... properties inherited from identified ...
  zero or more  <sbol:sequence rdf:resource="..."/> element
  one or more   <sbol:type rdf:resource="..."/> elements
  zero or more  <sbol:role rdf:resource="..."/> elements
  zero or more  <sbol:component>
                  <sbol:Component rdf:about="...">...</sbol:Component>
                </sbol:component> elements
  zero or more <sbol:sequenceAnnotation>
                  <sbol:SequenceAnnotation rdf:about="...">...</sbol:SequenceAnnotation>
                </sbol:sequenceAnnotation> elements
  zero or more <sbol:sequenceConstraint>
                  <sbol:SequenceConstraint rdf:about="...">...</sbol:SequenceConstraint>
                </sbol:sequenceConstraint> elements
</sbol:ComponentDefinition>
```

The example below shows the serialization for the ComponentDefinition of a promoter. The BioPAX term DnaRegion and the ChEBI term CHEBI:4705 (double-stranded DNA) are used to indicate that the type of biological entity represented by this ComponentDefinition is DNA. Its role is specified using the SO terms SO:0000167 (promoter) and the more specific SO:0000613 (bacterial_RNApol_promoter).

```
<?xml version="1.0" ?>
<rdf:RDF xmlns:rdf="http://www.w3.org/1999/02/22-rdf-syntax-ns#" xmlns:dcterms="http://purl.org/dc/terms/" xmlns:prov="http://www.
      w3.org/ns/prov#" xmlns:sbol="http://sbols.org/v2#">
  <sbol:ComponentDefinition rdf:about="http://partsregistry.org/cd/BBa_J23119">
    <sbol:persistentIdentity rdf:resource="http://partsregistry.org/cd/BBa_J23119"/>
    <sbol:displayId>BBa_J23119</sbol:displayId>
    <prov:wasDerivedFrom rdf:resource="http://partsregistry.org/Part:BBa_J23119"/>
    <dcterms:title>J23119 promoter</dcterms:title>
    <dcterms:description>Constitutive promoter</dcterms:description>
    <sbol:type rdf:resource="http://www.biopax.org/release/biopax-level3.owl#DnaRegion"/>
```

```
    <sbol:type rdf:resource="http://identifiers.org/so/SO:0000987">
    <sbol:role rdf:resource="http://identifiers.org/so/SO:0000167"/>
    <sbol:role rdf:resource="http://identifiers.org/so/SO:0000613"/>
    <sbol:sequence rdf:resource="http://partsregistry.org/seq/BBa_J23119"/>
  </sbol:ComponentDefinition>
</rdf:RDF>
```

#### 7.7.1 ComponentInstance

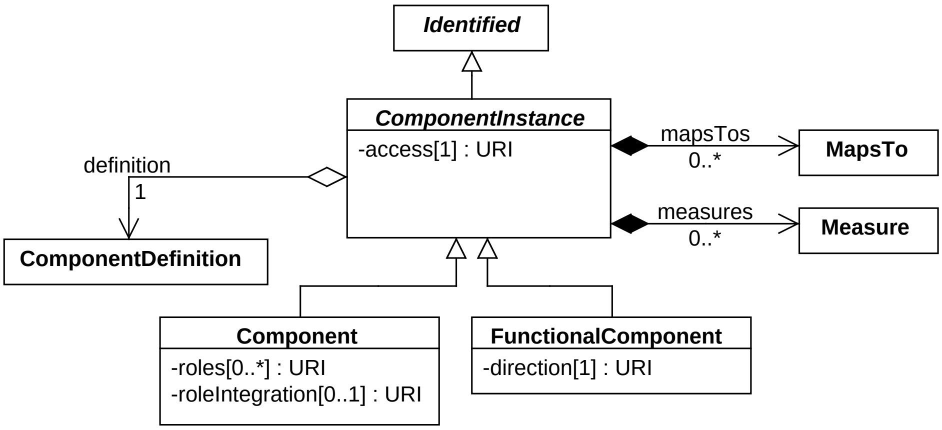

***Figure 8:** Diagram of the `ComponentInstance` class and its associated properties.*

The `ComponentInstance` abstract class is inherited by SBOL classes that represent the usage or occurrence of a `ComponentDefinition` within a larger design (that is, another `ComponentDefinition` or `ModuleDefinition`). Currently, there are two subclasses of `ComponentInstance`:

- The `Component` class is used to specify the structural usage of a `ComponentDefinition` inside another `ComponentDefinition` via the `components` property.
- The `FunctionalComponent` class is used to specify the functional usage of a `ComponentDefinition` inside a `ModuleDefinition` via the `functionalComponents` property. This class is described in Section 7.9.2.

##### *The `definition` property*

The `definition` property is a REQUIRED `URI` that refers to the `ComponentDefinition` of the `ComponentInstance`. As described in the previous section, this `ComponentDefinition` effectively provides information about the `types` and `roles` of the `ComponentInstance`.

The `definition` property MUST NOT refer to the same `ComponentDefinition` as the one that contains the `ComponentInstance`. Furthermore, `ComponentInstance` objects MUST NOT form a cyclical chain of references via their `definition` properties and the `ComponentDefinition` objects that contain them. For example, consider the `ComponentInstance` objects $A$ and $B$ and the `ComponentDefinition` objects $X$ and $Y$. The reference chain "$X$ contains $A$, $A$ is defined by $Y$, $Y$ contains $B$, and $B$ is defined by $X$" is cyclical.

##### *The `mapsTos` property*

The `mapsTos` property is OPTIONAL and MAY contain a set of `MapsTo` objects that refer to and link together `ComponentInstance` objects (both `Component` objects and `FunctionalComponent` objects) within a larger design.

Section 7.7.3 contains a more detailed description of the `MapsTo` class.

##### *The access property*

The access property is a REQUIRED URI that indicates whether the ComponentInstance can be referred to remotely by a MapsTo on another ComponentInstance or Module contained by a different parent ComponentDefinition or ModuleDefinition (one that does not contain this ComponentInstance).

Table 5 provides a list of REQUIRED access URIs. The value of the access property MUST be one of these URIs.

| Access URI | Description |
|---|---|
| http://sbols.org/v2#public | The ComponentInstance MAY be referred to by remote MapsTo objects. |
| http://sbols.org/v2#private | The ComponentInstance MUST NOT be referred to by remote MapsTo objects. |

*Table 5: REQUIRED URIs for the access property.*

In some cases, a designer might want to set the access property of a ComponentInstance such that others cannot map to the ComponentInstance when they reuse its parent ComponentDefinition. For example, a designer who is concerned about retroactivity might set the access of the ComponentInstance to “private” in order to prevent its mapping to another ComponentInstance that participates in a new Interaction as part of a composite design.

2.3.0

##### *The measures property*

The measures property is OPTIONAL and MAY contain a set of Measure objects. Section 13.2.1 contains a more detailed description of the Measure class.

##### *Serialization*

No serialization is defined for the ComponentInstance class, since this class is only used indirectly through the Component and FunctionalComponent subclasses.

#### 7.7.2 Component

The Component class is used to compose ComponentDefinition objects into a structural hierarchy. For example, the ComponentDefinition of a gene could contain four Component objects: a promoter, RBS, CDS, and terminator. In turn, the ComponentDefinition of the promoter Component could contain Component objects defined as various operator sites.

2.1.0

##### *The roles property*

[New in 2.1.0; see SEP 004: https://github.com/SynBioDex/SEPs/blob/master/sep_004.md]

The expected purpose and function of a genetic part are described by the roles property of ComponentDefinition. However, the same building block might be used for a different purpose in an actual design. In other words, purpose and function are sometimes determined by context.

The roles property comprises an OPTIONAL set of zero or more role URIs describing the purpose or potential function of this Component’s included sub-ComponentDefinition in the *context* of its parent ComponentDefinition. If provided, these role URIs MUST identify terms from appropriate ontologies. Roles are not restricted to describing biological function; they may annotate a Component’s function in any domain for which an ontology exists.

It is RECOMMENDED that these role URIs identify terms that are compatible with the type properties of both this Component’s parent ComponentDefinition and its included sub-ComponentDefinition. For example, a role of a Component which belongs to a ComponentDefinition of type DNA and includes a sub-ComponentDefinition of type DNA might refer to terms from the Sequence Ontology. A table of recommended ontology terms for roles is given in Table 4.

2.1.0

##### *The roleIntegration property*

[New in 2.1.0; see SEP 004: https://github.com/SynBioDex/SEPs/blob/master/sep_004.md]

A roleIntegration specifies the relationship between a Component instance's own set of roles and the set of roles on the included sub-ComponentDefinition.

The roleIntegration property has a data type of URI. A Component instance with zero roles MAY OPTIONALLY specify a roleIntegration. A Component instance with one or more roles MUST specify a roleIntegration from Table 6. If zero Component roles are given and no Component roleIntegration is given, then http://sbols.org/v2#mergeRoles is assumed. It is RECOMMENDED to specify a set of Component roles only if the integrated result set of roles would differ from the set of roles belonging to this Component's included sub-ComponentDefinition.

2.1.0

| roleIntegration URI | Description |
|---|---|
| http://sbols.org/v2#overrideRoles | In the context of this Component, ignore any roles given for the included sub-ComponentDefinition. Instead use only the set of zero or more roles given for this Component. |
| http://sbols.org/v2#mergeRoles | Use the union of the two sets: both the set of zero or more roles given for this Component as well as the set of zero or more roles given for the included sub-ComponentDefinition. |

***Table 6:*** *Each roleIntegration mode is associated with a rule governing how a Component's roles are to be combined with the included sub-ComponentDefinition's roles.*

2.3.0

#### *The sourceLocations property*

[New in 2.3.0; see SEP 013: https://github.com/SynBioDex/SEPs/blob/master/sep_013.md]

The sourceLocations property is an OPTIONAL set of zero or more Location objects that indicate which elements of a ComponentDefinition's Sequence are to be included in the Component's parent. The sourceLocations property allows for only a portion of a ComponentDefinition's Sequence to be included, rather than its entirety.

If the sourceLocations property is not set, then the whole Sequence is assumed to be included. Alternatively, if the sourceLocations property is set, then the relationship between the original ComponentDefinition's Sequence and the included Sequence is defined identically to the locations property on the SequenceAnnotation object.

#### *Serialization*

2.1.0 The serialization of a Component MUST have the following form:

```
<sbol:Component rdf:about="...">
  ... properties inherited from identified ...
  one          <sbol:access rdf:resource="..."/> element
  one          <sbol:definition rdf:resource="..."/> element
  zero or more <sbol:mapsTo rdf:resource="..."/> elements
  zero or more <sbol:role rdf:resource="..."/> elements
  zero or one  <sbol:roleIntegration rdf:resource="..."/> element
  zero or one  <sbol:sourceLocation rdf:resource="..."/> element
</sbol:Component>
```

The example below shows the serialization of a Component that represents an instance of a promoter:

```
<sbol:Component rdf:about="http://partsregistry.org/cd/BBa_F2620/pLuxR">
  <sbol:persistentIdentity rdf:resource="http://partsregistry.org/cd/BBa_F2620/pLuxR"/>
  <sbol:displayId>pLuxR</sbol:displayId>
  <sbol:access rdf:resource="http://sbols.org/v2#public"/>
  <sbol:definition rdf:resource="http://partsregistry.org/cd/BBa_R0062"/>
</sbol:Component>
```

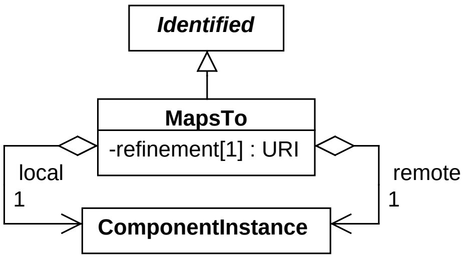

***Figure 9:** Diagram of the MapsTo class and its associated properties.*

#### 7.7.3 MapsTo

When ComponentDefinition and ModuleDefinition objects are composed into structural and functional hierarchies using ComponentInstance and Module objects, it is often the case that some ComponentInstance objects are intended to represent the same entity in the overall design. The purpose of the MapsTo class is to make these identity relationships clear and explicit. For example, consider a ModuleDefinition for a genetic inverter that includes a FunctionalComponent for an abstract repressor protein. When this ModuleDefinition is instantiated within a "higher level" ModuleDefinition that includes a FunctionalComponent for a LacI protein, the MapsTo object can be used to indicate that the repressor protein in the first ModuleDefinition is LacI in the context of the composite design.

In particular, a MapsTo object provides two pieces of information:

- An identity relationship between two ComponentInstance objects, the first contained by the "lower level" definition of the ComponentInstance or Module that owns the MapsTo, and the second contained by the "higher level" definition that contains the ComponentInstance or Module that owns the MapsTo. The remote property of a MapsTo refers to the first "lower level" ComponentInstance, while the local property refers to the second "higher level" ComponentInstance.
- Instructions on how to interpret local and remote ComponentInstance objects that refer to different ComponentDefinition objects (that is, non-identical objects). These are specified using the refinement property of the MapsTo class.

To illustrate this concept, two examples are provided in Figure 10, in which the ComponentDefinition of a transcriptional unit is specified by composing two "lower level" ComponentDefinition objects. In both examples, the two "lower level" ComponentDefinition objects each contain a RBS Component that is intended to represent the same design entity in the "higher level" ComponentDefinition of the transcriptional unit.

In order to explicitly represent the identity relationships in this example, a new RBS Component needs to be created inside the "higher level" ComponentDefinition. This "higher level" Component then needs to be linked to the equivalent "lower level" Component objects by means of the MapsTo class, using one MapsTo object per link. For example, in order to link the "higher level" RBS Component to the "lower level" RBS Component of the promoter-RBS ComponentDefinition, a MapsTo has to be created on the "higher level" promoter-RBS Component. The local property of this MapsTo then has to refer to the "higher level" RBS Component, while its remote property has to refer to the "lower level" RBS Component. In this way, many "lower level" Component objects can be linked together at the "higher level" using as an equal number of MapsTo objects, each one referring to a different remote Component, but all referring to the same local Component.

The same types of identity relationships can also be declared between FunctionalComponent objects contained by ModuleDefinition objects, or between Component objects and FunctionalComponent objects contained by ComponentDefinition objects and ModuleDefinition objects, respectively. See Section 9 and Section B for additional examples using the MapsTo class.

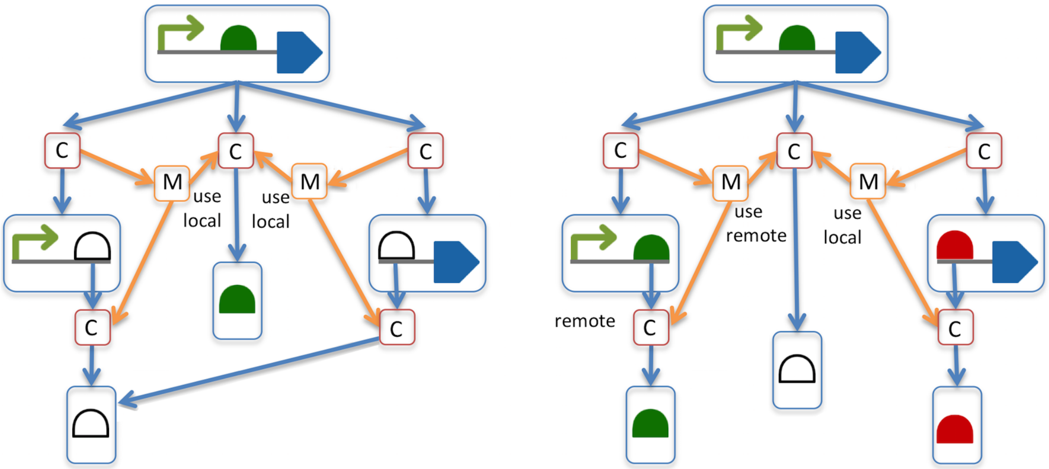

*Figure 10: Linking `Component` objects using `MapsTo` entities. Boxes with diagrams represent `ComponentDefinition` objects, boxes with the C label represent `Component` objects, and boxes with the M label represent `MapsTo` objects. In both diagrams, a promoter-RBS `ComponentDefinition` and a RBS-CDS `ComponentDefinition` are being composed to form the `ComponentDefinition` of a complete transcriptional unit. In the left-hand diagram, the two `Component` objects inside the promoter-RBS `ComponentDefinition` and RBS-CDS `ComponentDefinition` objects both refer to an abstract RBS `ComponentDefinition` that lacks a sequence (white semicircle). Through the use of `MapsTo` objects with `refinement` set to useLocal, these "lower level" `ComponentDefinition` objects are effectively overridden by that of the green RBS in the `ComponentDefinition` of the complete transcriptional unit. In the right-hand diagram, however, the two "lower level" RBS `ComponentDefinition` objects do not lack sequences and it is the "higher level" RBS `ComponentDefinition` that is abstract. In this case, one of the `MapsTo` objects has a useRemote `refinement`, resulting in the green RBS `ComponentDefinition` overriding that of the abstract RBS in the "higher level" `ComponentDefinition`.*

#### *The `local` property*

This REQUIRED property has a data type of `URI` and is used to refer to the `ComponentInstance` contained by the "higher level" `ComponentDefinition` or `ModuleDefinition`. This `local` `ComponentInstance` MUST be contained by the `ComponentDefinition` or `ModuleDefinition` that contains the `ComponentInstance` or `Module` that owns the `MapsTo`.

#### *The `remote` property*

This REQUIRED property has a data type of `URI` and is used to refer to the `ComponentInstance` contained by the "lower level" `ComponentDefinition` or `ModuleDefinition`. This `remote` `ComponentInstance` MUST be contained by the `ComponentDefinition` or `ModuleDefinition` that is the `definition` of the `ComponentInstance` or `Module` that owns the `MapsTo`. Lastly, the `access` property of the `remote` `ComponentInstance` MUST be set to "public."

#### *The `refinement` property*

The `refinement` property is REQUIRED and has a data type of `URI`. Each `MapsTo` object MUST specify the relationship between its `local` and `remote` `ComponentInstance` objects using one of the REQUIRED `refinement` `URI`s provided in Table 7. Note that if multiple `MapsTo`s belonging to the `Component`s of a `ComponentDefinition` have
2.0.1 `local` properties that refer to the same `Component`, then there MUST NOT be more than one such `MapsTo` that has a `refinement` property that contains the URI `http://sbols.org/v2#useRemote`. Similarly, if multiple `MapsTo`s belonging the `Module`s and `FunctionalComponent`s of a `ModuleDefinition` have `local` properties that refer to the same `FunctionalComponent`, then there MUST NOT be more than one such `MapsTo` that has a `refinement` property that contains the URI `http://sbols.org/v2#useRemote`.

#### *Serialization*

The serialization of `MapsTo` MUST have the following form.

| Refinement URI | Description |
|---|---|
| http://sbols.org/v2#useRemote | All references to the local ComponentInstance MUST dereference to the remote ComponentInstance instead. |
| http://sbols.org/v2#useLocal | In the context of the ComponentDefinition or ModuleDefinition that contains the owner of the MapsTo, all references to the remote ComponentInstance MUST dereference to the local ComponentInstance instead. |
| http://sbols.org/v2#verifyIdentical | The definition properties of the local and remote ComponentInstance objects MUST refer to the same ComponentDefinition. |
| http://sbols.org/v2#merge | In the context of the ComponentDefinition or ModuleDefinition that contains the owner of the MapsTo, all references to the local ComponentInstance or the remote ComponentInstance MUST dereference to both objects. |

***Table 7:*** *REQUIRED URIs for the refinement property.*

```
<sbol:MapsTo rdf:about="...">
  ... properties inherited from identified ...
  one <sbol:refinement rdf:resource="..."/> element
  one <sbol:remote rdf:resource="..."/> element
  one <sbol:local rdf:resource="..."/> element
</sbol:MapsTo>
```

In the example below, a FunctionalComponent in a "higher level" ModuleDefinition of a genetic toggle switch is linked to a FunctionalComponent in a "lower level" LacI inverter ModuleDefinition. The full example can be found in Section 9.

```
<sbol:MapsTo rdf:about="http://sbolstandard.org/example/toggle_switch/laci_inverter/LacI_mapping">
  <sbol:persistentIdentity rdf:resource="http://sbolstandard.org/example/toggle_switch/laci_inverter/LacI_mapping"/>
  <sbol:displayId>LacI_mapping</sbol:displayId>
  <sbol:refinement rdf:resource="http://sbols.org/v2#useRemote"/>
  <sbol:remote rdf:resource="http://sbolstandard.org/example/laci_inverter/TF"/>
  <sbol:local rdf:resource="http://sbolstandard.org/example/toggle_switch/LacI"/>
</sbol:MapsTo>
```

#### 7.7.4 SequenceAnnotation

The SequenceAnnotation class describes one or more regions of interest on the Sequence objects referred to by its parent ComponentDefinition. In addition, SequenceAnnotation objects can describe the substructure of their parent ComponentDefinition through association with the Component objects contained by this ComponentDefinition.

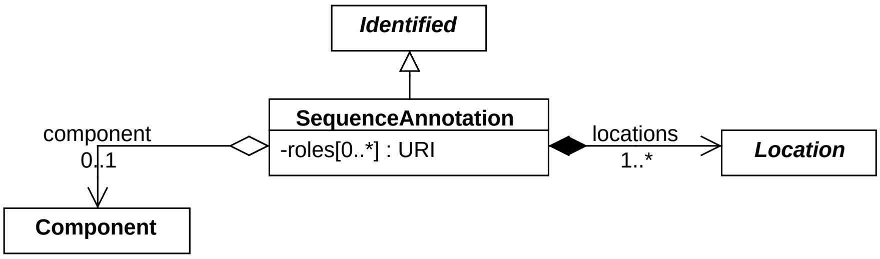

***Figure 11:*** *Diagram of the SequenceAnnotation class and its associated properties.*

#### *The `locations` property*

The locations property is a REQUIRED set of one or more Location objects that indicate which elements of a Sequence are described by the SequenceAnnotation.

Allowing multiple Location objects on a single SequenceAnnotation is intended to enable representation of discontinuous regions (for example, a Component encoded across a set of exons with interspersed introns). As such, the Location objects of a single SequenceAnnotation SHOULD NOT specify overlapping regions, since it is not clear what this would mean. There is no such concern with different SequenceAnnotation objects, however, which can freely overlap in Location (for example, specifying overlapping linkers for sequence assembly).

#### *The `component` property*

The component property is OPTIONAL and has a data type of URI. This URI MUST refer to a Component that is contained by the same parent ComponentDefinition that contains the SequenceAnnotation. In this way, the properties of the SequenceAnnotation, such as its description and locations, are associated with part of the substructure of its parent ComponentDefinition.

2.1.0

#### *The `roles` property*

[New in 2.1.0; see SEP 004: https://github.com/SynBioDex/SEPs/blob/master/sep_004.md]
[New in 2.1.0; see SEP 010: https://github.com/SynBioDex/SEPs/blob/master/sep_010.md]

Alternatively to describing substructure, a SequenceAnnotation can be utilized to identify a feature, such as a GenBank feature, of a specified Sequence. In this use case, the SequenceAnnotation MUST NOT have a component property, but instead it would have a roles property.

The roles property comprises an OPTIONAL set of zero or more URIs describing the specified sequence feature being annotated. If provided, these role URIs MUST identify terms from appropriate ontologies. Roles are not restricted to describing biological function; they may annotate Sequences' function in any domain for which an ontology exists.

It is RECOMMENDED that these role URIs identify terms that are compatible with the type properties of this SequenceAnnotation's parent ComponentDefinition. For example, a role of a SequenceAnnotation which belongs to a ComponentDefinition of type DNA might refer to terms from the Sequence Ontology. A table of recommended ontology terms for roles is given in Table 4.

#### *Serialization*

2.1.0 The serialization of a SequenceAnnotation MUST have the form below. In this template, A_LOCATION_SUBCLASS represents one of the Location subclasses.

```
<sbol:SequenceAnnotation rdf:about="...">
  ... properties inherited from identified ...
  zero or one <sbol:component rdf:resource="..."/> element
  one or more <sbol:location>
               <sbol:A_LOCATION_SUBCLASS rdf:about="...">...</sbol:A_LOCATION_SUBCLASS>
            </sbol:location> elements
  zero or more <sbol:role rdf:resource="..."/> elements
</sbol:SequenceAnnotation>
```

The example below shows the serialization of a SequenceAnnotation object. It specifies the region occupied by a Component named BBa_F2620.

```
<sbol:SequenceAnnotation rdf:about="http://partsregistry.org/cd/BBa_F2620/anno2">
  <sbol:persistentIdentity rdf:resource="http://partsregistry.org/cd/BBa_F2620/anno2"/>
  <sbol:displayId>anno2</sbol:displayId>
  <sbol:location>
    <sbol:Range rdf:about="http://partsregistry.org/cd/BBa_F2620/anno2/range">
      <sbol:persistentIdentity rdf:resource="http://partsregistry.org/cd/BBa_F2620/anno2/range"/>
      <sbol:displayId>range</sbol:displayId>
```

```
      <sbol:start>56</sbol:start>
      <sbol:end>68</sbol:end>
      <sbol:orientation rdf:resource="http://sbols.org/v2#inline"/>
    </sbol:Range>
  </sbol:location>
  <sbol:component rdf:resource="http://partsregistry.org/cd/BBa_F2620/rbs"/>
</sbol:SequenceAnnotation>
```

#### 7.7.5 Location

The `Location` class is extended by the `Range`, `Cut`, and `GenericLocation` classes.

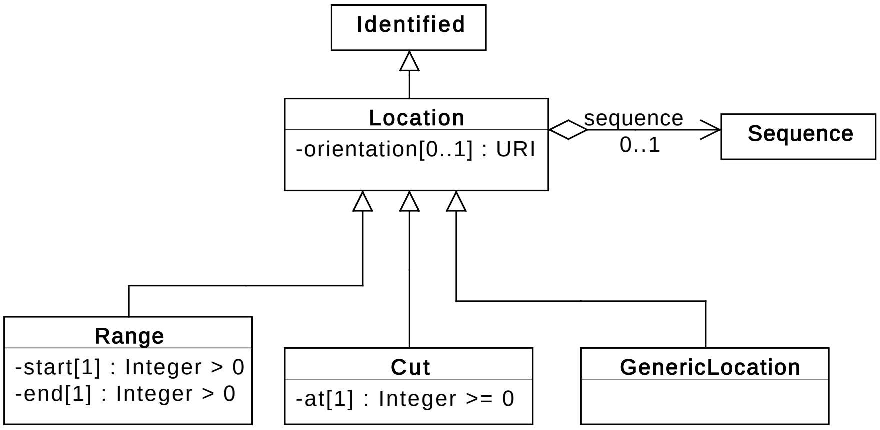

***Figure 12:*** *Diagram of the `Location` class and its associated properties.*

##### *The `orientation` property*

The `orientation` property is OPTIONAL and has a data type of `URI`. All subclasses of `Location` share this property, which can be used to indicate how the region specified by the `SequenceAnnotation` and any associated double-stranded `Component` is oriented on the `elements` of a `Sequence` from their parent `ComponentDefinition`. Table 8 provides a list of REQUIRED `orientation` `URI`s. If a `Location` object has an `orientation`, then it MUST come from Table 8.

2.3.0

##### *The `sequence` property*

The `sequence` property is OPTIONAL and MAY contain the `URI` of a `Sequence` object. All subclasses of `Location` share this property, which indicates which `Sequence` object referenced by the containing `ComponentDefinition` is referenced by the `Location`.

| Orientation URI | Description |
|---|---|
| `http://sbols.org/v2#inline` | The region specified by this `Location` is on the `elements` of a `Sequence`. |
| `http://sbols.org/v2#reverseComplement` | The region specified by this `Location` is on the reverse-complement translation of the `elements` of a `Sequence`. The exact nature of this translation depends on the `encoding` of the `Sequence`. |

***Table 8:*** *REQUIRED `URI`s for the `orientation` property*

##### Range

A Range object specifies a region via discrete, inclusive start and end positions that correspond to indices for characters in the elements String of a Sequence.

Note that the index of the first location is 1, as is typical practice in biology, rather than 0, as is typical practice in computer science.

###### The start property

The start property specifies the inclusive starting position of the Range. This property is REQUIRED and MUST contain an Integer value greater than zero.

###### The end property

The end property specifies the inclusive ending position of the Range. This property is REQUIRED and MUST contain an Integer value greater than zero. In addition, this Integer value MUST be greater than or equal to that of the start property.

###### Serialization

The serialization of a Range MUST have the following form:

```
<sbol:Range rdf:about="...">
  ... properties inherited from identified ...
  one         <sbol:start>...</sbol:start> element
  one         <sbol:end>...</sbol:end> element
  zero or one <sbol:orientation rdf:resource="..."/> element
  zero or one <sbol:sequence rdf:resource="..."/> element
</sbol:Range>
```

The example below shows the serialization of a Range object. It specifies the region between the inclusive positions 56 and 68, with an orientation of "inline."

```
<sbol:Range rdf:about="http://partsregistry.org/cd/BBa_F2620/anno2/range">
  <sbol:persistentIdentity rdf:resource="http://partsregistry.org/cd/BBa_F2620/anno2/range"/>
  <sbol:displayId>range</sbol:displayId>
  <sbol:start>56</sbol:start>
  <sbol:end>68</sbol:end>
  <sbol:orientation rdf:resource="http://sbols.org/v2#inline"/>
  <sbol:sequence rdf:resource="http://partsregistry.org/cd/BBa_F2620_sequence"/>
</sbol:Range>
```

##### Cut

The Cut class has been introduced to enable the specification of a region between two discrete positions. This specification is accomplished using the at property, which specifies a discrete position that that corresponds to the index of a character in the elements String of a Sequence (except in the case when at is equal to zero—see below).

###### The at property

The at property is REQUIRED and MUST contain an Integer value greater than or equal to zero. The region specified by the Cut is between the position specified by this property and the position that immediately follows it. When the at property is equal to zero, the specified region is immediately before the first discrete position or character in the elements String of a Sequence.

###### Serialization

The serialization of a Cut MUST have the following form:

```
<sbol:Cut rdf:about="...">
  ... properties inherited from identified ...
  one         <sbol:at>...</sbol:at> element
  zero or one <sbol:orientation rdf:resource="..."/> element
  zero or one <sbol:sequence rdf:resource="..."/> element
</sbol:Cut>
```

The example below shows the serialization of a Cut object. It specifies a region in between positions 10 and 11, with an orientation of "inline."

```
<sbol:Cut rdf:about="http://partsregistry.org/cd/BBa_J23119/cutat10/cut">
  <sbol:persistentIdentity rdf:resource="http://partsregistry.org/cd/BBa_J23119/cutat10/cut"/>
  <sbol:displayId>cut</sbol:displayId>
  <sbol:at>10</sbol:at>
  <sbol:orientation rdf:resource="http://sbols.org/v2#inline"/>
  <sbol:sequence rdf:resource="http://partsregistry.org/cd/BBa_J23119_sequence"/>
</sbol:Cut>
```

##### *GenericLocation*

While the Range and Cut classes are best suited to specifying regions on Sequence objects with IUPAC encodings, the GenericLocation class is included as a starting point for specifying regions on Sequence objects with different encoding properties and potentially nonlinear structure. This class can also be used to set the orientation of a SequenceAnnotation and any associated Component when their parent ComponentDefinition is a partial design that lacks a Sequence.

##### *Serialization*

The serialization of a GenericLocation MUST have the following form:

```
<sbol:GenericLocation rdf:about="...">
  ... properties inherited from identified ...
  zero or one <sbol:orientation rdf:resource="..."/> element
  zero or one <sbol:sequence rdf:resource="..."/> element
</sbol:GenericLocation>
```

The example below shows the serialization of a GenericLocation object with an orientation of "reverse complement":

```
<sbol:GenericLocation rdf:about="http://www.partsregistry.org/Part:BBa_F2620/anno5/location">
  <sbol:orientation rdf:resource="http://sbols.org/v2#reverseComplement"/>
  <sbol:sequence rdf:resource="http://www.partsregistry.org/Part:BBa_F2620#sequence"/>
</sbol:GenericLocation>
```

#### *7.7.6 SequenceConstraint*

The SequenceConstraint class can be used to assert restrictions on the relative, sequence-based positions of pairs of Component objects contained by the same parent ComponentDefinition. The primary purpose of this class is to enable the specification of partially designed ComponentDefinition objects, for which the precise positions or orientations of their contained Component objects are not yet fully determined. Each SequenceConstraint includes the restriction, subject, and object properties.

##### *The `subject` property*

The subject property is REQUIRED and MUST contain a URI that refers to a Component contained by the same parent ComponentDefinition that contains the SequenceConstraint.

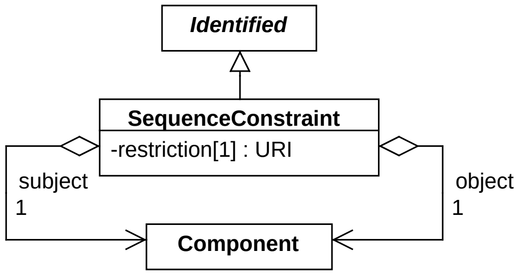

***Figure 13:*** *Diagram of the `SequenceConstraint` class and its associated properties.*

#### The `object` property

The `object` property is REQUIRED and MUST contain a `URI` that refers to a `Component` contained by the same parent `ComponentDefinition` that contains the `SequenceConstraint`. This `Component` MUST NOT be the same `Component` that the `SequenceConstraint` refers to via its `subject` property.

#### The `restriction` property

The `restriction` property is REQUIRED and has a data type of `URI`. This property MUST indicate the type of structural restriction on the positions, orientations, or structural identities of the `subject` and `object` `Component` objects in relation to each other. The `URI` value of this property SHOULD come from the RECOMMENDED `URI`s in Table 9.

2.2.0

| Restriction URI | Description |
| --- | --- |
| `http://sbols.org/v2#precedes` | The position of the `subject` `Component` MUST precede that of the `object` `Component`. If each one is associated with a `SequenceAnnotation`, then the `SequenceAnnotation` associated with the `subject` `Component` MUST specify a region that starts before the region specified by the `SequenceAnnotation` associated with the `object` `Component`. |
| `http://sbols.org/v2#sameOrientationAs` | The `subject` and `object` `Component` objects MUST have the same orientation. If each one is associated with a `SequenceAnnotation`, then the `orientation` `URI`s of the `Location` objects of the first `SequenceAnnotation` MUST be among those of the second `SequenceAnnotation`, and vice versa. |
| `http://sbols.org/v2#oppositeOrientationAs` | The `subject` and `object` `Component` objects MUST have opposite orientations. If each one is associated with a `SequenceAnnotation`, then the `orientation` `URI`s of the `Location` objects of one `SequenceAnnotation` MUST NOT be among those of the other `SequenceAnnotation`. |
| `http://sbols.org/v2#differentFrom` | The `definition` property of the `subject` `Component` MUST NOT refer to the same `ComponentDefinition` as that of the `object` `Component`. |

***Table 9:*** *RECOMMENDED `URI`s for the `restriction` property.*

#### Serialization

The serialization of a `SequenceConstraint` MUST have the following form:

```
<sbol:SequenceConstraint rdf:about="...">
  ... properties inherited from identified ...
  one <sbol:restriction rdf:resource="..."/> element
  one <sbol:subject rdf:resource="..."/> element
  one <sbol:object rdf:resource="..."/> element
</sbol:SequenceConstraint>
```

The example below shows the serialization of a `SequenceConstraint` belonging to the `ComponentDefinition` of a LacI-repressible promoter. This `SequenceConstraint` has a "precedes" `restriction` that indicates that the `subject` `Component`, which represents the core of the promoter, is positioned before the `object` `Component`, which represents the LacI operator of the promoter.

```
<sbol:SequenceConstraint rdf:about="http://partsregistry.org/cd/BBa_K174004/r1">
  <sbol:persistentIdentity rdf:resource="http://partsregistry.org/cd/BBa_K174004/r1"/>
  <sbol:displayId>r1</sbol:displayId>
  <sbol:restriction rdf:resource="http://sbols.org/v2#precedes"/>
  <sbol:subject rdf:resource="http://partsregistry.org/cd/pspac"/>
  <sbol:object rdf:resource="http://partsregistry.org/cd/LacI_operator"/>
</sbol:SequenceConstraint>
```

### 7.8 Model

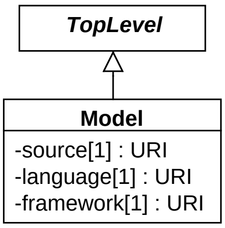

***Figure 14:*** *Diagram of the `Model` class and its associated properties.*

The purpose of the `Model` class is to serve as a placeholder for an external computational model and provide additional meta-data to enable better reasoning about the contents of this model. In this way, there is minimal duplication of standardization efforts and users of SBOL can formalize the function of a `ModuleDefinition` in the language of their choice.

The meta-data provided by the `Model` class include the following properties: the `source` or location of the actual content of the model, the `language` in which the model is implemented, and the model's `framework`.

#### *The `source` property*

The `source` property is REQUIRED and MUST contain a `URI` reference to the source file for a model.

#### *The `language` property*

The `language` property is REQUIRED and MUST contain a `URI` that specifies the language in which the model is implemented. It is RECOMMENDED that this `URI` refer to a term from the EMBRACE Data and Methods (EDAM) ontology. Table 10 provides a list of terms from this ontology and their `URI`s. If the `language` property of a `Model` is well-described by one these terms, then it MUST contain the `URI` for this term as its value.

| Model Language | URI for EDAM Term |
|---|---|
| SBML | http://identifiers.org/edam/format_2585 |
| CellML | http://identifiers.org/edam/format_3240 |
| BioPAX | http://identifiers.org/edam/format_3156 |

***Table 10:*** *Terms from the EDAM ontology to specify the* `language` *property of a* `Model`*.*

#### The `framework` property

The `framework` property is REQUIRED and MUST contain a `URI` that specifies the framework in which the model is implemented. It is RECOMMENDED this `URI` refer to a term from the modeling framework branch of the SBO when possible. A few suggested modeling frameworks and their corresponding `URI`s are shown in Table 11. If the `framework` property of a `Model` is well-described by one these terms, then it MUST contain the `URI` for this term as its value.

| Framework | URI for SBO Term |
|---|---|
| Continuous | http://identifiers.org/biomodels.sbo/SBO:0000062 |
| Discrete | http://identifiers.org/biomodels.sbo/SBO:0000063 |

***Table 11:*** *SBO terms to specify the* `framework` *property of a* `Model`*.*

#### Serialization

The serialization of a `Model` MUST have the following form:

```
<sbol:Model rdf:about="http://www.sbolstandard.org/examples/toggleswitch">
  ... properties inherited from identified ...
  one <sbol:source rdf:resource="..."/> element
  one <sbol:language rdf:resource="..."/> element
  one <sbol:framework rdf:resource="..."/> element
</sbol:Model>
```

The example below shows the serialization of a `Model` object that refers to a quantitative model of a genetic toggle switch. The model is implemented in the SBML `language` and adheres to a continuous modeling `framework`. Lastly, the model can be retrieved from a model repository via its `source` `URI`, which is a URL.

```
<?xml version="1.0" ?>
<rdf:RDF xmlns:rdf="http://www.w3.org/1999/02/22-rdf-syntax-ns#" xmlns:dcterms="http://purl.org/dc/terms/" xmlns:prov="http://www.
     w3.org/ns/prov#" xmlns:sbol="http://sbols.org/v2#">
  <sbol:Model rdf:about="http://www.sbolstandard.org/examples/pIKE_Toggle_1">
    <sbol:persistentIdentity rdf:resource="http://www.sbolstandard.org/examples/pIKE_Toggle_1"/>
    <sbol:displayId>pIKE_Toggle_1</sbol:displayId>
    <dcterms:title>pIKE_Toggle_1 toggle switch</dcterms:title>
    <sbol:source rdf:resource="http://virtualparts.org/part/pIKE_Toggle_1"/>
    <sbol:language rdf:resource="http://identifiers.org/edam/format_2585"/>
    <sbol:framework rdf:resource="http://identifiers.org/biomodels.sbo/SBO:0000062"/>
  </sbol:Model>
</rdf:RDF>
```

### 7.9 ModuleDefinition

The `ModuleDefinition` class represents a grouping of structural and functional entities in a biological design. The primary usage of this class is to assert the molecular interactions and abstract function of its child entities.

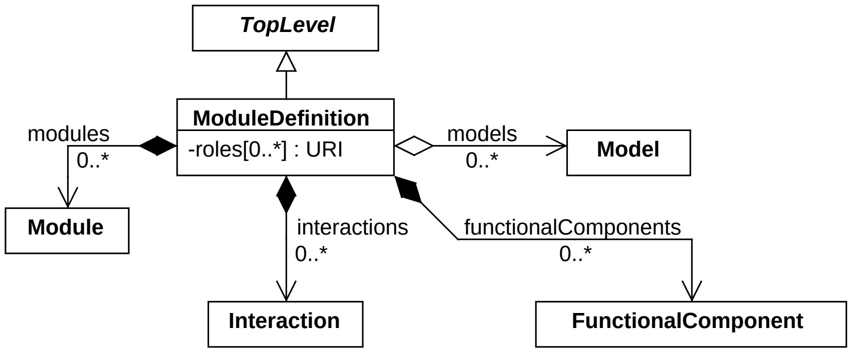

***Figure 15:*** *Diagram of the `ModuleDefinition` class and its associated properties.*

As shown in Figure 15, these child entities are aggregated via the `functionalComponents` `modules` properties, while representation of their abstract function is accomplished via the `roles` property. More detailed descriptions of the function of a `ModuleDefinition` are provided by its `interactions` and `models` properties. Lastly, since `ModuleDefinition` objects can be more abstract and represent entities of engineering design rather than biology, they can have designated "inputs" and "outputs" expressed by the `direction` properties on its `FunctionalComponent` objects.

#### The `roles` property

The `roles` property is an OPTIONAL set of `URI`s that clarifies the intended function of a `ModuleDefinition`.

These `URI`s might identify descriptive biological roles, such as "metabolic pathway" and "signaling cascade," but they can also identify identify "logical" roles, such as "inverter" or "AND gate", or other abstract roles for describing the function of design. Interpretation of the meaning of such roles currently depends on the software tools that read and write them.

#### The `modules` property

The `modules` property is OPTIONAL and MAY specify a set of `Module` objects contained by the `ModuleDefinition`. Note that the set of relations between `Module` and `ModuleDefinition` objects is strictly acyclic.

While the `ModuleDefinition` class is analogous to a specification sheet for a system of interacting biological elements, the `Module` class represents the occurrence of a particular subsystem within the system. Hence, this class allows a system design to include multiple instances of a subsystem, all defined by reference to the same `ModuleDefinition`. For example, consider the `ModuleDefinition` for a network of two-input repressor devices in which the particular repressors have not been chosen yet. This `ModuleDefinition` could contain multiple `Module` objects that refer to the same `ModuleDefinition` of an abstract two-input repressor device.

#### The `functionalComponents` property

The `functionalComponents` property is OPTIONAL and MAY specify a set of `FunctionalComponent` objects contained by the `ModuleDefinition`.

Just as a `Module` represents an instance of a subsystem in the overall system represented by a `ModuleDefinition`, a `FunctionalComponent` represents an instance of a structural entity (represented by a `ComponentDefinition`) in the system. This concept allows a `ModuleDefinition` to assert different interactions for separate copies of the same structural entity if needed. For example, a `ModuleDefinition` might contain multiple `FunctionalComponent` objects that refer to the same promoter `ComponentDefinition`, but assert different interactions for these promoter copies based on their separate positions in another `ComponentDefinition` that represents the structure of the entire system.

#### The `interactions` property

The `interactions` property is OPTIONAL and MAY specify a set of `Interaction` objects within the `ModuleDefinition`.

The `Interaction` class provides an abstract, machine-readable representation of entity behavior within a `ModuleDefinition` (whereas a more detailed model of the system might not be suited to machine reasoning, depending on its implementation). Each `Interaction` contains `Participation` objects that indicate the roles of the `FunctionalComponent` objects involved in the `Interaction`.

#### The `models` property

The `models` property is OPTIONAL and MAY specify a set of `URI` references to `Model` objects.

`Model` objects are placeholders that link `ModuleDefinition` objects to computational models of any format. A `ModuleDefinition` object can link to more than one `Model` since each might encode system behavior in a different way or at a different level of detail.

#### Serialization

The serialization of `ModuleDefinition` has the following form:

```
<sbol:ModuleDefinition rdf:about="...">
  ... properties inherited from identified ...
  zero or more  <sbol:role rdf:resource="..."/> elements
  zero or more  <sbol:model rdf:resource="..."/> elements
  zero or more <sbol:functionalComponent>
                 <sbol:FunctionalComponent rdf:about="...">...</sbol:FunctionalComponent >
               </sbol:functionalComponent> elements
  zero or more <sbol:module>
                 <sbol:Module rdf:about="...">...</sbol:Module>
               </sbol:module> elements
  zero or more <sbol:interaction>
                 <sbol:Interaction rdf:about="...">...</sbol:Interaction>
               </sbol:interaction> elements
</sbol:ModuleDefinition>
```

The example below shows a simple `ModuleDefinition` containing two components, a `FunctionalComponent` for a DNA sequence encoding constitutive expression of GFP and another for the GFP protein expressed from this sequence, plus an interaction describing that relation.

```
<sbol:ModuleDefinition rdf:about="http://sbolstandard.org/example/md/GFP_expression">
  <sbol:functionalComponent>
    <sbol:FunctionalComponent rdf:about="http://sbolstandard.org/example/md/GFP_expression/GFP_protein">
      <sbol:definition rdf:resource="http://sbolstandard.org/example/GFP"/>
      <sbol:access rdf:resource="http://sbols.org/v2#public"/>
      <sbol:direction rdf:resource="http://sbols.org/v2#output"/>
    </sbol:FunctionalComponent>
  </sbol:functionalComponent>
  <sbol:functionalComponent>
    <sbol:FunctionalComponent rdf:about="http://sbolstandard.org/example/md/GFP_expression/Constitutive_GFP">
      <sbol:definition rdf:resource="http://sbolstandard.org/example/GFP_generator"/>
      <sbol:access rdf:resource="http://sbols.org/v2#public"/>
      <sbol:direction rdf:resource="http://sbols.org/v2#none"/>
    </sbol:FunctionalComponent>
  </sbol:functionalComponent>
  <sbol:interaction>
    <sbol:Interaction rdf:about="http://sbolstandard.org/example/md/GFP_expression/express_GFP">
      ...
    </sbol:Interaction>
```

```
  </sbol:interaction>
</sbol:ModuleDefinition>
```

#### 7.9.1 Module

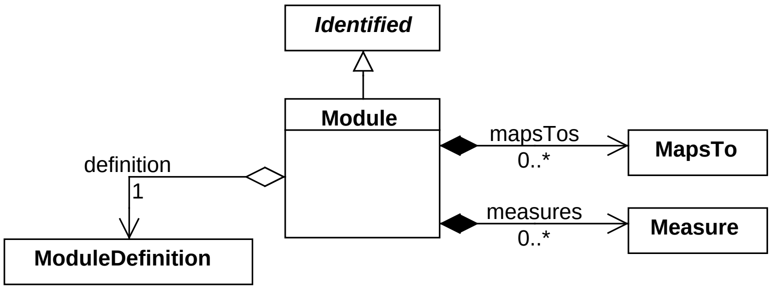

***Figure 16:*** *Diagram of the `Module` class and its associated properties.*

The `Module` class represents the usage or occurrence of a `ModuleDefinition` within a larger design (that is, another `ModuleDefinition`).

##### *The* `definition` *property*

The `definition` property is a REQUIRED `URI` that refers to the `ModuleDefinition` for the `Module`.

The `definition` property MUST NOT refer to the same `ModuleDefinition` as that which contains the `Module`. Furthermore, `Module` objects MUST NOT form a cyclical chain of references via their `definition` properties and the `ModuleDefinition` objects that contain them. For example, consider the `Module` objects $A$ and $B$ and the `ModuleDefinition` objects $X$ and $Y$. The reference chain "$X$ contains $A$, $A$ is defined by $Y$, $Y$ contains $B$, and $B$ is defined by $X$" is cyclical.

##### *The* `mapsTo` *property*

The `mapsTos` property is an OPTIONAL set of `MapsTo` objects that refer to and link `ComponentInstance` objects together within the heterarchy of `Module`, `ModuleDefinition`, `ComponentInstance`, and `ComponentDefinition` objects.

Section 7.7.3 contains a detailed description of the `MapsTo` class.

2.3.0

##### *The* `measures` *property*

The `measures` property is OPTIONAL and MAY contain a set of `Measure` objects. Section 13.2.1 contains a more detailed description of the `Measure` class.

##### *Serialization*

The serialization of `Module`s has the following form.

```
<sbol:Module rdf:about="...">
  ... properties inherited from identified ...
  one          <sbol:definition rdf:resource="..."/> element
  zero or more <sbol:mapsTo>
                 <sbol:MapsTo rdf:about="...">...</sbol:MapsTo>
               </sbol:mapsTo> element
</sbol:Module>
```

The example below specifies a TetR inverter that is being used as a part of a genetic toggle switch:

```
<sbol:Module rdf:about="http://sbolstandard.org/example/toggle_switch/tetr_inverter">
  <sbol:definition rdf:resource="http://sbolstandard.org/example/tetr_inverter"/>
  ...
</sbol:Module>
```

#### 7.9.2 FunctionalComponent

A FunctionalComponent is an instance of a ComponentDefinition being used as part of a ModuleDefinition. The ModuleDefinition describes how the that describes how the FunctionalComponent interacts with others and summarizes their aggregate function.

The FunctionalComponent class inherits from the ComponentInstance class and therefore has the definition, access, and mapsTos properties. In addition, it has a direction property that specifies whether it serves as an input, output, both, or neither with regards to the ModuleDefinition that contains it.

##### *The* **direction** *property*

Each FunctionalComponent MUST specify via the direction property whether it serves as an input, output, both, or neither for its parent ModuleDefinition object. The value for this property MUST be one of the URIs given in Table 12.

| Direction URI | Description |
|---|---|
| http://sbols.org/v2#in | Indicates that the FunctionalComponent is an input. |
| http://sbols.org/v2#out | Indicates that the FunctionalComponent is an output. |
| http://sbols.org/v2#inout | Indicates that the FunctionalComponent is both an input and output |
| http://sbols.org/v2#none | Indicates that the FunctionalComponent is neither an input or output. |

***Table 12:*** *REQUIRED URIs for the direction property.*

The direction property is a means to encode how a designer thinks about the “purpose” of a connection in a system. In SBOL, such a connection is represented with a FunctionalComponent, and a system is represented as with a ModuleDefinition. For example, consider a system that has been designed to sense the concentration of the cell-to-cell signaling molecule $3OC_6HSL$ and report it via the concentration of another gene product. In this system, the concentration of $3OC_6HSL$ is being sensed by the system, so the FunctionalComponent for $3OC_6HSL$ would have a direction of “input.” In turn, the concentration of the reporter gene product is intended to be read/consumed by other biological systems, so the FunctionalComponent for this product would have a direction of “output.” The CDS encoding the product, however, is not intended to directly transfer information into or out of the ModuleDefinition for the system, so its FunctionalComponent would have a direction of “neither.”

##### *Serialization*

The serialization of a FunctionalComponent has the following form.

```
<sbol:FunctionalComponent rdf:about="...">
  ... properties inherited from identified ...
  one          <sbol:definition rdf:resource="..."/> element
  one          <sbol:access rdf:resource="..."/> element
  one          <sbol:direction rdf:resource="..."/> element
  zero or more <sbol:mapsTo rdf:resource="..."/> elements
</sbol:FunctionalComponent>
```

In the example below, the functional component is defined as a public input/output. The component refers to the `Part:BBa_R0010` promoter from the iGEM Parts Registry.

```
<sbol:FunctionalComponent rdf:about="http://sbolstandard.org/example/laci_inverter/promoter">
  <sbol:definition rdf:resource="http://www.partsregistry.org/BBa_R0010"/>
  <sbol:access rdf:resource="http://sbols.org/v2#public"/>
  <sbol:direction rdf:resource="http://sbols.org/v2#inout"/>
</sbol:FunctionalComponent>
```

#### *7.9.3 Interaction*

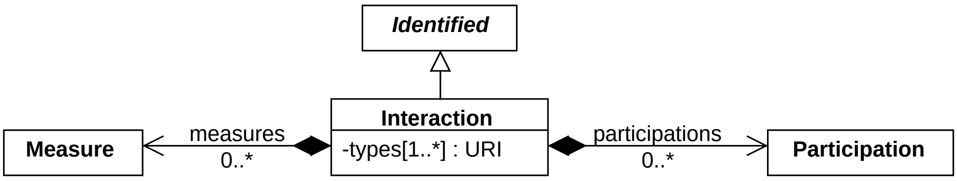

***Figure 17:*** *Diagram of the `Interaction` class and its associated properties.*

The `Interaction` class provides more detailed description of how the `FunctionalComponent` objects of a `ModuleDefinition` are intended to work together. For example, this class can be used to represent different forms of genetic regulation (e.g., transcriptional activation or repression), processes from the central dogma of biology (e.g. transcription and translation), and other basic molecular interactions (e.g., non-covalent binding or enzymatic phosphorylation). Each `Interaction` includes a `types` property that refers to descriptive ontology terms and a `participations` property that describes which `FunctionalComponent` objects participate in the `Interaction`.

##### *The `types` property*

The `types` property is a REQUIRED set of `URI`s that describes the behavior represented by an `Interaction`.

The `types` property MUST contain one or more `URI`s that MUST identify terms from appropriate ontologies. It is RECOMMENDED that exactly one `URI` contained by the `types` property refer to a term from the occurring entity
2.0.1 branch of the Systems Biology Ontology (SBO). (See `http://www.ebi.ac.uk/sbo/main/`) Table 13 provides a list of possible SBO terms for the `types` property and their corresponding `URI`s.

2.0.1

| Interaction Type | URI for SBO Term |
|---|---|
| Inhibition | `http://identifiers.org/biomodels.sbo/SBO:0000169` |
| Stimulation | `http://identifiers.org/biomodels.sbo/SBO:0000170` |
| Biochemical Reaction | `http://identifiers.org/biomodels.sbo/SBO:0000176` |
| Non-Covalent Binding | `http://identifiers.org/biomodels.sbo/SBO:0000177` |
| Degradation | `http://identifiers.org/biomodels.sbo/SBO:0000179` |
| Genetic Production | `http://identifiers.org/biomodels.sbo/SBO:0000589` |
| Control | `http://identifiers.org/biomodels.sbo/SBO:0000168` |

***Table 13:*** *SBO terms to specify the `types` property of an `Interaction`.*

If an `Interaction` is well described by one of the terms from Table 13, then its `types` property MUST contain the `URI` that identifies this term. Lastly, if the `types` property of an `Interaction` contains multiple `URI`s, then they MUST identify non-conflicting terms. For example, the SBO terms “stimulation” and “inhibition” would conflict.

#### *The participations property*

The participations property is an OPTIONAL and MAY contain a set of Participation objects, each of which identifies the roles that its referenced FunctionalComponent plays in the Interaction.

Even though an Interaction generally contains at least one Participation, the case of zero Participation objects is allowed because it is plausible that a designer might want to specify that an Interaction will exist, even if its participants have not yet been determined.

2.3.0

#### *The measures property*

The measures property is OPTIONAL and MAY contain a set of Measure objects. Section 13.2.1 contains a more detailed description of the Measure class.

#### *Serialization*

The serialization of an Interaction has the following form.

```
<sbol:Interaction rdf:about="...">
  ... properties inherited from identified ...
  one or more  <sbol:type rdf:resource="..."/> elements
  zero or more <sbol:participation>
                 <sbol:Participation rdf:about="...">...</sbol:Participation>
               </sbol:participation> elements
</sbol:Interaction>
```

The example below shows an Interaction representing an inhibition relationship (SBO:0000169) between a repressor (SBO:0000020, full Participation details shown) and a promoter:

```
<sbol:Interaction rdf:about="http://sbolstandard.org/example/laci_inverter/LacI_pLacI">
  <sbol:type rdf:resource="http://identifiers.org/biomodels.sbo/SBO:0000169"/>
  <sbol:participation>
    <sbol:Participation rdf:about="http://sbolstandard.org/example/laci_inverter/LacI_pLacI/P03023">
      <sbol:role rdf:resource="http://identifiers.org/biomodels.sbo/SBO:0000020"/>
      <sbol:participant rdf:resource="http://sbolstandard.org/example/laci_inverter/TF"/>
    </sbol:Participation>
  </sbol:participation>
  <sbol:participation>
    <sbol:Participation rdf:about="...">
      ...
    </sbol:Participation>
  </sbol:participation>
</sbol:Interaction>
```

2.0.1

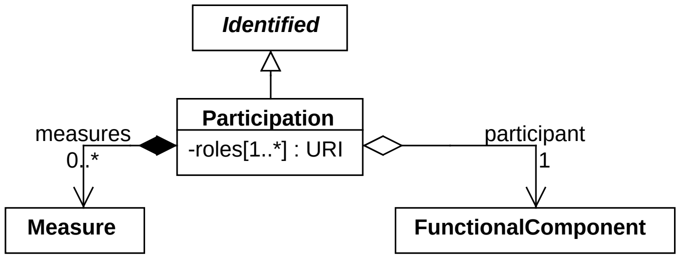

***Figure 18:*** *Diagram of the Participation class and its associated properties.*

#### 7.9.4 Participation

Each Participation represents how a particular FunctionalComponent behaves in its parent Interaction.

##### The roles property

2.0.1 The roles property is a REQUIRED set of URIs that describes the behavior of a Participation (and by extension its referenced FunctionalComponent) in the context of its parent Interaction.

The roles property MUST contain one or more URIs that MUST identify terms from appropriate ontologies. It is RECOMMENDED that exactly one URI contained by the roles property refer to a term from the participant role branch of the SBO. Table 14 provides a list of possible SBO terms for the roles property and their corresponding URIs.

| Participation Role | URI for SBO Term | Interaction Types |
|---|---|---|
| Inhibitor | http://identifiers.org/biomodels.sbo/SBO:0000020 | Inhibition |
| Inhibited | http://identifiers.org/biomodels.sbo/SBO:0000642 | Inhibition |
| Stimulator | http://identifiers.org/biomodels.sbo/SBO:0000459 | Stimulation |
| Stimulated | http://identifiers.org/biomodels.sbo/SBO:0000643 | Stimulation |
| Reactant | http://identifiers.org/biomodels.sbo/SBO:0000010 | Non-Covalent Binding, Degradation Biochemical Reaction |
| Product | http://identifiers.org/biomodels.sbo/SBO:0000011 | Non-Covalent Binding, Genetic Production, Biochemical Reaction |
| Promoter | http://identifiers.org/biomodels.sbo/SBO:0000598 | Inhibition, Stimulation, Genetic Production |
| Modifier | http://identifiers.org/biomodels.sbo/SBO:0000019 | Biochemical Reaction, Control |
| Modified | http://identifiers.org/biomodels.sbo/SBO:0000644 | Biochemical Reaction, Control |
| Template | http://identifiers.org/biomodels.sbo/SBO:0000645 | Genetic Production |

***Table 14:*** *SBO terms to specify the roles property of a Participation.*

If a Participation is well described by one of the terms from Table 14, then its roles property MUST contain the URI that identifies this term. Also, if a Participation belongs to an Interaction that has a type listed in Table 13,
2.0.1 then the Participation SHOULD have a role that is cross-listed with this type in Table 14. Lastly, if the roles property of a Participation contains multiple URIs, then they MUST identify non-conflicting terms. For example, the SBO terms “stimulator” and “inhibitor” would conflict.

##### The participant property

The participant property MUST specify precisely one FunctionalComponent object that plays the designated role in its parent Interaction object.

2.3.0
#### The measures property

The measures property is OPTIONAL and MAY contain a set of Measure objects. Section 13.2.1 contains a more detailed description of the Measure class.

##### Serialization

The serialization of Participation objects has the following form.

```
<sbol:Participation rdf:about="...">
  ... properties inherited from identified ...
  zero or more <sbol:role rdf:resource="..."/> elements
  one          <sbol:participant rdf:resource="..."/> element
</sbol:Participation>
```

In the example below, the role of participating `FunctionalComponent` is defined to be `inhibitor`, using the `SBO:0000020` term. This component is specified using the `participant` property of the `Participation` entity.

```
<sbol:Participation rdf:about="http://sbolstandard.org/example/laci_inverter/LacI_pLacI/P03023">
  <sbol:role rdf:resource="http://identifiers.org/biomodels.sbo/SBO:0000020"/>
  <sbol:participant rdf:resource="http://sbolstandard.org/example/laci_inverter/TF"/>
</sbol:Participation>
```

### 7.10 Collection

The `Collection` class is a class that groups together a set of `TopLevel` objects that have something in common. Some examples of `Collection` objects:

- Results of a query to find all `ComponentDefinition` objects in a repository that function as promoters.
- A set of `ModuleDefinition` objects representing a library of genetic logic gates.
- A `ModuleDefinition` for a complex design, and all of the `ModuleDefinition`, `ComponentDefinition`, `Sequence`, and `Model` objects used to provide its full specification.

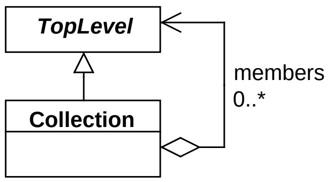

***Figure 19:*** *Diagram of the `Collection` class and its associated properties.*

#### *The `members` property*

The `members` property of a `Collection` is OPTIONAL and MAY contain a set of `URI` references to zero or more `TopLevel` objects.

#### *Serialization*

The serialization of a `Collection` has the following form:

```
<sbol:Collection rdf:about="...">
  ... properties inherited from identified ...
  zero or more <sbol:member rdf:resource="..."/> element
</sbol:Collection>
```

The example below shows the serialization of a `Collection` object grouping together a library of constitutive promoters.

```
<?xml version="1.0" ?>
<rdf:RDF xmlns:rdf="http://www.w3.org/1999/02/22-rdf-syntax-ns#" xmlns:dcterms="http://purl.org/dc/terms/" xmlns:prov="http://www.
      w3.org/ns/prov#" xmlns:sbol="http://sbols.org/v2#">
  <sbol:Collection rdf:about="http://parts.igem.org/Promoters/Catalog/Anderson">
    <sbol:persistentIdentity rdf:resource="http://parts.igem.org/Promoters/Catalog/Anderson"/>
    <sbol:displayId>Anderson</sbol:displayId>
    <dcterms:title>Anderson promoters</dcterms:title>
```

```
    <dcterms:description>The Anderson promoter collection</dcterms:description>
    <sbol:member rdf:resource="http://partsregistry.org/Part:BBa_J23119"/>
    ...
    <sbol:member rdf:resource="http://partsregistry.org/Part:BBa_J23118"/>
  </sbol:Collection>
</rdf:RDF>
```

2.2.0

### 7.11 CombinatorialDerivation

[New in 2.2.0; see SEP 007: https://github.com/SynBioDex/SEPs/blob/master/sep_007.md]

The purpose of the CombinatorialDerivation class is to specify combinatorial genetic designs without having to specify every possible design variant. For example, a CombinatorialDerivation can be used to specify a library of reporter gene variants that include different promoters and RBSs without having to specify a ComponentDefinition for every possible combination of promoter, RBS, and CDS in the library. ComponentDefinition objects that realize a CombinatorialDerivation can be derived in accordance with the class properties template, variableComponents, and strategy (see Figure 20).

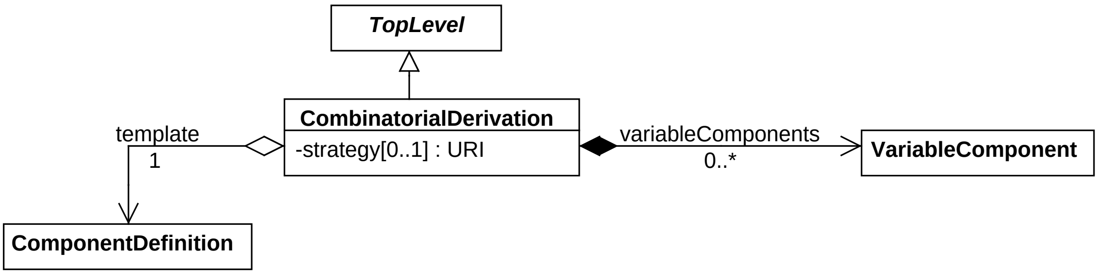

***Figure 20:*** *Diagram of the CombinatorialDerivation class and its associated properties.*

#### *The `template` property*

The template property is REQUIRED and MUST contain a URI that refers to a ComponentDefinition. This ComponentDefinition is expected to serve as a template for the derivation of new ComponentDefinition objects. Consequently, its components property SHOULD contain one or more Component objects that describe its substructure (referred to hereafter as template Component objects), and its sequenceConstraints property MAY also contain one or more SequenceConstraint objects that constrain this substructure.

When a ComponentDefinition is derived in accordance with a CombinatorialDerivation, the wasDerivedFroms property of the derived ComponentDefinition SHOULD refer to the CombinatorialDerivation. When multiple ComponentDefinition objects are derived in accordance with the same CombinatorialDerivation, they MAY be referred to by the members property of a Collection, in which case the wasDerivedFroms property of the Collection SHOULD also refer to this CombinatorialDerivation.

If the types property of the template ComponentDefinition contains one or more URIs, then the types property of the derived ComponentDefinition SHOULD also contain those URIs. The same holds true for the roles properties of these ComponentDefinition objects.

#### *The `variableComponents` property*

The variableComponents property is OPTIONAL and MAY contain a set of VariableComponent objects. These VariableComponent objects are expected to denote the choices available when deriving the substructure of a new ComponentDefinition in accordance with a CombinatorialDerivation. The variableComponents property

MUST NOT contain two or more VariableComponent objects that refer to the same template Component via their variable properties.

If the variable property of one of these VariableComponent objects refers to a template Component, then the components property of the derived ComponentDefinition SHOULD contain as many Component objects derived from the template Component as specified by the operator property of the VariableComponent (see Table 16). In addition, the definition properties of these derived Component objects MUST refer to ComponentDefinition objects specified by the variants, variantCollections, or variantDerivations property of the VariableComponent.

If no variable property of one of these VariableComponent objects refers to a template Component, then the components property of the derived ComponentDefinition SHOULD contain exactly one Component with a wasDerivedFroms property that refers to the template Component. The definition property of this derived Component MUST refer to the ComponentDefinition referred to by the definition property of the template Component.

Finally, all of these derived Component objects MUST follow the restriction properties of any SequenceConstraint objects that refer to their corresponding template Component objects.

#### The `strategy` property

The strategy property is OPTIONAL and has a data type of URI. Table 15 provides a list of REQUIRED strategy URIs. If the strategy property is not empty, then it MUST contain a URI from Table 15. This property recommends how many ComponentDefinition objects a user SHOULD derive from the template ComponentDefinition.

| Strategy URI | Description |
|---|---|
| http://sbols.org/v2#enumerate | A user SHOULD derive all possible ComponentDefinition objects specified by the CombinatorialDerivation. |
| http://sbols.org/v2#sample | A user SHOULD derive a subset of all possible ComponentDefinition objects specified by CombinatorialDerivation. The manner in which this subset is chosen is for the user to decide. |

**Table 15:** *REQUIRED URIs for the strategy property.*

2.2.0
## Serialization

The serialization of a CombinatorialDerivation MUST have the following form:

```
<sbol:CombinatorialDerivation rdf:about="...">
  ... properties inherited from identified ...
  one <sbol:template rdf:resource="..."/> element
  zero or more  <sbol:variableComponent>
                  <sbol:VariableComponent rdf:about="...">...</sbol:VariableComponent>
                </sbol:variableComponent> elements
  zero or one <sbol:strategy rdf:resource="..."/> element
</sbol:CombinatorialDerivation>
```

2.2.0 The example below shows the serialization of a CombinatorialDerivation that specifies the combinatorial design for a library of GFP reporter variants. Each GFP reporter ComponentDefinition sampled from this CombinatorialDerivation would be driven by one of two possible glucose-sensitive promoters and contain one GFP variant from a library of CDSs.

```
<?xml version="1.0" ?>
<rdf:RDF xmlns:rdf="http://www.w3.org/1999/02/22-rdf-syntax-ns#" xmlns:dcterms="http://purl.org/dc/terms/" xmlns:prov="http://www.
     w3.org/ns/prov#" xmlns:sbol="http://sbols.org/v2#">
  <sbol:CombinatorialDerivation rdf:about="http://www.sbolstandard.org/gfp_reporter_derivation/1.0.0">
    <sbol:persistentIdentity rdf:resource="http://www.sbolstandard.org/gfp_reporter_derivation"/>
```

```
    <sbol:displayId>gfp_reporter_derivation</sbol:displayId>
    <dcterms:title>GFP Reporter Derivation</dcterms:title>
    <sbol:template rdf:resource="http://www.sbolstandard.org/gfp_reporter_template/1.0.0"/>
    <sbol:strategy rdf:resource="http://sbols.org/v2#sample"/>
    <sbol:variableComponent>
      <sbol:VariableComponent rdf:about="http://www.sbolstandard.org/gfp_reporter_derivation/promoter_variable/1.0.0">
        <sbol:operator rdf:resource="http://sbols.org/v2#one"/>
        <sbol:variable rdf:resource="http://www.sbolstandard.org/gfp_reporter_template/p1/1.0.0"/>
        <sbol:variant rdf:resource="http://www.sbolstandard.org/glucose_sensitive_1/1.0.0"/>
        <sbol:variant rdf:resource="http://www.sbolstandard.org/glucose_sensitive_2/1.0.0"/>
      </sbol:VariableComponent>
    </sbol:variableComponent>
    <sbol:variableComponent>
      <sbol:VariableComponent rdf:about="http://www.sbolstandard.org/gfp_reporter_derivation/cds_variable/1.0.0">
        <sbol:operator rdf:resource="http://sbols.org/v2#one"/>
        <sbol:variable rdf:resource="http://www.sbolstandard.org/gfp_reporter_template/c1/1.0.0"/>
        <sbol:variantCollection rdf:resource="http://www.sbolstandard.org/gfp_cds_library/1.0.0"/>
      </sbol:VariableComponent>
    </sbol:variableComponent>
  </sbol:CombinatorialDerivation>
</rdf:RDF>
```

2.2.0

#### 7.11.1 VariableComponent

The VariableComponent class can be used to specify a choice of ComponentDefinition objects for any new Component derived from a template Component in the template ComponentDefinition. This specification is made using the class properties variable, variants, variantCollections, and variantDerivations (see Figure 21). While the variants, variantCollections, and variantDerivations properties are OPTIONAL, at least one of them MUST NOT be empty. If these properties contain duplicate URIs, then they SHOULD be treated as a single URI for the purpose of selection. For example, if the variants property contains two copies of URI $A$ and one copy of URI $B$, $A$ SHOULD NOT be selected twice during enumeration, and it SHOULD NOT be selected twice as much as $B$ during sampling.

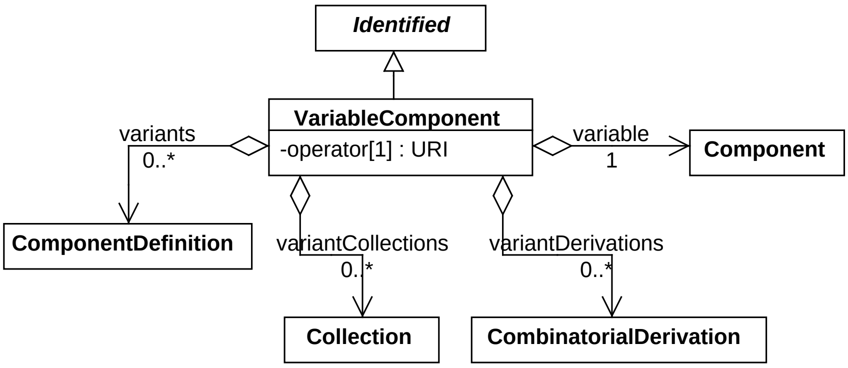

***Figure 21:** Diagram of the VariableComponent class and its associated properties.*

##### The variable property

The variable property is REQUIRED and MUST contain a URI that refers to a template Component in the template ComponentDefinition. If the wasDerivedFroms property of a Component refers to this template Component, then the definition property of the derived Component MUST refer to either (1) a ComponentDefinition referred to by the variants property of the VariableComponent, (2) a ComponentDefinition from a Collection referred to by the variantCollections property of the VariableComponent, or (3) a ComponentDefinition derived from a

CombinatorialDerivation referred to by the variantDerivations property of the VariableComponent.

If the roles property of the template Component contains one or more URIs, then the roles property of the derived Component SHOULD also contain those URIs.

#### The variants property

The variants property is OPTIONAL and MAY contain zero or more URIs that each refer to a ComponentDefinition. This property specifies individual ComponentDefinition objects to serve as options when deriving a new Component from the template Component.

#### The variantCollections property

The variantCollections property is OPTIONAL and MAY contain zero or more URIs that each refer to a Collection. The members property of each Collection referred to in this way MUST NOT be empty. This property enables the convenient specification of existing groups of ComponentDefinition objects to serve as options when deriving a new Component from the template Component.

#### The variantDerivations property

The variantDerivations property is OPTIONAL and MAY contain zero or more URIs that each refer to a CombinatorialDerivation. This property enables the convenient specification of ComponentDefinition objects derived in accordance with another CombinatorialDerivation to serve as options when deriving a new Component from the template Component. The variantDerivations property of a VariableComponent MUST NOT refer to the CombinatorialDerivation that contains this VariableComponent. Furthermore, VariableComponent objects MUST NOT form a cyclical chain of references via their variantDerivations properties and the CombinatorialDerivation objects that contain them. For example, consider the VariableComponent objects A and B and the CombinatorialDerivation objects X and Y. The reference chain X contains A, A has variant derivation Y, Y contains B, and B has variant derivation X is cyclical.

#### The operator property

The operator property is REQUIRED and has a data type of URI. This property specifies how many Component objects SHOULD be derived from the template Component during the derivation of a new ComponentDefinition. The URI value of this property MUST come from the URIs provided in Table 16.

| Operator URI | Description |
|---|---|
| http://sbols.org/v2#zeroOrOne | No more than one Component in the derived ComponentDefinition SHOULD have a wasDerivedFroms property that refers to the template Component. |
| http://sbols.org/v2#one | Exactly one Component in the derived ComponentDefinition SHOULD have a wasDerivedFroms property that refers to the template Component. |
| http://sbols.org/v2#zeroOrMore | Any number of Component objects in the derived ComponentDefinition MAY have wasDerivedFroms properties that refer to the template Component. |
| http://sbols.org/v2#oneOrMore | At least one Component in the derived ComponentDefinition SHOULD have a wasDerivedFroms property that refers to the template Component. |

***Table 16:*** *REQUIRED URIs for the operator property.*

2.2.0

### Serialization

The serialization of a VariableComponent MUST have the following form:

```
<sbol:VariableComponent rdf:about="...">
  ... properties inherited from identified ...
```

```
  one <sbol:variable rdf:resource="..."/> element
  zero or more <sbol:variant rdf:resource="..."/> element
  zero or more <sbol:variantCollection rdf:resource="..."/> element
  zero or more <sbol:variantDerivation rdf:resource="..."/> element
  one <sbol:operator rdf:resource="..."/> element
</sbol:VariableComponent>
```

2.2.0 The example below shows the serialization of a VariableComponent that specifies a choice of one or more operator sites from a library of variants for the derivation of a synthetic CMV promoter.

```
<sbol:VariableComponent rdf:about="http://www.sbolstandard.org/cmv_promoter_derivation/operator_variable/1.0.0">
  <sbol:operator rdf:resource="http://sbols.org/v2#oneOrMore"/>
  <sbol:variable rdf:resource="http://www.sbolstandard.org/cmv_promoter_template/o1/1.0.0"/>
  <sbol:variantCollection rdf:resource="http://www.sbolstandard.org/operator_library/1.0.0"/>
</sbol:VariableComponent>
```

2.2.0

### 7.12 Implementation

[New in 2.2.0; see SEP 019: https://github.com/SynBioDex/SEPs/blob/master/sep_019.md]

An Implementation represents an instance of a synthetic biological construct, and describes the build phase of a design-built-test-learn workflow. Importantly, an Implementation can be associated with a laboratory sample that was already built, or that is to be built in the future. An Implementation can also represent virtual and simulated instances. An Implementation may be linked back to its original design (either a ModuleDefinition or ComponentDefinition) using the wasDerivedFroms property inherited from the Identified superclass. An Implementation may also link to a ModuleDefinition or ComponentDefinition that specifies its realized structure and/or function.

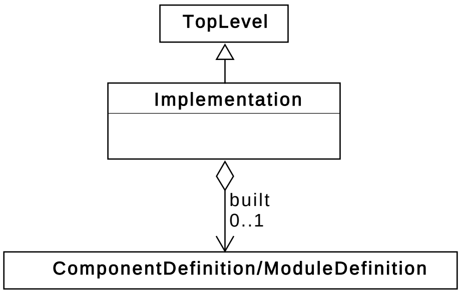

***Figure 22:*** *Diagram of the Implementation class and its associated properties.*

#### *The built property*

The built property is OPTIONAL and MAY contain a URI that MUST refer to a TopLevel object that is either a ComponentDefinition or ModuleDefinition. This ComponentDefinition or ModuleDefinition is intended to describe the actual physical structure and/or functional behavior of the Implementation. When the built property refers to a ComponentDefinition or ModuleDefinition that is also linked to the Implementation via PROV-O properties such as wasDerivedFroms (see Section 13.1), it can be inferred that the actual structure and/or function of the Implementation matches its original design. When the built property refers to a different ComponentDefinition or ModuleDefinition, it can be inferred that the Implementation has deviated from the original design. For example, the latter could be used to document when the DNA sequencing results for an assembled construct do not match the original target sequence.

2.2.0

#### *Serialization*

The serialization of an Implementation has the following form:

```
<sbol:Implementation rdf:about="...">
  ... properties inherited from Identified ...
  one          <sbol:built rdf:resource="..."/> element
</sbol:Implementation>
```

2.2.0 In the example below, an Implementation links back to its target design via the wasDerivedFroms property. Since this particular sample did not match its target structure when synthesized in the lab, a ComponentDefinition representing its mutated structure is linked by the built field.

```
<sbol:Implementation rdf:about="http://examples.org/Implementation/build/1.0.0">
 <sbol:displayId>build</sbol:displayId>
  <sbol:persistentIdentity rdf:resource="http://examples.org/Implementation/build"/>
  <sbol:version>1.0.0</sbol:version>
  <prov:wasDerivedFrom rdf:resource="http://examples.org/ComponentDefinition/design/1.0.0"/>
  <sbol:built rdf:resource="http://examples.org/ComponentDefinition/mutant/1.0.0"/>
</sbol:Implementation>
```

2.2.0

### 7.13 Attachment

[New in 2.2.0; see SEP 018: https://github.com/SynBioDex/SEPs/blob/master/sep_018.md]

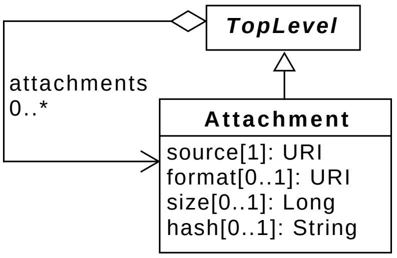

***Figure 23:*** *Diagram of the Attachment class and its associated properties.*

The purpose of the Attachment class is to serve as a general container for data files, especially experimental data files. It provides a means for linking files and metadata to SBOL designs.

The meta-data provided by the Attachment class include the following properties: the source or location of the actual file of the attachment, the format of the file, the size of the file, and the hash for the file.

#### *The source property*

The source property is REQUIRED and MUST contain a URI reference to the source file.

#### *The format property*

The format property is OPTIONAL and MAY contain a URI that specifies the format of the attached file. It is RECOMMENDED that this URI refer to a term from the EMBRACE Data and Methods (EDAM) ontology.

#### *The size property*

The size property is OPTIONAL and MAY contain a long indicating the file size in bytes.

#### *The* **hash** *property*

The `hash` property is OPTIONAL and MAY contain a SHA-1 hash of the file contents represented as a hexadecimal digest.

#### *Serialization*

The serialization of an `Attachment` MUST have the following form:

```
<sbol:Attachment rdf:about="...">
  ... properties inherited from identified ...
  one <sbol:source rdf:resource="..."/> element
  zero or one <sbol:format rdf:resource="..."/> element
  zero or one <sbol:size>...</sbol:size> element
  zero or one <sbol:hash>...</sbol:hash> element
</sbol:Attachment>
```

2.2.0 The example below shows the serialization of an `Attachment` object that refers to a PNG file containing a plot of simulation data. The attachment in this example can be retrieved from a repository via its `source` URI, which is a URL.

```
<?xml version="1.0" ?>
<rdf:RDF xmlns:rdf="http://www.w3.org/1999/02/22-rdf-syntax-ns#" xmlns:dcterms="http://purl.org/dc/terms/" xmlns:prov="http://www.
     w3.org/ns/prov#" xmlns:sbol="http://sbols.org/v2#">
  <sbol:Attachment rdf:about="https://synbiohub.org/public/ACS_SynBio/attachment_00009Les8pDTQCmKA7A5T6">
    <sbol:persistentIdentity rdf:resource="https://synbiohub.org/public/ACS_SynBio/attachment_00009Les8pDTQCmKA7A5T6"/>
    <sbol:displayId>attachment_00009Les8pDTQCmKA7A5T6</sbol:displayId>
    <dcterms:title>NicePlot.png</dcterms:title>
    <sbol:source rdf:resource="https://synbiohub.org/public/ACS_SynBio/attachment_00009Les8pDTQCmKA7A5T6/1/download"/>
    <sbol:format rdf:resource="http://identifiers.org/edam/format_3603"/>
    <sbol:size>31399</sbol:size>
    <sbol:hash>404966a5062857f63edb5c3ba16bb581d7fb6a1e</sbol:hash>
  </sbol:Attachment>
</rdf:RDF>
```

2.3.0

### 7.14 ExperimentalData

[New in 2.3.0; see SEP 021: https://github.com/SynBioDex/SEPs/blob/master/sep_021.md]

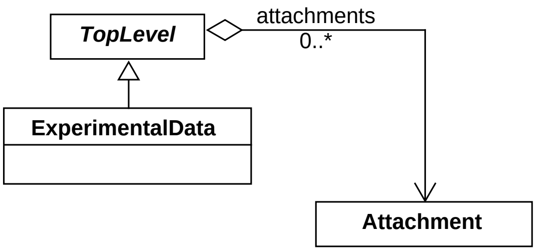

***Figure 24:*** *Diagram of the* `ExperimentalData` *class and its associated properties.*

The purpose of the `ExperimentalData` class is to aggregate links to experimental data files. An `ExperimentalData` is typically associated with a single sample, lab instrument, or experimental condition and can be used to describe the output of the test phase of a design-build-test-learn workflow. For an example of the latter, see Figure 36.

As shown in Figure 24, the `ExperimentalData` class aggregates links to experimental data files using the OPTIONAL `attachments` property that it inherits from the `TopLevel` class.

#### Serialization

The serialization of an ExperimentalData MUST have the following form:

```
<sbol:ExperimentalData rdf:about="...">
  ... properties inherited from TopLevel ...
</sbol:ExperimentalData>
```

2.3.0 The example below shows the serialization of an ExperimentalData that refers to a pair of Attachment objects for FASTQ files from a paired-end DNA sequencing experiment.

```
<?xml version="1.0" ?>
<rdf:RDF xmlns:rdf="http://www.w3.org/1999/02/22-rdf-syntax-ns#" xmlns:dcterms="http://purl.org/dc/terms/" xmlns:prov="http://www.
      w3.org/ns/prov#" xmlns:sbol="http://sbols.org/v2#">
  <sbol:ExperimentalData rdf:about="http://sbolstandard.org/example/X1Y0A0C2286_S1_sequencing_data/1">
    <sbol:persistentIdentity rdf:resource="http://sbolstandard.org/example/X1Y0A0C2286_S1_sequencing_data"/>
    <sbol:displayId>X1Y0A0C2286_S1_sequencing_data</sbol:displayId>
    <dcterms:title>X1Y0A0C2286 Sequencing Data</dcterms:title>
    <sbol:attachment rdf:resource="http://sbolstandard.org/example/X1Y0A0C2286_S1_L001_R1/1"/>
    <sbol:attachment rdf:resource="http://sbolstandard.org/example/X1Y0A0C2286_S1_L001_R2/1"/>
    <prov:wasDerivedFrom rdf:resource="http://sbolstandard.org/example/X1Y0A0C2286/1"/>
  </sbol:ExperimentalData>
</rdf:RDF>
```

2.3.0

### 7.15 Experiment

[New in 2.3.0; see SEP 021: https://github.com/SynBioDex/SEPs/blob/master/sep_021.md]

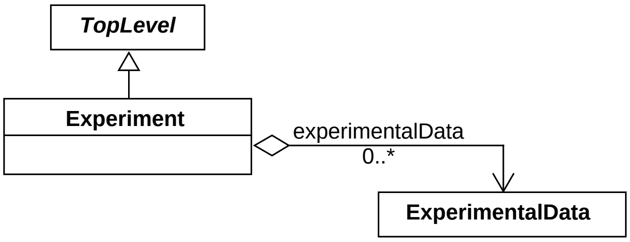

***Figure 25:*** *Diagram of the Experiment class and its associated properties.*

The purpose of the Experiment class is to aggregate experimental data sets for subsequent analysis, usually in accordance with an experimental design.

As shown in Figure 24, the Experiment class aggregates ExperimentalData objects via the experimentalData property.

#### The experimentalData property

The experimentalData property is OPTIONAL and MAY contain a set of URI references to ExperimentalData objects. The same ExperimentalData MAY be referred to by more than one Experiment in this way.

#### Serialization

The serialization of an Experiment MUST have the following form:

```
<sbol:Experiment rdf:about="...">
  ... properties inherited from TopLevel ...
```

```
  zero or more <sbol:experimentalData rdf:resource="..."/> element
</sbol:Experiment>
```

2.3.0 The example below shows the serialization of an Experiment that aggregates the DNA sequencing results for related samples (e.g. that implement related variants of the same genetic design).

```
<?xml version="1.0" ?>
<rdf:RDF xmlns:rdf="http://www.w3.org/1999/02/22-rdf-syntax-ns#" xmlns:dcterms="http://purl.org/dc/terms/" xmlns:prov="http://www.
     w3.org/ns/prov#" xmlns:sbol="http://sbols.org/v2#">
  <sbol:Experiment rdf:about="http://sbolstandard.org/example/sequencing_exp_032619/1">
    <sbol:persistentIdentity rdf:resource="http://sbolstandard.org/example/sequencing_exp_032619"/>
    <sbol:displayId>sequencing_exp_032619</sbol:displayId>
    <dcterms:title>Sequencing Experiment 032619</dcterms:title>
    <sbol:experimentalData rdf:resource="http://sbolstandard.org/example/X1Y0A0C2286_S1_sequencing_data/1"/>
    <sbol:experimentalData rdf:resource="http://sbolstandard.org/example/X1Y0A0C2288_S1_sequencing_data/1"/>
    <sbol:experimentalData rdf:resource="http://sbolstandard.org/example/X1Y0A0C2290_S1_sequencing_data/1"/>
    <sbol:experimentalData rdf:resource="http://sbolstandard.org/example/B1C0C0C1100_S1_sequencing_data/1"/>
  </sbol:Experiment>
</rdf:RDF>
```

### 7.16 Annotation and Extension of SBOL

SBOL does not currently represent all types of biological design data, since many of these data types (e.g., biological context and design performance metrics) lack a clear consensus on their proper representation. In addition, some types of biological data are not directly relevant to design and are therefore outside of the scope of SBOL.

To enable representation of these data, SBOL allows developers to embed custom data within SBOL objects and documents, such that these data can be exchanged without being damaged or lost. This annotation and extension mechanism is designed to enable new types of data to be easily incorporated into the SBOL standard once there is community consensus on their proper representation.

Several methods are supported for connecting the SBOL data model with other types of application-specific data:

- Custom data can be added to an SBOL object by annotating that object with non-conflicting properties. These properties could contain literal data types such as Strings or URIs that require a resolution mechanism to obtain external data. An example is annotating a ComponentDefinition with a property that contains a String description and URI for the parts registry from which its source data was originally imported.
- Custom data in the form of independent objects can be added to an SBOL document by creating GenericTopLevel objects and annotating them as described above. An example is a GenericTopLevel object that is annotated such that it represents a data sheet that describes the performance of a ModuleDefinition in a particular context.
- Finally, just as custom objects can be embedded in an SBOL document, external documents can embed or refer to SBOL objects. Support for this last case is not explicitly provided in this specification. Rather, this case depends on the external non-SBOL system managing its relationship to SBOL and data serialized in RDF/XML, and is included here for completeness.

#### *7.16.1 Annotating SBOL objects*

Each Identified object MAY contain any number of Annotation objects that store data in the form of name/value property pairs. The qName is REQUIRED and MUST contain a QName, which is composed of a namespace, an OPTIONAL prefix, and a local name. The qName property MUST be stored in the data model to allow for proper serialization as described below. The value property is also REQUIRED and MUST contain a literal (i.e., a String, Integer, Double, Boolean), URI, or NestedAnnotations object. A NestedAnnotations object MUST contain nestedQName and nestedURI properties, and it MAY contain an annotations property that contains zero or more Annotation objects.

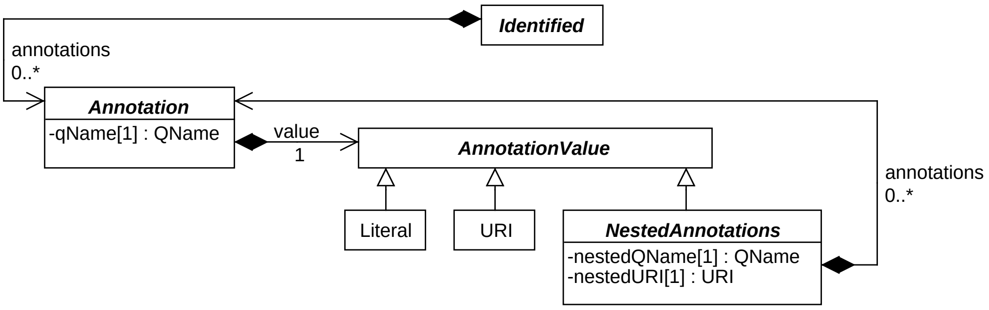

***Figure 26:*** *Diagram of the `Annotation` class and its association with `Identified` and `AnnotationValue` objects, which is used for annotating SBOL entities with application specific data.*

#### *Serialization*

The serialization of an `Annotation` has the following form:

```
<?xml version="1.0" ?>
<rdf:RDF xmlns:rdf="http://www.w3.org/1999/02/22-rdf-syntax-ns#"
  xmlns:sbol="http://sbols.org/v2#"
  xmlns:prefix1="NAMESPACE_1"
  xmlns:prefix2="NAMESPACE_2"
  xmlns:nestedObjectPrefix="A_NESTED_OBJECT_NAMESPACE"
  ...
 >
<sbol:A_TOPLEVELOBJECT rdf:about="...">
              ...
  zero or more <prefix1:LOCAL_NAME_1>A_LITERAL</prefix1:LOCAL_NAME_1> elements
  zero or more <prefix1:LOCAL_NAME_2 rdf:resource=URI/> elements
  zero or more <prefix2:LOCAL_NAME_3>
                <nestedObjectPrefix:NESTED_LOCAL_NAME rdf:about="...">
                  ...
                </nestedObjectPrefix:NESTED_LOCAL_NAME>
              </prefix2:LOCAL_NAME_3> elements
</sbol:TOPLEVELOBJECT>
```

The `qName` property specifies a namespace, prefix, and local part/name. The use of such qualified names is described in detail by the W3C (`http://www.w3.org/TR/1999/REC-xml-names-19990114/#ns-using`). Essentially, the “xmlns” property defines the prefix `String` to use as an alias for the namespace. The prefix can be any `String`. Its use is OPTIONAL, since it simply replaces the full namespace, thereby making the serialization easier for a human to read.

The first form of `Annotation` shown above is for an `Annotation` that contains a `literal` as its `value`. The second form is for an `Annotation` that contains a `URI` as its value. Finally, the third form is for an `Annotation` that contains a `NestedAnnotations` object as its value. In the last case, the `nestedQName` property specifies the nested namespace, nested prefix, and nested local part/name, while the `nestedURI` property species the `URI` for the `NestedAnnotations` object.

The example below shows how the serialization for a promoter `ComponentDefinition` can be annotated with custom data. `Annotation`s are added containing the relevant information from the iGEM Parts Registry. Each property serialization of an `Annotation` is qualified with the `http://www.partsregistry.org/` namespace, which is prefixed using `pr`. The first `Annotation` is named `pr:group`. It specifies the iGEM group that has designed the promoter and has a `String` value. The second `Annotation` is named `pr:experience`. It contains a `URI` value that is serialized as an RDF resource and can be resolved to the information Web page on the Parts Registry for the promoter. Finally, the third `Annotation` is named `pr:information`. It contains a `NestedAnnotations` object that is serialized as shown and includes information about the regulatory details of the promoter using Annotations that correspond to Parts Registry categories.

```
<?xml version="1.0" ?>
<rdf:RDF xmlns:pr="http://partsregistry.org/" xmlns:rdf="http://www.w3.org/1999/02/22-rdf-syntax-ns#" xmlns:dcterms="http://purl.
      org/dc/terms/" xmlns:prov="http://www.w3.org/ns/prov#" xmlns:sbol="http://sbols.org/v2#">
  <sbol:ComponentDefinition rdf:about="http://partsregistry.org/cd/BBa_J23119">
    <sbol:persistentIdentity rdf:resource="http://partsregistry.org/cd/BBa_J23119"/>
    <sbol:displayId>BBa_J23119</sbol:displayId>
    <pr:group>iGEM2006_Berkeley</pr:group>
    <pr:experience rdf:resource="http://parts.igem.org/cgi/partsdb/part_info.cgi?part_name=BBa_J23119"/>
    <pr:information>
      <pr:Information rdf:about="http://partsregistry.org/cd/BBa_J23119/information">
        <pr:sigmafactor>//rnap/prokaryote/ecoli/sigma70</pr:sigmafactor>
        <pr:regulation>//regulation/constitutive</pr:regulation>
      </pr:Information>
    </pr:information>
    <dcterms:title>J23119</dcterms:title>
    <dcterms:description>Constitutive promoter</dcterms:description>
    <sbol:type rdf:resource="http://www.biopax.org/release/biopax-level3.owl#DnaRegion"/>
    <sbol:role rdf:resource="http://identifiers.org/so/SO:0000167"/>
  </sbol:ComponentDefinition>
</rdf:RDF>
```

#### *7.16.2 GenericTopLevel*

Custom data can also be embedded at the top level of an SBOL document. The GenericTopLevel class is used to represent top-level entities whose purpose is to contain a set of annotations that are independent of any other class of SBOL object. Entities that have independent existence and are not recognized by the SBOL standard are deserialized to GenericTopLevel objects. These GenericTopLevel objects can be safely used by tools to exchange non-SBOL data.

As with other TopLevel objects, GenericTopLevel objects MAY include the properties displayId, name, description, etc. The type of data annotating a GenericTopLevel object is indicated using the REQUIRED rdfType property, which MUST contain a QName. As before with the qName property, the rdfType property is used to set the namespace, prefix, and local part/name during serialization.

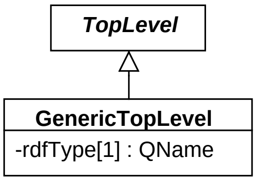

***Figure 27:*** *Diagram of the GenericTopLevel class and its associated properties, which is used for embedding externally defined entities into SBOL documents.*

#### *Serialization*

The serialization of the GenericTopLevel class has the following form, where the prefix, namespace, and local part/name are defined by the rdfType property:

```
<?xml version="1.0" ?>
```

```
<rdf:RDF xmlns:rdf="http://www.w3.org/1999/02/22-rdf-syntax-ns#"
  xmlns:sbol="http://sbols.org/v2#"
  xmlns:applicationPrefix="APPLICATION_NAMESPACE"
  ...
 >
<applicationPrefix:APPLICATION_OBJECT_NAME rdf:about="...">
  ... properties inherited from identified ...
  ... any non-conflicting application-specific properties ...
</applicationPrefix:APPLICATION_OBJECT_NAME>
```

The example below shows how a datasheet object can be added to an SBOL document using the `GenericTopLevel` class. The J23119 promoter `ComponentDefinition` is annotated with the `myapp:datasheet` property that contains the `URI` of a `TopLevel` Datasheet object. The Datasheet object is further annotated with the transcription rate and `URI` for the actual characterization data using the `myapp:transcriptionRate` and `myapp:characterizationData` properties, respectively. As specified by their `rdfType` and `qName` properties, the `TopLevel` Datasheet object and all `Annotation` objects in this example are serialized with the custom `http://www.myapp.org/` namespace and `myapp` prefix.

```
<?xml version="1.0" ?>
<rdf:RDF xmlns:myapp="http://www.myapp.org/" xmlns:rdf="http://www.w3.org/1999/02/22-rdf-syntax-ns#" xmlns:dcterms="http://purl.
      org/dc/terms/" xmlns:prov="http://www.w3.org/ns/prov#" xmlns:sbol="http://sbols.org/v2#">
  <sbol:ComponentDefinition rdf:about="http://www.partsregistry.org/cd/BBa_J23119">
    <sbol:persistentIdentity rdf:resource="http://www.partsregistry.org/cd/BBa_J23119"/>
    <sbol:displayId>BBa_J23119</sbol:displayId>
    <prov:wasDerivedFrom rdf:resource="http://www.partsregistry.org/Part:BBa_J23119"/>
    <myapp:datasheet rdf:resource="http://www.partsregistry.org/gen/datasheet1"/>
    <dcterms:title>J23119</dcterms:title>
    <dcterms:description>Constitutive promoter</dcterms:description>
    <sbol:type rdf:resource="http://www.biopax.org/release/biopax-level3.owl#DnaRegion"/>
    <sbol:role rdf:resource="http://identifiers.org/so/SO:0000167"/>
  </sbol:ComponentDefinition>
  <myapp:Datasheet rdf:about="http://www.partsregistry.org/gen/datasheet1">
    <sbol:persistentIdentity rdf:resource="http://www.partsregistry.org/gen/datasheet1"/>
    <sbol:displayId>datasheet1</sbol:displayId>
    <myapp:characterizationData rdf:resource="http://www.myapp.org/measurement/1"/>
    <myapp:transcriptionRate>1</myapp:transcriptionRate>
    <dcterms:title>Datasheet 1</dcterms:title>
  </myapp:Datasheet>
</rdf:RDF>
```

## 8 Mapping Between SBOL 1.1 and SBOL 2.x

Figure 28 depicts the mapping of SBOL 1.1 classes to SBOL 2.x classes, indicating corresponding classes/properties by color. The SBOL 2.x `Model` and `ModuleDefinition` classes have no SBOL 1.1 equivalent, and thus are not shown. In particular:

- SBOL 1.1 `Collection` objects containing `DnaComponent` objects map to SBOL 2.x `Collection` objects that contain `ComponentDefinition` objects with DNA `types` properties.
- SBOL 1.1 `DnaComponent` objects maps to SBOL 2.x `ComponentDefinition` objects with DNA `types` properties.
- SBOL 1.1 `DnaSequence` objects maps to an SBOL 2.x `Sequence` objects with `IUPAC DNA` `encoding` properties.
- SBOL 1.1 `SequenceAnnotation` objects with `bioStart` and `bioEnd` properties map to SBOL 2.x `SequenceAnnotation` objects that contain `Range` objects.
- SBOL 1.1 `SequenceAnnotation` objects that lack `bioStart` and `bioEnd` properties map to an SBOL 2.x `SequenceAnnotation` objects that contain `GenericLocation` objects.
- Each SBOL 1.1 `SequenceAnnotation` also maps to an SBOL 2.x `Component`, which represents the instantiation or usage of the appropriate `ComponentDefinition`.
- Each SBOL 1.1 `precedes` property maps to an SBOL 2.x `SequenceConstraint` that specifies a precedes `restriction` property.

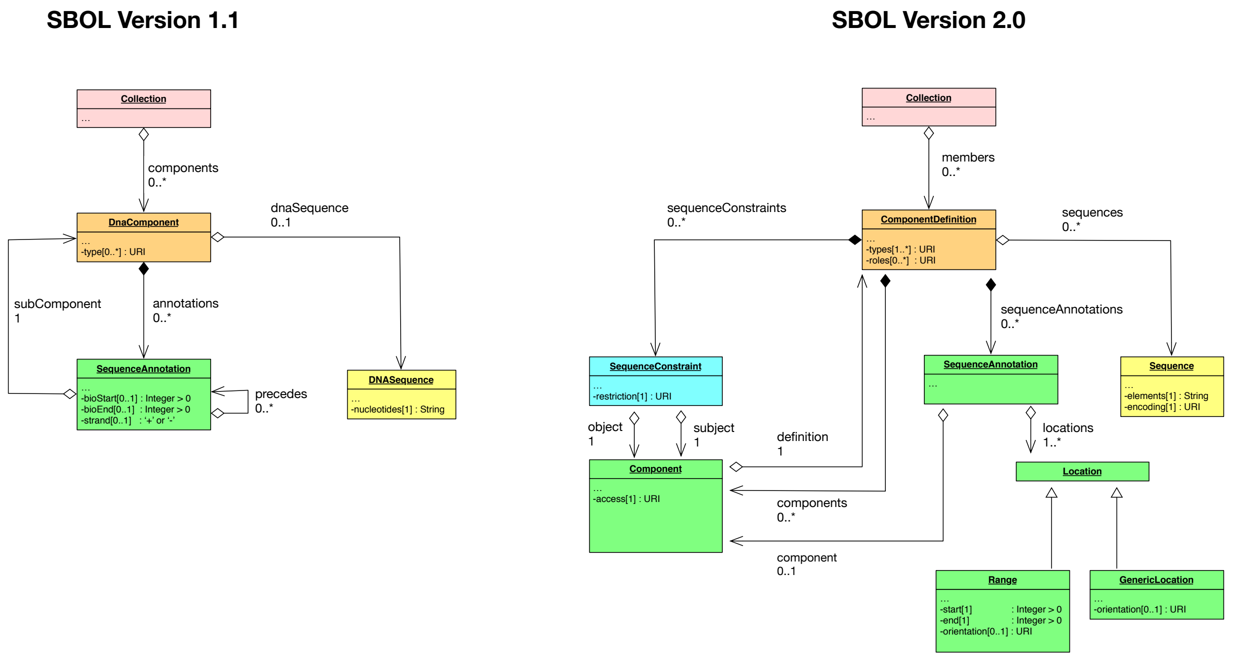

***Figure 28:*** *The mapping from the SBOL 1.1 data model to the SBOL 2.x data model, indicating corresponding classes/properties by color.*

## 9 Data Model Examples

This section illustrates how to use the SBOL data model by specifying the design of a LacI/TetR toggle switch similar to those constructed in Gardner et al. (2000). This design is visualized conceptually in Figure 29 and in detail in Figure 30.

Conceptually, the toggle switch is constructed from two mutually repressing genes. With repressors LacI and TetR, this results in a bi-stable system that will tend to settle into a state where precisely one of the two repressors is strongly expressed, repressing the other. Each of these repressors can have its activity disrupted by a small molecule (IPTG for LacI, aTc for TetR), which enables the system to be “toggled” from one state to the other by dosing it with the appropriate small molecule.

2.2.0

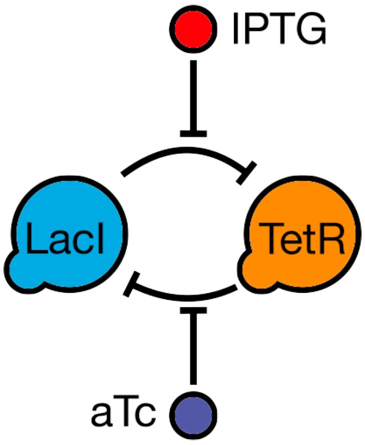

***Figure 29:*** *Conceptual diagram of LacI/TetR toggle switch: the LacI and TetR transcription factors are arranged to mutually repress each other’s expression, creating a bi-stable system. Transition between the two states is triggered by the small-molecule signals aTc (which disrupts TetR repression) and IPTG (which disrupts LacI repression).*

The LacI/TetR toggle switch is modeled in SBOL as two parallel hierarchies of structure and function. The structural hierarchy of the toggle switch is represented using `ComponentDefinition`s:

- The base elements of the hierarchy are DNA components, transcription factor proteins, and small molecules. As an example, Figure 31 is a UML diagram of the `ComponentDefinition` objects that represent these elements.
- Base elements are composed to form more complex structures at the top of the hierarchy, including genes and non-covalent complexes between transcription factor proteins and small molecules. As an example, Figure 32 is a UML diagram of the composite `ComponentDefinition` objects that represent the TetR gene and IPTG-LacI complex.

2.0.1 The functional hierarchy of the toggle switch is represented using `ModuleDefinition`s:

- The base elements of the hierarchy are LacI-dependent repression of TetR expression (the LacI inverter) and TetR-dependent repression of LacI (the TetR inverter). As an example, Figure 33 is a UML diagram of the `ModuleDefinition` that represents the LacI inverter.
- Base elements are composed to form the toggle switch at the top of the hierarchy. As an example, Figure 34 is a UML diagram of the `ModuleDefinition` that represents the toggle switch.

2.0.1

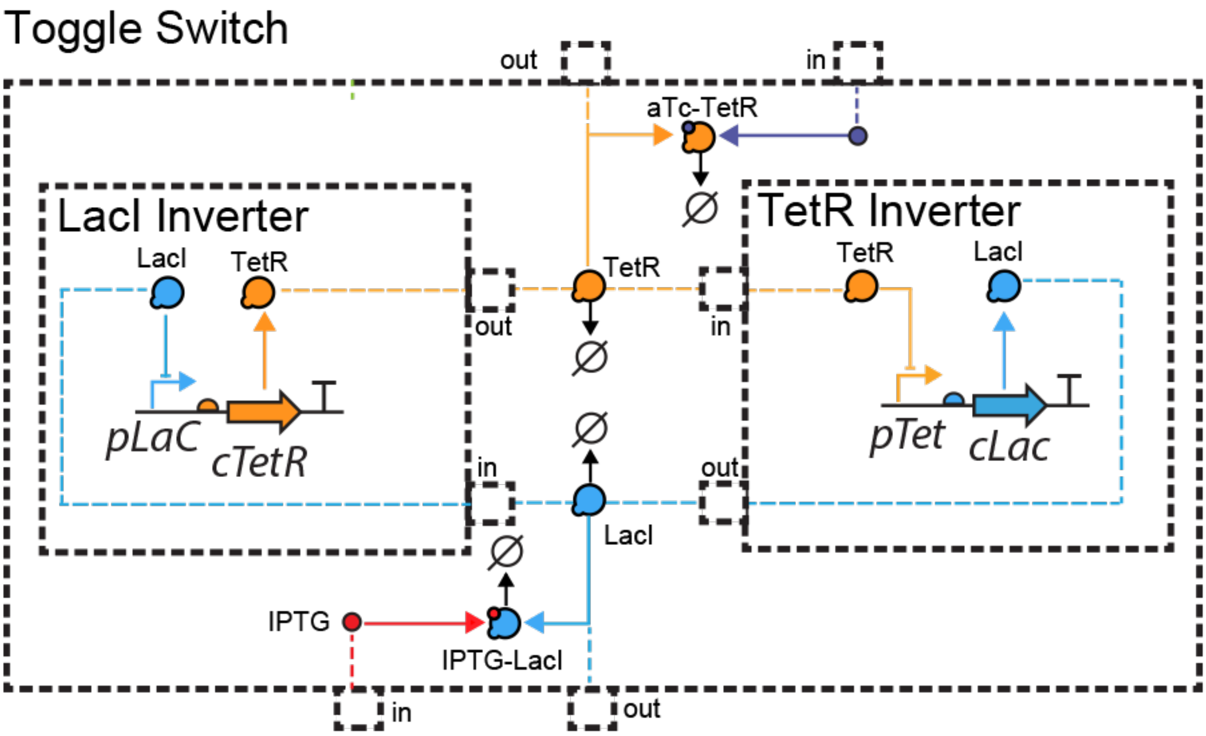

***Figure 30:*** *Design of a LacI/TetR toggle switch. This design is composed of two inverter sub-designs, each containing a single gene. These genes mutually repress each other's expression via their encoded protein transcription factors, LacI and TetR. Furthermore, both LacI and TetR are bound by specific small molecules that sequester them and prevent them from acting as repressors. In this design, arrows represent different molecular interactions, including the repression of pLac via LacI, the non-covalent binding of IPTG to LacI, the transcription of TetR mRNA, and the translation of TetR. Dashed lines serve to map between transcription factors in the inverter sub-designs and those in the overall toggle switch design.*

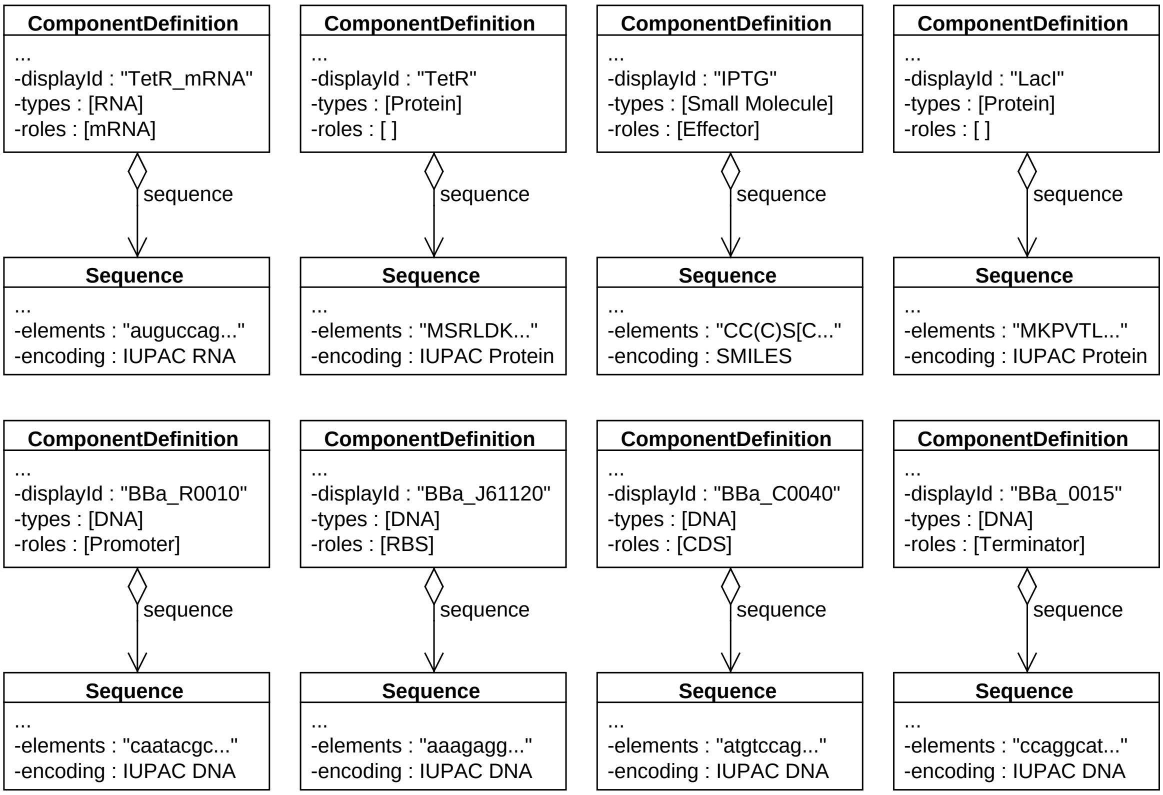

***Figure 31:*** *`ComponentDefinition` objects for the LacI inverter. These include `ComponentDefinition` objects based on DNA parts from the iGEM Parts Registry and `ComponentDefinition` objects that represent TetR mRNA, TetR, LacI, and IPTG. Each `ComponentDefinition` is associated with a `Sequence` that has an `IUPAC DNA/RNA` or `IUPAC protein` `encoding`, except the `ComponentDefinition` of IPTG, which is associated with a `Sequence` that has a `SMILES` `encoding`.*

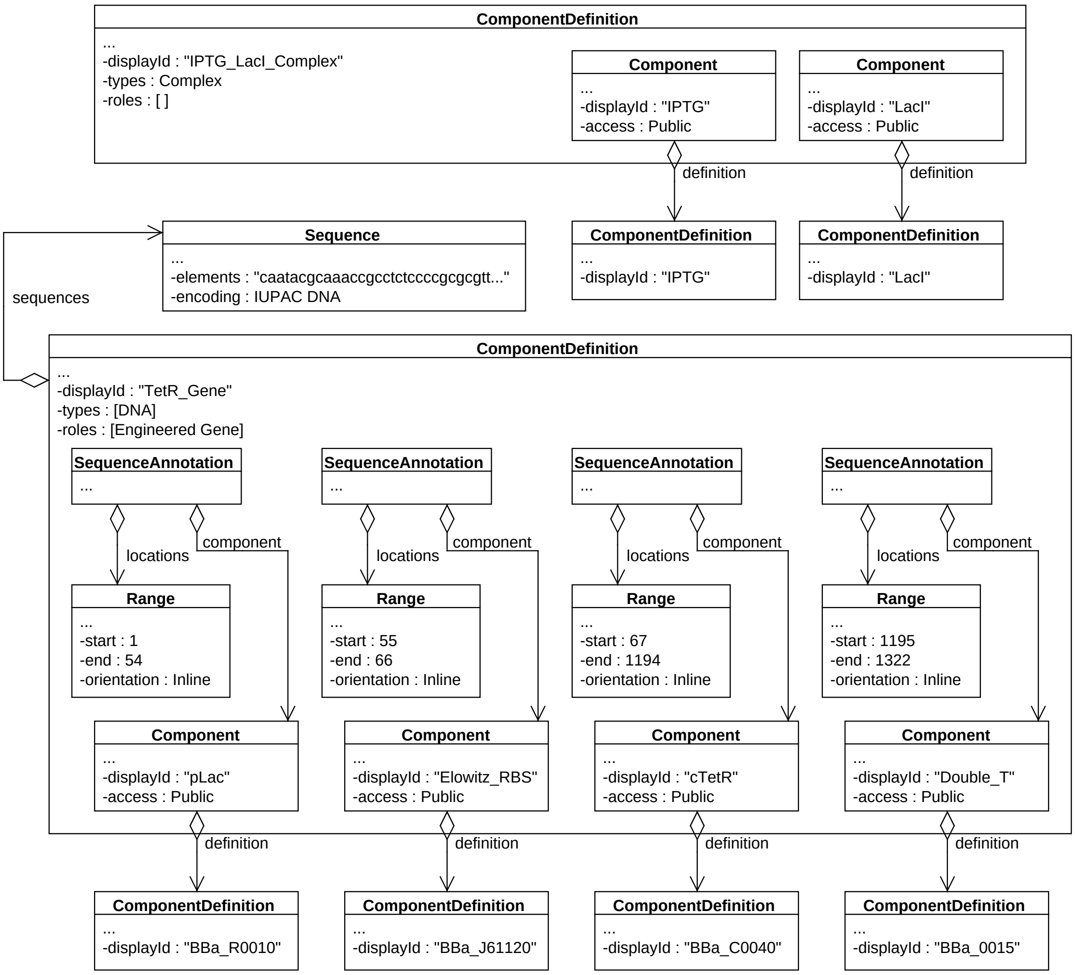

***Figure 32:*** *Composite `ComponentDefinition` objects for the LacI inverter. In the case of the `ComponentDefinition` that represents the TetR gene, its sub-`Component` objects are located as `Range`s along its `Sequence` using `SequenceAnnotation` objects. The `ComponentDefinition` that represents the IPTG-LacI complex, however, has no `Sequence` and its sub-`Component` objects are composed without any data about their relative positions.*

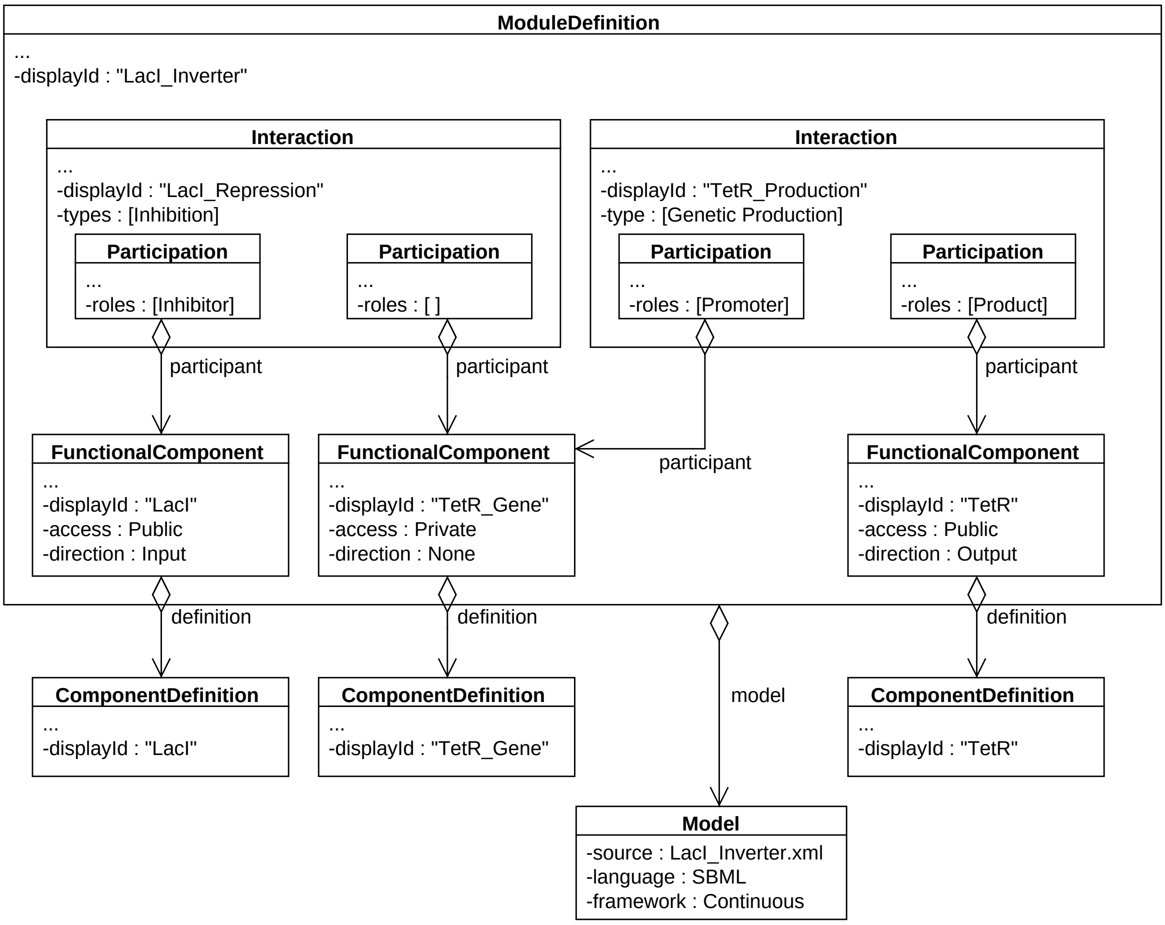

***Figure 33:*** *`ModuleDefinition` of the LacI inverter. This `ModuleDefinition` contains `FunctionalComponent` objects that instantiate the `ComponentDefinition` objects for the LacI/TetR transcription factors and TetR gene. These `FunctionalComponent` objects participate in a repression `Interaction` and a genetic production `Interaction`, thereby indicating which biological structures carry out the function of the LacI inverter `ModuleDefinition`. In this case, the transcription and translation of TetR are represented as a single genetic production `Interaction` that abstracts away the presence of the intermediate TetR mRNA. In addition, this `ModuleDefinition` is also associated with a continuous `Model` written in the SBML source file “LacI_Inverter.xml.”*

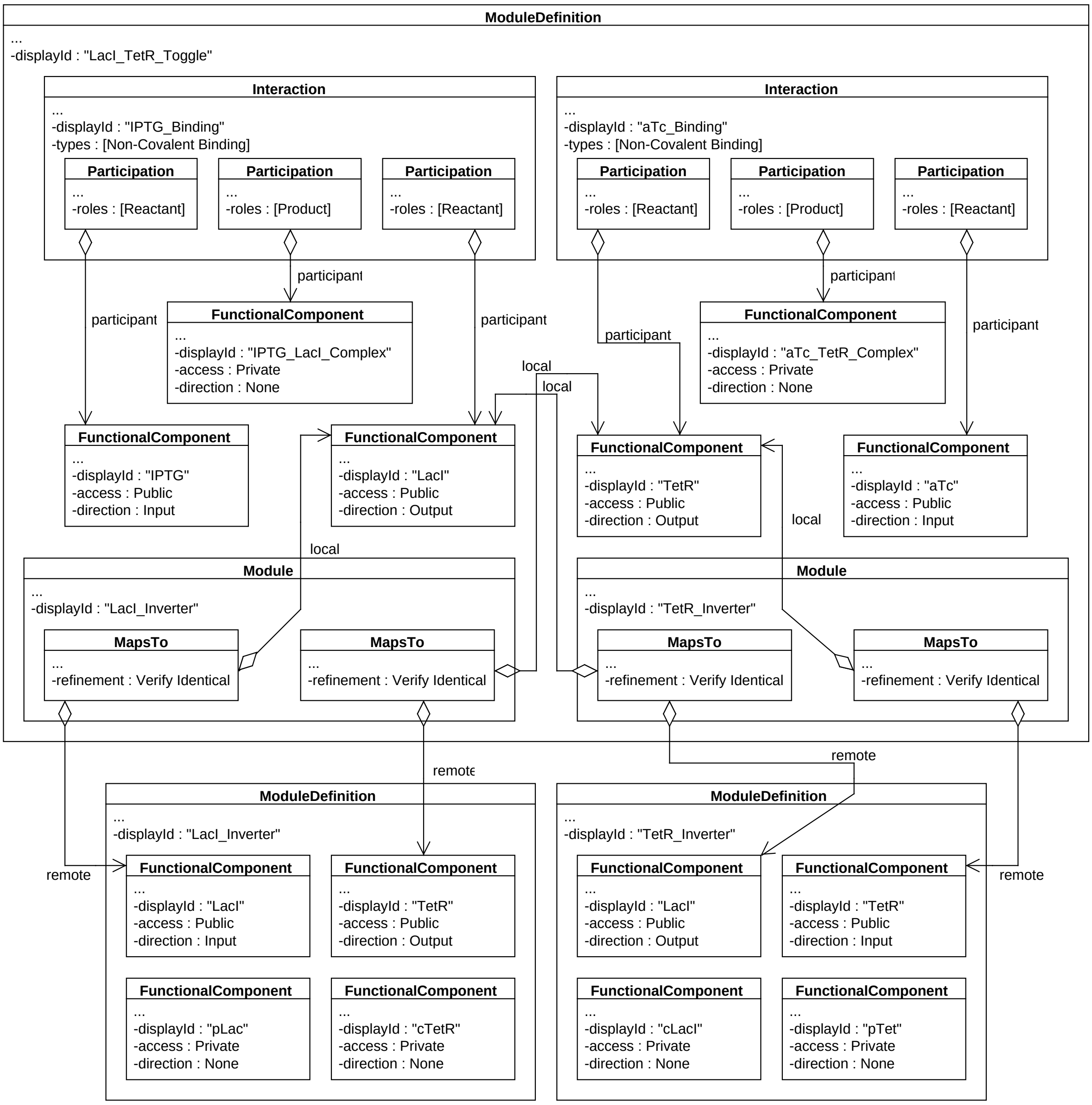

***Figure 34:*** *Composite `ModuleDefinition` of the LacI/TetR toggle switch. This `ModuleDefinition` contains the `Module` objects that instantiate LacI and TetR inverter `ModuleDefinition` objects. It also contains `FunctionalComponent` objects that instantiate the `ComponentDefinition` objects for the LacI/TetR transcription factors and IPTG/aTc small molecules. These `FunctionalComponent` objects each participate in a non-covalent binding `Interaction`. To complete the composition of the toggle switch, `MapsTo` objects are used to indicate that the output of the LacI inverter `ModuleDefinition` is identical to the input of the TetR inverter `ModuleDefinition` and vice versa.*

## 10 SBOL RDF Serialization

In order for SBOL objects to be readily stored and exchanged, it is important that they are able to be *serialized*, i.e., converted to a sequence of bytes that can be stored in a file or exchanged over a network. The serialization format for SBOL is designed to meet several competing requirements. First, SBOL needs to support ad-hoc annotations and extensions. Second, SBOL needs to support processing by general database and semantic web software tools that have little or no knowledge of the SBOL data model. Finally, it ought to be relatively simple to write a new software implementation, so that SBOL can be readily used even in software environments where community-maintained implementations are not available.

To meet these goals, the canonical serialization of SBOL has been selected to be a strict dialect of RDF/XML Beckett and McBride (2004), a syntactic standard defined for Semantic Web data exchange. This serialization provides a standard base from which to meet further requirements. Moreover, it allows any RDF/XML-aware software tool to consume and operate on an SBOL file without needing any customization to support SBOL. Where possible, we have re-used predicates from widely-used terminologies (such as Dublin Core DCMI Usage Board (2012)) to expose as much of the data as practical to such standard RDF tooling.

Arbitrary RDF/XML, however, provides a sometimes problematically large amount of flexibility in how equivalent data can be serialized. This flexibility can result in different serializations when processing RDF/XML files using standard off-the-shelf XML tools, such as DOM-OO mappings. To address this issue, we define a canonical association between the nesting of data structures within the SBOL UML data model and the RDF/XML file. For all ownership associations (filled diamonds), the RDF/XML for the owned entity is embedded within the owner's RDF/XML (note, however, that the property values MAY be listed in any order). For all associations that are by reference (open diamonds), the RDF/XML for the referenced property is linked via a resource `URI`. For example, the serialization of a `ComponentDefinition` embeds the serializations of the `SequenceConstraint` and `Component` objects associated with it. Those `SequenceConstraint` objects, however, link to the `Component` objects with a `URI` rather than embedding another copy.

Every SBOL document MUST be a valid RDF/XML document. Accordingly, each SBOL document starts with an XML declaration that has its XML version set to "1.0." As shown in the example below, this declaration is then followed by an `rdf:RDF` XML element that includes the namespace declarations for RDF, Dublin Core, PROV, and SBOL. The SBOL namespace, which is `http://sbols.org/v2#`, is used to indicate which entities and properties in the SBOL document are defined by SBOL, and MUST NOT be used for any entities or properties not defined in this specification.

```
<?xml version="1.0" ?>
<rdf:RDF xmlns:rdf="http://www.w3.org/1999/02/22-rdf-syntax-ns#" xmlns:dcterms="http://purl.org/dc/terms/" xmlns:prov="http://www.
      w3.org/ns/prov#" xmlns:sbol="http://sbols.org/v2#">
...
</rdf:RDF>
```

All first-class SBOL data types (i.e., those enumerated in Section 7) have an associated identifying `URI`. In the RDF, this is the resource `URI` used by instances of that type. For example, `ComponentDefinition` has the type `URI` `sbol:ComponentDefinition`. Properties and associations are then asserted as nested RDF/XML assertions. Section 7 provides the serialization template and an example at the end of its description of each data type. All of the data types that are `TopLevel` are so named because they always appear at the top-most level of the RDF/XML serialization. All other data types will always appear nested within their parent container and, ultimately, some `TopLevel` object. For example, a `ComponentDefinition` is a `TopLevel` and therefore listed at the top-most level of the RDF/XML serialization, and contains its `SequenceConstraint` objects, since they are not `TopLevel`. Its `Sequence`, however, is also `TopLevel` and is therefore not nested within and instead linked via a `URI`.

Each instance of a first-class SBOL data type MAY have annotations attached, as described in Section 7.16. These annotations are composed of a name and a value. They are serialized to RDF as a conceptual triple with the subject being the identity of the instance they annotate, the predicate being the name of the annotation, and the object being the value of that annotation. Annotation values are always nested within the RDF/XML serialization of the instance that they annotate. For example, a ModuleDefinition might add a DOI annotation that links to the scientific article that first described the system that it represents.

SBOL also supports top-level, user-defined data, again as described in Section 7.16. This is to allow non-standardized but necessary information to be carried around as part of a design. For example, a particular sub-community might have an internal standard for genetic device characterization data sheets. Such data can be represented as a GenericTopLevel object with internal structured annotations. For example, each individual data sheet might be contained in its own GenericTopLevel instance. This custom data is serialized into the RDF/XML in the usual way, as an RDF/XML block at the top level of the file. Other objects can refer to this entity through their annotations by reference, and this generic top-level entity can refer to other entities via references. For example, a ModuleDefinition might use an annotation to refer to the data sheet GenericTopLevel that documents its properties.

2.0.1 As previously stated, XML is the preferred RDF serialization for SBOL files, and these files should use the ".xml" file extension. While any RDF serialization is valid, as the contents of the triples and not their format determine the validity, restriction to XML enhances human ability to classify a document as SBOL easily. Furthermore, documents can be human-examined by using XML formatting tools and syntax highlighters. It should be noted that XML was chosen for no particular reason than the simple fact of its widespread use in the community and by existing libraries. The proposers are aware that many of these benefits apply to several serializations of SBOL. The choice of XML is simply a codification of the existent standard and practice.

By adopting this paradigm of RDF/XML serialization, SBOL is able to adapt to future changes in the standard without requiring large-scale alterations to the RDF files. Since exactly the same scheme is used to serialize annotations as is used to serialize specification-defined properties and associations, it is possible to update the SBOL standard to recognize a different range of properties and associations. Those properties not recognized by the specification will always be available through the API as annotations. Similarly, by allowing arbitrary top-level entities in a SBOL file, we enable future specifications or extensions to ratify the structure of other top-level objects. These entities would then become part of the explicit data model, but the identical RDF serialization would be used. Applications lacking support for a given extension can safely read in, manipulate, and write out the top-level data that is not understood, treating it as a top-level structured annotation, without data loss or corruption. Finally, the very regimented control of nesting versus referencing also allows the XML structure to be very predictable, enabling XML/DOM-based tooling to work with SBOL RDF/XML files safely.

## 11 SBOL Compliance

2.0.1 There are different types of software compliance with respect to the SBOL specification. First, a software tool can either support all classes of the SBOL 2.x data model or only its structural subset. The structural subset includes the following classes:

- `Sequence`
- `ComponentDefinition`
    - `Component`
    - `SequenceAnnotation`
    - `SequenceConstraint`
- `Collection`
- `Annotation`
- `GenericTopLevel`

Second, an SBOL-compliant software tool can support import of SBOL, export of SBOL, or both. If it supports both import and export, it can do so in either a lossy or lossless fashion.

In order to test import compliance, developers are encouraged to use the SBOL test files found here:
`https://github.com/SynBioDex/SBOLTestSuite`
Examples of every meaningful subset of objects are provided, including both structural-only SBOL (that is, annotated DNA sequence data) and complete tests.

In order to test export compliance, developers are encouraged to validate SBOL files generated by their software with the SBOL Validator found here:
`http://www.async.ece.utah.edu/sbol-validator/`
This validator can also be used to check lossless import/export support, since it can compare the data content of files imported and exported by a software tool.

Finally, developers of SBOL-compliant tools are encouraged to notify the SBOL editors (sbol-editors@googlegroups.com) when they have determined that their tool is SBOL compliant, so their tool can be publicly categorized as such on the SBOL website.

## 12 Recommended Best Practices

2.0.1

### 12.1 SBOL Namespaces

Namespaces for different versions of SBOL SHOULD NOT be semantically mixed in the same document. For example, an SBOL 2.x `ComponentInstance` SHOULD NOT refer to an SBOL 1.x DNAComponent. However, namespaces for different versions MAY be present in the same document so long as they are semantically independent (that is, so long as objects belonging to these namespaces do not refer to each other). When multiple SBOL namespaces are present in a document, libraries SHOULD default to interpreting as SBOL only those objects belonging to the namespace for the most recent version of SBOL in the document. Any remaining objects belonging to any other SBOL namespace SHOULD be interpreted as `GenericTopLevels` and custom `Annotations`.

### 12.2 Use of the Version Property

Once an SBOL object has been published where others might have access it (e.g., to an online repository), it might be the case that other objects come to depend on the particular contents of the published object. Thus, in order to avoid creating conflicting data, if a person wants to change the properties of a published object, they SHOULD do so by making a new copy of object that incorporates the change and has an `identity` property that contains a new `URI`.

The relationship between the old and new objects (i.e., that the new object was derived from the old object), however, is not visible unless it is explicitly declared. This is RECOMMENDED to be done using the `persistentIdentity`, and `version` properties. The preferred practice for declaring such a relationship is to use the same `persistentIdentity` for both objects, but give the newer object a later `version`. Then, when the new object is published, it can be clear to both humans and machines that this object is intended to update the previously published object. In this way, when a user wants the latest version of an object, they can obtain it by referencing the object via its `persistentIdentity` and rely on a tool to find the object with that `persistentIdentity` and the latest `version`.

As stated in Section 7.4, it is RECOMMENDED that version numbering follow the conventions of semantic versions (`http://semver.org/`), particularly as implemented by Maven (`http://maven.apache.org/`). This convention represents versions as sequences of numbers and qualifiers separated by the characters `.` and `-` and compared in lexicographical order (for example, 1 < 1.3.1 < 2.0-beta). For a full explanation, see the linked resources.

### 12.3 Compliant SBOL Objects

Maintaining unique `identity` `URI`s for all SBOL objects can be a very challenging implementation task. To reduce this burden, users of SBOL 2.x are encouraged to follow a few simple rules when constructing the `identity` properties and related properties for SBOL objects. When these rules are followed in constructing an SBOL object, we say that this object is *compliant*. These rules are as follows:

1. The `identity` of a compliant SBOL object MUST begin with a *URI prefix* that maps to a domain over which the user has control. Namely, the user can guarantee uniqueness of identities within this domain.
2. The `persistentIdentity` and `displayId` properties are REQUIRED of a compliant SBOL object.
3. The `persistentIdentity` of a compliant `TopLevel` object MUST end with a delimiter ('/', '#', or ':') followed by the `displayId` of the object.
4. The `persistentIdentity` of a compliant SBOL object that is not also a `TopLevel` object MUST begin with the `persistentIdentity` of its parent object and be immediately followed by a delimiter ('/', '#', or ':') and the `displayId` of the compliant object.
5. If a compliant SBOL object is not given a `version`, then its `identity` and `persistentIdentity` properties MUST contain the same `URI`.

6. If a compliant SBOL object has a version, then its identity property MUST contain a URI of the form “⟨persistentIdentity⟩/⟨version⟩”.
7. The version of a compliant SBOL object that is not also a TopLevel object MUST contain the same String as the version property of the compliant object’s parent object.
8. The identity, persistentIdentity, displayId, and version properties of a compliant SBOL object MUST NOT be changed once set.

All examples in this specification use compliant URIs.

### 12.4 Annotations: Embedded Objects vs. External References

When annotating an SBOL document with additional information, there are two general methods that can be used:

- Embed the information in the SBOL document, either as non-SBOL properties or GenericTopLevel objects.
- Store the information separately and annotate the SBOL document with URIs that point to it.

In theory, either method can be used in any case. (Note that a third case not discussed here is to use SBOL to annotate external objects with linking to SBOL documents, rather than annotate SBOL documents with links external objects.)

In practice, embedding massive amounts of non-SBOL data into SBOL documents is likely to cause problems for people and software tools trying to manage and exchange such documents. Therefore, it is RECOMMENDED that small amounts of information (e.g., design notes or preferred graphical layout) be embedded in the SBOL model, while large amounts of information (e.g., the contents of the scientific publication from which a model was derived or flow cytometry data that characterizes performance) be linked with URIs pointing to external resources. The boundary between “small” and “large” is left deliberately vague, recognizing that it will likely depend on the particulars of a given SBOL application.

### 12.5 Completeness and Validation

RDF documents containing serialized SBOL objects might or might not be entirely self-contained. A SBOL document is self-contained or “complete” if every SBOL object referred to in the document is contained in the document. It is RECOMMENDED that serializations be complete whenever practical. In order words, when serializing an SBOL object, serialize all of the other objects that it points to, then serialize all of the other objects that these objects point to, etc., until the document is complete.

It is important to note that there is no guarantee that an RDF document contains valid SBOL. When an RDF document is deserialized to SBOL objects, the program doing so SHOULD verify that all of the property values encoded therein have the right data type (e.g., that the object pointed to by the sequences property of a ComponentDefinition is really a Sequence). For complete files, this validation can be carried out entirely locally. For files that are not complete, an implementation either needs to have a means of validating those external references (e.g., by retrieving them from various repositories), or it needs to mark them as unverified and not depend on their correctness.

### 12.6 Recommended Ontologies for External Terms

External ontologies and controlled vocabularies are an integral part of SBOL. SBOL uses URIs to access existing biological information through these resources. New SBOL specific terms are defined only when necessary. For example, ComponentDefinition types, such as DNA or protein, are described using BioPAX terms. Similarly, the roles of a DNA or RNA ComponentDefinition are described via SO terms. Although RECOMMENDED ontologies
2.0.1 have been indicated in relevant sections where possible, other resources providing similar terms can also be used. A summary of these external sources can be found in Table 17.

2.1.0

| SBOL Entity | Property | Preferred External Resource | More Information |
|---|---|---|---|
| **ComponentDefinition** | types | BioPAX | http://www.biopax.org |
| | types | SO (nucleic acid topology) | http://www.sequenceontology.org |
| | roles | SO (*DNA* or *RNA*) | http://www.sequenceontology.org |
| | roles | CHEBI (*small molecule*) | https://www.ebi.ac.uk/chebi/ |
| **Interaction** | types | SBO (occurring entity branch) | http://www.ebi.ac.uk/sbo/main/ |
| **Participation** | roles | SBO (participant roles branch) | http://www.ebi.ac.uk/sbo/main/ |
| **Model** | language | EDAM | http://bioportal.bioontology.org/ontologies/EDAM |
| | framework | SBO (modeling framework branch) | http://www.ebi.ac.uk/sbo/main/ |

***Table 17:*** *Preferred external resources from which to draw values for various SBOL properties.*

The URIs for ontological terms SHOULD come from identifiers.org. However, it is acceptable to use terms from purl.org as an alternative, for example when RDF tooling requires URIs to be represented as compliant QNames. SBOL software may convert between these forms as required.

2.0.1

### 12.7 Annotating Entities with Date & Time

Entities in an SBOL document can be annotated with creation and modification date. It is RECOMMENDED that predicates, or properties, from DCMI Metadata Terms SHOULD be used to include date and time information. The `created` and `modified` terms SHOULD respectively be used to annotate SBOL entities with creation and modification dates. Date and time values SHOULD be expressed using the XML Schema `DateTime` datatype (Biron et al., 2004). For example, "`2016-03-16T20:12:00Z`" specifies that the day is 16 March 2016 and the time is 20:12pm in UTC (Coordinated Universal Time). An example serialization is shown below:

```
<?xml version="1.0" ?>
<rdf:RDF xmlns:rdf="http://www.w3.org/1999/02/22-rdf-syntax-ns#" xmlns:dcterms="http://purl.org/dc/terms/" xmlns:prov="http://www.
      w3.org/ns/prov#" xmlns:sbol="http://sbols.org/v2#">
  <sbol:CLASS_NAME rdf:about="...">
    <dcterms:created>DATE_TIME_STRING</dcterms:created>
    <dcterms:modified>DATE_TIME_STRING</dcterms:modified>
             ...
  </sbol:CLASS_NAME>
  ...
</rdf:RDF>
```

2.2.1

### 12.8 Annotating Entities with Authorship information

Authorship information should ideally be added to `TopLevel` entities, where possible. It is RECOMMENDED that the `creator` DCMI Metadata term SHOULD be used to annotate SBOL entities with authorship information. This property can be repeated for each author. The example below shows the use of this property for two authors and the values shown are free text `String` literals.

```
<?xml version="1.0" ?>
<rdf:RDF xmlns:rdf="http://www.w3.org/1999/02/22-rdf-syntax-ns#" xmlns:dcterms="http://purl.org/dc/terms/" xmlns:prov="http://www.
      w3.org/ns/prov#" xmlns:sbol="http://sbols.org/v2#">
  <sbol:CLASS_NAME rdf:about="...">
    <dcterms:creator>First author</dcterms:creator>
    <dcterms:creator>Second author</dcterms:creator>
             ...
  </sbol:CLASS_NAME>
  ...
</rdf:RDF>
```

## 13 Complementary Standards

2.1.0 
## 13.1 Adding Provenance with PROV-O

[New in 2.1.0; see SEP 009: https://github.com/SynBioDex/SEPs/blob/master/sep_009.md]

Provenance is central to a range of quality control and attribution tasks within the Synthetic Biology design process. Tracking attribution and derivation of one resource from another is paramount for managing intellectual property purposes. Source designs are often modified in systematic ways to generate derived designs, for example, by applying codon optimization or systematically removing all of a class of restriction enzyme sites. Documenting the transformation used, and any associated parameters, makes this explicit and potentially allows the process to be reproduced systematically. If a design has been used within other designs, and is later found to be defective, it is paramount that all uses of it, including uses of edited versions of the design, can be identified, and ideally replaced with a non-defective alternative. When importing data from external sources, it is important not only to attribute the original source (for example, GenBank), but also the tool used to perform the import, as this may have made arbitrary choices as to how to represent the source knowledge as SBOL. All these activities have in common that it is necessary to track what resource, and what transformation process was applied by whom to derive an SBOL design.

The PROV-O ontology (https://www.w3.org/TR/prov-o/) already defines a data model for provenance. PROV-O's `wasDerivedFroms` property was adopted in SBOL 2.x to describe the derivation of an SBOL entity from another in a light-weight provenance scheme. Here, a subset of PROV-O is adopted for SBOL as a best practice to describe these activities in more detail[1]. PROV-O defines three core classes: `Entity`, `Activity` and `Agent`. An Agent (for example a software or a person) runs an Activity (according to a Plan) to generate one Entity from another. Although, PROV-O provides many other classes and rich set of terms. this specification describes the minimal subset of PROV-O that a provenance-aware SBOL tool should handle. This subset (Figure 35) includes the PROV-O's `Activity`, `Usage`, `Association`, `Agent` and `Plan` classes. Any SBOL's `Identified` object implicitly can act as an instance of PROV-O's `Entity` class and can include provenance through relationships to different activities. Resources representing `Agent`, `Activity` and `Plan` classes should be handled as `GenericTopLevel`, whilst `Usage` and `Association` resources should be nested within their parent activities.

Providers of provenance information are free to make use of more of PROV-O than is described here. It is acceptable for tools that understand more than this subset to use as much as they are able. Tools that only understand this subset must treat any additional data as annotations. Tools that are not aware of SBOL provenance at all must maintain and provide access to this information as annotations. This specification does not state what the newly added properties must point to. As long as they are resources that are consistent with the PROV-O property domains, they are legal. For example, a ComponentDefinition may be derived from another ComponentDefinition, but it would probably not make sense for it to be derived from a Collection.

2.2.0 The PROV-O specification permits that any kind of SBOL object may be used to generate another. The meaning of these relationships are specified using ontology terms for the `roles` properties on `Usage` and `Association` classes. This specification gives users the flexibility to construct and track provenance histories for their own custom applications, but this flexibility also presents an obstacle to standardized data exchange. Therefore a simple ontology (see Table 20 and Table 19) has been adopted to describe common provenance connections expected in synthetic biology workflows, based on the design-build-test-learn formalization of engineering.

The design-build-test-learn cycle is a common theme in synthetic biology and engineering literature. The design-build-test-learn cycle is the scientific method applied to engineering. Stages of the cycle include designing an initial prototype, testing that prototype, analyzing its performance against specific metrics, learning what worked and what did not work, designing a new prototype based on what was learned, and completing the cycle again. Ideally each iteration of the cycle generates new understanding that feeds back into new cycles as alternative approaches or reformulated problems. Therefore, the design-build-test-learn cycle is a *de facto* ontology upon which to base an SBOL data model for workflow abstraction. Other workflow activities in synthetic biology, such as analyzing, modeling, verifying, and evolving, by and large should fit into this design-build-test-learn abstraction.

[1] We thank Dr Paolo Missier from the School of Computing Science, Newcastle University for discussions regarding the use of PROV-O.

It is expected that users will develop their own ontology terms to specify how SBOL objects are used in a recipe, protocol, or computational analysis. However, these home-made ontologies will be very domain specific, and may not be intelligible to users working in another domain. For example a modeler should not be expected to understand an ontology of `Usage` `roles` for DNA assembly. The terms "design", "build", "test", and "learn" provide a high level workflow abstraction that allows tool-builders to quickly search for and isolate provenance histories relevant to their domain, while keeping track of the flow of data between different users working in different domains of synthetic biology. An example of how these terms are used is provided in Figure 36.

Provenance semantics defined through `wasGeneratedBys` relationships are distinctly different from versioning semantics. Generation of a new object is defined by the W3C PROV-O specification as follows:

> ...the completion of production of a new entity by an activity. This entity did not exist before generation and becomes available for usage after this generation.

These semantics are somewhat different from the versioning semantics defined in section Section 7.4. The SBOL specification defines a new version of an object as an update of a previously published object (and therefore a previously existing object). Therefore, an SBOL object which is "generated" from another SHOULD BE regarded as a new entity rather than a new version of an existing entity. However, this distinction is somewhat subjective (see Theseus's paradox). Therefore, we RECOMMEND as a best practice that objects linked by Activities not be successive versions of each other, though this is left to the discretion of users and library developers.

2.2.0

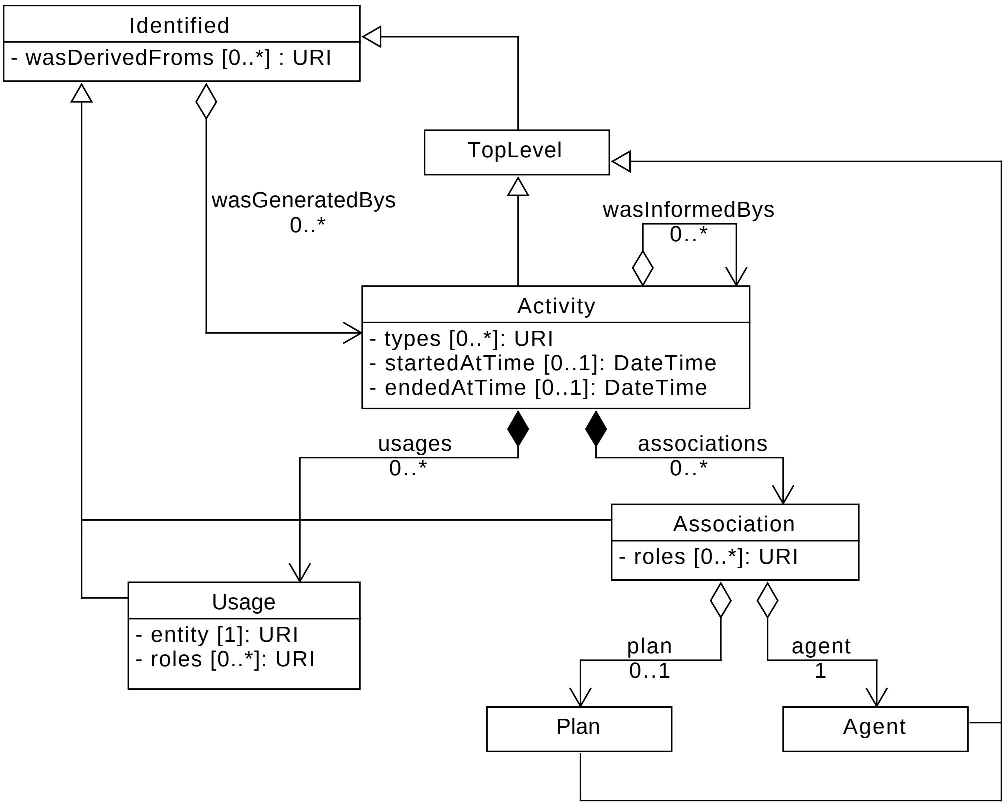

***Figure 35:*** *Relationships between SBOL and PROV-O classes. The PROV-O classes* **`Activity`**, **`Plan`**, *and* **`Agent`** *are all serialized as* `TopLevel` *classes in an SBOL document.*

2.1.0

#### 13.1.1 Activity

A generated `Entity` is linked through a `wasGeneratedBy` relationship to an `Activity`, which is used to describe how different `Agents` and other entities were used. An `Activity` is linked through a `associations` to `Associations`, to describe the role of agents, and is linked through `usages` to `Usages` to describe the role of other entities used as part of the activity. Moreover, each `Activity` includes optional startedAtTime and endedAtTime properties. When using `Activity` to capture how an entity was derived, it is expected that any additional information needed will be attached as annotations. This may include software settings or textual notes. Activities can also be linked together using the `wasInformedBys` relationship to provide dependency without explicitly specifying start and end times.

2.3.0

##### *The `types` property*

The `types` property is an OPTIONAL set of `URI`s that explicitly specify the type of the provenance `Activity` in more detail. If specified, it is RECOMMENDED that at least one `URI` of the `types` property of an `Activity` refers to a `URI` from Table 18.

| Activity Type | URI |
|---|---|
| Design | `http://sbols.org/v2#design` |
| Build | `http://sbols.org/v2#build` |
| Test | `http://sbols.org/v2#test` |
| Learn | `http://sbols.org/v2#learn` |

***Table 18:*** *URIs to specify the `types` property of an `Activity`.*

##### *The `startedAtTime` property*

The `startedAtTime` property is OPTIONAL and contains a DateTime (see section Section 12.7) value, indicating when the activity started. If this property is present, then the `endedAtTime` property is REQUIRED.

##### *The `endedAtTime` property*

The `endedAtTime` property is OPTIONAL and contains a DateTime (see section Section 12.7) value, indicating when the activity ended.

##### *The `associations` property*

The `associations` property is OPTIONAL and MAY contain a set of `URI`s that refers to `Association` objects.

##### *The `usages` property*

The `usages` property is OPTIONAL and MAY contain a set of `URI`s that refers to `Usage` objects.

##### *The `wasInformedBys` property*

The `wasInformedBys` property is OPTIONAL and MAY contain a set of `URI`s that refers to other `Activity` objects.

2.3.0

##### *Serialization*

The serialization of an `Activity` MUST have the following form:

```
<prov:Activity rdf:about="...">
  ... properties inherited from identified ...
  zero or more  <prov:qualifiedAssociation>
                   <prov:Association rdf:about="...">...</prov:Association>
                </prov:qualifiedAssociation> elements
  zero or more  <prov:qualifiedUsage>
                   <prov:Usage rdf:about="...">...</prov:Usage>
```

```
              </prov:qualifiedUsage> elements
  zero or more  <sbol:type rdf:resource="... "/>  elements
  zero or one  <prov:startedAtTime>...</prov:startedAtTime> element
  zero or one  <prov:endedAtTime>...</prov:startedAtTime> element
  zero or more  <prov:wasInformedBy rdf:resource="..."/> element
</prov:Activity>
```

2.2.0 Note that the tags prov:qualifiedUsage and prov:qualifiedAssociation are used for usages and associations, respectively.

2.1.0

#### 13.1.2 Usage

How different entities are used in an Activity is specified with the Usage class, which is linked from an Activity through the Usage relationship. A Usage is then linked to an Entity through the Entity's URI and the role of this entity is qualified with the roles property. When the wasDerivedFroms property is used together with the full provenance described here, the entity pointed at by the wasDerivedFroms property MUST be included in a Usage.

##### The entity property

The entity property is REQUIRED and MUST contain a URI which MAY refer to an SBOL Identified object.

##### The roles property

2.2.0 The roles property is an OPTIONAL set of URIs that refer to particular term(s) describing the usage of an entity referenced by the entity property. Recommended terms that are defined in Table 19 can be used to indicate how the referenced entity is being used in this Activity.

2.2.0

| URI for Usage roles | Description |
|---|---|
| http://sbols.org/v2#design | Design describes the process by which a conceptual representation of an engineer's imagined and intended design for a biological system is derived, possibly from a predictive model or by modifying a pre-existing design. In the context of a Usage, the term indicates that the referenced entity was generated by some previous design Activity and was used by the present Activity as a design for a new object. |
| http://sbols.org/v2#build | Build describes the process by which a biological construct, sample, or clone is implemented in the laboratory. In the context of a Usage, the term indicates that the referenced entity was generated by some previous build Activity and was used by the present Activity as a built object. |
| http://sbols.org/v2#test | Test describes the process of performing experimental measurements to characterize a synthetic biological construct. In the context of a Usage, the term indicates that the referenced entity was generated by some previous test Activity and is used as test data in the present Activity. |
| http://sbols.org/v2#learn | Learn describes the process of analyzing experimental measurements to produce a new entity that represents biological knowledge. In the context of a Usage, the term indicates that the referenced entity was generated by some previous learn Activity and is used in the present Activity as a source of scientifically verified knowledge. |

***Table 19:*** *Terms to specify the roles property of a Usage.*

2.1.0

#### 13.1.3 Association

An Association is linked to an Agent through the agent relationship. The Association includes the roles property to qualify the role of the Agent in the Activity.

##### *The `agent` property*

The `agent` property is REQUIRED and MUST contain a `URI` that refers to an `Agent` object.

##### *The `roles` property*

2.2.0 The `roles` property is an OPTIONAL set of `URI`s that refers to particular term(s) that describes the the role of the `agent` in the parent `Activity`. The recommended terms that are defined in Table 20 can be used to specify the kind of `Activity` performed by the `Agent`.

2.3.0

| URI for Association roles | Description |
|---|---|
| `http://sbols.org/v2#design` | Design describes the process by which a conceptual representation of an engineer's imagined and intended design for a biological system is derived, possibly from a predictive model or by modifying a pre-existing design. In the context of an `Association`, the term design indicates that the `Agent` performed the parent `Activity` to generate a design. |
| `http://sbols.org/v2#build` | Build describes the process by which a biological construct, sample, or clone is implemented in the laboratory. In the context of an `Association`, the term build indicates that the `Agent` performed the parent `Activity` to implement a design. More generally, the term may represent any kind of experimental manipulation of a biological sample, including propagating, passaging, or evolving cell lines. |
| `http://sbols.org/v2#test` | Test describes the process of performing experimental measurements to characterize a synthetic biological construct. In the context of an `Association`, the `Agent` performed the parent `Activity` to perform experimental measurements resulting in raw data represented by an `ExperimentalData`. |
| `http://sbols.org/v2#learn` | Learn describes the process of analyzing the experimental measurements in order to produce a new entity that represents biological knowledge. In the context of an `Association`, the `Agent` processed the raw experimental data to produce an analysis. This process generates a new entity that represents biological knowledge, including tables or graphs referenced by the `Attachment`s of an `ExperimentalData`, a `Model` produced by a fitting process, a consensus `Sequence` derived from sequencing results, etc. |

***Table 20:*** *Terms to specify the `roles` property of an `Association`.*

2.1.0

##### *The `plan` property*

The `plan` property is OPTIONAL and contains a `URI` that refers to a `Plan`.

##### *Serialization*

The serialization of an `Association` MUST have the following form:

```
<prov:Association rdf:about="...">
  ... properties inherited from identified ...
  zero or more  <prov:hadRole rdf:resource="..."/> element
  zero or one  <prov:hadPlan rdf:resource="..."/> element
  one  <prov:agent rdf:resource="..."/> element
 </prov:Association>
```

2.2.0 Note that the tags prov:hadRole and prov:hadPlan are used for `roles` and `plan`, respectively.

#### 13.1.4 Plan

2.1.0 The Plan entity can be used as a place holder to describe the steps (for example scripts or lab protocols) taken when an `Agent` is used in a particular `Activity`.

##### Serialization

The serialization of an `Usage` MUST have the following form:

```
<prov:Plan rdf:about="...">
  ... properties inherited from identified ...
  ... other annotations ...
 </prov:Plan>
```

#### 13.1.5 Agent

2.1.0 Examples of agents are person, organization or software. These agents should be annotated with additional information, such as software version, needed to be able to run the same software again.

##### Serialization

The serialization of an `Agent` MUST have the following form:

```
<prov:Agent rdf:about="...">
  ... properties inherited from identified ...
  ... other annotations ...
 </prov:Agent>
```

##### Example - Codon optimization

2.1.0 Codon optimization is a practical real-wold example where provenance properties can be applied. Using the current specification, the relationship between an original CDS and the codon-optimized version could simply be represented using the prov:wasDerivedFrom predicate, in a light-weight form. With more comprehensive use of the PROV ontology, the codon optimization can be represented as an `Activity`. This `Activity` can then include additional information, such as the `Agent` responsible (in this case, codon-optimizing software), and additional parameters.

```
<?xml version="1.0" ?>
<rdf:RDF xmlns:prov="http://www.w3.org/ns/prov#" xmlns:rdf="http://www.w3.org/1999/02/22-rdf-syntax-ns#" xmlns:myapp="http://
      myapp.com/" xmlns:sbol="http://sbols.org/v2#" xmlns:dcterms="http://purl.org/dc/terms/">
  <sbol:ComponentDefinition rdf:about="http://myapp.com/codon_optimized">
    <sbol:persistentIdentity rdf:resource="http://myapp.com/codon_optimized"/>
    <sbol:displayId>codon_optimized</sbol:displayId>
    <prov:wasDerivedFrom rdf:resource="http://myapp.com/non_codon_optimized"/>
    <prov:wasGeneratedBy rdf:resource="http://myapp.com/codon_optimization_activity"/>
    <dcterms:title>Codon optimized CDS</dcterms:title>
    <sbol:type rdf:resource="http://www.biopax.org/release/biopax-level3.owl#DnaRegion"/>
    <sbol:role rdf:resource="http://identifiers.org/so/SO:0000316"/>
  </sbol:ComponentDefinition>
  <sbol:ComponentDefinition rdf:about="http://myapp.com/non_codon_optimized">
    <sbol:persistentIdentity rdf:resource="http://myapp.com/non_codon_optimized"/>
    <sbol:displayId>non_codon_optimized</sbol:displayId>
    <dcterms:title>Non Codon optimized CDS</dcterms:title>
    <sbol:type rdf:resource="http://www.biopax.org/release/biopax-level3.owl#DnaRegion"/>
    <sbol:role rdf:resource="http://identifiers.org/so/SO:0000316"/>
  </sbol:ComponentDefinition>
  <prov:Activity rdf:about="http://myapp.com/codon_optimization_activity">
    <sbol:persistentIdentity rdf:resource="http://myapp.com/codon_optimization_activity"/>
    <sbol:displayId>codon_optimization_activity</sbol:displayId>
    <dcterms:title>Codon Optimization Activity</dcterms:title>
    <prov:qualifiedAssociation>
      <prov:Association rdf:about="http://myapp.com/codon_optimization_activity/association">
```

```
        <sbol:persistentIdentity rdf:resource="http://myapp.com/codon_optimization_activity/association"/>
        <sbol:displayId>association</sbol:displayId>
        <prov:hadRole rdf:resource="http://myapp.com/codonoptimizer"/>
        <prov:agent rdf:resource="http://myapp.com/codon_optimization_software"/>
      </prov:Association>
    </prov:qualifiedAssociation>
    <prov:qualifiedUsage>
      <prov:Usage rdf:about="http://myapp.com/codon_optimization_activity/usage">
        <sbol:persistentIdentity rdf:resource="http://myapp.com/codon_optimization_activity/usage"/>
        <sbol:displayId>usage</sbol:displayId>
        <prov:hadRole rdf:resource="http://sbols.org/v2#source"/>
        <prov:entity rdf:resource="http://myapp.com/non_codon_optimized"/>
      </prov:Usage>
    </prov:qualifiedUsage>
  </prov:Activity>
  <prov:Agent rdf:about="http://myapp.com/codon_optimization_software">
    <sbol:persistentIdentity rdf:resource="http://myapp.com/codon_optimization_software"/>
    <sbol:displayId>codon_optimization_software</sbol:displayId>
    <dcterms:title>Codon Optimization Software</dcterms:title>
  </prov:Agent>
</rdf:RDF>
```

#### *Example - Deriving strains*

2.1.0 Bacterial strains are often derived from other strains through modifications such as gene knockouts or mutations. For example, the `Bacillus subtilis` 168 strain was derived from the NCIMB3610 strain in the 1940s through x-radiation. *B. subtilis* 168 is a laboratory strain and has several advantages as a model organism in synthetic biology. Particularly, the 168 strain is easy to transform and is not motile, facilitating the analysis of engineered cells. The parent strain, on the other hand, is motile but more difficult to transform. The example below shows the derivation of the 168 strain using the new provenance classes.

```
<?xml version="1.0" ?>
<rdf:RDF xmlns:prov="http://www.w3.org/ns/prov#" xmlns:rdf="http://www.w3.org/1999/02/22-rdf-syntax-ns#" xmlns:myapp="http://
      myapp.com/" xmlns:sbol="http://sbols.org/v2#" xmlns:dcterms="http://purl.org/dc/terms/">
  <sbol:ComponentDefinition rdf:about="http://myapp.com/bsubtilisncimb3610">
    <sbol:persistentIdentity rdf:resource="http://myapp.com/bsubtilisncimb3610"/>
    <sbol:displayId>bsubtilisncimb3610</sbol:displayId>
    <dcterms:title>Bacillus subtilis NCIMB3610</dcterms:title>
    <sbol:type rdf:resource="http://www.biopax.org/release/biopax-level3.owl#DnaRegion"/>
    <sbol:role rdf:resource="http://identifiers.org/so/SO:0000316"/>
  </sbol:ComponentDefinition>
  <sbol:ComponentDefinition rdf:about="http://myapp.com/bsubtilis168">
    <sbol:persistentIdentity rdf:resource="http://myapp.com/bsubtilis168"/>
    <sbol:displayId>bsubtilis168</sbol:displayId>
    <prov:wasDerivedFrom rdf:resource="http://myapp.com/bsubtilisncimb3610"/>
    <prov:wasGeneratedBy rdf:resource="http://myapp.com/xraymutagenesis"/>
    <dcterms:title>Bacillus subtilis 168</dcterms:title>
    <sbol:type rdf:resource="http://www.biopax.org/release/biopax-level3.owl#DnaRegion"/>
    <sbol:role rdf:resource="http://identifiers.org/so/SO:0000316"/>
  </sbol:ComponentDefinition>
  <prov:Activity rdf:about="http://myapp.com/xraymutagenesis">
    <sbol:persistentIdentity rdf:resource="http://myapp.com/xraymutagenesis"/>
    <sbol:displayId>xraymutagenesis</sbol:displayId>
    <dcterms:title>X-ray mutagenesis</dcterms:title>
    <prov:qualifiedAssociation>
      <prov:Association rdf:about="http://myapp.com/xraymutagenesis/association">
        <sbol:persistentIdentity rdf:resource="http://myapp.com/xraymutagenesis/association"/>
        <sbol:displayId>association</sbol:displayId>
        <prov:hadRole rdf:resource="http://myapp.com/mutagen"/>
        <prov:agent rdf:resource="http://myapp.com/x_ray"/>
      </prov:Association>
    </prov:qualifiedAssociation>
    <prov:qualifiedUsage>
      <prov:Usage rdf:about="http://myapp.com/xraymutagenesis/usage">
        <sbol:persistentIdentity rdf:resource="http://myapp.com/xraymutagenesis/usage"/>
```

```
        <sbol:displayId>usage</sbol:displayId>
        <prov:hadRole rdf:resource="http://sbols.org/v2#source"/>
        <prov:entity rdf:resource="http://myapp.com/bsubtilisncimb3610"/>
      </prov:Usage>
    </prov:qualifiedUsage>
  </prov:Activity>
  <prov:Agent rdf:about="http://myapp.com/x_ray">
    <sbol:persistentIdentity rdf:resource="http://myapp.com/x_ray"/>
    <sbol:displayId>x_ray</sbol:displayId>
    <dcterms:title>X-ray</dcterms:title>
  </prov:Agent>
</rdf:RDF>
```

***Example - Design-build-test-learn Workflow***

2.3.0 This particular example represents an idealized workflow for model-based design. The workflow begins with a Model which describes the hypothesized behavior of a biological device. Using a computational tool, a new Design (ModuleDefinition) is composed of biological parts which links back to its Model. A genetic construct is then produced in the laboratory via an assembly protocol, and this biological sample is represented by a Build (Implementation). Once constructed, the Build is then characterized in the laboratory using an automated measurement protocol on a Tecan plate reader, thus generating Test data (represented by an ExperimentalData). Finally, a new Model is derived from these data using some a fitting algorithm implemented in the Python programming language. The final Model may not match the beginning Model, as the observed behavior may not match the prediction. This example illustrates one complete iteration through a design-build-test-learn cycle, as shown in Figure 36.

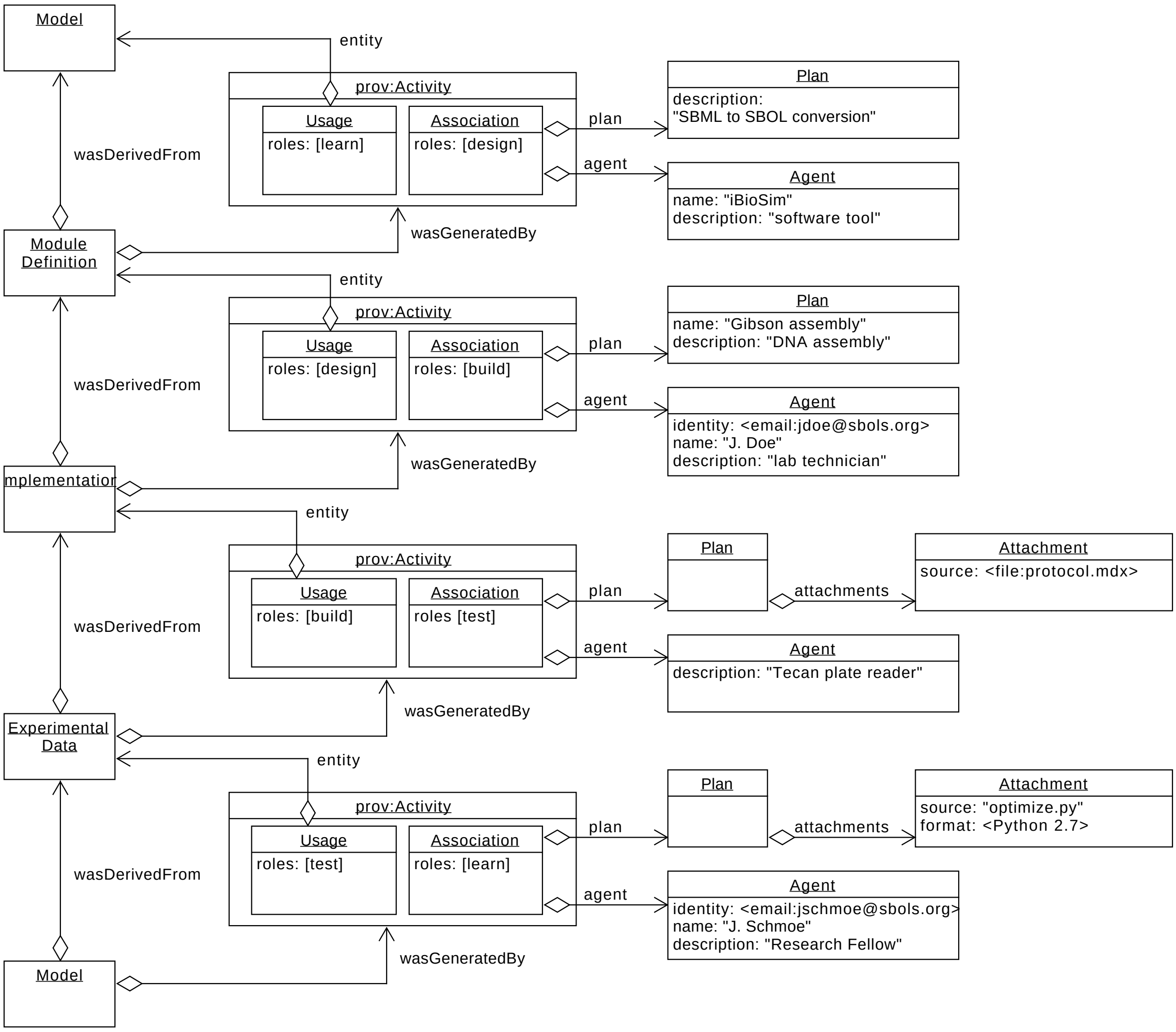

***Figure 36:*** *An example data structure representing an idealized workflow for model-based design*

2.2.1

```
<?xml version="1.0" encoding="utf-8"?>
<rdf:RDF xmlns:dcterms="http://purl.org/dc/terms/" xmlns:prov="http://www.w3.org/ns/prov#" xmlns:rdf="http://www.w3.org
      /1999/02/22-rdf-syntax-ns#" xmlns:sbol="http://sbols.org/v2#" xmlns:sys-bio="http://sys-bio.org#">
  <prov:Activity rdf:about="http://sys-bio.org/Activity_build_generation/1.0.0">
    <sbol:displayId>Activity_build_generation</sbol:displayId>
    <sbol:persistentIdentity rdf:resource="http://sys-bio.org/Activity_build_generation"/>
    <sbol:version>1.0.0</sbol:version>
    <prov:qualifiedAssociation>
      <prov:Association rdf:about="http://sys-bio.org/Activity_build_generation/build_generation_association/1.0.0">
        <sbol:displayId>build_generation_association</sbol:displayId>
        <sbol:persistentIdentity rdf:resource="http://sys-bio.org/Activity_build_generation/build_generation_association"/>
        <sbol:version>1.0.0</sbol:version>
        <prov:agent rdf:resource="https://synbiohub.org/jdoe/1.0.0"/>
        <prov:hadPlan rdf:resource="http://sys-bio.org/Plan_gibson_assembly/1.0.0"/>
        <prov:hadRole rdf:resource="http://sbols.org/v2#build"/>
      </prov:Association>
    </prov:qualifiedAssociation>
    <prov:qualifiedUsage>
      <prov:Usage rdf:about="http://sys-bio.org/Activity_build_generation/design_usage/1.0.0">
        <sbol:displayId>design_usage</sbol:displayId>
        <sbol:persistentIdentity rdf:resource="http://sys-bio.org/Activity_build_generation/design_usage"/>
        <sbol:version>1.0.0</sbol:version>
        <prov:entity rdf:resource="http://sys-bio.org/ModuleDefinition_design/1.0.0"/>
        <prov:hadRole rdf:resource="http://sbols.org/v2#design"/>
      </prov:Usage>
    </prov:qualifiedUsage>
  </prov:Activity>
  <prov:Activity rdf:about="http://sys-bio.org/Activity_design_generation/1.0.0">
    <sbol:displayId>Activity_design_generation</sbol:displayId>
    <sbol:persistentIdentity rdf:resource="http://sys-bio.org/Activity_design_generation"/>
    <sbol:version>1.0.0</sbol:version>
    <prov:qualifiedAssociation>
      <prov:Association rdf:about="http://sys-bio.org/Activity_design_generation/design_generation_association/1.0.0">
        <sbol:displayId>design_generation_association</sbol:displayId>
        <sbol:persistentIdentity rdf:resource="http://sys-bio.org/Activity_design_generation/design_generation_association"/>
        <sbol:version>1.0.0</sbol:version>
        <prov:agent rdf:resource="http://www.async.ece.utah.edu/ibiosim"/>
        <prov:hadPlan rdf:resource="http://sys-bio.org/Plan_conversion/1.0.0"/>
        <prov:hadRole rdf:resource="http://sbols.org/v2#design"/>
      </prov:Association>
    </prov:qualifiedAssociation>
    <prov:qualifiedUsage>
      <prov:Usage rdf:about="http://sys-bio.org/Activity_design_generation/predicted_usage/1.0.0">
        <sbol:displayId>predicted_usage</sbol:displayId>
        <sbol:persistentIdentity rdf:resource="http://sys-bio.org/Activity_design_generation/predicted_usage"/>
        <sbol:version>1.0.0</sbol:version>
        <prov:entity rdf:resource="http://sys-bio.org/Model_predicted/1.0.0"/>
        <prov:hadRole rdf:resource="http://sbols.org/v2#learn"/>
      </prov:Usage>
    </prov:qualifiedUsage>
  </prov:Activity>
  <prov:Activity rdf:about="http://sys-bio.org/Activity_fit_model_generation/1.0.0">
    <sbol:displayId>Activity_fit_model_generation</sbol:displayId>
    <sbol:persistentIdentity rdf:resource="http://sys-bio.org/Activity_fit_model_generation"/>
    <sbol:version>1.0.0</sbol:version>
    <prov:qualifiedAssociation>
      <prov:Association rdf:about="http://sys-bio.org/Activity_fit_model_generation/fit_model_generation_association/1.0.0">
        <sbol:displayId>fit_model_generation_association</sbol:displayId>
        <sbol:persistentIdentity rdf:resource="http://sys-bio.org/Activity_fit_model_generation/fit_model_generation_association"
      />
        <sbol:version>1.0.0</sbol:version>
        <prov:agent rdf:resource="https://synbiohub.org/jschmoe/1.0.0"/>
        <prov:hadPlan rdf:resource="http://sys-bio.org/Plan_model_fitting/1.0.0"/>
        <prov:hadRole rdf:resource="http://sbols.org/v2#learn"/>
      </prov:Association>
    </prov:qualifiedAssociation>
```

```
      <prov:qualifiedUsage>
        <prov:Usage rdf:about="http://sys-bio.org/Activity_fit_model_generation/raw_data_usage/1.0.0">
          <sbol:displayId>raw_data_usage</sbol:displayId>
          <sbol:persistentIdentity rdf:resource="http://sys-bio.org/Activity_fit_model_generation/raw_data_usage"/>
          <sbol:version>1.0.0</sbol:version>
          <prov:entity rdf:resource="http://sys-bio.org/ExperimentalData_raw_data/1.0.0"/>
          <prov:hadRole rdf:resource="http://sbols.org/v2#test"/>
        </prov:Usage>
      </prov:qualifiedUsage>
    </prov:Activity>
    <prov:Activity rdf:about="http://sys-bio.org/Activity_raw_data_generation/1.0.0">
      <sbol:displayId>Activity_raw_data_generation</sbol:displayId>
      <sbol:persistentIdentity rdf:resource="http://sys-bio.org/Activity_raw_data_generation"/>
      <sbol:version>1.0.0</sbol:version>
      <prov:qualifiedAssociation>
        <prov:Association rdf:about="http://sys-bio.org/Activity_raw_data_generation/raw_data_generation_association/1.0.0">
          <sbol:displayId>raw_data_generation_association</sbol:displayId>
          <sbol:persistentIdentity rdf:resource="http://sys-bio.org/Activity_raw_data_generation/raw_data_generation_association"/>
          <sbol:version>1.0.0</sbol:version>
          <prov:agent rdf:resource="http://sys-bio.org/plate_reader_1"/>
          <prov:hadPlan rdf:resource="http://sys-bio.org/Plan_characterization_protocol/1.0.0"/>
          <prov:hadRole rdf:resource="http://sbols.org/v2#test"/>
        </prov:Association>
      </prov:qualifiedAssociation>
      <prov:qualifiedUsage>
        <prov:Usage rdf:about="http://sys-bio.org/Activity_raw_data_generation/build_usage/1.0.0">
          <sbol:displayId>build_usage</sbol:displayId>
          <sbol:persistentIdentity rdf:resource="http://sys-bio.org/Activity_raw_data_generation/build_usage"/>
          <sbol:version>1.0.0</sbol:version>
          <prov:entity rdf:resource="http://sys-bio.org/Implementation_build/1.0.0"/>
          <prov:hadRole rdf:resource="http://sbols.org/v2#build"/>
        </prov:Usage>
      </prov:qualifiedUsage>
    </prov:Activity>
    <sbol:Attachment rdf:about="http://sys-bio.org/Attachment_characterization_protocol/1.0.0">
      <sbol:displayId>Attachment_characterization_protocol</sbol:displayId>
      <sbol:persistentIdentity rdf:resource="http://sys-bio.org/Attachment_characterization_protocol"/>
      <sbol:source rdf:resource="dynamic_measurement.mdx"/>
      <sbol:version>1.0.0</sbol:version>
    </sbol:Attachment>
    <sbol:Attachment rdf:about="http://sys-bio.org/Attachment_model_fitting_script/1.0.0">
      <sbol:displayId>Attachment_model_fitting_script</sbol:displayId>
      <sbol:format rdf:resource="http://www.ebi.ac.uk/swo/SWO_0000147"></sbol:format>
      <sbol:persistentIdentity rdf:resource="http://sys-bio.org/Attachment_model_fitting_script"/>
      <sbol:source rdf:resource="file://server.sys-bio.org/Dev/optimize.py"/>
      <sbol:version>1.0.0</sbol:version>
    </sbol:Attachment>
    <sbol:ExperimentalData rdf:about="http://sys-bio.org/ExperimentalData_raw_data/1.0.0">
      <sbol:displayId>ExperimentalData_raw_data</sbol:displayId>
      <sbol:persistentIdentity rdf:resource="http://sys-bio.org/ExperimentalData_raw_data"/>
      <sbol:attachment rdf:resource="http://sys-bio.org/Attachment_raw_data/1.0.0"/>
      <sbol:version>1.0.0</sbol:version>
      <prov:wasDerivedFrom rdf:resource="http://sys-bio.org/Implementation_build/1.0.0"/>
      <prov:wasGeneratedBy rdf:resource="http://sys-bio.org/Activity_raw_data_generation/1.0.0"/>
    </sbol:ExperimentalData>
    <sbol:Attachment rdf:about="http://sys-bio.org/Attachment_raw_data/1.0.0">
      <sbol:displayId>Attachment_raw_data</sbol:displayId>
      <sbol:format rdf:resource="http://edamontology.org/format_3752"/>
      <sbol:persistentIdentity rdf:resource="http://sys-bio.org/Attachment_raw_data"/>
      <sbol:source rdf:resource="file://server.sys-bio.org/data/plate_reader_expt_012218.csv"/>
      <sbol:version>1.0.0</sbol:version>
    </sbol:Attachment>
    <sbol:Implementation rdf:about="http://sys-bio.org/Implementation_build/1.0.0">
      <sbol:displayId>Implementation_build</sbol:displayId>
      <sbol:persistentIdentity rdf:resource="http://sys-bio.org/Implementation_build"/>
      <sbol:version>1.0.0</sbol:version>
      <prov:wasDerivedFrom rdf:resource="http://sys-bio.org/ModuleDefinition_design/1.0.0"/>
      <prov:wasGeneratedBy rdf:resource="http://sys-bio.org/Activity_build_generation/1.0.0"/>
    </sbol:Implementation>
```

```
<sbol:Model rdf:about="http://sys-bio.org/Model_fit_model/1.0.0">
  <sbol:displayId>Model_fit_model</sbol:displayId>
  <sbol:framework rdf:resource="http://identifiers.org/biomodels.sbo/SBO:0000062"/>
  <sbol:language rdf:resource="http://identifiers.org/edam/format_2585"/>
  <sbol:persistentIdentity rdf:resource="http://sys-bio.org/Model_fit_model"/>
  <sbol:source rdf:resource="file://server.sys-bio.org/models/model_fit.xml"/>
  <sbol:version>1.0.0</sbol:version>
  <prov:wasDerivedFrom rdf:resource="http://sys-bio.org/ExperimentalData_raw_data/1.0.0"/>
  <prov:wasGeneratedBy rdf:resource="http://sys-bio.org/Activity_fit_model_generation/1.0.0"/>
</sbol:Model>
<sbol:Model rdf:about="http://sys-bio.org/Model_predicted/1.0.0">
  <sbol:displayId>Model_predicted</sbol:displayId>
  <sbol:framework rdf:resource="http://identifiers.org/biomodels.sbo/SBO:0000062"/>
  <sbol:language rdf:resource="http://identifiers.org/edam/format_2585"/>
  <sbol:persistentIdentity rdf:resource="http://sys-bio.org/Model_predicted"/>
  <sbol:source rdf:resource="file://server.sys-bio.org/circuit_simulation.xml"/>
  <sbol:version>1.0.0</sbol:version>
</sbol:Model>
<sbol:ModuleDefinition rdf:about="http://sys-bio.org/ModuleDefinition_design/1.0.0">
  <sbol:displayId>ModuleDefinition_design</sbol:displayId>
  <sbol:persistentIdentity rdf:resource="http://sys-bio.org/ModuleDefinition_design"/>
  <sbol:version>1.0.0</sbol:version>
  <prov:wasDerivedFrom rdf:resource="http://sys-bio.org/Model_predicted/1.0.0"/>
  <prov:wasGeneratedBy rdf:resource="http://sys-bio.org/Activity_design_generation/1.0.0"/>
</sbol:ModuleDefinition>
<prov:Plan rdf:about="http://sys-bio.org/Plan_characterization_protocol/1.0.0">
  <sbol:attachment rdf:resource="http://sys-bio.org/Attachment_characterization_protocol/1.0.0"/>
  <sbol:displayId>Plan_characterization_protocol</sbol:displayId>
  <sbol:persistentIdentity rdf:resource="http://sys-bio.org/Plan_characterization_protocol"/>
  <sbol:version>1.0.0</sbol:version>
</prov:Plan>
<prov:Plan rdf:about="http://sys-bio.org/Plan_conversion/1.0.0">
  <dcterms:description>SBML to SBOL conversion</dcterms:description>
  <sbol:displayId>Plan_conversion</sbol:displayId>
  <sbol:persistentIdentity rdf:resource="http://sys-bio.org/Plan_conversion"/>
  <sbol:version>1.0.0</sbol:version>
</prov:Plan>
<prov:Plan rdf:about="http://sys-bio.org/Plan_gibson_assembly/1.0.0">
  <dcterms:description>DNA assembly</dcterms:description>
  <sbol:displayId>Plan_gibson_assembly</sbol:displayId>
  <sbol:persistentIdentity rdf:resource="http://sys-bio.org/Plan_gibson_assembly"/>
  <sbol:version>1.0.0</sbol:version>
</prov:Plan>
<prov:Plan rdf:about="http://sys-bio.org/Plan_model_fitting/1.0.0">
  <sbol:attachment rdf:resource="http://sys-bio.org/Attachment_model_fitting_script/1.0.0"/>
  <sbol:displayId>Plan_model_fitting</sbol:displayId>
  <sbol:persistentIdentity rdf:resource="http://sys-bio.org/Plan_model_fitting"/>
  <sbol:version>1.0.0</sbol:version>
</prov:Plan>
<prov:Agent rdf:about="http://sys-bio.org/plate_reader_1/1.0.0">
  <sbol:persistentIdentity rdf:resource="http://sys-bio.org/plate_reader_1"></sbol:persistentIdentity>
  <sbol:version>1.0.0</sbol:version>
  <sbol:displayId>plate_reader_1</sbol:displayId>
</prov:Agent>
<prov:Agent rdf:about="http://www.async.ece.utah.edu/ibiosim/1.0.0">
  <dcterms:description>Software application for the modeling, analysis, and design of genetic circuits</dcterms:description>
  <sbol:persistentIdentity rdf:resource="http://www.async.ece.utah.edu/ibiosim"/>
  <sbol:version>1.0.0</sbol:version>
  <sbol:displayId>ibiosim</sbol:displayId>
</prov:Agent>
<prov:Agent rdf:about="https://synbiohub.org/jdoe/1.0.0">
  <dcterms:description>Lab Technician</dcterms:description>
  <dcterms:title>John Doe</dcterms:title>
  <sbol:persistentIdentity rdf:resource="https://synbiohub.org/jdoe"/>
  <sbol:version>1.0.0</sbol:version>
  <sbol:displayId>jdoe</sbol:displayId>
</prov:Agent>
<prov:Agent rdf:about="https://synbiohub.org/jschmoe/1.0.0">
  <dcterms:description>Research Fellow</dcterms:description>
```

```
    <dcterms:title>Joe Schmoe</dcterms:title>
    <sbol:persistentIdentity rdf:resource="https://synbiohub.org/jschmoe"/>
    <sbol:version>1.0.0</sbol:version>
    <sbol:displayId>jschmoe</sbol:displayId>
  </prov:Agent>
</rdf:RDF>
```

#### 2.2.1 *Example - Combinatorial Derivation*

This example illustrates how the Prov ontology should be used to reference to link a generated design to the combinatorial derivation that it was generated from. In this example, there is a top-level derivation (Promoter_Derivation) which specifies two possible promoters for this design, as well as an additional derivation (Terminator_Derivation) to be used for the Gen_Component. The second derivation (Terminator_Derivation) specifies two possible terminators to used within the Gen_Component. The derived design (Derivation_example_GeneratedInstance11) has a reference in the `wasDerivedFroms` list to the `CombinatorialDerivation` that it was derived from (i.e., Promoter_Derivation). Also, each component has reference in the `wasDerivedFroms` list to the component within the `template` that it is derived from. The Gen_Component in this derived design refers to a derived design for it (i.e., Gen_GeneratedInstance1). This design has a reference in the `wasDerivedFroms` list that refers to the `CombinatorialDerivation` that is used to derive it (i.e., Terminator_Derivation). Once again, each component has a reference in the `wasDerivedFroms` list to the component within the `template` that it is derived from. The advantage of these provenance links is that they provide sufficient information to validate that this derived design has been properly derived from the specified `CombinatorialDerivation`s.

```
<?xml version="1.0" ?>
<rdf:RDF xmlns:rdf="http://www.w3.org/1999/02/22-rdf-syntax-ns#" xmlns:sbh="http://wiki.synbiohub.org/wiki/Terms/synbiohub#"
      xmlns:sbol="http://sbols.org/v2#" xmlns:dcterms="http://purl.org/dc/terms/" xmlns:prov="http://www.w3.org/ns/prov#" xmlns:dc
      ="http://purl.org/dc/elements/1.1/">
  <sbol:ComponentDefinition rdf:about="https://synbiohub.programmingbiology.org/public/Cello_Parts/P1/1">
    <sbol:persistentIdentity rdf:resource="https://synbiohub.programmingbiology.org/public/Cello_Parts/P1"/>
    <sbol:displayId>P1</sbol:displayId>
    <sbol:version>1</sbol:version>
    <prov:wasGeneratedBy rdf:resource="https://synbiohub.programmingbiology.org/public/Cello_Parts/CelloUCF2sbol_Activity/1"/>
    <dcterms:title>P1</dcterms:title>
    <dcterms:created>2018-05-09T19:41:51.056Z</dcterms:created>
    <sbh:ownedBy rdf:resource="https://synbiohub.programmingbiology.org/user/myers"/>
    <sbh:topLevel rdf:resource="https://synbiohub.programmingbiology.org/public/Cello_Parts/P1/1"/>
    <sbol:type rdf:resource="http://www.biopax.org/release/biopax-level3.owl#DnaRegion"/>
    <sbol:role rdf:resource="http://identifiers.org/so/SO:0000139"/>
    <sbol:sequence rdf:resource="https://synbiohub.programmingbiology.org/public/Cello_Parts/P1_sequence/1"/>
  </sbol:ComponentDefinition>
  <sbol:ComponentDefinition rdf:about="http://async.ece.utah.edu/Gen/1">
    <sbol:persistentIdentity rdf:resource="http://async.ece.utah.edu/Gen"/>
    <sbol:displayId>Gen</sbol:displayId>
    <sbol:version>1</sbol:version>
    <prov:wasGeneratedBy rdf:resource="http://async.ece.utah.edu/Derivation_example_GeneratedInstance21_SBOLDesignerActivity/1"/>
    <prov:wasGeneratedBy rdf:resource="http://async.ece.utah.edu/Derivation_example_SBOLDesignerActivity/1"/>
    <sbol:type rdf:resource="http://www.biopax.org/release/biopax-level3.owl#DnaRegion"/>
    <sbol:role rdf:resource="http://identifiers.org/so/SO:0000804"/>
    <sbol:component>
      <sbol:Component rdf:about="http://async.ece.utah.edu/Gen/RBS_Component/1">
        <sbol:persistentIdentity rdf:resource="http://async.ece.utah.edu/Gen/RBS_Component"/>
        <sbol:displayId>RBS_Component</sbol:displayId>
        <sbol:version>1</sbol:version>
        <sbol:access rdf:resource="http://sbols.org/v2#public"/>
        <sbol:definition rdf:resource="https://synbiohub.programmingbiology.org/public/Cello_Parts/P1/1"/>
      </sbol:Component>
    </sbol:component>
    <sbol:component>
      <sbol:Component rdf:about="http://async.ece.utah.edu/Gen/Ter_Component/1">
        <sbol:persistentIdentity rdf:resource="http://async.ece.utah.edu/Gen/Ter_Component"/>
        <sbol:displayId>Ter_Component</sbol:displayId>
        <sbol:version>1</sbol:version>
        <sbol:access rdf:resource="http://sbols.org/v2#public"/>
```

```
      <sbol:definition rdf:resource="http://async.ece.utah.edu/Ter/1"/>
    </sbol:Component>
  </sbol:component>
  <sbol:component>
    <sbol:Component rdf:about="http://async.ece.utah.edu/Gen/CDS_Component/1">
      <sbol:persistentIdentity rdf:resource="http://async.ece.utah.edu/Gen/CDS_Component"/>
      <sbol:displayId>CDS_Component</sbol:displayId>
      <sbol:version>1</sbol:version>
      <sbol:access rdf:resource="http://sbols.org/v2#public"/>
      <sbol:definition rdf:resource="https://synbiohub.programmingbiology.org/public/Cello_Parts/PhlF/1"/>
    </sbol:Component>
  </sbol:component>
  <sbol:sequenceAnnotation>
    <sbol:SequenceAnnotation rdf:about="http://async.ece.utah.edu/Gen/Gen_SequenceAnnotation1/1">
      <sbol:persistentIdentity rdf:resource="http://async.ece.utah.edu/Gen/Gen_SequenceAnnotation1"/>
      <sbol:displayId>Gen_SequenceAnnotation1</sbol:displayId>
      <sbol:version>1</sbol:version>
      <sbol:location>
        <sbol:Range rdf:about="http://async.ece.utah.edu/Gen/Gen_SequenceAnnotation1/Gen_SequenceAnnotation1_Range/1">
          <sbol:persistentIdentity rdf:resource="http://async.ece.utah.edu/Gen/Gen_SequenceAnnotation1/
   Gen_SequenceAnnotation1_Range"/>
          <sbol:displayId>Gen_SequenceAnnotation1_Range</sbol:displayId>
          <sbol:version>1</sbol:version>
          <sbol:start>34</sbol:start>
          <sbol:end>636</sbol:end>
          <sbol:orientation rdf:resource="http://sbols.org/v2#inline"/>
        </sbol:Range>
      </sbol:location>
      <sbol:component rdf:resource="http://async.ece.utah.edu/Gen/CDS_Component/1"/>
    </sbol:SequenceAnnotation>
  </sbol:sequenceAnnotation>
  <sbol:sequenceAnnotation>
    <sbol:SequenceAnnotation rdf:about="http://async.ece.utah.edu/Gen/Gen_SequenceAnnotation2/1">
      <sbol:persistentIdentity rdf:resource="http://async.ece.utah.edu/Gen/Gen_SequenceAnnotation2"/>
      <sbol:displayId>Gen_SequenceAnnotation2</sbol:displayId>
      <sbol:version>1</sbol:version>
      <sbol:location>
        <sbol:GenericLocation rdf:about="http://async.ece.utah.edu/Gen/Gen_SequenceAnnotation2/GenericLocation/1">
          <sbol:persistentIdentity rdf:resource="http://async.ece.utah.edu/Gen/Gen_SequenceAnnotation2/GenericLocation"/>
          <sbol:displayId>GenericLocation</sbol:displayId>
          <sbol:version>1</sbol:version>
          <sbol:orientation rdf:resource="http://sbols.org/v2#inline"/>
        </sbol:GenericLocation>
      </sbol:location>
      <sbol:component rdf:resource="http://async.ece.utah.edu/Gen/Ter_Component/1"/>
    </sbol:SequenceAnnotation>
  </sbol:sequenceAnnotation>
  <sbol:sequenceAnnotation>
    <sbol:SequenceAnnotation rdf:about="http://async.ece.utah.edu/Gen/Gen_SequenceAnnotation/1">
      <sbol:persistentIdentity rdf:resource="http://async.ece.utah.edu/Gen/Gen_SequenceAnnotation"/>
      <sbol:displayId>Gen_SequenceAnnotation</sbol:displayId>
      <sbol:version>1</sbol:version>
      <sbol:location>
        <sbol:Range rdf:about="http://async.ece.utah.edu/Gen/Gen_SequenceAnnotation/Gen_SequenceAnnotation_Range/1">
          <sbol:persistentIdentity rdf:resource="http://async.ece.utah.edu/Gen/Gen_SequenceAnnotation/
   Gen_SequenceAnnotation_Range"/>
          <sbol:displayId>Gen_SequenceAnnotation_Range</sbol:displayId>
          <sbol:version>1</sbol:version>
          <sbol:start>1</sbol:start>
          <sbol:end>33</sbol:end>
          <sbol:orientation rdf:resource="http://sbols.org/v2#inline"/>
        </sbol:Range>
      </sbol:location>
      <sbol:component rdf:resource="http://async.ece.utah.edu/Gen/RBS_Component/1"/>
    </sbol:SequenceAnnotation>
  </sbol:sequenceAnnotation>
  <sbol:sequenceConstraint>
    <sbol:SequenceConstraint rdf:about="http://async.ece.utah.edu/Gen/Gen_SequenceConstraint/1">
      <sbol:persistentIdentity rdf:resource="http://async.ece.utah.edu/Gen/Gen_SequenceConstraint"/>
```

```
      <sbol:displayId>Gen_SequenceConstraint</sbol:displayId>
      <sbol:version>1</sbol:version>
      <sbol:restriction rdf:resource="http://sbols.org/v2#precedes"/>
      <sbol:subject rdf:resource="http://async.ece.utah.edu/Gen/RBS_Component/1"/>
      <sbol:object rdf:resource="http://async.ece.utah.edu/Gen/CDS_Component/1"/>
    </sbol:SequenceConstraint>
  </sbol:sequenceConstraint>
  <sbol:sequenceConstraint>
    <sbol:SequenceConstraint rdf:about="http://async.ece.utah.edu/Gen/Gen_SequenceConstraint1/1">
      <sbol:persistentIdentity rdf:resource="http://async.ece.utah.edu/Gen/Gen_SequenceConstraint1"/>
      <sbol:displayId>Gen_SequenceConstraint1</sbol:displayId>
      <sbol:version>1</sbol:version>
      <sbol:restriction rdf:resource="http://sbols.org/v2#precedes"/>
      <sbol:subject rdf:resource="http://async.ece.utah.edu/Gen/CDS_Component/1"/>
      <sbol:object rdf:resource="http://async.ece.utah.edu/Gen/Ter_Component/1"/>
    </sbol:SequenceConstraint>
  </sbol:sequenceConstraint>
  <sbol:sequence rdf:resource="http://async.ece.utah.edu/GenSequence/1"/>
</sbol:ComponentDefinition>
<sbol:ComponentDefinition rdf:about="https://synbiohub.programmingbiology.org/public/Cello_Parts/pAmtR/1">
  <sbol:persistentIdentity rdf:resource="https://synbiohub.programmingbiology.org/public/Cello_Parts/pAmtR"/>
  <sbol:displayId>pAmtR</sbol:displayId>
  <sbol:version>1</sbol:version>
  <prov:wasGeneratedBy rdf:resource="https://synbiohub.programmingbiology.org/public/Cello_Parts/CelloUCF2sbol_Activity/1"/>
  <dcterms:title>pAmtR</dcterms:title>
  <dcterms:created>2018-05-09T19:41:51.056Z</dcterms:created>
  <sbh:ownedBy rdf:resource="https://synbiohub.programmingbiology.org/user/myers"/>
  <sbh:topLevel rdf:resource="https://synbiohub.programmingbiology.org/public/Cello_Parts/pAmtR/1"/>
  <sbol:type rdf:resource="http://www.biopax.org/release/biopax-level3.owl#DnaRegion"/>
  <sbol:role rdf:resource="http://identifiers.org/so/SO:0000167"/>
  <sbol:sequence rdf:resource="https://synbiohub.programmingbiology.org/public/Cello_Parts/pAmtR_sequence/1"/>
</sbol:ComponentDefinition>
<sbol:ComponentDefinition rdf:about="https://synbiohub.programmingbiology.org/public/Cello_Parts/PhlF/1">
  <sbol:persistentIdentity rdf:resource="https://synbiohub.programmingbiology.org/public/Cello_Parts/PhlF"/>
  <sbol:displayId>PhlF</sbol:displayId>
  <sbol:version>1</sbol:version>
  <prov:wasGeneratedBy rdf:resource="https://synbiohub.programmingbiology.org/public/Cello_Parts/CelloUCF2sbol_Activity/1"/>
  <dcterms:title>PhlF</dcterms:title>
  <dcterms:created>2018-05-09T19:41:51.056Z</dcterms:created>
  <sbh:ownedBy rdf:resource="https://synbiohub.programmingbiology.org/user/myers"/>
  <sbh:topLevel rdf:resource="https://synbiohub.programmingbiology.org/public/Cello_Parts/PhlF/1"/>
  <sbol:type rdf:resource="http://www.biopax.org/release/biopax-level3.owl#DnaRegion"/>
  <sbol:role rdf:resource="http://identifiers.org/so/SO:0000316"/>
  <sbol:sequence rdf:resource="https://synbiohub.programmingbiology.org/public/Cello_Parts/PhlF_sequence/1"/>
</sbol:ComponentDefinition>
<sbol:ComponentDefinition rdf:about="http://async.ece.utah.edu/Gen_GeneratedInstance1/1">
  <sbol:persistentIdentity rdf:resource="http://async.ece.utah.edu/Gen_GeneratedInstance1"/>
  <sbol:displayId>Gen_GeneratedInstance1</sbol:displayId>
  <sbol:version>1</sbol:version>
  <prov:wasDerivedFrom rdf:resource="http://async.ece.utah.edu/Terminator_Derivation/1"/>
  <prov:wasDerivedFrom rdf:resource="http://async.ece.utah.edu/Gen/1"/>
  <prov:wasGeneratedBy rdf:resource="http://async.ece.utah.edu/Derivation_example_GeneratedInstance21_SBOLDesignerActivity/1"/>
  <prov:wasGeneratedBy rdf:resource="http://async.ece.utah.edu/Derivation_example_SBOLDesignerActivity/1"/>
  <sbol:type rdf:resource="http://www.biopax.org/release/biopax-level3.owl#DnaRegion"/>
  <sbol:role rdf:resource="http://identifiers.org/so/SO:0000804"/>
  <sbol:component>
    <sbol:Component rdf:about="http://async.ece.utah.edu/Gen_GeneratedInstance1/Ter_Component/1">
      <sbol:persistentIdentity rdf:resource="http://async.ece.utah.edu/Gen_GeneratedInstance1/Ter_Component"/>
      <sbol:displayId>Ter_Component</sbol:displayId>
      <sbol:version>1</sbol:version>
      <prov:wasDerivedFrom rdf:resource="http://async.ece.utah.edu/Gen/Ter_Component/1"/>
      <sbol:access rdf:resource="http://sbols.org/v2#public"/>
      <sbol:definition rdf:resource="https://synbiohub.programmingbiology.org/public/Cello_Parts/L3S3P31/1"/>
    </sbol:Component>
  </sbol:component>
  <sbol:component>
    <sbol:Component rdf:about="http://async.ece.utah.edu/Gen_GeneratedInstance1/RBS_Component/1">
      <sbol:persistentIdentity rdf:resource="http://async.ece.utah.edu/Gen_GeneratedInstance1/RBS_Component"/>
      <sbol:displayId>RBS_Component</sbol:displayId>
```

```
      <sbol:version>1</sbol:version>
      <prov:wasDerivedFrom rdf:resource="http://async.ece.utah.edu/Gen/RBS_Component/1"/>
      <sbol:access rdf:resource="http://sbols.org/v2#public"/>
      <sbol:definition rdf:resource="https://synbiohub.programmingbiology.org/public/Cello_Parts/P1/1"/>
    </sbol:Component>
  </sbol:component>
  <sbol:component>
    <sbol:Component rdf:about="http://async.ece.utah.edu/Gen_GeneratedInstance1/CDS_Component/1">
      <sbol:persistentIdentity rdf:resource="http://async.ece.utah.edu/Gen_GeneratedInstance1/CDS_Component"/>
      <sbol:displayId>CDS_Component</sbol:displayId>
      <sbol:version>1</sbol:version>
      <prov:wasDerivedFrom rdf:resource="http://async.ece.utah.edu/Gen/CDS_Component/1"/>
      <sbol:access rdf:resource="http://sbols.org/v2#public"/>
      <sbol:definition rdf:resource="https://synbiohub.programmingbiology.org/public/Cello_Parts/PhlF/1"/>
    </sbol:Component>
  </sbol:component>
  <sbol:sequenceConstraint>
    <sbol:SequenceConstraint rdf:about="http://async.ece.utah.edu/Gen_GeneratedInstance1/Gen_SequenceConstraint1/1">
      <sbol:persistentIdentity rdf:resource="http://async.ece.utah.edu/Gen_GeneratedInstance1/Gen_SequenceConstraint1"/>
      <sbol:displayId>Gen_SequenceConstraint1</sbol:displayId>
      <sbol:version>1</sbol:version>
      <sbol:restriction rdf:resource="http://sbols.org/v2#precedes"/>
      <sbol:subject rdf:resource="http://async.ece.utah.edu/Gen_GeneratedInstance1/CDS_Component/1"/>
      <sbol:object rdf:resource="http://async.ece.utah.edu/Gen_GeneratedInstance1/Ter_Component/1"/>
    </sbol:SequenceConstraint>
  </sbol:sequenceConstraint>
  <sbol:sequenceConstraint>
    <sbol:SequenceConstraint rdf:about="http://async.ece.utah.edu/Gen_GeneratedInstance1/Gen_SequenceConstraint/1">
      <sbol:persistentIdentity rdf:resource="http://async.ece.utah.edu/Gen_GeneratedInstance1/Gen_SequenceConstraint"/>
      <sbol:displayId>Gen_SequenceConstraint</sbol:displayId>
      <sbol:version>1</sbol:version>
      <sbol:restriction rdf:resource="http://sbols.org/v2#precedes"/>
      <sbol:subject rdf:resource="http://async.ece.utah.edu/Gen_GeneratedInstance1/RBS_Component/1"/>
      <sbol:object rdf:resource="http://async.ece.utah.edu/Gen_GeneratedInstance1/CDS_Component/1"/>
    </sbol:SequenceConstraint>
  </sbol:sequenceConstraint>
  <sbol:sequence rdf:resource="http://async.ece.utah.edu/GenSequence/1"/>
</sbol:ComponentDefinition>
<sbol:ComponentDefinition rdf:about="http://async.ece.utah.edu/Derivation_example/1">
  <sbol:persistentIdentity rdf:resource="http://async.ece.utah.edu/Derivation_example"/>
  <sbol:displayId>Derivation_example</sbol:displayId>
  <sbol:version>1</sbol:version>
  <prov:wasGeneratedBy rdf:resource="http://async.ece.utah.edu/Derivation_example_GeneratedInstance21_SBOLDesignerActivity/1"/>
  <prov:wasGeneratedBy rdf:resource="http://async.ece.utah.edu/Derivation_example_SBOLDesignerActivity/1"/>
  <dcterms:title>Derivation Example</dcterms:title>
  <dcterms:description>An example derivation</dcterms:description>
  <sbol:type rdf:resource="http://www.biopax.org/release/biopax-level3.owl#DnaRegion"/>
  <sbol:role rdf:resource="http://identifiers.org/so/SO:0000804"/>
  <sbol:component>
    <sbol:Component rdf:about="http://async.ece.utah.edu/Derivation_example/Pro_Component/1">
      <sbol:persistentIdentity rdf:resource="http://async.ece.utah.edu/Derivation_example/Pro_Component"/>
      <sbol:displayId>Pro_Component</sbol:displayId>
      <sbol:version>1</sbol:version>
      <sbol:access rdf:resource="http://sbols.org/v2#public"/>
      <sbol:definition rdf:resource="http://async.ece.utah.edu/Pro/1"/>
    </sbol:Component>
  </sbol:component>
  <sbol:component>
    <sbol:Component rdf:about="http://async.ece.utah.edu/Derivation_example/Gen_Component/1">
      <sbol:persistentIdentity rdf:resource="http://async.ece.utah.edu/Derivation_example/Gen_Component"/>
      <sbol:displayId>Gen_Component</sbol:displayId>
      <sbol:version>1</sbol:version>
      <sbol:access rdf:resource="http://sbols.org/v2#public"/>
      <sbol:definition rdf:resource="http://async.ece.utah.edu/Gen/1"/>
    </sbol:Component>
  </sbol:component>
  <sbol:sequenceAnnotation>
    <sbol:SequenceAnnotation rdf:about="http://async.ece.utah.edu/Derivation_example/Derivation_example_SequenceAnnotation1/1">
      <sbol:persistentIdentity rdf:resource="http://async.ece.utah.edu/Derivation_example/
```

```
      Derivation_example_SequenceAnnotation1"/>
        <sbol:displayId>Derivation_example_SequenceAnnotation1</sbol:displayId>
        <sbol:version>1</sbol:version>
        <sbol:location>
          <sbol:Range rdf:about="http://async.ece.utah.edu/Derivation_example/Derivation_example_SequenceAnnotation1/
      Derivation_example_SequenceAnnotation1_Range/1">
            <sbol:persistentIdentity rdf:resource="http://async.ece.utah.edu/Derivation_example/
      Derivation_example_SequenceAnnotation1/Derivation_example_SequenceAnnotation1_Range"/>
            <sbol:displayId>Derivation_example_SequenceAnnotation1_Range</sbol:displayId>
            <sbol:version>1</sbol:version>
            <sbol:start>1</sbol:start>
            <sbol:end>636</sbol:end>
            <sbol:orientation rdf:resource="http://sbols.org/v2#inline"/>
          </sbol:Range>
        </sbol:location>
        <sbol:component rdf:resource="http://async.ece.utah.edu/Derivation_example/Gen_Component/1"/>
      </sbol:SequenceAnnotation>
    </sbol:sequenceAnnotation>
    <sbol:sequenceAnnotation>
      <sbol:SequenceAnnotation rdf:about="http://async.ece.utah.edu/Derivation_example/Derivation_example_SequenceAnnotation/1">
        <sbol:persistentIdentity rdf:resource="http://async.ece.utah.edu/Derivation_example/Derivation_example_SequenceAnnotation
      "/>
        <sbol:displayId>Derivation_example_SequenceAnnotation</sbol:displayId>
        <sbol:version>1</sbol:version>
        <sbol:location>
          <sbol:GenericLocation rdf:about="http://async.ece.utah.edu/Derivation_example/Derivation_example_SequenceAnnotation/
      GenericLocation/1">
            <sbol:persistentIdentity rdf:resource="http://async.ece.utah.edu/Derivation_example/
      Derivation_example_SequenceAnnotation/GenericLocation"/>
            <sbol:displayId>GenericLocation</sbol:displayId>
            <sbol:version>1</sbol:version>
            <sbol:orientation rdf:resource="http://sbols.org/v2#inline"/>
          </sbol:GenericLocation>
        </sbol:location>
        <sbol:component rdf:resource="http://async.ece.utah.edu/Derivation_example/Pro_Component/1"/>
      </sbol:SequenceAnnotation>
    </sbol:sequenceAnnotation>
    <sbol:sequenceConstraint>
      <sbol:SequenceConstraint rdf:about="http://async.ece.utah.edu/Derivation_example/Derivation_example_SequenceConstraint/1">
        <sbol:persistentIdentity rdf:resource="http://async.ece.utah.edu/Derivation_example/Derivation_example_SequenceConstraint
      "/>
        <sbol:displayId>Derivation_example_SequenceConstraint</sbol:displayId>
        <sbol:version>1</sbol:version>
        <sbol:restriction rdf:resource="http://sbols.org/v2#precedes"/>
        <sbol:subject rdf:resource="http://async.ece.utah.edu/Derivation_example/Pro_Component/1"/>
        <sbol:object rdf:resource="http://async.ece.utah.edu/Derivation_example/Gen_Component/1"/>
      </sbol:SequenceConstraint>
    </sbol:sequenceConstraint>
    <sbol:sequence rdf:resource="http://async.ece.utah.edu/Derivation_exampleSequence/1"/>
  </sbol:ComponentDefinition>
  <sbol:ComponentDefinition rdf:about="https://synbiohub.programmingbiology.org/public/Cello_Parts/L3S3P31/1">
    <sbol:persistentIdentity rdf:resource="https://synbiohub.programmingbiology.org/public/Cello_Parts/L3S3P31"/>
    <sbol:displayId>L3S3P31</sbol:displayId>
    <sbol:version>1</sbol:version>
    <prov:wasGeneratedBy rdf:resource="https://synbiohub.programmingbiology.org/public/Cello_Parts/CelloUCF2sbol_Activity/1"/>
    <dcterms:title>L3S3P31</dcterms:title>
    <dcterms:created>2018-05-09T19:41:51.056Z</dcterms:created>
    <sbh:ownedBy rdf:resource="https://synbiohub.programmingbiology.org/user/myers"/>
    <sbh:topLevel rdf:resource="https://synbiohub.programmingbiology.org/public/Cello_Parts/L3S3P31/1"/>
    <sbol:type rdf:resource="http://www.biopax.org/release/biopax-level3.owl#DnaRegion"/>
    <sbol:role rdf:resource="http://identifiers.org/so/SO:0000141"/>
    <sbol:sequence rdf:resource="https://synbiohub.programmingbiology.org/public/Cello_Parts/L3S3P31_sequence/1"/>
  </sbol:ComponentDefinition>
  <sbol:ComponentDefinition rdf:about="http://async.ece.utah.edu/Pro/1">
    <sbol:persistentIdentity rdf:resource="http://async.ece.utah.edu/Pro"/>
    <sbol:displayId>Pro</sbol:displayId>
    <sbol:version>1</sbol:version>
    <prov:wasGeneratedBy rdf:resource="http://async.ece.utah.edu/Derivation_example_GeneratedInstance21_SBOLDesignerActivity/1"/>
    <prov:wasGeneratedBy rdf:resource="http://async.ece.utah.edu/Derivation_example_SBOLDesignerActivity/1"/>
```

```xml
    <sbol:type rdf:resource="http://www.biopax.org/release/biopax-level3.owl#DnaRegion"/>
    <sbol:role rdf:resource="http://identifiers.org/so/SO:0000167"/>
  </sbol:ComponentDefinition>
  <sbol:ComponentDefinition rdf:about="http://async.ece.utah.edu/Derivation_example_GeneratedInstance11/1">
    <sbol:persistentIdentity rdf:resource="http://async.ece.utah.edu/Derivation_example_GeneratedInstance11"/>
    <sbol:displayId>Derivation_example_GeneratedInstance11</sbol:displayId>
    <sbol:version>1</sbol:version>
    <prov:wasDerivedFrom rdf:resource="http://async.ece.utah.edu/Derivation_example/1"/>
    <prov:wasDerivedFrom rdf:resource="http://async.ece.utah.edu/Promoter_Derivation/1"/>
    <prov:wasGeneratedBy rdf:resource="http://async.ece.utah.edu/Derivation_example_GeneratedInstance21_SBOLDesignerActivity/1"/>
    <prov:wasGeneratedBy rdf:resource="http://async.ece.utah.edu/Derivation_example_SBOLDesignerActivity/1"/>
    <dcterms:title>Derivation Example</dcterms:title>
    <dcterms:description>An example derivation</dcterms:description>
    <sbol:type rdf:resource="http://www.biopax.org/release/biopax-level3.owl#DnaRegion"/>
    <sbol:role rdf:resource="http://identifiers.org/so/SO:0000804"/>
    <sbol:component>
      <sbol:Component rdf:about="http://async.ece.utah.edu/Derivation_example_GeneratedInstance11/Pro_Component/1">
        <sbol:persistentIdentity rdf:resource="http://async.ece.utah.edu/Derivation_example_GeneratedInstance11/Pro_Component"/>
        <sbol:displayId>Pro_Component</sbol:displayId>
        <sbol:version>1</sbol:version>
        <prov:wasDerivedFrom rdf:resource="http://async.ece.utah.edu/Derivation_example/Pro_Component/1"/>
        <sbol:access rdf:resource="http://sbols.org/v2#public"/>
        <sbol:definition rdf:resource="https://synbiohub.programmingbiology.org/public/Cello_Parts/pAmtR/1"/>
      </sbol:Component>
    </sbol:component>
    <sbol:component>
      <sbol:Component rdf:about="http://async.ece.utah.edu/Derivation_example_GeneratedInstance11/Gen_Component/1">
        <sbol:persistentIdentity rdf:resource="http://async.ece.utah.edu/Derivation_example_GeneratedInstance11/Gen_Component"/>
        <sbol:displayId>Gen_Component</sbol:displayId>
        <sbol:version>1</sbol:version>
        <prov:wasDerivedFrom rdf:resource="http://async.ece.utah.edu/Derivation_example/Gen_Component/1"/>
        <sbol:access rdf:resource="http://sbols.org/v2#public"/>
        <sbol:definition rdf:resource="http://async.ece.utah.edu/Gen_GeneratedInstance1/1"/>
      </sbol:Component>
    </sbol:component>
    <sbol:sequenceConstraint>
      <sbol:SequenceConstraint rdf:about="http://async.ece.utah.edu/Derivation_example_GeneratedInstance11/
     Derivation_example_SequenceConstraint/1">
        <sbol:persistentIdentity rdf:resource="http://async.ece.utah.edu/Derivation_example_GeneratedInstance11/
     Derivation_example_SequenceConstraint"/>
        <sbol:displayId>Derivation_example_SequenceConstraint</sbol:displayId>
        <sbol:version>1</sbol:version>
        <sbol:restriction rdf:resource="http://sbols.org/v2#precedes"/>
        <sbol:subject rdf:resource="http://async.ece.utah.edu/Derivation_example_GeneratedInstance11/Pro_Component/1"/>
        <sbol:object rdf:resource="http://async.ece.utah.edu/Derivation_example_GeneratedInstance11/Gen_Component/1"/>
      </sbol:SequenceConstraint>
    </sbol:sequenceConstraint>
    <sbol:sequence rdf:resource="http://async.ece.utah.edu/Derivation_exampleSequence/1"/>
  </sbol:ComponentDefinition>
  <sbol:ComponentDefinition rdf:about="http://async.ece.utah.edu/Ter/1">
    <sbol:persistentIdentity rdf:resource="http://async.ece.utah.edu/Ter"/>
    <sbol:displayId>Ter</sbol:displayId>
    <sbol:version>1</sbol:version>
    <prov:wasGeneratedBy rdf:resource="http://async.ece.utah.edu/Derivation_example_GeneratedInstance21_SBOLDesignerActivity/1"/>
    <prov:wasGeneratedBy rdf:resource="http://async.ece.utah.edu/Derivation_example_SBOLDesignerActivity/1"/>
    <sbol:type rdf:resource="http://www.biopax.org/release/biopax-level3.owl#DnaRegion"/>
    <sbol:role rdf:resource="http://identifiers.org/so/SO:0000141"/>
  </sbol:ComponentDefinition>
  <sbol:Sequence rdf:about="https://synbiohub.programmingbiology.org/public/Cello_Parts/L3S2P55_sequence/1">
    <sbol:persistentIdentity rdf:resource="https://synbiohub.programmingbiology.org/public/Cello_Parts/L3S2P55_sequence"/>
    <sbol:displayId>L3S2P55_sequence</sbol:displayId>
    <sbol:version>1</sbol:version>
    <prov:wasGeneratedBy rdf:resource="https://synbiohub.programmingbiology.org/public/Cello_Parts/CelloUCF2sbol_Activity/1"/>
    <dcterms:title>L3S2P55_sequence</dcterms:title>
    <dcterms:created>2018-05-09T19:41:51.056Z</dcterms:created>
    <sbh:ownedBy rdf:resource="https://synbiohub.programmingbiology.org/user/myers"/>
    <sbh:topLevel rdf:resource="https://synbiohub.programmingbiology.org/public/Cello_Parts/L3S2P55_sequence/1"/>
    <sbol:elements>CTCGGTACCAAAGACGAACAATAAGACGCTGAAAAGCGTCTTTTTTCGTTTTGGTCC</sbol:elements>
    <sbol:encoding rdf:resource="http://www.chem.qmul.ac.uk/iubmb/misc/naseq.html"/>
```

```
</sbol:Sequence>
<sbol:Sequence rdf:about="https://synbiohub.programmingbiology.org/public/Cello_Parts/P1_sequence/1">
  <sbol:persistentIdentity rdf:resource="https://synbiohub.programmingbiology.org/public/Cello_Parts/P1_sequence"/>
  <sbol:displayId>P1_sequence</sbol:displayId>
  <sbol:version>1</sbol:version>
  <prov:wasGeneratedBy rdf:resource="https://synbiohub.programmingbiology.org/public/Cello_Parts/CelloUCF2sbol_Activity/1"/>
  <dcterms:title>P1_sequence</dcterms:title>
  <dcterms:created>2018-05-09T19:41:51.056Z</dcterms:created>
  <sbh:ownedBy rdf:resource="https://synbiohub.programmingbiology.org/user/myers"/>
  <sbh:topLevel rdf:resource="https://synbiohub.programmingbiology.org/public/Cello_Parts/P1_sequence/1"/>
  <sbol:elements>CTATGGACTATGTTTGAAAGGGAGAAATACTAG</sbol:elements>
  <sbol:encoding rdf:resource="http://www.chem.qmul.ac.uk/iubmb/misc/naseq.html"/>
</sbol:Sequence>
<sbol:Sequence rdf:about="https://synbiohub.programmingbiology.org/public/Cello_Parts/pAmtR_sequence/1">
  <sbol:persistentIdentity rdf:resource="https://synbiohub.programmingbiology.org/public/Cello_Parts/pAmtR_sequence"/>
  <sbol:displayId>pAmtR_sequence</sbol:displayId>
  <sbol:version>1</sbol:version>
  <prov:wasGeneratedBy rdf:resource="https://synbiohub.programmingbiology.org/public/Cello_Parts/CelloUCF2sbol_Activity/1"/>
  <dcterms:title>pAmtR_sequence</dcterms:title>
  <dcterms:created>2018-05-09T19:41:51.056Z</dcterms:created>
  <sbh:ownedBy rdf:resource="https://synbiohub.programmingbiology.org/user/myers"/>
  <sbh:topLevel rdf:resource="https://synbiohub.programmingbiology.org/public/Cello_Parts/pAmtR_sequence/1"/>
  <sbol:elements>CTTGTCCAACCAAATGATTCGTTACCAATTGACAGTTTCTATCGATCTATAGATAATGCTAGC</sbol:elements>
  <sbol:encoding rdf:resource="http://www.chem.qmul.ac.uk/iubmb/misc/naseq.html"/>
</sbol:Sequence>
<sbol:Sequence rdf:about="https://synbiohub.programmingbiology.org/public/Cello_Parts/pBetI_sequence/1">
  <sbol:persistentIdentity rdf:resource="https://synbiohub.programmingbiology.org/public/Cello_Parts/pBetI_sequence"/>
  <sbol:displayId>pBetI_sequence</sbol:displayId>
  <sbol:version>1</sbol:version>
  <prov:wasGeneratedBy rdf:resource="https://synbiohub.programmingbiology.org/public/Cello_Parts/CelloUCF2sbol_Activity/1"/>
  <dcterms:title>pBetI_sequence</dcterms:title>
  <dcterms:created>2018-05-09T19:41:51.056Z</dcterms:created>
  <sbh:ownedBy rdf:resource="https://synbiohub.programmingbiology.org/user/myers"/>
  <sbh:topLevel rdf:resource="https://synbiohub.programmingbiology.org/public/Cello_Parts/pBetI_sequence/1"/>
  <sbol:elements>AGCGCGGGTGAGAGGGATTCGTTACCAATTGACAATTGATTGGACGTTCAATATAATGCTAGC</sbol:elements>
  <sbol:encoding rdf:resource="http://www.chem.qmul.ac.uk/iubmb/misc/naseq.html"/>
</sbol:Sequence>
<sbol:Sequence rdf:about="http://async.ece.utah.edu/Derivation_exampleSequence/1">
  <sbol:persistentIdentity rdf:resource="http://async.ece.utah.edu/Derivation_exampleSequence"/>
  <sbol:displayId>Derivation_exampleSequence</sbol:displayId>
  <sbol:version>1</sbol:version>
  <prov:wasGeneratedBy rdf:resource="http://async.ece.utah.edu/Derivation_example_GeneratedInstance21_SBOLDesignerActivity/1"/>
  <prov:wasGeneratedBy rdf:resource="http://async.ece.utah.edu/Derivation_example_SBOLDesignerActivity/1"/>
  <sbol:elements>CTATGGACTATGTTTGAAAGGGAGAAATACTAGATGGCACGTACCCCGAGCCGTAGCAGCATTGGTAGCC
  TGCGTAGTCCGCATACCCATAAAGCAATTCTGACCAGCACCATTGAAATCCTGAAAGAATGTGGTTATAGCGGTCTGAGCATT
  GAAAGCGTTGCACGTCGTGCCGGTGCAAGCAAACCGACCATTTATCGTTGGTGGACCAATAAAGCAGCACTGATTGCCGAAGTG
  TATGAAAATGAAAGCGAACAGGTGCGTAAATTTCCGGATCTGGGTAGCTTTAAAGCCGATCTGGATTTTCTGCTGCGTAATCTGT
  GGAAAGTTTGGCGTGAAACCATTTGTGGTGAAGCATTTCGTTGTGTTATTGCAGAAGCACAGCTGGACCCTGCAACCCTGACCCA
  GCTGAAAGATCAGTTTATGGAACGTCGTCGTGAGATGCCGAAAAAACTGGTTGAAAATGCCATTAGCAATGGTGAACTGCCGAAA
  GATACCAATCGTGAACTGCTGCTGGATATGATTTTTGGTTTTTGTTGGTATCGCCTGCTGACCGAACAGCTGACCGTTGAACAGGA
  TATTGAAGAATTTACCTTCCTGCTGATTAATGGTGTTTGTCCGGGTACACAGCGTTAA</sbol:elements>
  <sbol:encoding rdf:resource="http://www.chem.qmul.ac.uk/iubmb/misc/naseq.html"/>
</sbol:Sequence>
<sbol:Sequence rdf:about="http://async.ece.utah.edu/GenSequence/1">
  <sbol:persistentIdentity rdf:resource="http://async.ece.utah.edu/GenSequence"/>
  <sbol:displayId>GenSequence</sbol:displayId>
  <sbol:version>1</sbol:version>
  <prov:wasGeneratedBy rdf:resource="http://async.ece.utah.edu/Derivation_example_GeneratedInstance21_SBOLDesignerActivity/1"/>
  <prov:wasGeneratedBy rdf:resource="http://async.ece.utah.edu/Derivation_example_SBOLDesignerActivity/1"/>
  <sbol:elements>CTATGGACTATGTTTGAAAGGGAGAAATACTAGATGGCACGTACCCCGAGCCGTAGCAGCATTGGTAGCCTG
  CGTAGTCCGCATACCCATAAAGCAATTCTGACCAGCACCATTGAAATCCTGAAAGAATGTGGTTATAGCGGTCTGAGCATTGAAA
  GCGTTGCACGTCGTGCCGGTGCAAGCAAACCGACCATTTATCGTTGGTGGACCAATAAAGCAGCACTGATTGCCGAAGTGTATGA
  AAATGAAAGCGAACAGGTGCGTAAATTTCCGGATCTGGGTAGCTTTAAAGCCGATCTGGATTTTCTGCTGCGTAATCTGTGGAAAG
  TTTGGCGTGAAACCATTTGTGGTGAAGCATTTCGTTGTGTTATTGCAGAAGCACAGCTGGACCCTGCAACCCTGACCCAGCTGAAA
  GATCAGTTTATGGAACGTCGTCGTGAGATGCCGAAAAAACTGGTTGAAAATGCCATTAGCAATGGTGAACTGCCGAAAGATACCA
  ATCGTGAACTGCTGCTGGATATGATTTTTGGTTTTTGTTGGTATCGCCTGCTGACCGAACAGCTGACCGTTGAACAGGATATTGAA
  GAATTTACCTTCCTGCTGATTAATGGTGTTTGTCCGGGTACACAGCGTTAA</sbol:elements>
  <sbol:encoding rdf:resource="http://www.chem.qmul.ac.uk/iubmb/misc/naseq.html"/>
</sbol:Sequence>
```

```
<sbol:Sequence rdf:about="https://synbiohub.programmingbiology.org/public/Cello_Parts/L3S3P31_sequence/1">
  <sbol:persistentIdentity rdf:resource="https://synbiohub.programmingbiology.org/public/Cello_Parts/L3S3P31_sequence"/>
  <sbol:displayId>L3S3P31_sequence</sbol:displayId>
  <sbol:version>1</sbol:version>
  <prov:wasGeneratedBy rdf:resource="https://synbiohub.programmingbiology.org/public/Cello_Parts/CelloUCF2sbol_Activity/1"/>
  <dcterms:title>L3S3P31_sequence</dcterms:title>
  <dcterms:created>2018-05-09T19:41:51.056Z</dcterms:created>
  <sbh:ownedBy rdf:resource="https://synbiohub.programmingbiology.org/user/myers"/>
  <sbh:topLevel rdf:resource="https://synbiohub.programmingbiology.org/public/Cello_Parts/L3S3P31_sequence/1"/>
  <sbol:elements>CCAATTATTGAACACCCTAACGGGTGTTTTTTTTTTTTTGGTCTACC</sbol:elements>
  <sbol:encoding rdf:resource="http://www.chem.qmul.ac.uk/iubmb/misc/naseq.html"/>
</sbol:Sequence>
<sbol:Sequence rdf:about="https://synbiohub.programmingbiology.org/public/Cello_Parts/PhlF_sequence/1">
  <sbol:persistentIdentity rdf:resource="https://synbiohub.programmingbiology.org/public/Cello_Parts/PhlF_sequence"/>
  <sbol:displayId>PhlF_sequence</sbol:displayId>
  <sbol:version>1</sbol:version>
  <prov:wasGeneratedBy rdf:resource="https://synbiohub.programmingbiology.org/public/Cello_Parts/CelloUCF2sbol_Activity/1"/>
  <dcterms:title>PhlF_sequence</dcterms:title>
  <dcterms:created>2018-05-09T19:41:51.056Z</dcterms:created>
  <sbh:ownedBy rdf:resource="https://synbiohub.programmingbiology.org/user/myers"/>
  <sbh:topLevel rdf:resource="https://synbiohub.programmingbiology.org/public/Cello_Parts/PhlF_sequence/1"/>
  <sbol:elements>ATGGCACGTACCCCGAGCCGTAGCAGCATTGGTAGCCTGCGTAGTCCGCATACCCATAAAGCAATTCTGA
  CCAGCACCATTGAAATCCTGAAAGAATGTGGTTATAGCGGTCTGAGCATTGAAAGCGTTGCACGTCGTGCCGGTGCAAGCAAAC
  CGACCATTTATCGTTGGTGGACCAATAAAGCAGCACTGATTGCCGAAGTGTATGAAAATGAAAGCGAACAGGTGCGTAAATTTC
  CGGATCTGGGTAGCTTTAAAGCCGATCTGGATTTTCTGCTGCGTAATCTGTGGAAAGTTTGGCGTGAAACCATTTGTGGTGAAGC
  ATTTCGTTGTGTTATTGCAGAAGCACAGCTGGACCCTGCAACCCTGACCCAGCTGAAAGATCAGTTTATGGAACGTCGTCGTGAG
  ATGCCGAAAAAACTGGTTGAAAATGCCATTAGCAATGGTGAACTGCCGAAAGATACCAATCGTGAACTGCTGCTGGATATGATTT
  TTGGTTTTTGTTGGTATCGCCTGCTGACCGAACAGCTGACCGTTGAACAGGATATTGAAGAATTTACCTTCCTGCTGATTAATGGTG
  TTTGTCCGGGTACACAGCGTTAA</sbol:elements>
  <sbol:encoding rdf:resource="http://www.chem.qmul.ac.uk/iubmb/misc/naseq.html"/>
</sbol:Sequence>
<sbol:CombinatorialDerivation rdf:about="http://async.ece.utah.edu/Promoter_Derivation/1">
  <sbol:persistentIdentity rdf:resource="http://async.ece.utah.edu/Promoter_Derivation"/>
  <sbol:displayId>Promoter_Derivation</sbol:displayId>
  <sbol:version>1</sbol:version>
  <sbol:template rdf:resource="http://async.ece.utah.edu/Derivation_example/1"/>
  <sbol:strategy rdf:resource="http://sbols.org/v2#enumerate"/>
  <sbol:variableComponent>
    <sbol:VariableComponent rdf:about="http://async.ece.utah.edu/Promoter_Derivation/Pro_Component_VariableComponent/1">
      <sbol:persistentIdentity rdf:resource="http://async.ece.utah.edu/Promoter_Derivation/Pro_Component_VariableComponent"/>
      <sbol:displayId>Pro_Component_VariableComponent</sbol:displayId>
      <sbol:version>1</sbol:version>
      <sbol:variable rdf:resource="http://async.ece.utah.edu/Derivation_example/Pro_Component/1"/>
      <sbol:operator rdf:resource="http://sbols.org/v2#one"/>
      <sbol:variant rdf:resource="https://synbiohub.programmingbiology.org/public/Cello_Parts/pBetI/1"/>
      <sbol:variant rdf:resource="https://synbiohub.programmingbiology.org/public/Cello_Parts/pAmtR/1"/>
    </sbol:VariableComponent>
  </sbol:variableComponent>
  <sbol:variableComponent>
    <sbol:VariableComponent rdf:about="http://async.ece.utah.edu/Promoter_Derivation/Gen_Component_VariableComponent/1">
      <sbol:persistentIdentity rdf:resource="http://async.ece.utah.edu/Promoter_Derivation/Gen_Component_VariableComponent"/>
      <sbol:displayId>Gen_Component_VariableComponent</sbol:displayId>
      <sbol:version>1</sbol:version>
      <sbol:variable rdf:resource="http://async.ece.utah.edu/Derivation_example/Gen_Component/1"/>
      <sbol:operator rdf:resource="http://sbols.org/v2#one"/>
      <sbol:variantDerivation rdf:resource="http://async.ece.utah.edu/Terminatior_Derivation/1"/>
    </sbol:VariableComponent>
  </sbol:variableComponent>
</sbol:CombinatorialDerivation>
<sbol:CombinatorialDerivation rdf:about="http://async.ece.utah.edu/Terminatior_Derivation/1">
  <sbol:persistentIdentity rdf:resource="http://async.ece.utah.edu/Terminatior_Derivation"/>
  <sbol:displayId>Terminatior_Derivation</sbol:displayId>
  <sbol:version>1</sbol:version>
  <sbol:template rdf:resource="http://async.ece.utah.edu/Gen/1"/>
  <sbol:variableComponent>
    <sbol:VariableComponent rdf:about="http://async.ece.utah.edu/Terminatior_Derivation/Ter_Component_VariableComponent/1">
      <sbol:persistentIdentity rdf:resource="http://async.ece.utah.edu/Terminatior_Derivation/Ter_Component_VariableComponent"/>

<sbol:displayId>Ter_Component_VariableComponent</sbol:displayId>
```

```
        <sbol:version>1</sbol:version>
        <sbol:variable rdf:resource="http://async.ece.utah.edu/Gen/Ter_Component/1"/>
        <sbol:operator rdf:resource="http://sbols.org/v2#one"/>
        <sbol:variant rdf:resource="https://synbiohub.programmingbiology.org/public/Cello_Parts/L3S3P31/1"/>
        <sbol:variant rdf:resource="https://synbiohub.programmingbiology.org/public/Cello_Parts/L3S2P55/1"/>
      </sbol:VariableComponent>
    </sbol:variableComponent>
  </sbol:CombinatorialDerivation>
</rdf:RDF>
```

2.3.0

### 13.2 Adding Measures/Parameters with OM

[New in 2.3.0; see SEP 028: https://github.com/SynBioDex/SEPs/blob/master/sep_028.md]

There are at least two well-established cases for including measures/parameters and their associated units in SBOL design specifications. These use cases are the specification of genetic circuit designs and their associated parameters (such as rates of transcription) and the specification of environmental conditions for biological system designs (such as growth media concentrations and temperatures). In the first use case, parameters are necessary to enable the generation of quantitive models of circuit behavior from circuit design specifications. In the second use case, measures are necessary to define experimental conditions and enable the analysis of system behavior or characterization with respect to environmental context.

The Ontology of Units of Measure (OM) (http://www.ontology-of-units-of-measure.org/resource/om-2) already defines a data model for representing measures and their associated units. Here, a subset of OM is adopted by SBOL to describe these concepts for biological design specifications. As shown in Figure 37, SBOL leverages three of the base classes defined by the OM: Measure, Unit and Prefix. A Measure links a numerical value to a Unit, which may or may not have a Prefix (e.g. centi, milli, micro, etc.). As these classes are adopted by SBOL, Measure is treated as a subclass of Identified, while Unit and Prefix are treated as subclasses of TopLevel. In addition, SBOL adopts the following OM Unit subclasses: SingularUnit, CompoundUnit, UnitMultiplication, UnitDivision, UnitExponentiation, and PrefixedUnit. Lastly, SBOL adopts the following Prefix subclasses from OM: SIPrefix and BinaryPrefix.

SBOL-compliant tools are allowed to read, write, and modify data belonging to OM classes other than those described here, but this specification does not provide any guidance for the interpretation or use of these data in the context of SBOL.

2.3.0

#### 13.2.1 Measure

The purpose of the Measure class is to link a numerical value to a Unit.

##### The hasNumericalValue property

The hasNumericalValue property is REQUIRED and MUST contain a single xsd:float.

##### The hasUnit property

The hasUnit property is REQUIRED and MUST contain a URI that refers to a Unit. The OM provides URIs for many existing instances of the Unit class for reference (for example, http://www.ontology-of-units-of-measure.org/resource/om-2/gramPerLitre).

##### The types property

The types property is OPTIONAL and MAY contain a set of URIs. It is RECOMMENDED that one of these URIs identify a term from the Systems Biology Ontology (SBO) (http://www.ebi.ac.uk/sbo/main/). This types property of the Measure class is not specified in the OM and is added by SBOL to describe different types of parameters (for example, rate of reaction is identified by the SBO term http://identifiers.org/biomodels.sbo/SBO:0000612).

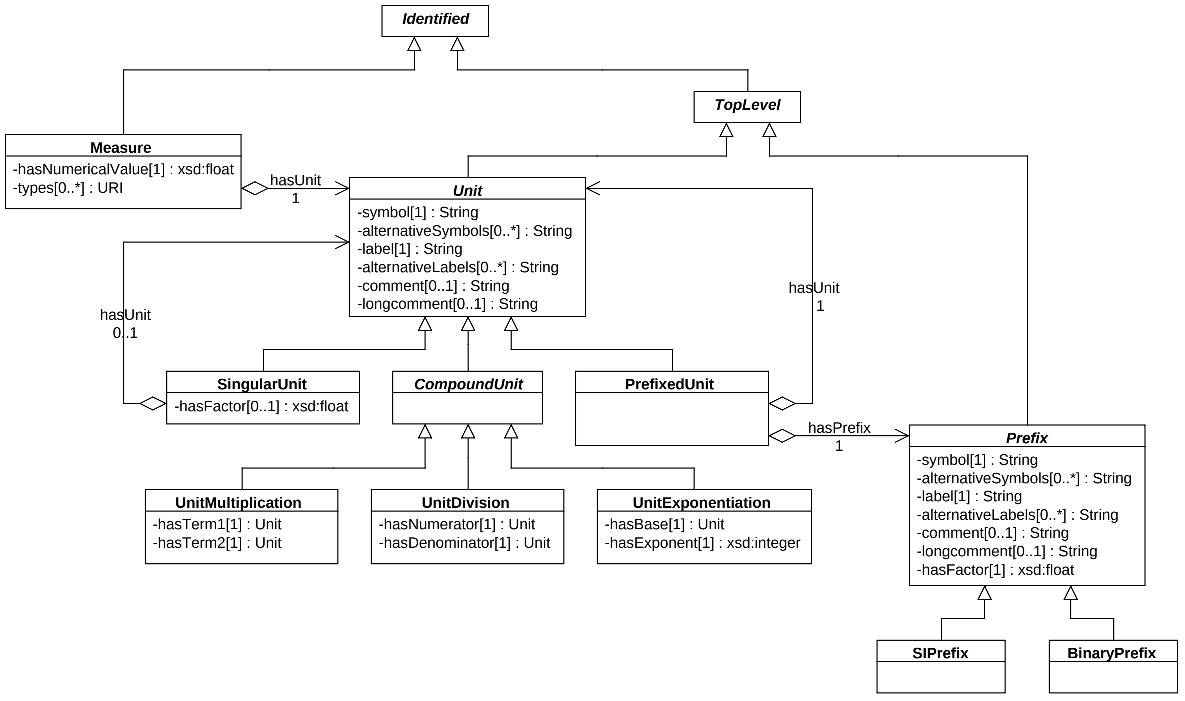

***Figure 37:*** *OM classes adopted by SBOL and their subclass relationships to* `Identified` *and* `TopLevel`

#### *Serialization*

The serialization of a `Measure` MUST have the following form:

```
<om:Measure rdf:about="...">
  ... properties inherited from Identified ...
  one   <om:hasNumericalValue>...</om:hasNumericalValue> element
  one   <om:hasUnit rdf:resource="..."/> element
  zero or more  <sbol:type rdf:resource="..."/> element
</om:Measure>
```

2.3.0 The example below shows the serialization of a `Measure` that describes the Michaelis constant for an enzymatic reaction.

```
<?xml version="1.0" ?>
<rdf:RDF xmlns:rdf="http://www.w3.org/1999/02/22-rdf-syntax-ns#" xmlns:dcterms="http://purl.org/dc/terms/" xmlns:om="http://www.
      ontology-of-units-of-measure.org/resource/om-2/" xmlns:xsd="http://www.w3.org/2001/XMLSchema#" xmlns:sbol="http://sbols.org/
      v2#">
  <om:Measure rdf:about="http://sbolstandard.org/example/ribonuclease_michaelis_constant/1">
    <sbol:persistentIdentity rdf:resource="http://sbolstandard.org/example/ribonuclease_michaelis_constant"/>
    <sbol:displayId>ribonuclease_michaelis_constant</sbol:displayId>
    <dcterms:title>Ribonuclease Michaelis Constant</dcterms:title>
    <om:hasNumericalValue>"7.9"^^xsd:float<om:hasNumericalValue/>
    <om:hasUnit rdf:resource="http://www.ontology-of-units-of-measure.org/resource/om-2/millimolair"/>
    <sbol:type rdf:resource="http://identifiers.org/biomodels.sbo/SBO:0000027"/>
  </om:Measure>
</rdf:RDF>
```

2.3.0

#### 13.2.2 Unit

As adopted by SBOL, Unit is an abstract class that is extended by other classes to describe units of measure using a shared set of properties.

##### The symbol property

The symbol property is REQUIRED and MUST contain a String. This String is commonly used to abbreviate the unit of measure's name. For example, the unit of measure named "gram per liter" is commonly abbreviated using the String "g/l".

##### The alternativeSymbols property

The alternativeSymbols property is OPTIONAL and MAY contain a set of Strings. This property can be used to specify alternative abbreviations other than that specified using the symbol property.

##### The label property

The label property is REQUIRED and MUST contain a String. This String is a common name for the unit of measure and SHOULD be identical to any String contained by the name property inherited from Identified.

##### The alternativeLabels property

The alternativeLabels property is OPTIONAL and MAY contain a set of Strings. This property can be used to specify alternative common names other than that specified using the label property.

##### The comment property

The comment property is OPTIONAL and MAY contain a String. This String is a description of the unit of measure and SHOULD be identical to any String contained by the description property inherited from Identified.

##### The longcomment property

The longcomment property is OPTIONAL and MAY contain a String. This String is a long description of the unit of measure and SHOULD be longer than any String contained by the comment property.

2.3.0

#### 13.2.3 SingularUnit

The purpose of the SingularUnit class is to describe a unit of measure that is not explicitly represented as a combination of multiple units, but could be equivalent to such a representation. For example, a joule is considered to be a SingularUnit, but it is equivalent to the multiplication of a newton and a meter.

##### The hasUnit property

The hasUnit is OPTIONAL and MAY contain a URI. This URI MUST refer to another Unit. The hasUnit propery can be used in conjunction with the hasFactor property to specify whether a SingularUnit is equivalent to another Unit multiplied by a factor. For example, an angstrom is equivalent to $10^{-10}$ meters.

##### The hasFactor property

The hasFactor property is OPTIONAL and MAY contain a xsd:float. If the hasFactor property of a SingularUnit is non-empty, then its hasUnit property SHOULD also be non-empty.

##### Serialization

The serialization of a SingularUnit MUST have the following form:

```
<om:SingularUnit rdf:about="...">
  ... properties inherited from TopLevel ...
  one  <om:symbol>...<om:symbol/> element
```

```
  zero or more  <om:alternativeSymbol>...<om:alternativeSymbol/> element
  one  <rdfs:label>...<rdfs:label/> element
  zero or more  <om:alternativeLabel>...<om:alternativeLabel/> element
  zero or one  <rdfs:comment>...<rdfs:comment/> element
  zero or one  <om:longcomment>...<om:longcomment/> element
  zero or one  <om:hasUnit rdf:resource="..."/> element
  zero or one  <om:hasFactor rdf:resource="..."/> element
</om:SingularUnit>
```

2.3.0 The example below shows the serialization of a SingularUnit that describes a genetic unit of length not present in the OM: the base pair (bp). One bp is equivalent to 3.4 angstroms.

```
<?xml version="1.0" ?>
<rdf:RDF xmlns:rdf="http://www.w3.org/1999/02/22-rdf-syntax-ns#" xmlns:rdfs="http://www.w3.org/2000/01/rdf-schema#" xmlns:dcterms
      ="http://purl.org/dc/terms/" xmlns:om="http://www.ontology-of-units-of-measure.org/resource/om-2/" xmlns:xsd="http://www.w3.
      org/2001/XMLSchema#" xmlns:sbol="http://sbols.org/v2#">
  <om:SingularUnit rdf:about="http://sbolstandard.org/example/base_pair/1">
    <sbol:persistentIdentity rdf:resource="http://sbolstandard.org/example/base_pair"/>
    <sbol:displayId>base_pair</sbol:displayId>
    <dcterms:title>Base Pair</dcterms:title>
    <om:symbol>bp</om:symbol>
    <rdfs:label xml:lang="en">Base Pair</rdfs:label>
    <om:hasUnit rdf:resource="http://www.ontology-of-units-of-measure.org/resource/om-2/angstrom"/>
    <om:hasFactor rdf:datatype="&xsd;float">3.4</om:hasFactor>
  </om:SingularUnit>
</rdf:RDF>
```

2.3.0

#### 13.2.4 CompoundUnit

As adopted by SBOL, CompoundUnit is an abstract class that is extended by other classes to describe units of measure that can be represented as combinations of multiple other units of measure.

2.3.0

#### 13.2.5 UnitMultiplication

The purpose of the UnitMultiplication class is to describe a unit of measure that is the multiplication of two other units of measure.

##### The hasTerm1 property

The hasTerm1 property is REQUIRED and MUST contain a URI that refers to another Unit. This Unit is the first multiplication term.

##### The hasTerm2 property

The hasTerm2 property is REQUIRED and MUST contain a URI that refers to another Unit. This Unit is the second multiplication term. It is okay if the Unit referred to by hasTerm1 is the same as that referred to by hasTerm2.

##### Serialization

The serialization of a UnitMultiplication MUST have the following form:

```
<om:UnitMultiplication rdf:about="...">
  ... properties inherited from TopLevel ...
  one  <om:symbol>...<om:symbol/> element
  zero or more  <om:alternativeSymbol>...<om:alternativeSymbol/> element
  one  <rdfs:label>...<rdfs:label/> element
  zero or more  <om:alternativeLabel>...<om:alternativeLabel/> element
  zero or one  <rdfs:comment>...<rdfs:comment/> element
  zero or one  <om:longcomment>...<om:longcomment/> element
  one  <om:hasTerm1 rdf:resource="..."/> element
  one  <om:hasTerm2 rdf:resource="..."/> element
</om:UnitMultiplication>
```

2.3.0

#### 13.2.6 UnitDivision

The purpose of the UnitDivision class is to describe a unit of measure that is the division of one unit of measure by another.

##### The hasNumerator property

The hasNumerator property is REQUIRED and MUST contain a URI that refers to another Unit.

##### The hasDenominator property

The hasDenominator property is REQUIRED and MUST contain a URI that refers to another Unit.

##### Serialization

The serialization of a UnitDivision MUST have the following form:

```
<om:UnitDivision rdf:about="...">
  ... properties inherited from TopLevel ...
  one  <om:symbol>...<om:symbol/> element
  zero or more  <om:alternativeSymbol>...<om:alternativeSymbol/> element
  one  <rdfs:label>...<rdfs:label/> element
  zero or more  <om:alternativeLabel>...<om:alternativeLabel/> element
  zero or one  <rdfs:comment>...<rdfs:comment/> element
  zero or one  <om:longcomment>...<om:longcomment/> element
  one  <om:hasNumerator rdf:resource="..."/> element
  one  <om:hasDenominator rdf:resource="..."/> element
</om:UnitDivision>
```

2.3.0 The example below shows the serialization of a UnitDivision that describes a unit of cell density not present in the OM: colony forming unit (CFU) per centimeter squared.

```
<?xml version="1.0" ?>
<rdf:RDF xmlns:rdf="http://www.w3.org/1999/02/22-rdf-syntax-ns#" xmlns:rdfs="http://www.w3.org/2000/01/rdf-schema#" xmlns:dcterms
     ="http://purl.org/dc/terms/" xmlns:om="http://www.ontology-of-units-of-measure.org/resource/om-2/" xmlns:sbol="http://sbols.
     org/v2#">
  <om:UnitDivision rdf:about="http://sbolstandard.org/example/cfu_per_centimeter_squared/1">
    <sbol:persistentIdentity rdf:resource="http://sbolstandard.org/example/cfu_per_centimeter_squared"/>
    <sbol:displayId>cfu_per_centimeter_squared</sbol:displayId>
    <dcterms:title>CFU per Centimeter Squared</dcterms:title>
    <om:symbol>CFU/cm2</om:symbol>
    <rdfs:label xml:lang="en">CFU per Centimeter Squared</rdfs:label>
    <om:hasNumerator rdf:resource="http://www.ontology-of-units-of-measure.org/resource/om-2/colonyFormingUnit"/>
    <om:hasDenominator rdf:resource="http://www.ontology-of-units-of-measure.org/resource/om-2/colonyFormingUnitsquareCentimetre"
     />
  </om:UnitDivision>
</rdf:RDF>
```

2.3.0

#### 13.2.7 UnitExponentiation

The purpose of the UnitExponentiation class is to describe a unit of measure that is raised to an integer power.

##### The hasBase property

The hasBase property is REQUIRED and MUST contain a URI that refers to another Unit.

##### The hasExponent property

The hasExponent property is REQUIRED and MUST contain an xsd:integer.

##### Serialization

The serialization of a UnitExponentiation MUST have the following form:

```
<om:UnitExponentiation rdf:about="...">
  ... properties inherited from TopLevel ...
  one  <om:symbol>...<om:symbol/> element
  zero or more  <om:alternativeSymbol>...<om:alternativeSymbol/> element
  one  <rdfs:label>...<rdfs:label/> element
  zero or more  <om:alternativeLabel>...<om:alternativeLabel/> element
  zero or one  <rdfs:comment>...<rdfs:comment/> element
  zero or one  <om:longcomment>...<om:longcomment/> element
  one  <om:hasBase rdf:resource="..."/> element
  one  <om:hasExponent>...<om:hasExponent/> element
</om:UnitExponentiation>
```

2.3.0

#### 13.2.8 PrefixedUnit

The purpose of the `PrefixedUnit` class is to describe a unit of measure that is the multiplication of another unit of measure and a factor represented by a standard prefix such as “milli,” “centi,” “kilo,” etc.

##### The `hasUnit` property

The `hasUnit` property is REQUIRED and MUST contain a `URI` that refers to another `Unit`.

##### The `hasPrefix` property

The `hasPrefix` property is REQUIRED and MUST contain a `URI` that refers to a `Prefix`.

##### Serialization

The serialization of a `PrefixedUnit` MUST have the following form:

```
<om:PrefixedUnit rdf:about="...">
  ... properties inherited from TopLevel ...
  one  <om:symbol>...<om:symbol/> element
  zero or more  <om:alternativeSymbol>...<om:alternativeSymbol/> element
  one  <rdfs:label>...<rdfs:label/> element
  zero or more  <om:alternativeLabel>...<om:alternativeLabel/> element
  zero or one  <rdfs:comment>...<rdfs:comment/> element
  zero or one  <om:longcomment>...<om:longcomment/> element
  one  <om:hasUnit rdf:resource="..."/> element
  one  <om:hasPrefix rdf:resource="..."/> element
</om:PrefixedUnit>
```

2.3.0 The example below shows the serialization of a `PrefixedUnit` that describes an absolute unit of fluorescence not present in the OM: the kilo-Molecules of Equivalent Fluorescein (kilo-MEFL).

```
<?xml version="1.0" ?>
<rdf:RDF xmlns:rdf="http://www.w3.org/1999/02/22-rdf-syntax-ns#" xmlns:rdfs="http://www.w3.org/2000/01/rdf-schema#" xmlns:dcterms
     ="http://purl.org/dc/terms/" xmlns:om="http://www.ontology-of-units-of-measure.org/resource/om-2/" xmlns:sbol="http://sbols.
     org/v2#">
  <om:PrefixedUnit rdf:about="http://sbolstandard.org/example/kiloMEFL/1">
    <sbol:persistentIdentity rdf:resource="http://sbolstandard.org/example/kiloMEFL"/>
    <sbol:displayId>kiloMEFL</sbol:displayId>
    <dcterms:title>kilo-Molecules of Equivalent Fluorescein</dcterms:title>
    <om:symbol>kMEFL</om:symbol>
    <rdfs:label xml:lang="en">kilo-Molecules of Equivalent Fluorescein</rdfs:label>
    <om:hasUnit rdf:resource="http://sbolstandard.org/example/MEFL/1"/>
    <om:hasPrefix rdf:resource="http://www.ontology-of-units-of-measure.org/resource/om-2/kilo"/>
  </om:PrefixedUnit>
</rdf:RDF>
```

2.3.0

#### 13.2.9 Prefix

As adopted by SBOL, Prefix is an abstract class that is extended by other classes to describe factors that are commonly represented by standard unit prefixes. For example, the factor $10^{-3}$ is represented by the standard unit prefix "milli."

##### The symbol property

The symbol property is REQUIRED and MUST contain a String. This String is commonly used to abbreviate the name of the unit prefix. For example, the String "m" is commonly used to abbreviate the name "milli."

##### The alternativeSymbols property

The alternativeSymbols property is OPTIONAL and MAY contain a set of Strings. This property can be used to specify alternative abbreviations other than that specified using the symbol property.

##### The label property

The label property is REQUIRED and MUST contain a String. This String is a common name for the unit prefix and SHOULD be identical to any String contained by the name property inherited from Identified.

##### The alternativeLabels property

The alternativeLabels property is OPTIONAL and MAY contain a set of Strings. This property can be used to specify alternative common names other than that specified using the label property.

##### The comment property

The comment property is OPTIONAL and MAY contain a String. This String is a description of the unit prefix and SHOULD be identical to any String contained by the description property inherited from Identified.

##### The longcomment property

The longcomment property is OPTIONAL and MAY contain a String. This String is a long description of the unit of measure and SHOULD be longer than any String contained by the comment property.

##### The hasFactor property

The hasFactor property is REQUIRED and MUST contain an xsd:float.

2.3.0

#### 13.2.10 SIPrefix

The purpose of the SIPrefix class is to describe standard SI prefixes such as "milli," "centi," "kilo," etc.

##### Serialization

The serialization of a SIPrefix MUST have the following form:

```
<om:SIPrefix rdf:about="...">
  ... properties inherited from TopLevel ...
  one  <om:symbol>...<om:symbol/> element
  zero or more  <om:alternativeSymbol>...<om:alternativeSymbol/> element
  one  <rdfs:label>...<rdfs:label/> element
  zero or more  <om:alternativeLabel>...<om:alternativeLabel/> element
  zero or one  <rdfs:comment>...<rdfs:comment/> element
  zero or one  <om:longcomment>...<om:longcomment/> element
  one  <om:hasFactor rdf:resource="..."/> element
</om:SIPrefix>
```

2.3.0

##### 13.2.11 BinaryPrefix

The purpose of the `BinaryPrefix` class is to describe standard binary prefixes such as “kibi,” “mebi,” “gibi,” etc. These prefixes commonly precede units of information such as “bit” and “byte.”

###### Serialization

The serialization of a `BinaryPrefix` MUST have the following form:

```
<om:BinaryPrefix rdf:about="...">
  ... properties inherited from TopLevel ...
  one  <om:symbol>...<om:symbol/> element
  zero or more  <om:alternativeSymbol>...<om:alternativeSymbol/> element
  one  <rdfs:label>...<rdfs:label/> element
  zero or more  <om:alternativeLabel>...<om:alternativeLabel/> element
  zero or one  <rdfs:comment>...<rdfs:comment/> element
  zero or one  <om:longcomment>...<om:longcomment/> element
  one  <om:hasFactor rdf:resource="..."/> element
</om:BinaryPrefix>
```

###### Example - Growth Media Recipe

2.3.0 Figure 38 shows an example of using instances of the `Measure` and `Unit` classes in conjunction with instances of the SBOL `ModuleDefinition` and `ComponentDefinition` classes to specify a growth media recipe. Note that this specification for M9 Glucose CAA media is partly composed from the specification of another growth media, Teknova M1902. Namespace abbreviations used in this example include om for `http://www.ontology-of-units-of-measure.org/resource/om-2/`, obo for `http://purl.obolibrary.org/obo/`, chebi for `http://identifiers.org/chebi/`, and sbo for `http://identifiers.org/biomodels.sbo/`. For specifying `ModuleDefinition roles`, MC85504 codes for the term "growth medium" in the National Cancer Institute Thesaurus (NCIT). For specifying `Measure types` with the SBO, 0000196 codes for “concentration of an entity pool”, 0000226 codes for “density of an entity pool”, and 0000470 codes for “mass fraction.”

2.3.0

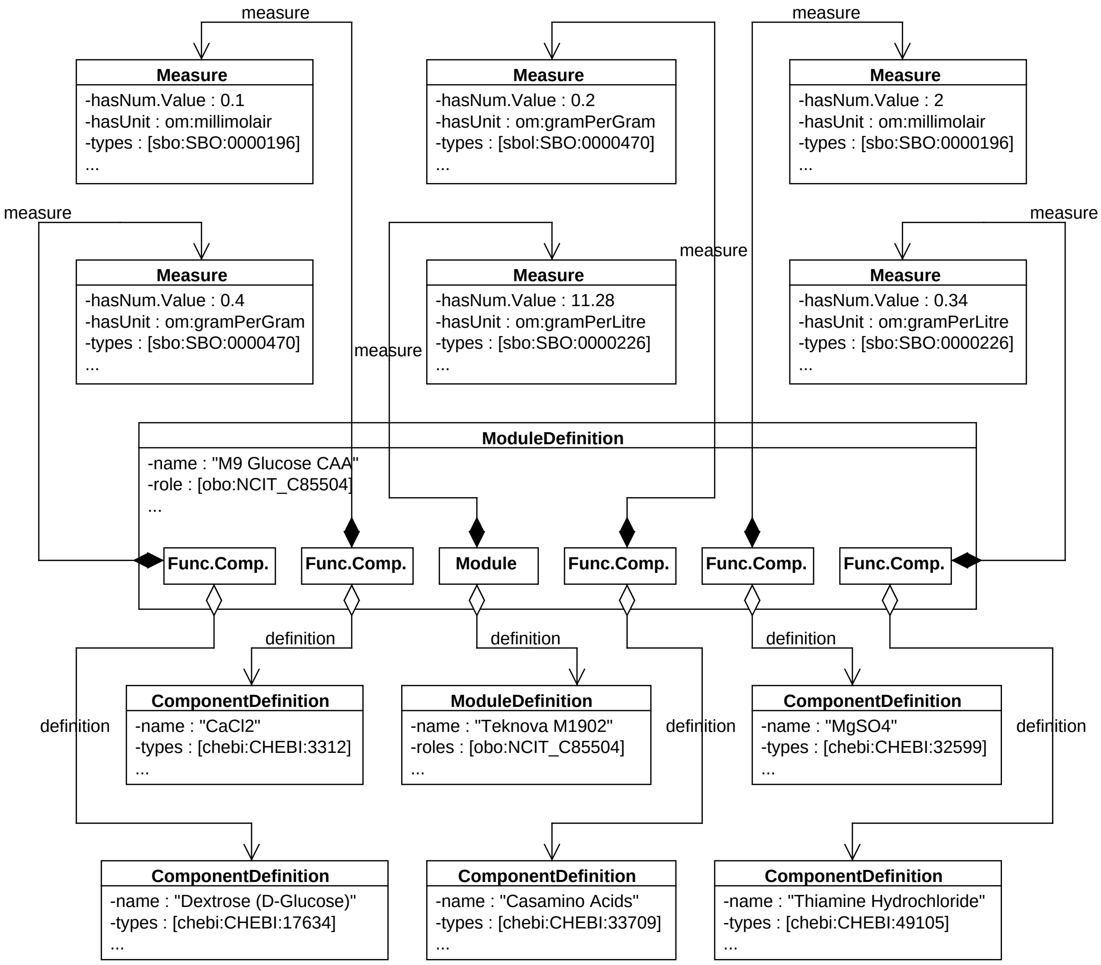

***Figure 38:*** *Growth media recipe represented using instances of the* `Measure` *and* `Unit` *classes from the OM.*

## A Validation Rules

This section summarizes all the conditions that either MUST be or are RECOMMENDED to be true of an SBOL Version 2.3 document. There are different degrees of rule strictness. Rules of the former kind are strict SBOL validation rules—data encoded in SBOL MUST conform to all of them in order to be considered valid. Rules of the latter kind are consistency rules that SBOL data are RECOMMENDED to adhere to as a best practice. To help highlight these differences, we use the following symbols next to the rule numbers:

2.0.1

- ☑ A checked box indicates a strong REQUIRED condition for SBOL conformance. If a SBOL document does not follow this rule, it does not conform to the SBOL specification.
- ● A circle indicates a weak REQUIRED condition for SBOL conformance. While this rule MUST be followed, there are conditions under which it cannot be checked by a machine.
- ★ A star indicates a RECOMMENDED condition for following best practices. This rule is not strictly a matter of SBOL conformance, but its recommendation comes from logical reasoning. If an SBOL document does not follow this rule, it is still valid SBOL, but it might have degraded functionality in some tools.

We also include a fourth type of rule that represents a required condition for SBOL-compliance that cannot be checked by a machine. Therefore, violations of these rules are not expected to be reported as errors by any of the software libraries implementing SBOL 2.1 or above. It is the user's responsibility to make sure that these validation rules are followed.

- ▲ A triangle indicates a weak REQUIRED condition for SBOL conformance. While this rule MUST be followed, it is not possible in practice for a machine to automatically check whether the rule has been followed.

The validation rules listed in the following subsections are all believed to be stated or implied in the rest of this specification document. They are enumerated here for convenience and to provide a "master checklist" for SBOL validation. In case of a conflict between this section and other portions of the specification (though there are believed to be none), this section is considered authoritative for the purpose of determining the validity of an SBOL document.

☞ For convenience and brevity, we use the shorthand "`sbol:x`" to stand for an attribute or element name `x` in the namespace for the SBOL specification, using the namespace prefix `sbol`. In reality, the prefix string can be different from the literal "`sbol`" used here (and indeed, it can be any valid XML namespace prefix that the software chooses). We use "`sbol:x`" because it is shorter than to write a full explanation everywhere we refer to an attribute or element in the SBOL specification namespace.

#### *General rules for an SBOL document*

**sbol-10101** ☑ An SBOL document MUST declare the use of the following XML namespace: "`http://sbols.org/v2#`".
Reference: Section 10 on page 67

2.0.1

**sbol-10102** ☑ ~~An SBOL document MUST declare the use of the following XML namespace: `http://www.w3.org/1999/02/22-rdf-syntax-ns#`.~~
~~Reference: Section 10 on page 54~~

2.0.1

**sbol-10103** ☑ ~~If an SBOL document includes any name or description properties, then it MUST declare the use of the following XML namespace: `http://purl.org/dc/terms/`.~~
~~Reference: Section 10 on page 54~~

2.0.1

**sbol-10104** ☑ ~~If an SBOL document includes any wasDerivedFrom properties, then it MUST declare the use of the following XML namespace: `http://www.w3.org/ns/prov#`. Reference: Section 10 on page 54~~

2.0.1 **sbol-10105** ☑ An SBOL document MUST be serialized as a well-formed RDF/XML file that adheres to the grammar defined by:
"`https://www.w3.org/TR/rdf-syntax-grammar/`".
Reference: Section 10 on page 67

2.2.1 **sbol-10106** ☑ All namespaces specified in an SBOL document MUST end with a delimiter ('/', '#', or ':').
Reference: Section 10 on page 67

#### *Rules for the Identified class*

**sbol-10201** ☑ The `identity` property of an `Identified` object is REQUIRED and MUST contain a `URI` that adheres to the syntax defined by:
"`http://www.w3.org/TR/xmlschema11-2/#anyURI`"
Reference: Section 7.4 on page 17

2.0.1 **sbol-10202** ● The `identity` property of an `Identified` object MUST be globally unique.
Reference: Section 7.4 on page 17

**sbol-10203** ☑ The `persistentIdentity` property of an `Identified` object is OPTIONAL and MAY contain a `URI` that MUST adhere to the syntax defined by:
"`http://www.w3.org/TR/xmlschema11-2/#anyURI`"
Reference: Section 7.4 on page 17

**sbol-10204** ☑ The `displayId` property of an `Identified` object is OPTIONAL and MAY contain a `String` that MUST be composed of only alphanumeric or underscore characters and MUST NOT begin with a digit.
Reference: Section 7.4 on page 17

2.0.1 **sbol-10205** ★ ~~The displayId property of an Identified object SHOULD be locally unique. Reference: Section 7.4 on page 16~~

**sbol-10206** ☑ The `version` property of an `Identified` object is OPTIONAL and MAY contain a `String` that MUST be composed of only alphanumeric characters, underscores, hyphens, or periods and MUST begin with a digit.
Reference: Section 7.4 on page 17

2.0.1 **sbol-10207** ★ (moved to sbol-10302)

2.2.0 **sbol-10208** ☑ The `wasDerivedFroms` property of an `Identified` object is OPTIONAL and MAY contain a set of `URI`s.
Reference: Section 7.4 on page 17

2.0.1 **sbol-10209** ☑ (moved to sbol-10303)

**sbol-10210** ● (moved to sbol-10304)

**sbol-10211** ▲ (moved to sbol-10305)

**sbol-10212** ☑ The name property of an Identified object is OPTIONAL and MAY contain a String.
Reference: Section 7.4 on page 17

**sbol-10213** ☑ The description property of an Identified object is OPTIONAL and MAY contain a String.
Reference: Section 7.4 on page 17

2.0.1 **sbol-10214** ▲ The annotations property of an Identified object is OPTIONAL and MAY contain a set of Annotation objects.
Reference: Section 7.4 on page 17

**sbol-10215** ★ The displayId property of a compliant Identified object is REQUIRED.
Reference: Section 12.3 on page 70

**sbol-10216** ★ The persistentIdentity property of a compliant TopLevel object is REQUIRED and MUST contain a URI that ends with a delimiter ('/', '#', or ':') followed by the displayId of the TopLevel object.
Reference: Section 12.3 on page 70

**sbol-10217** ★ The persistentIdentity property of a compliant Identified object that is not also a TopLevel object is REQUIRED and MUST contain a URI that begins with the persistentIdentity of the compliant object's parent and is immediately followed by a delimiter ('/', '#', or ':') and the displayId of the compliant object.
Reference: Section 12.3 on page 70

**sbol-10218** ★ If a compliant Identified object has no version property, then its identity property MUST contain the same URI as its persistentIdentity property. Otherwise, the compliant object's identity property MUST contain a URI that begins with its persistentIdentity and is immediately followed by a delimiter ('/', '#', or ':') and its version.
Reference: Section 12.3 on page 70

**sbol-10219** ★ The version property of a compliant Identified object that is not also a TopLevel object is REQUIRED to contain the same String as the version property of the compliant object's parent.
Reference: Section 12.3 on page 70

2.0.1 **sbol-10220** ☑ Objects with the same persistentIdentity MUST be instances of the same class.
Reference: Section 7.4 on page 17

2.2.0 **sbol-10221** ☑ The wasGeneratedBys property of an Identified object is OPTIONAL and MAY contain a set of URIs.
Reference: Section 13.1 on page 73

2.2.0 **sbol-10222** ● Each URI contained by the wasGeneratedBys property of an Identified object MUST refer to an Activity object.
Reference: Section 13.1 on page 73

2.2.1 **sbol-10223** ● Provenance history formed by wasGeneratedBys properties of Identified objects and entity references in Usage objects MUST NOT form circular reference chains.
Reference: Section 13.1 on page 73

2.2.0 **sbol-10224** ★ An Identified with a wasGeneratedBys property that includes a reference to an Activity with a child Association that has a roles property that contains the URI http://sbols.org/v2#design SHOULD be a TopLevel object other than Implementation.
Reference: Section 13.1 on page 73

**sbol-10225** ★ An Identified with a wasGeneratedBys property that includes a reference to an Activity with a child Association that has a roles property that contains the URI http://sbols.org/v2#build SHOULD be an Implementation.
Reference: Section 13.1 on page 73

**sbol-10226** ★ An Identified with a wasGeneratedBys property that includes a reference to an Activity with a child Association that has a roles property that contains the URI http://sbols.org/v2#test SHOULD be an ExperimentalData.
Reference: Section 13.1 on page 73

**sbol-10227** ★ An Identified with a wasGeneratedBys property that includes a reference to an Activity with a child Association that has a roles property that contains the URI http://sbols.org/v2#learn SHOULD not be an Implementation.
Reference: Section 13.1 on page 73

2.2.1 **sbol-10228** ☑ An Identified object must have no more than one rdfType property in each of the "http://sbols.org/v2#" and "http://www.w3.org/ns/prov#" namespaces.
Reference: Section 7.4 on page 17

#### Rules for the TopLevel class

2.0.1 **sbol-10301** ☑ ~~A TopLevel object MUST inherit all properties of the Identified class.~~
~~Reference: Section 7.5 on page 19~~

**sbol-10302** ★ If the wasDerivedFroms property of one TopLevel object refers to another TopLevel object with the same persistentIdentity property, then the version property of the second TopLevel object SHOULD precede that of the first if both objects have a version and it is assumed that these versions follow the conventions of semantic versioning.
2.2.0
Reference: Section 7.4 on page 17

**sbol-10303** ☑ The wasDerivedFroms property of an TopLevel object MUST NOT contain a URI reference to the TopLevel object itself.
2.2.0
Reference: Section 7.4 on page 17

**sbol-10304** ● TopLevel objects MUST NOT form circular reference chains via their wasDerivedFroms properties.
2.2.0
Reference: Section 7.4 on page 17

**sbol-10305** ▲ If the wasDerivedFroms property of one TopLevel object refers to another TopLevel object with the same persistentIdentity property, then version property of the second TopLevel object MUST precede that of the first if both objects have a version.
2.2.0
Reference: Section 7.4 on page 17

2.2.1 **sbol-10306** ☑ The attachments property of a TopLevel is OPTIONAL and MAY contain a set of URIs.
Reference: Section 7.5 on page 20

2.2.1 **sbol-10307** ● Each URI contained by the attachments property of a TopLevel MUST refer to an Attachment object.
Reference: Section 7.5 on page 20

#### Rules for the Sequence class

2.0.1 **sbol-10401** ☑ A Sequence MUST NOT have properties other than the following: identity, persistentIdentity, displayId, version, wasDerivedFroms, wasGeneratedBys , name, description, annotations, elements, and encoding.
2.2.0
Reference: Section 7.6 on page 21

**sbol-10402** ☑ The `elements` property of a `Sequence` is REQUIRED and MUST contain a `String`.
Reference: Section 7.6 on page 21

**sbol-10403** ☑ The `encoding` property of `Sequence` is REQUIRED and MUST contain a `URI`.
Reference: Section 7.6 on page 21

**sbol-10404** ▲ The `encoding` property of a `Sequence` MUST indicate how the `elements` property of the `Sequence` is to be formed and interpreted.
Reference: Section 7.6 on page 21

2.0.1 **sbol-10405** ● The `elements` property of a `Sequence` MUST be consistent with its `encoding` property.
Reference: Section 7.6 on page 21

**sbol-10406** ▲ The `encoding` property of a `Sequence` MUST contain a `URI` from Table 1 if it is well-described by this `URI`.
Reference: Section 7.6 on page 21

2.0.1 **sbol-10407** ★ The `encoding` property of a `Sequence` SHOULD contain a `URI` from Table 1.
Reference: Section 7.6 on page 21

#### *Rules for the ComponentDefinition class*

2.0.1 **sbol-10501** ☑ A `ComponentDefinition` MUST NOT have properties other than the following: `identity`, `persistentIdentity`, `displayId`, `version`, `wasDerivedFroms`, `wasGeneratedBys` , `name`,
2.2.0 `description`, `annotations`, `types`, `roles`, `components`, `sequences`, `sequenceAnnotations`, and `sequenceConstraints`.
Reference: Section 7.7 on page 22

**sbol-10502** ☑ The `types` property of a `ComponentDefinition` is REQUIRED and MUST contain a non-empty set of `URI`s.
Reference: Section 7.7 on page 22

**sbol-10503** ☑ The `types` property of a `ComponentDefinition` MUST NOT contain more than one `URI` from Table 2.
Reference: Section 7.7 on page 22

**sbol-10504** ▲ Each `URI` contained by the `types` property of a `ComponentDefinition` MUST refer to an ontology term that describes the category of biochemical or physical entity that is represented by the `ComponentDefinition`.
Reference: Section 7.7 on page 22

**sbol-10505** ▲ The `types` property of a `ComponentDefinition` MUST contain a `URI` from Table 2 if it is well-described by this `URI`.
Reference: Section 7.7 on page 22

**sbol-10506** ▲ All `URI`s contained by the `types` property of a `ComponentDefinition` MUST refer to non-conflicting ontology terms.
Reference: Section 7.7 on page 22

**sbol-10507** ☑ The `roles` property of a `ComponentDefinition` is OPTIONAL and MAY contain a set of `URI`s.
Reference: Section 7.7 on page 22

**sbol-10508** ▲ Each `URI` contained by the `roles` property of a `ComponentDefinition` MUST refer to an ontology term that clarifies the potential function of the `ComponentDefinition` in a biochemical or physical context.
Reference: Section 7.7 on page 22

**sbol-10509** ▲ Each URI contained by the roles property of a ComponentDefinition MUST refer to an ontology term that is consistent with its types property.
Reference: Section 7.7 on page 22

**sbol-10510** ▲ The roles property of a ComponentDefinition MUST contain a URI from Table 4 if it is well-described by this URI.
Reference: Section 7.7 on page 22

2.0.1 **sbol-10511** ★ The roles property of a ComponentDefinition SHOULD NOT contain a URI that refers to a term from the sequence feature branch of the SO unless its types property contains the DNA or RNA type URI listed in Table 2.
Reference: Section 7.7 on page 22

**sbol-10512** ☑ The sequences property of a ComponentDefinition is OPTIONAL and MAY contain a set of URIs.
Reference: Section 7.7 on page 22

2.0.1 **sbol-10513** ● Each URI contained by the sequences property of a ComponentDefinition MUST refer to a Sequence object.
Reference: Section 7.7 on page 22

**sbol-10514** ▲ The Sequence objects referred to by the sequences property of a ComponentDefinition MUST be consistent with each other, such that well-defined mappings exist between their elements properties in accordance with their encoding properties.
Reference: Section 7.7 on page 22

**sbol-10515** ▲ The sequences property of a ComponentDefinition MUST NOT refer to Sequence objects with conflicting encoding properties.
Reference: Section 7.7 on page 22

2.0.1 **sbol-10516** ● If the sequences property of a ComponentDefinition refers to one or more Sequence objects, and one of the types of this ComponentDefinition comes from Table 2, then one of the Sequence objects MUST have the encoding that is cross-listed with this type in Table 1.
Reference: Section 7.7 on page 22

**sbol-10517** ▲ ~~If the sequences property of a ComponentDefinition refers to a Sequence with an encoding from Table 1, then the types property of the ComponentDefinition MUST contain the type from Table 2 that is cross-listed with this encoding in Table 1.~~
~~Reference: Section 7.7 on page 21~~

**sbol-10518** ★ If a ComponentDefinition refers to more than one Sequence with the same encoding, then the elements of these Sequence objects SHOULD have equal lengths.
Reference: Section 7.7 on page 22

**sbol-10519** ☑ The components property of a ComponentDefinition is OPTIONAL and MAY contain a set of Component objects.
Reference: Section 7.7 on page 22

2.3.0 **sbol-10520** ★ If a ComponentDefinition in a ComponentDefinition-Component hierarchy refers to one or more Sequence objects, and there exist ComponentDefinition objects lower in the hierarchy that refer to Sequence objects with the same encoding, then the elements properties of these Sequence objects SHOULD be consistent with each other, such that well-defined mappings exist from the "lower level" elements to the "higher level" elements in accordance with their shared encoding properties. This mapping is subject to any restrictions on the positions of the Component objects in the hierarchy that are imposed by the SequenceAnnotation or SequenceConstraint objects contained by the ComponentDefinition objects in the hierarchy. This mapping is further subject to any restrictions on the sequences property of ComponentDefinition objects implied by the sourceLocations property on the Component objects owned by those ComponentDefinition objects.
Reference: Section 7.7 on page 22

**sbol-10521** ☑ The sequenceAnnotations property of a ComponentDefinition is OPTIONAL and MAY contain a set of SequenceAnnotation objects.
Reference: Section 7.7 on page 22

**sbol-10522** ☑ The sequenceAnnotations property of a ComponentDefinition MUST NOT contain two or more SequenceAnnotation objects that refer to the same Component.
Reference: Section 7.7 on page 22

**sbol-10523** ★ If the sequences property of a ComponentDefinition refers to a Sequence with an IUPAC encoding from Table 1, then each SequenceAnnotation that includes a Range and/or Cut in the sequenceAnnotations property of the ComponentDefinition SHOULD specify a region on the elements of this Sequence.
Reference: Section 7.7 on page 22

**sbol-10524** ☑ The sequenceConstraints property of a ComponentDefinition is OPTIONAL and MAY contain a set of SequenceConstraint objects.
Reference: Section 7.7 on page 22

2.0.1

**sbol-10525** ★ The types property of a ComponentDefinition SHOULD contain a URI from Table 2.
Reference: Section 7.7 on page 22

**sbol-10526** ☑ If multiple MapsTos belonging to the Components of a ComponentDefinition have local properties that refer to the same Component, then there MUST NOT be more than one such MapsTo that has a refinement property that contains the URI http://sbols.org/v2#useRemote.
Reference: Section 7.7.3 on page 30

**sbol-10527** ★ If the types property of a ComponentDefinition contains the DNA or RNA type URI, then its roles property SHOULD contain exactly one URI that refers to a term from the sequence feature branch of the SO.
Reference: Section 7.7 on page 22

2.1.0

**sbol-10528** ★ If the types property of a ComponentDefinition contains the DNA or RNA type URI, then its types property SHOULD also contain at most one URI that refers to a term from the topology attribute branch of the SO.
Reference: Section 7.7 on page 22

2.1.0

**sbol-10529** ★ The types property of a ComponentDefinition SHOULD NOT contain a URI that refers to a term from the topology attribute or strand attribute branches of the SO unless its types property contains the DNA or RNA type URI listed in Table 2. Reference: Section 7.7 on page 22

2.1.0

**sbol-10530** ▲ If the types property of a ComponentDefinition contains the DNA or RNA type URI, then its types property MUST contain a URI that refers to a term from the topology attribute branch of the SO, if the topology is known.
Reference: Section 7.7 on page 22

#### *Rules for the ComponentInstance class*

2.0.1 **sbol-10601** ☑ ~~A ComponentInstance MUST inherit all properties of the Identified class.~~
~~Reference: Section 7.7.1 on page 24~~

**sbol-10602** ☑ The `definition` property of a `ComponentInstance` is REQUIRED and MUST contain a `URI`.
Reference: Section 7.7.1 on page 27

**sbol-10603** ☑ The `definition` property of a `ComponentInstance` MUST NOT contain a `URI` reference to the `ComponentDefinition` that contains the `ComponentInstance`.
Reference: Section 7.7.1 on page 27

2.0.1 **sbol-10604** ● The `URI` contained by the `definition` property MUST refer to a `ComponentDefinition` object.
Reference: Section 7.7.1 on page 27

**sbol-10605** ● `ComponentInstance` objects MUST NOT form circular reference chains via their `definition` properties and parent `ComponentDefinition` objects.
Reference: Section 7.7.1 on page 27

**sbol-10606** ☑ The `mapsTos` property of a `ComponentInstance` is OPTIONAL and MAY contain a set of `MapsTo` objects.
Reference: Section 7.7.1 on page 27

**sbol-10607** ☑ The `access` property of a `ComponentInstance` is REQUIRED and MUST contain a `URI` from Table 5
Reference: Section 7.7.1 on page 27

2.3.0 **sbol-10608** ☑ The `measures` property of a `ComponentInstance` is OPTIONAL and may contain a set of `Measure` objects.
Reference: Section 7.7.1 on page 27

#### *Rules for the Component class*

2.1.0 **sbol-10701** ☑ A `Component` MUST NOT have properties other than the following: `identity`, `persistentIdentity`, `displayId`, `version`, `wasDerivedFroms`, `wasGeneratedBys` ,
2.2.0 `name`, `description`, `annotations`, `access`, `definition`, `mapsTos`, `roles`, `roleIntegration`, and `sourceLocations` .
2.3.0 Reference: Section 7.7.2 on page 28

2.1.0 **sbol-10702** ☑ The `roles` property of a `Component` is OPTIONAL and MAY contain a set of `URI`s.
Reference: Section 7.7.2 on page 28

2.1.0 **sbol-10703** ▲ Each `URI` contained by the `roles` property of a `Component` MUST refer to an ontology term that clarifies the potential function of the `Component` in a biochemical or physical context.
Reference: Section 7.7.2 on page 28

2.1.0 **sbol-10704** ▲ Each `URI` contained by the `roles` property of a `Component` MUST refer to an ontology term that is consistent with the `types` property of the `ComponentDefinition` referred to by its `definition` property.
Reference: Section 7.7.2 on page 28

2.1.0 **sbol-10705** ▲ The `roles` property of a `Component` MUST contain a `URI` from Table 4 if it is well-described by this `URI`.
Reference: Section 7.7.2 on page 28

2.1.0 **sbol-10706** ★ The roles property of a Component SHOULD NOT contain a URI that refers to a term from the sequence feature branch of the SO unless the types property of the ComponentDefinition referred to by its definition property contains the DNA or RNA type URI listed in Table 2.
Reference: Section 7.7.2 on page 28

2.1.0 **sbol-10707** ★ If the types property of the ComponentDefinition referred to by its definition contains the DNA or RNA type URI, then its roles property SHOULD contain no more than one URI that refers to a term from the sequence feature branch of the SO.
Reference: Section 7.7.2 on page 28

2.1.0 **sbol-10708** ☑ The roleIntegration property of a Component, if provided, MUST contain a URI from Table 6.
Reference: Section 7.7.2 on page 28

2.1.0 **sbol-10709** ☑ The roleIntegration property of a Component is REQUIRED, if roles property includes one or more roles.
Reference: Section 7.7.2 on page 28

2.3.0 **sbol-10710** ☑ The sourceLocations property of a Component is OPTIONAL and MAY contain a set of Location objects.
Reference: Section 7.7.2 on page 28

2.3.0 **sbol-10711** ★ The Location objects contained by the sourceLocations property of a single Component SHOULD NOT specify overlapping regions.
Reference: Section 7.7.2 on page 28

2.3.0 **sbol-10712** ★ If the sourceLocations property of a Component refers to a set of Ranges, and that Component is referenced by the component property of a SequenceAnnotation whose locations property also refers to a set of Ranges, then the sum of lengths of the two sets of Ranges MUST be equal.
Reference: Section 7.7.2 on page 28

#### *Rules for the MapsTo class*

2.0.1 **sbol-10801** ☑ A MapsTo MUST NOT have properties other than the following: identity, persistentIdentity, displayId, version, wasDerivedFroms, wasGeneratedBys , name,
2.2.0 description, annotations, refinement, local, and remote
Reference: Section 7.7.3 on page 30

**sbol-10802** ☑ The local property of a MapsTo is REQUIRED and MUST contain a URI.
Reference: Section 7.7.3 on page 30

**sbol-10803** ☑ If a MapsTo is contained by a Component in a ComponentDefinition, then the local property of the MapsTo MUST refer to another Component in the ComponentDefinition.
Reference: Section 7.7.3 on page 30

**sbol-10804** ☑ If a MapsTo is contained by a FunctionalComponent or Module in a ModuleDefinition, then the local property of the MapsTo MUST refer to another FunctionalComponent in the ModuleDefinition.
Reference: Section 7.7.3 on page 30

**sbol-10805** ☑ The remote property of a MapsTo is REQUIRED and MUST contain a URI.
Reference: Section 7.7.3 on page 30

2.0.1 **sbol-10806** ▲ ~~The remote property of a MapsTo MUST refer to a ComponentInstance.~~
~~Reference: Section 7.7.3 on page 26~~

**sbol-10807** ● The `ComponentInstance` referred to by the `remote` property of a `MapsTo` MUST have an `access` property that contains the `URI` `http://sbols.org/v2#public`.
Reference: Section 7.7.3 on page 30

**sbol-10808** ● If a `MapsTo` is contained by a `ComponentInstance`, then the `remote` property of the `MapsTo` MUST refer to a `Component` in the `ComponentDefinition` that is referenced by the `definition` property of the `ComponentInstance`.
Reference: Section 7.7.3 on page 30

**sbol-10809** ● If a `MapsTo` is contained by a `Module`, then the `remote` property of the `MapsTo` MUST refer to a `FunctionalComponent` in the `ModuleDefinition` that is referenced by the `definition` property of the `Module`.
Reference: Section 7.7.3 on page 30

**sbol-10810** ☑ The `refinement` property of a `MapsTo` is REQUIRED and MUST contain a `URI` from Table 7.
Reference: Section 7.7.3 on page 30

2.0.1 **sbol-10811** ● If the `refinement` property of a `MapsTo` contains the `URI` `http://sbols.org/v2#verifyIdentical`, then the `ComponentInstance` objects referred to by `local` and `remote` properties of the `MapsTo` MUST refer to the same `ComponentDefinition` via their `definition` properties.
Reference: Section 7.7.3 on page 30

#### *Rules for the SequenceAnnotation class*

2.1.0 **sbol-10901** ☑ A `SequenceAnnotation` MUST NOT have properties other than the following: `identity`, `persistentIdentity`, `displayId`, `version`, `wasDerivedFroms`, `wasGeneratedBys` , `name`,
2.2.0 `description`, `annotations`, `component`, `locations`, and `roles`.
Reference: Section 7.7.4 on page 32

**sbol-10902** ☑ The `locations` property of a `SequenceAnnotation` is REQUIRED and MUST contain a non-empty set of `Location` objects.
Reference: Section 7.7.4 on page 32

**sbol-10903** ★ The `Location` objects contained by the `locations` property of a single `SequenceAnnotation` SHOULD NOT specify overlapping regions.
Reference: Section 7.7.4 on page 32

**sbol-10904** ☑ The `component` property is OPTIONAL and MAY contain a `URI` reference to a `Component`.
Reference: Section 7.7.4 on page 32

**sbol-10905** ☑ The `Component` referenced by the `component` property of a `SequenceAnnotation` MUST be contained by the `ComponentDefinition` that contains the `SequenceAnnotation`.
Reference: Section 7.7.4 on page 32

2.1.0 **sbol-10906** ☑ The `roles` property of a `SequenceAnnotation` is OPTIONAL and MAY contain a set of `URI`s.
Reference: Section 7.7.4 on page 32

2.1.0

**sbol-10907** ▲ Each URI contained by the roles property of a SequenceAnnotation MUST refer to an ontology term that clarifies the potential function of the SequenceAnnotation in a biochemical or physical context.
Reference: Section 7.7.4 on page 32

2.1.0 **sbol-10908** ▲ The roles property of a SequenceAnnotation MUST contain a URI from Table 4 if it is well-described by this URI.
Reference: Section 7.7.4 on page 32

2.1.0 **sbol-10909** ☑ A SequenceAnnotation MUST NOT include both a component property and a roles property.
Reference: Section 7.7.4 on page 32

#### Rules for the Location class

2.0.1 **sbol-11001** ☑ ~~A Location MUST inherit all properties of the Identified class.~~
~~Reference: Section 7.7.5 on page 30~~

**sbol-11002** ☑ The orientation property of a Location is OPTIONAL and MAY contain a URI from Table 8.
Reference: Section 7.7.5 on page 36

2.3.0 **sbol-11003** ● The sequence property of a Location is OPTIONAL. If present, it MUST contain the URI of a Sequence referenced by the ComponentDefinition containing the Location.
Reference: Section 7.7.5 on page 34

#### Rules for the Range class

2.0.1 **sbol-11101** ☑ A Range MUST NOT have properties other than the following: identity, persistentIdentity, displayId, version, wasDerivedFroms, wasGeneratedBys , name,
2.2.0 description, annotations, sequence, orientation, start, and end.
2.3.0 Reference: Section 7.7.5 on page 35

**sbol-11102** ☑ The start property of a Range is REQUIRED and MUST contain an Integer greater than zero.
Reference: Section 7.7.5 on page 35

**sbol-11103** ☑ The end property of a Range is REQUIRED and MUST contain an Integer greater than zero.
Reference: Section 7.7.5 on page 35

**sbol-11104** ☑ The value of the end property of a Range MUST be greater than or equal to the value of its start property.
Reference: Section 7.7.5 on page 35

2.3.0 **sbol-11105** ● The sequence property of a Range is OPTIONAL. If present, it MUST contain the URI of a Sequence referenced by the ComponentDefinition containing the Range.
Reference: Section 7.7.5 on page 34

#### Rules for the Cut class

2.0.1 **sbol-11201** ☑ A Cut MUST NOT have properties other than the following: identity, persistentIdentity, displayId, version, wasDerivedFroms, wasGeneratedBys , name, description,
2.2.0 annotations, orientation, sequence, and at.
2.3.0 Reference: Section 7.7.5 on page 35

**sbol-11202** ☑ The at property of a Cut is REQUIRED and MUST contain an Integer greater than or equal to zero.
Reference: Section 7.7.5 on page 35

2.3.0 **sbol-11203** ● The `sequence` property of a `Cut` is OPTIONAL. If present, it MUST contain the URI of a `Sequence` referenced by the `ComponentDefinition` containing the `Cut`.
Reference: Section 7.7.5 on page 34

#### *Rules for the GenericLocation class*

2.0.1 **sbol-11301** ☑ A `GenericLocation` MUST NOT have properties other than the following: `identity`, `persistentIdentity`, `displayId`, `version`, `wasDerivedFroms`, `wasGeneratedBys` , `name`,
2.2.0 `description`, `annotations`, `sequence`, and `orientation`.
2.3.0 Reference: Section 7.7.5 on page 36

2.3.0 **sbol-11302** ● The `sequence` property of a `GenericLocation` is OPTIONAL. If present, it MUST contain the URI of a `Sequence` referenced by the `ComponentDefinition` containing the `GenericLocation`.
Reference: Section 7.7.5 on page 34

#### *Rules for the SequenceConstraint class*

2.0.1 **sbol-11401** ☑ A `SequenceConstraint` MUST NOT have properties other than the following: `identity`, `persistentIdentity`, `displayId`, `version`, `wasDerivedFroms`, `wasGeneratedBys` , `name`,
2.2.0 `description`, `annotations`, `restriction`, `subject`, and `object`.
Reference: Section 7.7.6 on page 36

**sbol-11402** ☑ The `subject` property of a `SequenceConstraint` is REQUIRED and MUST contain a `URI` reference to a `Component`.
Reference: Section 7.7.6 on page 36

**sbol-11403** ☑ The `Component` referenced by the `subject` property of a `SequenceConstraint` MUST be contained by the `ComponentDefinition` that contains the `SequenceConstraint`.
Reference: Section 7.7.6 on page 36

**sbol-11404** ☑ The `object` property of a `SequenceConstraint` is REQUIRED and MUST contain a `URI` reference to a `Component`.
Reference: Section 7.7.6 on page 36

**sbol-11405** ☑ The `Component` referenced by the `object` property of a `SequenceConstraint` MUST be contained by the `ComponentDefinition` that contains the `SequenceConstraint`.
Reference: Section 7.7.6 on page 36

**sbol-11406** ☑ The `object` property of a `SequenceConstraint` MUST NOT refer to the same `Component` as the `subject` property of the `SequenceConstraint`.
Reference: Section 7.7.6 on page 36

**sbol-11407** ☑ The `restriction` property of a `SequenceConstraint` is REQUIRED and MUST contain a `URI`.
Reference: Section 7.7.6 on page 36

**sbol-11408** ▲ The `URI` contained by the `restriction` property of a `SequenceConstraint` MUST indicate the type of structural restriction on the relative, sequence-based positions or orientations of the `Component` objects referred to by the `subject` and `object` properties of the `SequenceConstraint`.
Reference: Section 7.7.6 on page 36

2.0.1 **sbol-11409** ● If the `restriction` property of a `SequenceConstraint` contains the `URI` `http://sbols.org/v2#precedes`, then the position of the `Component` referred to by the `subject` property of the `SequenceConstraint` MUST precede that of the `Component` referred to by its `object` property.
Reference: Section 7.7.6 on page 36

**sbol-11410** ● If the restriction property of a SequenceConstraint contains the URI http://sbols.org/v2#sameOrientationAs, then the orientation of the Component referred to by the subject property of the SequenceConstraint MUST be the same as that of the Component referred to by its object property.
Reference: Section 7.7.6 on page 36

**sbol-11411** ● If the restriction property of a SequenceConstraint contains the URI http://sbols.org/v2#oppositeOrientationAs, then the orientation of the Component referred to by the subject property of the SequenceConstraint MUST be opposite that of the Component referred to by its object property.
Reference: Section 7.7.6 on page 36

**sbol-11412** ★ The URI contained by the restriction property SHOULD come from Table 9.
Reference: Section 7.7.6 on page 36

2.2.0 **sbol-11413** ☑ If the restriction property of a SequenceConstraint contains the URI http://sbols.org/v2#differentFrom, then the definition property of the subject Component of the SequenceConstraint MUST NOT refer to the the same URI as the definition property of the object Component of the SequenceConstraint.
Reference: Section 7.7.6 on page 36

2.2.1 **sbol-11414** ● If the restriction property of a SequenceConstraint contains the URI http://sbols.org/v2#differentFrom, then the definition property of the subject Component of the SequenceConstraint MUST NOT refer to the same object as the definition property of the object Component of the SequenceConstraint.
Reference: Section 7.7.6 on page 36

2.2.1 **sbol-11415** ● If the restriction property of a SequenceConstraint contains the URI http://sbols.org/v2#differentFrom, then the definition property of the subject Component of the SequenceConstraint MUST NOT refer to the same object as the definition property of the object Component of the SequenceConstraint.
Reference: Section 7.7.6 on page 36

#### *Rules for the Model class*

2.0.1 **sbol-11501** ☑ A Model MUST NOT have properties other than the following: identity, persistentIdentity, displayId, version, wasDerivedFroms, wasGeneratedBys , name,
2.2.0 description, annotations, source, language, and framework.
Reference: Section 7.8 on page 38

**sbol-11502** ☑ The source property of a Model is REQUIRED and MUST contain a URI.
Reference: Section 7.8 on page 38

**sbol-11503** ▲ The URI contained by the source property of a Model MUST specify the location of the model's source file.
Reference: Section 7.8 on page 38

**sbol-11504** ☑ The language property of a Model is REQUIRED and MUST contain a URI.
Reference: Section 7.8 on page 38

**sbol-11505** ▲ The URI contained by the language property of a Model MUST specify the language in which the model is encoded.
Reference: Section 7.8 on page 38

**sbol-11506** ▲ The language property of a Model MUST contain a URI from Table 10 if it is well-described by this URI.
Reference: Section 7.8 on page 38

**sbol-11507** ★ The language property of a Model SHOULD contain a URI that refers to a term from the EDAM ontology.
Reference: Section 7.8 on page 38

**sbol-11508** ☑ The framework property of a Model is REQUIRED and MUST contain a URI.
Reference: Section 7.8 on page 38

**sbol-11509** ▲ The URI contained by the framework property of a Model MUST specify the modeling framework of the model.
Reference: Section 7.8 on page 38

**sbol-11510** ▲ The framework property of a Model MUST contain a URI from Table 11 if it is well-described by this URI.
Reference: Section 7.8 on page 38

**sbol-11511** ★ The framework property SHOULD contain a URI that refers to a term from the modeling framework branch of the SBO.
Reference: Section 7.8 on page 38

#### *Rules for the ModuleDefinition class*

2.0.1 **sbol-11601** ☑ A ModuleDefinition MUST NOT have properties other than the following: identity, persistentIdentity, displayId, version, wasDerivedFroms, wasGeneratedBys , name,
2.2.0 description, annotations, roles, modules, interactions, functionalComponents, and models.
Reference: Section 7.9 on page 39

**sbol-11602** ☑ The roles property is OPTIONAL and MAY contain a set of URIs.
Reference: Section 7.9 on page 39

**sbol-11603** ▲ Each URI contained by roles property of a ModuleDefinition MUST refer to a resource that clarifies the intended function of the ModuleDefinition.
Reference: Section 7.9 on page 39

**sbol-11604** ☑ The modules property OPTIONAL and MAY contain a set of Module objects.
Reference: Section 7.9 on page 39

**sbol-11605** ☑ The interactions property is OPTIONAL and MAY contain a set of Interaction objects.
Reference: Section 7.9 on page 39

**sbol-11606** ☑ The functionalComponents property is OPTIONAL and MAY contain a set of FunctionalComponent objects.
Reference: Section 7.9 on page 39

**sbol-11607** ☑ The models property is OPTIONAL and MAY contain a set of URIs.
Reference: Section 7.9 on page 39

2.0.1 **sbol-11608** ● Each URI contained by the models property of a ModuleDefinition MUST refer to a Model.
Reference: Section 7.9 on page 39

**sbol-11609** ☑ If multiple MapsTos belonging to the Modules and FunctionalComponents of a ModuleDefinition have local properties that refer to the same FunctionalComponent, then there MUST NOT be more than one such MapsTo that has a refinement property that contains the URI http://sbols.org/v2#useRemote.
Reference: Section 7.7.3 on page 30

#### *Rules for the Module class*

2.0.1 **sbol-11701** ☑ A Module MUST NOT have properties other than the following: identity, persistentIdentity, displayId, version, wasDerivedFroms, wasGeneratedBys , name,
2.2.0 description, annotations, definition, and mapsTos.
Reference: Section 7.9.1 on page 42

**sbol-11702** ☑ The definition property of a Module is REQUIRED and MUST contain a URI.
Reference: Section 7.9.1 on page 42

2.0.1 **sbol-11703** ● The URI contained by the definition property of Module MUST refer to a ModuleDefinition.
Reference: Section 7.9.1 on page 42

**sbol-11704** ☑ The definition property of a Module MUST NOT contain a URI reference to the ModuleDefinition that contains the Module.
Reference: Section 7.9.1 on page 42

2.0.1 **sbol-11705** ● Module objects MUST NOT form circular reference chains via their definition properties and parent ModuleDefinition objects.
Reference: Section 7.9.1 on page 42

**sbol-11706** ☑ The mapsTos property is OPTIONAL and MAY contain a set of MapsTo objects.
Reference: Section 7.9.1 on page 42

2.3.0 **sbol-11707** ☑ The measures property of a Module is OPTIONAL and may contain a set of Measure objects.
Reference: Section 7.9.1 on page 42

#### *Rules for the FunctionalComponent class*

2.0.1 **sbol-11801** ☑ A FunctionalComponent MUST NOT have properties other than the following: identity, persistentIdentity, displayId, version, wasDerivedFroms, wasGeneratedBys , name,
2.2.0 description, annotations, access, definition, and mapsTos, and direction.
Reference: Section 7.7.1 on page 27

**sbol-11802** ☑ The direction property of a FunctionalComponent is REQUIRED and MUST contain a URI from Table 12.
Reference: Section 7.9.2 on page 43

#### *Rules for the Interaction class*

2.0.1 **sbol-11901** ☑ An Interaction MUST NOT have properties other than the following: identity, persistentIdentity, displayId, version, wasDerivedFroms, wasGeneratedBys , name,
2.2.0 description, annotations, types, and participations.
Reference: Section 7.9.3 on page 44

**sbol-11902** ☑ The types property of an Interaction is REQUIRED and MUST contain a non-empty set of URIs.
Reference: Section 7.9.3 on page 44

**sbol-11903** ▲ Each URI contained by the types property of an Interaction MUST refer to an ontology term that describes the behavior represented by the Interaction.
Reference: Section 7.9.3 on page 44

**sbol-11904** ▲ All URIs contained by the types property of an Interaction MUST refer to non-conflicting ontology terms.
Reference: Section 7.9.3 on page 44

2.0.1 **sbol-11905** ★ Exactly one URI contained by the types property of an Interaction SHOULD refer to a term from the occurring entity relationship branch of the SBO.
Reference: Section 7.9.3 on page 44

**sbol-11906** ☑ The participations property of an Interaction is OPTIONAL and MAY contain a set of Participation objects.
Reference: Section 7.9.3 on page 44

2.0.1 **sbol-11907** ★ If the participations property of an Interaction refers to one or more Participation objects, and one of the types of this Interaction comes from Table 13, then the Participation objects SHOULD have a role from the set of roles that is cross-listed with this type in Table 14.
Reference: Section 7.9.4 on page 46

2.3.0 **sbol-11908** ☑ The measures property of an Interaction is OPTIONAL and may contain a set of Measure objects.
Reference: Section 7.9.3 on page 44

#### *Rules for the Participation class*

2.0.1 **sbol-12001** ☑ A Participation MUST NOT have properties other than the following: identity, persistentIdentity, displayId, version, wasDerivedFroms, wasGeneratedBys , name,
2.2.0 description, annotations, roles, and participant.
Reference: Section 7.9.4 on page 46

**sbol-12002** ☑ The participant property of a Participation is REQUIRED and MUST contain a URI reference to a FunctionalComponent.
Reference: Section 7.9.4 on page 46

**sbol-12003** ☑ The FunctionalComponent referenced by the participant property of a Participation MUST be contained by the ModuleDefinition that contains the Interaction which contains the Participation.
Reference: Section 7.9.4 on page 46

2.0.1 **sbol-12004** ☑ The roles property of a Participation is REQUIRED and MUST contain a non-empty set of URIs.
Reference: Section 7.9.4 on page 46

**sbol-12005** ▲ Each URI contained by the roles property of an Participation MUST refer to an ontology term that describes the behavior represented by the Participation.
Reference: Section 7.9.4 on page 46

**sbol-12006** ▲ All URIs contained by the roles property of an Participation MUST refer to non-conflicting ontology terms.
Reference: Section 7.9.4 on page 46

2.0.1

**sbol-12007** ★ Exactly one role in the set of `roles` SHOULD be a `URI` from the participant role branch of the SBO (see Table 14).
Reference: Section 7.9.4 on page 46

2.3.0 **sbol-12008** ☑ The `measures` property of a `Participation` is OPTIONAL and may contain a set of `Measure` objects.
Reference: Section 7.9.4 on page 46

#### *Rules for the Collection class*

2.0.1 **sbol-12101** ☑ A `Collection` MUST NOT have properties other than the following: `identity`, `persistentIdentity`, `displayId`, `version`, `wasDerivedFroms`, `wasGeneratedBys`, `name`,
2.2.0 `description`, `annotations`, and `members`.
Reference: Section 7.10 on page 47

**sbol-12102** ☑ The `members` property of a `Collection` is OPTIONAL and MAY contain a set of `URI`s.
Reference: Section 7.10 on page 47

2.0.1 **sbol-12103** ● Each `URI` contained by the `members` property of a `Collection` MUST reference a `TopLevel` object.
Reference: Section 7.10 on page 47

#### *Rules for the Annotation class*

2.2.0 **sbol-12201** ☑ The name property of an Annotation is REQUIRED and MUST contain a QName.
Reference: Section 7.16 on page 56

2.0.1 **sbol-12202** ☑ ~~The value property of an Annotation is REQUIRED and MUST contain an AnnotationValue.~~
~~Reference: Section 7.11 on page 46~~

2.2.0 **sbol-12203** ☑ An `AnnotationValue` MUST be a `literal` (a `String`, `Integer`, `Double`, or `Boolean`), `URI`, or a `NestedAnnotations` object.
Reference: Section 7.16 on page 56

**sbol-12204** ☑ The `nestedQName` property of a `NestedAnnotations` object is REQUIRED and MUST contain a `QName`.
Reference: Section 7.16 on page 56

**sbol-12205** ☑ The `nestedURI` property of a `NestedAnnotations` object is REQUIRED and MUST contain a `URI`.
Reference: Section 7.16 on page 56

**sbol-12206** ☑ The `annotations` property of a `NestedAnnotations` object is OPTIONAL and MAY contain a set of `Annotation` objects.
Reference: Section 7.16 on page 56

#### *Rules for the GenericTopLevel class*

2.0.1 **sbol-12301** ☑ A `GenericTopLevel` MUST NOT have properties other than the following: `identity`, `persistentIdentity`, `displayId`, `version`, `wasDerivedFroms`, `wasGeneratedBys` , `name`,
2.2.0 `description`, and `annotations`.
Reference: Section 7.16.2 on page 58

**sbol-12302** ☑ The `rdfType` property of a `GenericTopLevel` object is REQUIRED and MUST contain a `QName`.
Reference: Section 7.16.2 on page 58

2.2.1 **sbol-12303** ☑ The rdfType property of a GenericTopLevel object MUST NOT contain a QName in the "http://sbols.org/v2#" namespace.
Reference: Section 7.16.2 on page 58

2.2.0

### *Rules for the Activity class*

2.3.0 **sbol-12401** ☑ An Activity MUST NOT have properties other than the following: identity, persistentIdentity, displayId, version, wasDerivedFroms, wasGeneratedBys, name, description, annotations, types, startedAtTime, endedAtTime, wasInformedBys, usages, and associations.
Reference: Section 13.1.1 on page 75

**sbol-12402** ☑ The startedAtTime property of an Activity object is OPTIONAL and MAY contain a DateTime.
Reference: Section 13.1.1 on page 75

**sbol-12403** ☑ The endedAtTime property of an Activity object is OPTIONAL and MAY contain a DateTime.
Reference: Section 13.1.1 on page 75

**sbol-12404** ☑ The associations property of an Activity is OPTIONAL and MAY contain a set of Association objects.
Reference: Section 13.1.1 on page 75

**sbol-12405** ☑ The usages property of an Activity is OPTIONAL and MAY contain a set of Usage objects.
Reference: Section 13.1.1 on page 75

**sbol-12406** ☑ The wasInformedBys property of an Activity is OPTIONAL and MAY contain a set of URIs.
Reference: Section 13.1.1 on page 75

**sbol-12407** ● Each URI contained by the wasInformedBys property of an Activity MUST refer to a Activity object.
Reference: Section 13.1.1 on page 75

**sbol-12408** ★ An Activity with a child Association that has a roles property that contains the URI http://sbols.org/v2#design SHOULD NOT have child Usage objects that have roles properties that contain the URIs http://sbols.org/v2#build or http://sbols.org/v2#test.
Reference: Section 13.1.1 on page 75

**sbol-12409** ★ An Activity with a child Association that has a roles property that contains the URI http://sbols.org/v2#build SHOULD NOT have child Usage objects that have roles properties that contain the URIs http://sbols.org/v2#test or http://sbols.org/v2#learn.
Reference: Section 13.1.1 on page 75

**sbol-12410** ★ An Activity with a child Association that has a roles property that contains the URI http://sbols.org/v2#test SHOULD NOT have child Usage objects that have roles properties that contain the URIs http://sbols.org/v2#design or http://sbols.org/v2#learn.
Reference: Section 13.1.1 on page 75

**sbol-12411** ★ An Activity with a child Association that has a roles property that contains the URI http://sbols.org/v2#learn SHOULD NOT have child Usage objects that have roles properties that contain the URIs http://sbols.org/v2#design or http://sbols.org/v2#build.
Reference: Section 13.1.1 on page 75

2.3.0

**sbol-12412** ☑ The `types` property of an `Activity` object is OPTIONAL and MAY contain a set of `URI`s.
Reference: Section 13.1.1 on page 75

**sbol-12413** ★ If an `Activity` has a types value of `http://sbols.org/v2#design` and also contains an `Association`, then that `Association` MUST have a roles value of `http://sbols.org/v2#design`.
Reference: Section 13.1.1 on page 75

**sbol-12414** ★ If an `Activity` has a types value of `http://sbols.org/v2#build` and also contains an `Association`, then that `Association` MUST have a roles value of `http://sbols.org/v2#build`.
Reference: Section 13.1.1 on page 75

**sbol-12415** ★ If an `Activity` has a types value of `http://sbols.org/v2#test` and also contains an `Association`, then that `Association` MUST have a roles value of `http://sbols.org/v2#test`.
Reference: Section 13.1.1 on page 75

**sbol-12416** ★ If an `Activity` has a types value of `http://sbols.org/v2#learn` and also contains an `Association`, then that `Association` MUST have a roles value of `http://sbols.org/v2#learn`.
Reference: Section 13.1.1 on page 75

2.2.0

#### *Rules for the Usage class*

**sbol-12501** ☑ An `Usage` MUST NOT have properties other than the following: `identity`, `persistentIdentity`, `displayId`, `version`, `wasDerivedFroms`, `wasGeneratedBys`, `name`, `description`, `annotations`, `entity`, and `roles`.
Reference: Section 13.1.2 on page 76

**sbol-12502** ☑ The `entity` property of an `Usage` is REQUIRED and MUST contain a `URI`.
Reference: Section 13.1.2 on page 76

**sbol-12503** ☑ The `roles` property of an `Usage` is OPTIONAL and MAY contain a set of `URI`s.
Reference: Section 13.1.2 on page 76

**sbol-12504** ★ A `Usage` that has a `roles` property that contains the `URI` `http://sbols.org/v2#design` SHOULD refer to a `TopLevel` other than `Implementation`.
Reference: Section 13.1.2 on page 76

**sbol-12505** ★ A `Usage` that has a `roles` property that contains the `URI` `http://sbols.org/v2#build` SHOULD refer to an `Implementation`.
Reference: Section 13.1.2 on page 76

**sbol-12506** ★ A `Usage` that has a `roles` property that contains the `URI` `http://sbols.org/v2#test` SHOULD refer to an `ExperimentalData`.
Reference: Section 13.1.2 on page 76

**sbol-12507** ★ A `Usage` that has a `roles` property that contains the `URI` `http://sbols.org/v2#learn` SHOULD NOT refer to an `Implementation`.
Reference: Section 13.1.2 on page 76

#### *Rules for the Association class*

**sbol-12601** ☑ An Association MUST NOT have properties other than the following: identity, persistentIdentity, displayId, version, wasDerivedFroms, wasGeneratedBys, name, description, annotations, roles, plan, and agent.
Reference: Section 13.1.3 on page 76

**sbol-12602** ☑ The roles property of an Association is OPTIONAL and MAY contain a set of URIs.
Reference: Section 13.1.3 on page 76

**sbol-12603** ☑ The plan property of an Association is OPTIONAL and MAY contain a URI.
Reference: Section 13.1.3 on page 76

**sbol-12604** ● The URI contained by the plan property MUST refer to a Plan object.
Reference: Section 13.1.3 on page 76

**sbol-12605** ☑ The agent property of an Association is REQUIRED and MUST contain a URI.
Reference: Section 13.1.3 on page 76

**sbol-12606** ● The URI contained by the agent property MUST refer to an Agent object.
Reference: Section 13.1.3 on page 76

2.2.0

#### *Rules for the Plan class*

**sbol-12701** ☑ A Plan MUST NOT have properties other than the following: identity, persistentIdentity, displayId, version, wasDerivedFroms, wasGeneratedBys, name, description, and annotations.
Reference: Section 13.1.4 on page 78

2.2.0

#### *Rules for the Agent class*

**sbol-12801** ☑ An Agent MUST NOT have properties other than the following: identity, persistentIdentity, displayId, version, wasDerivedFroms, wasGeneratedBys, name, description, and annotations.
Reference: Section 13.1.5 on page 78

2.2.0

#### *Rules for the CombinatorialDerivation class*

**sbol-12901** ☑ A CombinatorialDerivation MUST NOT have properties other than the following: identity, persistentIdentity, displayId, version, wasDerivedFroms, wasGeneratedBys, name, description, annotations, strategy, template, and variableComponents.
Reference: Section 7.11 on page 48

**sbol-12902** ☑ The strategy property of a CombinatorialDerivation MUST contain a URI from Table 15 if the property is not empty.
Reference: Section 7.11 on page 48

**sbol-12903** ☑ If the strategy property of a CombinatorialDerivation contains the URI http://sbols.org/v2#enumerate, then its variableComponents property MUST NOT contain a VariableComponent with an operator property that contains the URI http://sbols.org/v2#zeroOrMore or the URI http://sbols.org/v2#oneOrMore.
Reference: Section 7.11 on page 48

**sbol-12904** ☑ The template property of a CombinatorialDerivation is REQUIRED and MUST contain a URI.
Reference: Section 7.11 on page 48

**sbol-12905** ● The URI contained by the template property of a CombinatorialDerivation MUST refer to a ComponentDefinition.
Reference: Section 7.11 on page 48

**sbol-12906** ☑ The variableComponents property of a CombinatorialDerivation is OPTIONAL and MAY contain a set of VariableComponent objects.
Reference: Section 7.11 on page 48

**sbol-12907** ☑ The variableComponents property of a CombinatorialDerivation MUST NOT contain two or more VariableComponent objects with variable properties that contain the same URI.
Reference: Section 7.11 on page 48

**sbol-12908** ● If the wasDerivedFroms property of a ComponentDefinition refers to a CombinatorialDerivation and the template ComponentDefinition of the CombinatorialDerivation contains SequenceConstraint objects, then any Component contained by the first ComponentDefinition that has a wasDerivedFroms property that refers to the subject or object Component of any of the SequenceConstraint objects MUST adhere to the restriction properties of these SequenceConstraint objects.
Reference: Section 7.11 on page 48

**sbol-12909** ★ The components property of the template ComponentDefinition of a CombinatorialDerivation SHOULD contain one or more Component objects.
Reference: Section 7.11 on page 48

**sbol-12910** ★ If the wasDerivedFroms property of a ComponentDefinition refers to a CombinatorialDerivation, then the types property of this ComponentDefinition SHOULD contain all URIs contained by the types property of the template ComponentDefinition of the CombinatorialDerivation.
Reference: Section 7.11 on page 48

**sbol-12911** ★ If the wasDerivedFroms property of a ComponentDefinition refers to a CombinatorialDerivation, then the roles property of this ComponentDefinition SHOULD contain all URIs contained by the roles property of the template ComponentDefinition of the CombinatorialDerivation.
Reference: Section 7.11 on page 48

**sbol-12912** ★ If the members property of a Collection refers only to objects with wasDerivedFroms properties that refer to a CombinatorialDerivation, then the wasDerivedFroms property of the Collection SHOULD also refer to the CombinatorialDerivation.
Reference: Section 7.11 on page 48

**sbol-12913** ★ If the wasDerivedFroms property of a Collection refers to a CombinatorialDerivation, then the wasDerivedFroms properties of the objects that are referred to by its members property SHOULD also refer to the CombinatorialDerivation.
Reference: Section 7.11 on page 48

2.2.1

**sbol-12914** ☑ The strategy property of an CombinatorialDerivation is OPTIONAL and MAY contain a URI.
Reference: Section 7.11 on page 48

2.2.0

#### *Rules for the VariableComponent class*

**sbol-13001** ☑ A VariableComponent MUST NOT have properties other than the following: identity, persistentIdentity, displayId, version, wasDerivedFroms, wasGeneratedBys, name, description, annotations, operator, variable, variants, variantCollections, and variantDerivations.
Reference: Section 7.11.1 on page 50

**sbol-13002** ☑ The operator property of a VariableComponent is REQUIRED and MUST contain a URI.
Reference: Section 7.11.1 on page 50

**sbol-13003** ☑ The URI contained by the operator property of a VariableComponent MUST come from Table 16.
Reference: Section 7.11.1 on page 50

**sbol-13004** ☑ The variable property of a VariableComponent is REQUIRED and MUST contain a URI.
Reference: Section 7.11.1 on page 50

**sbol-13005** ● The URI contained by the variable property of a VariableComponent MUST refer to a Component contained by the components property of the template ComponentDefinition of the CombinatorialDerivation that contains the VariableComponent.
Reference: Section 7.11.1 on page 50

2.2.1 **sbol-13006** ★ At least one of the variants property, variantCollections property, or variantDerivations property of a VariableComponent MUST NOT be empty.
Reference: Section 7.11.1 on page 50

2.2.0 **sbol-13007** ☑ The variants property of a VariableComponent is OPTIONAL and MAY contain a set of URIs.
Reference: Section 7.11.1 on page 50

**sbol-13008** ● The URIs contained by the variants property of a VariableComponent MUST refer to ComponentDefinition objects.
Reference: Section 7.11.1 on page 50

**sbol-13009** ☑ The variantCollections property of a VariableComponent is OPTIONAL and MAY contain a set of URIs.
Reference: Section 7.11.1 on page 50

**sbol-13010** ● The URIs contained by the variantCollections property of a VariableComponent MUST refer to Collection objects.
Reference: Section 7.11.1 on page 50

**sbol-13011** ● The members property of a Collection that is referred to by the variantCollections property of a VariableComponent MUST NOT be empty.
Reference: Section 7.11.1 on page 50

**sbol-13012** ● The members property of a Collection that is referred to by the variantCollections property of a VariableComponent MUST refer only to ComponentDefinition objects.
Reference: Section 7.11.1 on page 50

**sbol-13013** ☑ The variantDerivations property of a VariableComponent is OPTIONAL and MAY contain a set of URIs.
Reference: Section 7.11.1 on page 50

**sbol-13014** ● The URIs contained by the variantDerivations property of a VariableComponent MUST refer to CombinatorialDerivation objects.
Reference: Section 7.11.1 on page 50

**sbol-13015** ● VariableComponent objects MUST NOT form circular reference chains via their variantDerivations properties and parent CombinatorialDerivation objects.
Reference: Section 7.11.1 on page 50

2.2.1

**sbol-13016** ●
If the wasDerivedFroms property of a Component refers to a Component in the template ComponentDefinition of a CombinatorialDerivation, and the second Component is referred to by the variable property of any VariableComponent in the CombinatorialDerivation, then the definition property of the first Component MUST refer to a ComponentDefinition specified by the variants property, a ComponentDefinition that is one of the members of a Collection specified by the variantCollections property, or a ComponentDefinition with a wasDerivedFroms property that refers to a CombinatorialDerivation specified by the variantDerivations property of the VariableComponent.
Reference: Section 7.11.1 on page 50

2.2.0

**sbol-13017** ●
If the wasDerivedFroms property of a Component refers to a Component in the template ComponentDefinition of a CombinatorialDerivation, and the second Component is not referred to by the variable property of any VariableComponent in the CombinatorialDerivation, then the definition property of the first Component MUST refer to the same ComponentDefinition as the definition property of the second Component.
Reference: Section 7.11.1 on page 50

**sbol-13018** ★
If the wasDerivedFroms property of a ComponentDefinition refers to a CombinatorialDerivation and the wasDerivedFroms property of a Component contained by the ComponentDefinition refers to a Component contained by the template ComponentDefinition of the CombinatorialDerivation, then the roles property of the first Component SHOULD contain all URIs contained by the roles property of the second Component.
Reference: Section 7.11.1 on page 50

**sbol-13019** ★
If the wasDerivedFroms property of a ComponentDefinition refers to a CombinatorialDerivation and the CombinatorialDerivation contains a VariableComponent with an operator property that contains the URI http://sbols.org/v2#zeroOrOne, then the components property of the ComponentDefinition SHOULD NOT contain more than one Component with a wasDerivedFroms property that refers to the same Component as the variable property of the VariableComponent.
Reference: Section 7.11.1 on page 50

**sbol-13020** ★
If the wasDerivedFroms property of a ComponentDefinition refers to a CombinatorialDerivation and the CombinatorialDerivation contains a VariableComponent with an operator property that contains the URI http://sbols.org/v2#one, then the components property of the ComponentDefinition SHOULD contain exactly one Component with a wasDerivedFroms property that refers to the same Component as the variable property of the VariableComponent.
Reference: Section 7.11.1 on page 50

**sbol-13021** ★
If the wasDerivedFroms property of a ComponentDefinition refers to a CombinatorialDerivation and the CombinatorialDerivation contains a VariableComponent with an operator property that contains the URI http://sbols.org/v2#oneOrMore, then the components property of the ComponentDefinition SHOULD contain at least one Component with a wasDerivedFroms property that refers to the same Component as the variable property of the VariableComponent.
Reference: Section 7.11.1 on page 50

**sbol-13022** ★ If the wasDerivedFroms property of a ComponentDefinition refers to a CombinatorialDerivation and the template ComponentDefinition of the CombinatorialDerivation contains a Component that is not referred to by the variable property of any VariableComponent contained by the CombinatorialDerivation, then the first ComponentDefinition SHOULD contain exactly one Component with a wasDerivedFroms property that refers to the Component in the template ComponentDefinition.
Reference: Section 7.11.1 on page 50

2.2.0

#### Rules for the Implementation class

**sbol-13101** ☑ An Implementation MUST NOT have properties other than the following: identity, persistentIdentity, displayId, version, wasDerivedFroms, wasGeneratedBys, name, description, annotations, and built.
Reference: Section 7.12 on page 52

**sbol-13102** ☑ The built property of an Implementation is OPTIONAL and MAY contain a URI.
Reference: Section 7.12 on page 52

**sbol-13103** ● The built property URI, if specified, MUST refer to either a ComponentDefinition or a ModuleDefinition.
Reference: Section 7.12 on page 52

2.2.0

#### Rules for the Attachment class

**sbol-13201** ☑ An Attachment MUST NOT have properties other than the following: identity, persistentIdentity, displayId, version, wasDerivedFroms, wasGeneratedBys, name, description, annotations, source, format, size, and hash.
Reference: Section 7.13 on page 53

**sbol-13202** ☑ The source property of an Attachment is REQUIRED and MUST contain a URI.
Reference: Section 7.13 on page 53

**sbol-13203** ▲ The URI contained by the source property of an Attachment MUST specify the location of the model's source file.
Reference: Section 7.13 on page 53

**sbol-13204** ☑ The format property of an Attachment is OPTIONAL and MAY contain a URI.
Reference: Section 7.13 on page 53

**sbol-13205** ▲ The URI contained by the format property of an Attachment MUST specify the file type of the attachment.
Reference: Section 7.13 on page 53

**sbol-13206** ★ The format property of an Attachment SHOULD contain a URI that refers to a term from the EDAM ontology.
Reference: Section 7.13 on page 53

**sbol-13207** ☑ The size property of an Attachment is OPTIONAL and MAY contain a Long.
Reference: Section 7.13 on page 53

**sbol-13208** ☑ The hash property of an Attachment is OPTIONAL and MAY contain a String.
Reference: Section 7.13 on page 53

2.3.0

#### Rules for the ExperimentalData class

**sbol-13301** ☑ An ExperimentalData MUST NOT have properties other than the following: identity, persistentIdentity, displayId, version, wasDerivedFroms, wasGeneratedBys, name, description, annotations, and attachments.
Reference: Section 7.14 on page 54

2.3.0

#### Rules for the Experiment class

**sbol-13401** ☑ An Experiment MUST NOT have properties other than the following: identity, persistentIdentity, displayId, version, wasDerivedFroms, wasGeneratedBys, name, description, annotations, attachments, and experimentalData.
Reference: Section 7.15 on page 55

**sbol-13402** ☑ The experimentalData property of an Experiment is OPTIONAL and MAY contain a set of URIs.
Reference: Section 7.15 on page 55

**sbol-13403** ● The URIs contained by the experimentalData property of a Experiment MUST each refer to an ExperimentalData.
Reference: Section 7.15 on page 55

2.3.0

#### Rules for the Measure class

**sbol-13501** ☑ A Measure MUST NOT have properties other than the following: identity, persistentIdentity, displayId, version, wasDerivedFroms, wasGeneratedBys, name, description, annotations, hasNumericalValue, hasUnit, and types.
Reference: Section 13.2.1 on page 93

**sbol-13502** ☑ The hasNumericalValue property of a Measure is REQUIRED and MUST contain an xsd:float.
Reference: Section 13.2.1 on page 93

**sbol-13503** ☑ The hasUnit property of a Measure is REQUIRED and MUST contain a URI.
Reference: Section 13.2.1 on page 93

**sbol-13504** ☑ The types property of a Measure is OPTIONAL and MAY contain a set of URIs.
Reference: Section 13.2.1 on page 93

**sbol-13505** ★ If the types property of a Measure is non-empty, then exactly one of the URIs that this property contains SHOULD refer to a term from the systems description parameter branch of the SBO.
Reference: Section 13.2.1 on page 93

**sbol-13506** ● The URI contained by the hasUnit property of a Measure MUST refer to a Unit.
Reference: Section 13.2.1 on page 93

2.3.0

#### Rules for the Unit class

**sbol-13601** ☑ The symbol property of a Unit is REQUIRED and MUST contain a String.
Reference: Section 13.2.2 on page 95

**sbol-13602** ☑ The alternativeSymbols property of a Unit is OPTIONAL and MAY contain a set of Strings.
Reference: Section 13.2.2 on page 95

**sbol-13603** ☑ The label property of a Unit is REQUIRED and MUST contain a String.
Reference: Section 13.2.2 on page 95

**sbol-13604** ☑ The alternativeLabels property of a Unit is OPTIONAL and MAY contain a set of Strings.
Reference: Section 13.2.2 on page 95

**sbol-13605** ☑ The comment property of a Unit is OPTIONAL and MAY contain a String.
Reference: Section 13.2.2 on page 95

**sbol-13606** ☑ The longcomment property of a Unit is OPTIONAL and MAY contain a String.
Reference: Section 13.2.2 on page 95

**sbol-13607** ★ If both of the name property and label properties of a Unit are non-empty, then they SHOULD contain identical Strings.
Reference: Section 13.2.2 on page 95

**sbol-13608** ★ If both of the description property and comment properties of a Unit are non-empty, then they SHOULD contain identical Strings.
Reference: Section 13.2.2 on page 95

**sbol-13609** ★ If both of the comment property and longcomment properties of a Unit are non-empty, then the String contained by the longcomment property SHOULD be longer than the String contained by the comment property.
Reference: Section 13.2.2 on page 95

2.3.0
### *Rules for the SingularUnit class*

**sbol-13701** ☑ A SingularUnit MUST NOT have properties other than the following: identity, persistentIdentity, displayId, version, wasDerivedFroms, wasGeneratedBys, name, description, annotations, attachments, symbol, alternativeSymbols, label, alternativeLabels, comment, longcomment, hasUnit, and hasFactor.
Reference: Section 13.2.3 on page 95

**sbol-13702** ☑ The hasUnit property of a SingularUnit is OPTIONAL and MAY contain a URI.
Reference: Section 13.2.3 on page 95

**sbol-13703** ☑ The hasFactor property of a SingularUnit is OPTIONAL and MAY contain an xsd:float.
Reference: Section 13.2.3 on page 95

**sbol-13704** ☑ Any URI contained by the hasUnit property of a SingularUnit MUST NOT be identical to the URI contained by its identity property.
Reference: Section 13.2.3 on page 95

**sbol-13705** ★ If the hasFactor property of a SingularUnit is non-empty, then its hasUnit property SHOULD also be non-empty.
Reference: Section 13.2.3 on page 95

**sbol-13706** ● The URI contained by the hasUnit property of a SingularUnit MUST refer to a Unit.
Reference: Section 13.2.3 on page 95

2.3.0
### *Rules for the UnitMultiplication class*

**sbol-13801** ☑ A UnitMultiplication MUST NOT have properties other than the following: identity, persistentIdentity, displayId, version, wasDerivedFroms, wasGeneratedBys, name, description, annotations, attachments, symbol, alternativeSymbols, label, alternativeLabels, comment, longcomment, hasTerm1, and hasTerm2.
Reference: Section 13.2.5 on page 96

**sbol-13802** ☑ The hasTerm1 property of a UnitMultiplication is REQUIRED and MUST contain a URI.
Reference: Section 13.2.5 on page 96

**sbol-13803** ☑ The hasTerm2 property of a UnitMultiplication is REQUIRED and MUST contain a URI.
Reference: Section 13.2.5 on page 96

**sbol-13804** ☑ The URI contained by the hasTerm1 property of a UnitMultiplication MUST NOT be identical to the URI contained by its identity property.
Reference: Section 13.2.5 on page 96

**sbol-13805** ☑ The URI contained by the hasTerm2 property of a UnitMultiplication MUST NOT be identical to the URI contained by its identity property.
Reference: Section 13.2.5 on page 96

**sbol-13806** ● The URI contained by the hasTerm1 property of a UnitMultiplication MUST refer to a Unit.
Reference: Section 13.2.5 on page 96

**sbol-13807** ● The URI contained by the hasTerm2 property of a UnitMultiplication MUST refer to a Unit.
Reference: Section 13.2.5 on page 96

2.3.0
### Rules for the UnitDivision class

**sbol-14001** ☑ A UnitDivision MUST NOT have properties other than the following: identity, persistentIdentity, displayId, version, wasDerivedFroms, wasGeneratedBys, name, description, annotations, attachments, symbol, alternativeSymbols, label, alternativeLabels, comment, longcomment, hasNumerator, and hasDenominator.
Reference: Section 13.2.6 on page 97

**sbol-14002** ☑ The hasNumerator property of a UnitDivision is REQUIRED and MUST contain a URI.
Reference: Section 13.2.6 on page 97

**sbol-14003** ☑ The hasDenominator property of a UnitDivision is REQUIRED and MUST contain a URI.
Reference: Section 13.2.6 on page 97

**sbol-14004** ☑ The URI contained by the hasNumerator property of a UnitDivision MUST NOT be identical to the URI contained by its identity property.
Reference: Section 13.2.6 on page 97

**sbol-14005** ☑ The URI contained by the hasDenominator property of a UnitDivision MUST NOT be identical to the URI contained by its identity property.
Reference: Section 13.2.6 on page 97

**sbol-14006** ● The URI contained by the hasNumerator property of a UnitDivision MUST refer to a Unit.
Reference: Section 13.2.6 on page 97

**sbol-14007** ● The URI contained by the hasDenominator property of a UnitDivision MUST refer to a Unit.
Reference: Section 13.2.6 on page 97

2.3.0
### Rules for the UnitExponentiation class

**sbol-14101** ☑ A UnitExponentiation MUST NOT have properties other than the following: identity, persistentIdentity, displayId, version, wasDerivedFroms, wasGeneratedBys, name, description, annotations, attachments, symbol, alternativeSymbols, label, alternativeLabels, comment, longcomment, hasBase, and hasExponent.
Reference: Section 13.2.7 on page 97

**sbol-14102** ☑ The hasBase property of a UnitExponentiation is REQUIRED and MUST contain a URI.
Reference: Section 13.2.7 on page 97

**sbol-14103** ☑ The hasExponent property of a UnitExponentiation is REQUIRED and MUST contain an xsd:integer.
Reference: Section 13.2.7 on page 97

**sbol-14104** ☑ The URI contained by the hasBase property of a UnitExponentiation MUST NOT be identical to the URI contained by its identity property.
Reference: Section 13.2.7 on page 97

**sbol-14105** ● The URI contained by the hasBase property of a UnitExponentiation MUST refer to a Unit.
Reference: Section 13.2.7 on page 97

2.3.0

#### *Rules for the PrefixedUnit class*

**sbol-14201** ☑ A PrefixedUnit MUST NOT have properties other than the following: identity, persistentIdentity, displayId, version, wasDerivedFroms, wasGeneratedBys, name, description, annotations, attachments, symbol, alternativeSymbols, label, alternativeLabels, comment, longcomment, hasUnit, and hasPrefix.
Reference: Section 13.2.8 on page 98

**sbol-14202** ☑ The hasUnit property of a PrefixedUnit is REQUIRED and MUST contain a URI.
Reference: Section 13.2.8 on page 98

**sbol-14203** ☑ The hasPrefix property of a PrefixedUnit is REQUIRED and MUST contain a URI.
Reference: Section 13.2.8 on page 98

**sbol-14204** ☑ The URI contained by the hasUnit property of a PrefixedUnit MUST NOT be identical to the URI contained by its identity property.
Reference: Section 13.2.8 on page 98

**sbol-14205** ● The URI contained by the hasUnit property of a PrefixedUnit MUST refer to a Unit.
Reference: Section 13.2.8 on page 98

**sbol-14206** ● The URI contained by the hasPrefix property of a PrefixedUnit MUST refer to a Prefix.
Reference: Section 13.2.8 on page 98

2.3.0

#### *Rules for the Prefix class*

**sbol-14301** ☑ The symbol property of a Prefix is REQUIRED and MUST contain a String.
Reference: Section 13.2.9 on page 99

**sbol-14302** ☑ The alternativeSymbols property of a Prefix is OPTIONAL and MAY contain a set of Strings.
Reference: Section 13.2.9 on page 99

**sbol-14303** ☑ The label property of a Prefix is REQUIRED and MUST contain a String.
Reference: Section 13.2.9 on page 99

**sbol-14304** ☑ The alternativeLabels property of a Prefix is OPTIONAL and MAY contain a set of Strings.
Reference: Section 13.2.9 on page 99

**sbol-14305** ☑ The comment property of a Prefix is OPTIONAL and MAY contain a String.
Reference: Section 13.2.9 on page 99

**sbol-14306** ☑ The longcomment property of a Prefix is OPTIONAL and MAY contain a String.
Reference: Section 13.2.9 on page 99

**sbol-14307** ☑ The hasFactor property of a Prefix is REQUIRED and MUST contain an xsd:float.
Reference: Section 13.2.9 on page 99

**sbol-14308** ★ If both of the name property and label properties of a Prefix are non-empty, then they SHOULD contain identical Strings.
Reference: Section 13.2.9 on page 99

**sbol-14309** ★ If both of the description property and comment properties of a Prefix are non-empty, then they SHOULD contain identical Strings.
Reference: Section 13.2.9 on page 99

**sbol-14310** ★ If both of the comment property and longcomment properties of a Prefix are non-empty, then the String contained by the longcomment property SHOULD be longer than the String contained by the comment property.
Reference: Section 13.2.9 on page 99

2.3.0

#### Rules for the SIPrefix class

**sbol-14401** ☑ A SIPrefix MUST NOT have properties other than the following: identity, persistentIdentity, displayId, version, wasDerivedFroms, wasGeneratedBys, name, description, annotations, attachments, symbol, alternativeSymbols, label, alternativeLabels, comment, longcomment, and hasFactor.
Reference: Section 13.2.10 on page 99

2.3.0

#### Rules for the BinaryPrefix class

**sbol-14501** ☑ A BinaryPrefix MUST NOT have properties other than the following: identity, persistentIdentity, displayId, version, wasDerivedFroms, wasGeneratedBys, name, description, annotations, attachments, symbol, alternativeSymbols, label, alternativeLabels, comment, longcomment, and hasFactor.
Reference: Section 13.2.11 on page 100

## B Examples of Serialization

### B.1 Simple Examples

#### *B.1.1 Serializing Sequence Objects*

This example shows the serialization of a Sequence.

```
<?xml version="1.0" ?>
<rdf:RDF xmlns:rdf="http://www.w3.org/1999/02/22-rdf-syntax-ns#" xmlns:dcterms="http://purl.org/dc/terms/" xmlns:prov="http://www.
      w3.org/ns/prov#" xmlns:sbol="http://sbols.org/v2#">
  <sbol:Sequence rdf:about="http://partsregistry.org/seq/BBa_J23119">
    <sbol:persistentIdentity rdf:resource="http://partsregistry.org/seq/BBa_J23119"/>
    <sbol:displayId>BBa_J23119</sbol:displayId>
    <prov:wasDerivedFrom rdf:resource="http://parts.igem.org/Part:BBa_J23119:Design"/>
    <sbol:elements>ttgacagctagctcagtcctaggtataatgctagc</sbol:elements>
    <sbol:encoding rdf:resource="http://www.chem.qmul.ac.uk/iubmb/misc/naseq.html"/>
  </sbol:Sequence>
</rdf:RDF>
```

#### *B.1.2 Serializing ComponentDefinition Objects*

This example shows the serialization of a simple promoter ComponentDefinition and the Sequence to which it refers.

```
<?xml version="1.0" ?>
<rdf:RDF xmlns:rdf="http://www.w3.org/1999/02/22-rdf-syntax-ns#" xmlns:dcterms="http://purl.org/dc/terms/" xmlns:prov="http://www.
      w3.org/ns/prov#" xmlns:sbol="http://sbols.org/v2#">
  <sbol:ComponentDefinition rdf:about="http://partsregistry.org/cd/BBa_J23119">
    <sbol:persistentIdentity rdf:resource="http://partsregistry.org/cd/BBa_J23119"/>
    <sbol:displayId>BBa_J23119</sbol:displayId>
    <prov:wasDerivedFrom rdf:resource="http://partsregistry.org/Part:BBa_J23119"/>
    <dcterms:title>J23119 promoter</dcterms:title>
    <dcterms:description>Constitutive promoter</dcterms:description>
    <sbol:type rdf:resource="http://identifiers.org/chebi/CHEBI:4705"/>
    <sbol:type rdf:resource="http://www.biopax.org/release/biopax-level3.owl#DnaRegion"/>
    <sbol:role rdf:resource="http://identifiers.org/so/SO:0000167"/>
    <sbol:role rdf:resource="http://identifiers.org/so/SO:0000613"/>
    <sbol:sequence rdf:resource="http://partsregistry.org/seq/BBa_J23119"/>
  </sbol:ComponentDefinition>
  <sbol:Sequence rdf:about="http://partsregistry.org/seq/BBa_J23119">
    <sbol:persistentIdentity rdf:resource="http://partsregistry.org/seq/BBa_J23119"/>
    <sbol:displayId>BBa_J23119</sbol:displayId>
    <prov:wasDerivedFrom rdf:resource="http://parts.igem.org/Part:BBa_J23119:Design"/>
    <sbol:elements>ttgacagctagctcagtcctaggtataatgctagc</sbol:elements>
    <sbol:encoding rdf:resource="http://www.chem.qmul.ac.uk/iubmb/misc/naseq.html"/>
  </sbol:Sequence>
</rdf:RDF>
```

#### *B.1.3 Serializing SequenceConstraint Objects*

This example shows the serialization of SequenceConstraint between two Component objects in a composite promoter ComponentDefinition. In the example, the promoter ComponentDefinition has two sub-Components that instantiate the ComponentDefinition objects for a core promoter region and and a binding site. The SequenceConstraint specifies that the core promoter region precedes the binding site.

```
<?xml version="1.0" ?>
<rdf:RDF xmlns:rdf="http://www.w3.org/1999/02/22-rdf-syntax-ns#" xmlns:dcterms="http://purl.org/dc/terms/" xmlns:prov="http://www.
      w3.org/ns/prov#" xmlns:sbol="http://sbols.org/v2#">
  <sbol:ComponentDefinition rdf:about="http://partsregistry.org/cd/BBa_K174004">
```

```
    <sbol:persistentIdentity rdf:resource="http://partsregistry.org/cd/BBa_K174004"/>
    <sbol:displayId>BBa_K174004</sbol:displayId>
    <dcterms:title>pspac promoter</dcterms:title>
    <dcterms:description>LacI repressible promoter</dcterms:description>
    <sbol:type rdf:resource="http://www.biopax.org/release/biopax-level3.owl#DnaRegion"/>
    <sbol:role rdf:resource="http://identifiers.org/so/SO:0000167"/>
    <sbol:sequenceConstraint>
      <sbol:SequenceConstraint rdf:about="http://partsregistry.org/cd/BBa_K174004/r1">
        <sbol:persistentIdentity rdf:resource="http://partsregistry.org/cd/BBa_K174004/r1"/>
        <sbol:displayId>r1</sbol:displayId>
        <sbol:restriction rdf:resource="http://sbols.org/v2#precedes"/>
        <sbol:subject rdf:resource="http://partsregistry.org/cd/pspac"/>
        <sbol:object rdf:resource="http://partsregistry.org/cd/LacI_operator"/>
      </sbol:SequenceConstraint>
    </sbol:sequenceConstraint>
  </sbol:ComponentDefinition>
  <sbol:ComponentDefinition rdf:about="http://partsregistry.org/cd/pspac">
    <sbol:persistentIdentity rdf:resource="http://partsregistry.org/cd/pspac"/>
    <sbol:displayId>pspac</sbol:displayId>
    <dcterms:title>constitutive promoter</dcterms:title>
    <dcterms:description>pspac core promoter region</dcterms:description>
    <sbol:type rdf:resource="http://www.biopax.org/release/biopax-level3.owl#DnaRegion"/>
    <sbol:role rdf:resource="http://identifiers.org/so/SO:0000167"/>
  </sbol:ComponentDefinition>
  <sbol:ComponentDefinition rdf:about="http://partsregistry.org/cd/LacI_operator">
    <sbol:persistentIdentity rdf:resource="http://partsregistry.org/cd/LacI_operator"/>
    <sbol:displayId>LacI_operator</sbol:displayId>
    <dcterms:title>LacI operator</dcterms:title>
    <dcterms:description>LacI binding site</dcterms:description>
    <sbol:type rdf:resource="http://www.biopax.org/release/biopax-level3.owl#DnaRegion"/>
    <sbol:role rdf:resource="http://identifiers.org/so/SO:0000057"/>
  </sbol:ComponentDefinition>
</rdf:RDF>
```

#### B.1.4 Serializing Cut Location Objects

This example shows the serialization of a `Cut Location`.

```
<?xml version="1.0" ?>
<rdf:RDF xmlns:rdf="http://www.w3.org/1999/02/22-rdf-syntax-ns#" xmlns:dcterms="http://purl.org/dc/terms/" xmlns:prov="http://www.
      w3.org/ns/prov#" xmlns:sbol="http://sbols.org/v2#">
  <sbol:ComponentDefinition rdf:about="http://partsregistry.org/cd/BBa_J23119">
    <sbol:persistentIdentity rdf:resource="http://partsregistry.org/cd/BBa_J23119"/>
    <sbol:displayId>BBa_J23119</sbol:displayId>
    <prov:wasDerivedFrom rdf:resource="http://partsregistry.org/Part:BBa_J23119"/>
    <dcterms:title>J23119 promoter</dcterms:title>
    <dcterms:description>Constitutive promoter</dcterms:description>
    <sbol:type rdf:resource="http://www.biopax.org/release/biopax-level3.owl#DnaRegion"/>
    <sbol:role rdf:resource="http://identifiers.org/so/SO:0000167"/>
    <sbol:role rdf:resource="http://identifiers.org/so/SO:0000613"/>
    <sbol:sequenceAnnotation>
      <sbol:SequenceAnnotation rdf:about="http://partsregistry.org/cd/BBa_J23119/cutat10">
        <sbol:persistentIdentity rdf:resource="http://partsregistry.org/cd/BBa_J23119/cutat10"/>
        <sbol:displayId>cutat10</sbol:displayId>
        <sbol:location>
          <sbol:Cut rdf:about="http://partsregistry.org/cd/BBa_J23119/cutat10/cut1">
            <sbol:persistentIdentity rdf:resource="http://partsregistry.org/cd/BBa_J23119/cutat10/cut1"/>
            <sbol:displayId>cut1</sbol:displayId>
            <sbol:at>10</sbol:at>
            <sbol:orientation rdf:resource="http://sbols.org/v2#inline"/>
          </sbol:Cut>
        </sbol:location>
      </sbol:SequenceAnnotation>
    </sbol:sequenceAnnotation>
    <sbol:sequenceAnnotation>
      <sbol:SequenceAnnotation rdf:about="http://partsregistry.org/cd/BBa_J23119/cutat12">
        <sbol:persistentIdentity rdf:resource="http://partsregistry.org/cd/BBa_J23119/cutat12"/>
```

```
        <sbol:displayId>cutat12</sbol:displayId>
        <sbol:location>
          <sbol:Cut rdf:about="http://partsregistry.org/cd/BBa_J23119/cutat12/cut2">
            <sbol:persistentIdentity rdf:resource="http://partsregistry.org/cd/BBa_J23119/cutat12/cut2"/>
            <sbol:displayId>cut2</sbol:displayId>
            <sbol:at>12</sbol:at>
            <sbol:orientation rdf:resource="http://sbols.org/v2#inline"/>
          </sbol:Cut>
        </sbol:location>
      </sbol:SequenceAnnotation>
    </sbol:sequenceAnnotation>
    <sbol:sequence rdf:resource="http://partsregistry.org/seq/BBa_J23119"/>
  </sbol:ComponentDefinition>
  <sbol:Sequence rdf:about="http://partsregistry.org/seq/BBa_J23119">
    <sbol:persistentIdentity rdf:resource="http://partsregistry.org/seq/BBa_J23119"/>
    <sbol:displayId>BBa_J23119</sbol:displayId>
    <prov:wasDerivedFrom rdf:resource="http://parts.igem.org/Part:BBa_J23119:Design"/>
    <sbol:elements>ttgacagctagctcagtcctaggtataatgctagc</sbol:elements>
    <sbol:encoding rdf:resource="http://www.chem.qmul.ac.uk/iubmb/misc/naseq.html"/>
  </sbol:Sequence>
</rdf:RDF>
```

#### B.1.5 Serializing Model Objects

This example shows the serialization of a `Model`. In this example, the `Model` refers to an ODE model written in SBML that can be accessed the identified source repository.

```
<?xml version="1.0" ?>
<rdf:RDF xmlns:rdf="http://www.w3.org/1999/02/22-rdf-syntax-ns#" xmlns:dcterms="http://purl.org/dc/terms/" xmlns:prov="http://www.
      w3.org/ns/prov#" xmlns:sbol="http://sbols.org/v2#">
  <sbol:Model rdf:about="http://www.sbolstandard.org/examples/pIKE_Toggle_1">
    <sbol:persistentIdentity rdf:resource="http://www.sbolstandard.org/examples/pIKE_Toggle_1"/>
    <sbol:displayId>pIKE_Toggle_1</sbol:displayId>
    <dcterms:title>pIKE_Toggle_1 toggle switch</dcterms:title>
    <sbol:source rdf:resource="http://virtualparts.org/part/pIKE_Toggle_1"/>
    <sbol:language rdf:resource="http://identifiers.org/edam/format_2585"/>
    <sbol:framework rdf:resource="http://identifiers.org/biomodels.sbo/SBO:0000062"/>
  </sbol:Model>
</rdf:RDF>
```

#### B.1.6 Serializing ModuleDefinition Objects

This example shows the serialization of a simple `ModuleDefinition`. This `ModuleDefinition` includes an `Interaction` that represents the translation of a protein.

```
<?xml version="1.0" ?>
<rdf:RDF xmlns:rdf="http://www.w3.org/1999/02/22-rdf-syntax-ns#" xmlns:dcterms="http://purl.org/dc/terms/" xmlns:prov="http://www.
      w3.org/ns/prov#" xmlns:sbol="http://sbols.org/v2#">
  <sbol:ModuleDefinition rdf:about="http://sbolstandard.org/example/md/GFP_expression">
    <sbol:persistentIdentity rdf:resource="http://sbolstandard.org/example/md/GFP_expression"/>
    <sbol:displayId>GFP_expression</sbol:displayId>
    <sbol:functionalComponent>
      <sbol:FunctionalComponent rdf:about="http://sbolstandard.org/example/md/GFP_expression/Constitutive_GFP">
        <sbol:persistentIdentity rdf:resource="http://sbolstandard.org/example/md/GFP_expression/Constitutive_GFP"/>
        <sbol:displayId>Constitutive_GFP</sbol:displayId>
        <sbol:definition rdf:resource="http://sbolstandard.org/example/GFP_generator"/>
        <sbol:access rdf:resource="http://sbols.org/v2#public"/>
        <sbol:direction rdf:resource="http://sbols.org/v2#in"/>
      </sbol:FunctionalComponent>
    </sbol:functionalComponent>
    <sbol:functionalComponent>
      <sbol:FunctionalComponent rdf:about="http://sbolstandard.org/example/md/GFP_expression/GFP_protein">
        <sbol:persistentIdentity rdf:resource="http://sbolstandard.org/example/md/GFP_expression/GFP_protein"/>
        <sbol:displayId>GFP_protein</sbol:displayId>
```

```
        <sbol:definition rdf:resource="http://sbolstandard.org/example/GFP"/>
        <sbol:access rdf:resource="http://sbols.org/v2#public"/>
        <sbol:direction rdf:resource="http://sbols.org/v2#out"/>
      </sbol:FunctionalComponent>
    </sbol:functionalComponent>
    <sbol:interaction>
      <sbol:Interaction rdf:about="http://sbolstandard.org/example/md/GFP_expression/express_GFP">
        <sbol:persistentIdentity rdf:resource="http://sbolstandard.org/example/md/GFP_expression/express_GFP"/>
        <sbol:displayId>express_GFP</sbol:displayId>
        <sbol:type rdf:resource="Transcription"/>
        <sbol:participation>
          <sbol:Participation rdf:about="http://sbolstandard.org/example/md/GFP_expression/express_GFP/Protein">
            <sbol:persistentIdentity rdf:resource="http://sbolstandard.org/example/md/GFP_expression/express_GFP/Protein"/>
            <sbol:displayId>Protein</sbol:displayId>
            <sbol:participant rdf:resource="http://sbolstandard.org/example/md/GFP_expression/GFP_protein"/>
          </sbol:Participation>
        </sbol:participation>
        <sbol:participation>
          <sbol:Participation rdf:about="http://sbolstandard.org/example/md/GFP_expression/express_GFP/CDS">
            <sbol:persistentIdentity rdf:resource="http://sbolstandard.org/example/md/GFP_expression/express_GFP/CDS"/>
            <sbol:displayId>CDS</sbol:displayId>
            <sbol:participant rdf:resource="http://sbolstandard.org/example/md/GFP_expression/Constitutive_GFP"/>
          </sbol:Participation>
        </sbol:participation>
      </sbol:Interaction>
    </sbol:interaction>
  </sbol:ModuleDefinition>
</rdf:RDF>
</rdf:RDF>
```

#### B.1.7 Serializing Application Specific Data Within SBOL Objects

This example shows the serialization of application-specific data from `Annotation` objects. A `ComponentDefinition` that represents a promoter is annotated with custom data on the promoter's sigma factor and how it is regulated.

```
<?xml version="1.0" ?>
<rdf:RDF xmlns:pr="http://partsregistry.org/" xmlns:rdf="http://www.w3.org/1999/02/22-rdf-syntax-ns#" xmlns:dcterms="http://purl.
      org/dc/terms/" xmlns:prov="http://www.w3.org/ns/prov#" xmlns:sbol="http://sbols.org/v2#">
  <sbol:ComponentDefinition rdf:about="http://partsregistry.org/cd/BBa_J23119">
    <sbol:persistentIdentity rdf:resource="http://partsregistry.org/cd/BBa_J23119"/>
    <sbol:displayId>BBa_J23119</sbol:displayId>
    <pr:group>iGEM2006_Berkeley</pr:group>
    <pr:experience rdf:resource="http://parts.igem.org/Part:BBa_J23119:Experience"/>
    <pr:information>
      <pr:Information rdf:about="http://partsregistry.org/cd/BBa_J23119/information">
        <pr:sigmafactor>//rnap/prokaryote/ecoli/sigma70</pr:sigmafactor>
        <pr:regulation>//regulation/constitutive</pr:regulation>
      </pr:Information>
    </pr:information>
    <dcterms:title>J23119</dcterms:title>
    <dcterms:description>Constitutive promoter</dcterms:description>
    <sbol:type rdf:resource="http://www.biopax.org/release/biopax-level3.owl#DnaRegion"/>
    <sbol:role rdf:resource="http://identifiers.org/so/SO:0000167"/>
  </sbol:ComponentDefinition>
</rdf:RDF>
```

#### B.1.8 Serializing Application-Specific Data Outside SBOL Objects

This example shows the serialization of application-specific data from `GenericTopLevel` objects. Such data can be referenced by other SBOL objects via `Annotation` objects.

```
<?xml version="1.0" ?>
<rdf:RDF xmlns:myapp="http://www.myapp.org/" xmlns:rdf="http://www.w3.org/1999/02/22-rdf-syntax-ns#" xmlns:dcterms="http://purl.
```

```
      org/dc/terms/" xmlns:prov="http://www.w3.org/ns/prov#" xmlns:sbol="http://sbols.org/v2#">
  <sbol:ComponentDefinition rdf:about="http://www.partsregistry.org/cd/BBa_J23119">
    <sbol:persistentIdentity rdf:resource="http://www.partsregistry.org/cd/BBa_J23119"/>
    <sbol:displayId>BBa_J23119</sbol:displayId>
    <prov:wasDerivedFrom rdf:resource="http://www.partsregistry.org/Part:BBa_J23119"/>
    <myapp:datasheet rdf:resource="http://www.partsregistry.org/gen/datasheet1"/>
    <dcterms:title>J23119</dcterms:title>
    <dcterms:description>Constitutive promoter</dcterms:description>
    <sbol:type rdf:resource="http://www.biopax.org/release/biopax-level3.owl#DnaRegion"/>
    <sbol:role rdf:resource="http://identifiers.org/so/SO:0000167"/>
  </sbol:ComponentDefinition>
  <myapp:Datasheet rdf:about="http://www.partsregistry.org/gen/datasheet1">
    <sbol:persistentIdentity rdf:resource="http://www.partsregistry.org/gen/datasheet1"/>
    <sbol:displayId>datasheet1</sbol:displayId>
    <myapp:characterizationData rdf:resource="http://www.myapp.org/measurement/1"/>
    <myapp:transcriptionRate>1</myapp:transcriptionRate>
    <dcterms:title>Datasheet 1</dcterms:title>
  </myapp:Datasheet>
</rdf:RDF>
```

#### B.1.9 Serializing Collection Objects

This example shows the serialization of a `Collection`. This `Collection` represents a library of promoters.

```
<?xml version="1.0" ?>
<rdf:RDF xmlns:rdf="http://www.w3.org/1999/02/22-rdf-syntax-ns#" xmlns:dcterms="http://purl.org/dc/terms/" xmlns:prov="http://www.
      w3.org/ns/prov#" xmlns:sbol="http://sbols.org/v2#">
  <sbol:Collection rdf:about="http://parts.igem.org/Promoters/Catalog/Anderson">
    <sbol:persistentIdentity rdf:resource="http://parts.igem.org/Promoters/Catalog/Anderson"/>
    <sbol:displayId>Anderson</sbol:displayId>
    <dcterms:title>Anderson promoters</dcterms:title>
    <dcterms:description>The Anderson promoter collection</dcterms:description>
    <sbol:member rdf:resource="http://partsregistry.org/Part:BBa_J23119"/>
    <sbol:member rdf:resource="http://partsregistry.org/Part:BBa_J23118"/>
  </sbol:Collection>
</rdf:RDF>
```

### B.2 Complex Examples

#### B.2.1 PoPS Receiver

This example shows the serialization of the PoPS Receiver device designed by Canton and co-workers Canton et al. (2008). In particular, this example includes the serialization of a `ComponentDefinition` that composes five `Component` objects to define the structure of a detector for the cell-cell signaling molecule 3OC$_6$HSL. The five components are arranged in a sequence: first come four `Component` objects that together implement constitutive expression of the LuxR protein, which in turn responds to 3OC$_6$HSL: a constitutive promoter, 5'UTR, coding sequence for LuxR, and terminator. Finally, after these objects comes the `Component` object for the pLuxR promoter, which is activated in the presence of LuxR and 3OC$_6$HSL. Complete details of the device can be found in the cited paper and also at http://parts.igem.org/Part:BBa_F2620.

```
<?xml version="1.0" ?>
<rdf:RDF xmlns:pr="http://partsregistry.org/" xmlns:rdf="http://www.w3.org/1999/02/22-rdf-syntax-ns#" xmlns:dcterms="http://purl.
      org/dc/terms/" xmlns:prov="http://www.w3.org/ns/prov#" xmlns:sbol="http://sbols.org/v2#">
  <sbol:ComponentDefinition rdf:about="http://partsregistry.org/cd/BBa_F2620">
    <sbol:persistentIdentity rdf:resource="http://partsregistry.org/cd/BBa_F2620"/>
    <sbol:displayId>BBa_F2620</sbol:displayId>
    <dcterms:title>BBa_F2620</dcterms:title>
    <dcterms:description>3OC6HSL -&gt; PoPS Receiver</dcterms:description>
    <sbol:type rdf:resource="http://www.biopax.org/release/biopax-level3.owl#DnaRegion"/>
    <sbol:role rdf:resource="http://identifiers.org/so/SO:00001411"/>
    <sbol:component>
      <sbol:Component rdf:about="http://partsregistry.org/cd/BBa_F2620/pLuxR">
```

```
        <sbol:persistentIdentity rdf:resource="http://partsregistry.org/cd/BBa_F2620/pLuxR"/>
        <sbol:displayId>pLuxR</sbol:displayId>
        <sbol:access rdf:resource="http://sbols.org/v2#public"/>
        <sbol:definition rdf:resource="http://partsregistry.org/cd/BBa_R0062"/>
      </sbol:Component>
    </sbol:component>
    <sbol:component>
      <sbol:Component rdf:about="http://partsregistry.org/cd/BBa_F2620/luxR">
        <sbol:persistentIdentity rdf:resource="http://partsregistry.org/cd/BBa_F2620/luxR"/>
        <sbol:displayId>luxR</sbol:displayId>
        <sbol:access rdf:resource="http://sbols.org/v2#public"/>
        <sbol:definition rdf:resource="http://partsregistry.org/cd/BBa_C0062"/>
      </sbol:Component>
    </sbol:component>
    <sbol:component>
      <sbol:Component rdf:about="http://partsregistry.org/cd/BBa_F2620/pTetR">
        <sbol:persistentIdentity rdf:resource="http://partsregistry.org/cd/BBa_F2620/pTetR"/>
        <sbol:displayId>pTetR</sbol:displayId>
        <sbol:access rdf:resource="http://sbols.org/v2#public"/>
        <sbol:definition rdf:resource="http://partsregistry.org/cd/BBa_R0040"/>
      </sbol:Component>
    </sbol:component>
    <sbol:component>
      <sbol:Component rdf:about="http://partsregistry.org/cd/BBa_F2620/ter">
        <sbol:persistentIdentity rdf:resource="http://partsregistry.org/cd/BBa_F2620/ter"/>
        <sbol:displayId>ter</sbol:displayId>
        <sbol:access rdf:resource="http://sbols.org/v2#public"/>
        <sbol:definition rdf:resource="http://partsregistry.org/cd/BBa_B0015"/>
      </sbol:Component>
    </sbol:component>
    <sbol:component>
      <sbol:Component rdf:about="http://partsregistry.org/cd/BBa_F2620/rbs">
        <sbol:persistentIdentity rdf:resource="http://partsregistry.org/cd/BBa_F2620/rbs"/>
        <sbol:displayId>rbs</sbol:displayId>
        <sbol:access rdf:resource="http://sbols.org/v2#public"/>
        <sbol:definition rdf:resource="http://partsregistry.org/cd/BBa_B0034"/>
      </sbol:Component>
    </sbol:component>
    <sbol:sequenceAnnotation>
      <sbol:SequenceAnnotation rdf:about="http://partsregistry.org/cd/BBa_F2620/anno3">
        <sbol:persistentIdentity rdf:resource="http://partsregistry.org/cd/BBa_F2620/anno3"/>
        <sbol:displayId>anno3</sbol:displayId>
        <sbol:location>
          <sbol:Range rdf:about="http://partsregistry.org/cd/BBa_F2620/anno3/location3">
            <sbol:persistentIdentity rdf:resource="http://partsregistry.org/cd/BBa_F2620/anno3/location3"/>
            <sbol:displayId>location3</sbol:displayId>
            <sbol:start>69</sbol:start>
            <sbol:end>770</sbol:end>
            <sbol:orientation rdf:resource="http://sbols.org/v2#inline"/>
          </sbol:Range>
        </sbol:location>
        <sbol:component rdf:resource="http://partsregistry.org/cd/BBa_F2620/luxR"/>
      </sbol:SequenceAnnotation>
    </sbol:sequenceAnnotation>
    <sbol:sequenceAnnotation>
      <sbol:SequenceAnnotation rdf:about="http://partsregistry.org/cd/BBa_F2620/anno5">
        <sbol:persistentIdentity rdf:resource="http://partsregistry.org/cd/BBa_F2620/anno5"/>
        <sbol:displayId>anno5</sbol:displayId>
        <sbol:location>
          <sbol:Range rdf:about="http://partsregistry.org/cd/BBa_F2620/anno5/location5">
            <sbol:persistentIdentity rdf:resource="http://partsregistry.org/cd/BBa_F2620/anno5/location5"/>
            <sbol:displayId>location5</sbol:displayId>
            <sbol:start>901</sbol:start>
            <sbol:end>956</sbol:end>
            <sbol:orientation rdf:resource="http://sbols.org/v2#inline"/>
          </sbol:Range>
        </sbol:location>
        <sbol:component rdf:resource="http://partsregistry.org/cd/BBa_F2620/pLuxR"/>
      </sbol:SequenceAnnotation>
```

```
    </sbol:sequenceAnnotation>
    <sbol:sequenceAnnotation>
      <sbol:SequenceAnnotation rdf:about="http://partsregistry.org/cd/BBa_F2620/anno1">
        <sbol:persistentIdentity rdf:resource="http://partsregistry.org/cd/BBa_F2620/anno1"/>
        <sbol:displayId>anno1</sbol:displayId>
        <sbol:location>
          <sbol:Range rdf:about="http://partsregistry.org/cd/BBa_F2620/anno1/location1">
            <sbol:persistentIdentity rdf:resource="http://partsregistry.org/cd/BBa_F2620/anno1/location1"/>
            <sbol:displayId>location1</sbol:displayId>
            <sbol:start>1</sbol:start>
            <sbol:end>55</sbol:end>
            <sbol:orientation rdf:resource="http://sbols.org/v2#inline"/>
          </sbol:Range>
        </sbol:location>
        <sbol:component rdf:resource="http://partsregistry.org/cd/BBa_F2620/pTetR"/>
      </sbol:SequenceAnnotation>
    </sbol:sequenceAnnotation>
    <sbol:sequenceAnnotation>
      <sbol:SequenceAnnotation rdf:about="http://partsregistry.org/cd/BBa_F2620/anno4">
        <sbol:persistentIdentity rdf:resource="http://partsregistry.org/cd/BBa_F2620/anno4"/>
        <sbol:displayId>anno4</sbol:displayId>
        <sbol:location>
          <sbol:Range rdf:about="http://partsregistry.org/cd/BBa_F2620/anno4/location4">
            <sbol:persistentIdentity rdf:resource="http://partsregistry.org/cd/BBa_F2620/anno4/location4"/>
            <sbol:displayId>location4</sbol:displayId>
            <sbol:start>771</sbol:start>
            <sbol:end>900</sbol:end>
            <sbol:orientation rdf:resource="http://sbols.org/v2#inline"/>
          </sbol:Range>
        </sbol:location>
        <sbol:component rdf:resource="http://partsregistry.org/cd/BBa_F2620/ter"/>
      </sbol:SequenceAnnotation>
    </sbol:sequenceAnnotation>
    <sbol:sequenceAnnotation>
      <sbol:SequenceAnnotation rdf:about="http://partsregistry.org/cd/BBa_F2620/anno2">
        <sbol:persistentIdentity rdf:resource="http://partsregistry.org/cd/BBa_F2620/anno2"/>
        <sbol:displayId>anno2</sbol:displayId>
        <sbol:location>
          <sbol:Range rdf:about="http://partsregistry.org/cd/BBa_F2620/anno2/location2">
            <sbol:persistentIdentity rdf:resource="http://partsregistry.org/cd/BBa_F2620/anno2/location2"/>
            <sbol:displayId>location2</sbol:displayId>
            <sbol:start>56</sbol:start>
            <sbol:end>68</sbol:end>
            <sbol:orientation rdf:resource="http://sbols.org/v2#inline"/>
          </sbol:Range>
        </sbol:location>
        <sbol:component rdf:resource="http://partsregistry.org/cd/BBa_F2620/rbs"/>
      </sbol:SequenceAnnotation>
    </sbol:sequenceAnnotation>
  </sbol:ComponentDefinition>
  <sbol:ComponentDefinition rdf:about="http://partsregistry.org/cd/BBa_R0062">
    <sbol:persistentIdentity rdf:resource="http://partsregistry.org/cd/BBa_R0062"/>
    <sbol:displayId>BBa_R0062</sbol:displayId>
    <dcterms:title>pLuxR</dcterms:title>
    <dcterms:description>LuxR inducible promoter</dcterms:description>
    <sbol:type rdf:resource="http://www.biopax.org/release/biopax-level3.owl#DnaRegion"/>
    <sbol:role rdf:resource="http://identifiers.org/so/SO:0000167"/>
    <sbol:sequence rdf:resource="http://partsregistry.org/seq/BBa_R0062"/>
  </sbol:ComponentDefinition>
  <sbol:ComponentDefinition rdf:about="http://partsregistry.org/cd/BBa_B0015">
    <sbol:persistentIdentity rdf:resource="http://partsregistry.org/cd/BBa_B0015"/>
    <sbol:displayId>BBa_B0015</sbol:displayId>
    <dcterms:title>BBa_B0015</dcterms:title>
    <dcterms:description>Double terminator</dcterms:description>
    <sbol:type rdf:resource="http://www.biopax.org/release/biopax-level3.owl#DnaRegion"/>
    <sbol:role rdf:resource="http://identifiers.org/so/SO:0000141"/>
    <sbol:sequence rdf:resource="http://partsregistry.org/seq/BBa_B0015"/>
  </sbol:ComponentDefinition>
  <sbol:ComponentDefinition rdf:about="http://partsregistry.org/cd/BBa_C0062">
```

```
    <sbol:persistentIdentity rdf:resource="http://partsregistry.org/cd/BBa_C0062"/>
    <sbol:displayId>BBa_C0062</sbol:displayId>
    <dcterms:title>luxR</dcterms:title>
    <dcterms:description>luxR coding sequence</dcterms:description>
    <sbol:type rdf:resource="http://www.biopax.org/release/biopax-level3.owl#DnaRegion"/>
    <sbol:role rdf:resource="http://identifiers.org/so/SO:0000316"/>
    <sbol:sequence rdf:resource="http://partsregistry.org/seq/BBa_C0062"/>
  </sbol:ComponentDefinition>
  <sbol:ComponentDefinition rdf:about="http://partsregistry.org/cd/BBa_R0040">
    <sbol:persistentIdentity rdf:resource="http://partsregistry.org/cd/BBa_R0040"/>
    <sbol:displayId>BBa_R0040</sbol:displayId>
    <dcterms:title>pTetR</dcterms:title>
    <dcterms:description>TetR repressible promoter</dcterms:description>
    <sbol:type rdf:resource="http://www.biopax.org/release/biopax-level3.owl#DnaRegion"/>
    <sbol:role rdf:resource="http://identifiers.org/so/SO:0000167"/>
    <sbol:sequence rdf:resource="http://partsregistry.org/seq/BBa_R0040"/>
  </sbol:ComponentDefinition>
  <sbol:ComponentDefinition rdf:about="http://partsregistry.org/cd/BBa_B0034">
    <sbol:persistentIdentity rdf:resource="http://partsregistry.org/cd/BBa_B0034"/>
    <sbol:displayId>BBa_B0034</sbol:displayId>
    <dcterms:title>BBa_B0034</dcterms:title>
    <dcterms:description>RBS based on Elowitz repressilator</dcterms:description>
    <sbol:type rdf:resource="http://www.biopax.org/release/biopax-level3.owl#DnaRegion"/>
    <sbol:role rdf:resource="http://identifiers.org/so/SO:0000139"/>
    <sbol:sequence rdf:resource="http://partsregistry.org/seq/BBa_B0034"/>
  </sbol:ComponentDefinition>
  <sbol:Sequence rdf:about="http://partsregistry.org/seq/BBa_B0034">
    <sbol:persistentIdentity rdf:resource="http://partsregistry.org/seq/BBa_B0034"/>
    <sbol:displayId>BBa_B0034</sbol:displayId>
    <sbol:elements>aaagaggagaaa</sbol:elements>
    <sbol:encoding rdf:resource="http://www.chem.qmul.ac.uk/iubmb/misc/naseq.html"/>
  </sbol:Sequence>
  <sbol:Sequence rdf:about="http://partsregistry.org/seq/BBa_R0040">
    <sbol:persistentIdentity rdf:resource="http://partsregistry.org/seq/BBa_R0040"/>
    <sbol:displayId>BBa_R0040</sbol:displayId>
    <sbol:elements>tccctatcagtgatagagattgacatccctatcagtgatagagatactgagcac</sbol:elements>
    <sbol:encoding rdf:resource="http://www.chem.qmul.ac.uk/iubmb/misc/naseq.html"/>
  </sbol:Sequence>
  <sbol:Sequence rdf:about="http://partsregistry.org/seq/BBa_C0062">
    <sbol:persistentIdentity rdf:resource="http://partsregistry.org/seq/BBa_C0062"/>
    <sbol:displayId>BBa_C0062</sbol:displayId>
    <sbol:elements>atgcttatctgatatgactaaaatggtacattgtgaatattatttactcgcgatcatttatcctcattcta
    tggttaaatctgatatttcaatcctagataattaccctaaaaaatggaggcaatattatgatgacgctaatttaataaaatatgat
    cctatagtagattattctaactccaatcattcaccaattaattggaatatatttgaaaacaatgctgtaaataaaaaatctccaaa
    tgtaattaaagaagcgaaaacatcaggtcttatcactgggtttagtttccctattcatacggctaacaatggcttcggaatgctta
    gttttgcacattcagaaaaagacaactatatagatagtttatttttacatgcgtgtatgaacataccattaattgttccttctcta
    gttgataattatcgaaaaataaatatagcaaataataaatcaaacaacgatttaaccaaaagagaaaaagaatgtttagcgtgggc
    atgcgaaggaaaaagctcttgggatatttcaaaaatattaggttgcagtgagcgtactgtcactttccatttaaccaatgcgcaaa
    tgaaactcaatacaacaaaccgctgccaaagtatttctaaagcaattttaacaggagcaattgattgcccatactttaaaaattaa
    taacactgatagtgctagtgtagatcac</sbol:elements>
    <sbol:encoding rdf:resource="http://www.chem.qmul.ac.uk/iubmb/misc/naseq.html"/>
  </sbol:Sequence>
  <sbol:Sequence rdf:about="http://partsregistry.org/seq/BBa_R0062">
    <sbol:persistentIdentity rdf:resource="http://partsregistry.org/seq/BBa_R0062"/>
    <sbol:displayId>BBa_R0062</sbol:displayId>
    <sbol:elements>acctgtaggatcgtacaggtttacgcaagaaaatggtttgttatagtcgaataaa</sbol:elements>
    <sbol:encoding rdf:resource="http://www.chem.qmul.ac.uk/iubmb/misc/naseq.html"/>
  </sbol:Sequence>
  <sbol:Sequence rdf:about="http://partsregistry.org/seq/BBa_B0015">
    <sbol:persistentIdentity rdf:resource="http://partsregistry.org/seq/BBa_B0015"/>
    <sbol:displayId>BBa_B0015</sbol:displayId>
    <sbol:elements>ccaggcatcaaataaaacgaaaggctcagtcgaaagactgggcctttcgttttatctgttgtttgtcggtg
    aacgctctctactagagtcacactggctcaccttcgggtgggcctttctgcgtttata</sbol:elements>
    <sbol:encoding rdf:resource="http://www.chem.qmul.ac.uk/iubmb/misc/naseq.html"/>
  </sbol:Sequence>
</rdf:RDF>
```

#### *B.2.2 Toggle Switch*

This example shows the serialization of the `ComponentDefinition` and `ModuleDefinition` objects for a LacI/TetR toggle switch similar to those constructed in Gardner et al. (2000). This design is essentially similar to the one presented in Section 9, except that it uses some alternate groupings in how the total design is built up out of smaller entities.

```
<?xml version="1.0" ?>
<rdf:RDF xmlns:rdf="http://www.w3.org/1999/02/22-rdf-syntax-ns#" xmlns:dcterms="http://purl.org/dc/terms/" xmlns:prov="http://www.
     w3.org/ns/prov#" xmlns:sbol="http://sbols.org/v2#">
  <sbol:ModuleDefinition rdf:about="http://sbolstandard.org/example/toggle_switch">
    <sbol:persistentIdentity rdf:resource="http://sbolstandard.org/example/toggle_switch"/>
    <sbol:displayId>toggle_switch</sbol:displayId>
    <sbol:role rdf:resource="http://sbolstandard.org/example/module_role/toggle_switch"/>
    <sbol:functionalComponent>
      <sbol:FunctionalComponent rdf:about="http://sbolstandard.org/example/toggle_switch/TetR">
        <sbol:persistentIdentity rdf:resource="http://sbolstandard.org/example/toggle_switch/TetR"/>
        <sbol:displayId>TetR</sbol:displayId>
        <sbol:definition rdf:resource="http://identifiers.org/uniprot/Q6QR72"/>
        <sbol:access rdf:resource="http://sbols.org/v2#public"/>
        <sbol:direction rdf:resource="http://sbols.org/v2#inout"/>
      </sbol:FunctionalComponent>
    </sbol:functionalComponent>
    <sbol:functionalComponent>
      <sbol:FunctionalComponent rdf:about="http://sbolstandard.org/example/toggle_switch/LacI">
        <sbol:persistentIdentity rdf:resource="http://sbolstandard.org/example/toggle_switch/LacI"/>
        <sbol:displayId>LacI</sbol:displayId>
        <sbol:definition rdf:resource="http://identifiers.org/uniprot/P03023"/>
        <sbol:access rdf:resource="http://sbols.org/v2#public"/>
        <sbol:direction rdf:resource="http://sbols.org/v2#inout"/>
      </sbol:FunctionalComponent>
    </sbol:functionalComponent>
    <sbol:model rdf:resource="http://sbolstandard.org/example/toogleswitch"/>
    <sbol:module>
      <sbol:Module rdf:about="http://sbolstandard.org/example/toggle_switch/tetr_inverter">
        <sbol:persistentIdentity rdf:resource="http://sbolstandard.org/example/toggle_switch/tetr_inverter"/>
        <sbol:displayId>tetr_inverter</sbol:displayId>
        <sbol:definition rdf:resource="http://sbolstandard.org/example/tetr_inverter"/>
        <sbol:mapsTo>
          <sbol:MapsTo rdf:about="http://sbolstandard.org/example/toggle_switch/tetr_inverter/TetR_mapping">
            <sbol:persistentIdentity rdf:resource="http://sbolstandard.org/example/toggle_switch/tetr_inverter/TetR_mapping"/>
            <sbol:displayId>TetR_mapping</sbol:displayId>
            <sbol:refinement rdf:resource="http://sbols.org/v2#useRemote"/>
            <sbol:remote rdf:resource="http://sbolstandard.org/example/tetr_inverter/TF"/>
            <sbol:local rdf:resource="http://sbolstandard.org/example/toggle_switch/TetR"/>
          </sbol:MapsTo>
        </sbol:mapsTo>
      </sbol:Module>
    </sbol:module>
    <sbol:module>
      <sbol:Module rdf:about="http://sbolstandard.org/example/toggle_switch/laci_inverter">
        <sbol:persistentIdentity rdf:resource="http://sbolstandard.org/example/toggle_switch/laci_inverter"/>
        <sbol:displayId>laci_inverter</sbol:displayId>
        <sbol:definition rdf:resource="http://sbolstandard.org/example/laci_inverter"/>
        <sbol:mapsTo>
          <sbol:MapsTo rdf:about="http://sbolstandard.org/example/toggle_switch/laci_inverter/LacI_mapping">
            <sbol:persistentIdentity rdf:resource="http://sbolstandard.org/example/toggle_switch/laci_inverter/LacI_mapping"/>
            <sbol:displayId>LacI_mapping</sbol:displayId>
            <sbol:refinement rdf:resource="http://sbols.org/v2#useRemote"/>
            <sbol:remote rdf:resource="http://sbolstandard.org/example/laci_inverter/TF"/>
            <sbol:local rdf:resource="http://sbolstandard.org/example/toggle_switch/LacI"/>
          </sbol:MapsTo>
        </sbol:mapsTo>
      </sbol:Module>
    </sbol:module>
  </sbol:ModuleDefinition>
  <sbol:ModuleDefinition rdf:about="http://sbolstandard.org/example/laci_inverter">
```

```
    <sbol:persistentIdentity rdf:resource="http://sbolstandard.org/example/laci_inverter"/>
    <sbol:displayId>laci_inverter</sbol:displayId>
    <sbol:role rdf:resource="http://parts.igem.org/cgi/partsdb/pgroup.cgi?pgroup=inverter"/>
    <sbol:functionalComponent>
      <sbol:FunctionalComponent rdf:about="http://sbolstandard.org/example/laci_inverter/TF">
        <sbol:persistentIdentity rdf:resource="http://sbolstandard.org/example/laci_inverter/TF"/>
        <sbol:displayId>TF</sbol:displayId>
        <sbol:definition rdf:resource="http://identifiers.org/uniprot/P03023"/>
        <sbol:access rdf:resource="http://sbols.org/v2#public"/>
        <sbol:direction rdf:resource="http://sbols.org/v2#inout"/>
      </sbol:FunctionalComponent>
    </sbol:functionalComponent>
    <sbol:functionalComponent>
      <sbol:FunctionalComponent rdf:about="http://sbolstandard.org/example/laci_inverter/promoter">
        <sbol:persistentIdentity rdf:resource="http://sbolstandard.org/example/laci_inverter/promoter"/>
        <sbol:displayId>promoter</sbol:displayId>
        <sbol:definition rdf:resource="http://www.partsregistry.org/BBa_R0010"/>
        <sbol:access rdf:resource="http://sbols.org/v2#public"/>
        <sbol:direction rdf:resource="http://sbols.org/v2#inout"/>
      </sbol:FunctionalComponent>
    </sbol:functionalComponent>
    <sbol:interaction>
      <sbol:Interaction rdf:about="http://sbolstandard.org/example/laci_inverter/LacI_pLacI">
        <sbol:persistentIdentity rdf:resource="http://sbolstandard.org/example/laci_inverter/LacI_pLacI"/>
        <sbol:displayId>LacI_pLacI</sbol:displayId>
        <sbol:type rdf:resource="http://identifiers.org/biomodels.sbo/SBO:0000169"/>
        <sbol:participation>
          <sbol:Participation rdf:about="http://sbolstandard.org/example/laci_inverter/LacI_pLacI/BBa_R0010">
            <sbol:persistentIdentity rdf:resource="http://sbolstandard.org/example/laci_inverter/LacI_pLacI/BBa_R0010"/>
            <sbol:displayId>BBa_R0010</sbol:displayId>
            <sbol:role rdf:resource="http://identifiers.org/biomodels.sbo/SBO:0000598"/>
            <sbol:participant rdf:resource="http://sbolstandard.org/example/laci_inverter/promoter"/>
          </sbol:Participation>
        </sbol:participation>
        <sbol:participation>
          <sbol:Participation rdf:about="http://sbolstandard.org/example/laci_inverter/LacI_pLacI/P03023">
            <sbol:persistentIdentity rdf:resource="http://sbolstandard.org/example/laci_inverter/LacI_pLacI/P03023"/>
            <sbol:displayId>P03023</sbol:displayId>
            <sbol:role rdf:resource="http://identifiers.org/biomodels.sbo/SBO:0000020"/>
            <sbol:participant rdf:resource="http://sbolstandard.org/example/laci_inverter/TF"/>
          </sbol:Participation>
        </sbol:participation>
      </sbol:Interaction>
    </sbol:interaction>
  </sbol:ModuleDefinition>
  <sbol:ModuleDefinition rdf:about="http://sbolstandard.org/example/tetr_inverter">
    <sbol:persistentIdentity rdf:resource="http://sbolstandard.org/example/tetr_inverter"/>
    <sbol:displayId>tetr_inverter</sbol:displayId>
    <sbol:role rdf:resource="http://parts.igem.org/cgi/partsdb/pgroup.cgi?pgroup=inverter"/>
    <sbol:functionalComponent>
      <sbol:FunctionalComponent rdf:about="http://sbolstandard.org/example/tetr_inverter/TF">
        <sbol:persistentIdentity rdf:resource="http://sbolstandard.org/example/tetr_inverter/TF"/>
        <sbol:displayId>TF</sbol:displayId>
        <sbol:definition rdf:resource="http://identifiers.org/uniprot/Q6QR72"/>
        <sbol:access rdf:resource="http://sbols.org/v2#public"/>
        <sbol:direction rdf:resource="http://sbols.org/v2#inout"/>
      </sbol:FunctionalComponent>
    </sbol:functionalComponent>
    <sbol:functionalComponent>
      <sbol:FunctionalComponent rdf:about="http://sbolstandard.org/example/tetr_inverter/promoter">
        <sbol:persistentIdentity rdf:resource="http://sbolstandard.org/example/tetr_inverter/promoter"/>
        <sbol:displayId>promoter</sbol:displayId>
        <sbol:definition rdf:resource="http://www.partsregistry.org/BBa_R0040"/>
        <sbol:access rdf:resource="http://sbols.org/v2#public"/>
        <sbol:direction rdf:resource="http://sbols.org/v2#inout"/>
      </sbol:FunctionalComponent>
    </sbol:functionalComponent>
    <sbol:interaction>
      <sbol:Interaction rdf:about="http://sbolstandard.org/example/tetr_inverter/LacI_pLacI">
```

```
        <sbol:persistentIdentity rdf:resource="http://sbolstandard.org/example/tetr_inverter/LacI_pLacI"/>
        <sbol:displayId>LacI_pLacI</sbol:displayId>
        <sbol:type rdf:resource="http://identifiers.org/biomodels.sbo/SBO:0000169"/>
        <sbol:participation>
          <sbol:Participation rdf:about="http://sbolstandard.org/example/tetr_inverter/LacI_pLacI/Q6QR72">
            <sbol:persistentIdentity rdf:resource="http://sbolstandard.org/example/tetr_inverter/LacI_pLacI/Q6QR72"/>
            <sbol:displayId>Q6QR72</sbol:displayId>
            <sbol:role rdf:resource="http://identifiers.org/biomodels.sbo/SBO:0000020"/>
            <sbol:participant rdf:resource="http://sbolstandard.org/example/tetr_inverter/TF"/>
          </sbol:Participation>
        </sbol:participation>
        <sbol:participation>
          <sbol:Participation rdf:about="http://sbolstandard.org/example/tetr_inverter/LacI_pLacI/BBa_R0040">
            <sbol:persistentIdentity rdf:resource="http://sbolstandard.org/example/tetr_inverter/LacI_pLacI/BBa_R0040"/>
            <sbol:displayId>BBa_R0040</sbol:displayId>
            <sbol:role rdf:resource="http://identifiers.org/biomodels.sbo/SBO:0000598"/>
            <sbol:participant rdf:resource="http://sbolstandard.org/example/tetr_inverter/promoter"/>
          </sbol:Participation>
        </sbol:participation>
      </sbol:Interaction>
    </sbol:interaction>
  </sbol:ModuleDefinition>
  <sbol:Model rdf:about="http://sbolstandard.org/example/toogleswitch">
    <sbol:persistentIdentity rdf:resource="http://sbolstandard.org/example/toogleswitch"/>
    <sbol:displayId>toogleswitch</sbol:displayId>
    <sbol:source rdf:resource="http://virtualparts.org/part/pIKE_Toggle_1"/>
    <sbol:language rdf:resource="http://identifiers.org/edam/format_2585"/>
    <sbol:framework rdf:resource="http://identifiers.org/biomodels.sbo/SBO:0000062"/>
  </sbol:Model>
  <sbol:ComponentDefinition rdf:about="http://www.virtualparts.org/part/pIKE_Toggle_1">
    <sbol:persistentIdentity rdf:resource="http://www.virtualparts.org/part/pIKE_Toggle_1"/>
    <sbol:displayId>pIKE_Toggle_1</sbol:displayId>
    <dcterms:title>LacI/TetR Toggle Switch</dcterms:title>
    <dcterms:description>LacI/TetR Toggle Switch</dcterms:description>
    <sbol:type rdf:resource="http://www.biopax.org/release/biopax-level3.owl#DnaRegion"/>
    <sbol:role rdf:resource="http://identifiers.org/so/SO:0000280"/>
    <sbol:component>
      <sbol:Component rdf:about="http://www.virtualparts.org/part/pIKE_Toggle_1/pIKERightCassette_1">
        <sbol:persistentIdentity rdf:resource="http://www.virtualparts.org/part/pIKE_Toggle_1/pIKERightCassette_1"/>
        <sbol:displayId>pIKERightCassette_1</sbol:displayId>
        <sbol:access rdf:resource="http://sbols.org/v2#public"/>
        <sbol:definition rdf:resource="http://www.virtualparts.org/part/pIKERightCassette_1"/>
      </sbol:Component>
    </sbol:component>
    <sbol:component>
      <sbol:Component rdf:about="http://www.virtualparts.org/part/pIKE_Toggle_1/pIKELeftCassette_1">
        <sbol:persistentIdentity rdf:resource="http://www.virtualparts.org/part/pIKE_Toggle_1/pIKELeftCassette_1"/>
        <sbol:displayId>pIKELeftCassette_1</sbol:displayId>
        <sbol:access rdf:resource="http://sbols.org/v2#public"/>
        <sbol:definition rdf:resource="http://www.virtualparts.org/part/pIKELeftCassette_1"/>
      </sbol:Component>
    </sbol:component>
    <sbol:sequenceAnnotation>
      <sbol:SequenceAnnotation rdf:about="http://www.virtualparts.org/part/pIKE_Toggle_1/anno2">
        <sbol:persistentIdentity rdf:resource="http://www.virtualparts.org/part/pIKE_Toggle_1/anno2"/>
        <sbol:displayId>anno2</sbol:displayId>
        <sbol:location>
          <sbol:Range rdf:about="http://www.virtualparts.org/part/pIKE_Toggle_1/anno2/location2">
            <sbol:persistentIdentity rdf:resource="http://www.virtualparts.org/part/pIKE_Toggle_1/anno2/location2"/>
            <sbol:displayId>location2</sbol:displayId>
            <sbol:start>1286</sbol:start>
            <sbol:end>2834</sbol:end>
            <sbol:orientation rdf:resource="http://sbols.org/v2#inline"/>
          </sbol:Range>
        </sbol:location>
        <sbol:component rdf:resource="http://www.virtualparts.org/part/pIKE_Toggle_1/pIKERightCassette_1"/>
      </sbol:SequenceAnnotation>
    </sbol:sequenceAnnotation>
    <sbol:sequenceAnnotation>
```

```
      <sbol:SequenceAnnotation rdf:about="http://www.virtualparts.org/part/pIKE_Toggle_1/anno1">
        <sbol:persistentIdentity rdf:resource="http://www.virtualparts.org/part/pIKE_Toggle_1/anno1"/>
        <sbol:displayId>anno1</sbol:displayId>
        <sbol:location>
          <sbol:Range rdf:about="http://www.virtualparts.org/part/pIKE_Toggle_1/anno1/location1">
            <sbol:persistentIdentity rdf:resource="http://www.virtualparts.org/part/pIKE_Toggle_1/anno1/location1"/>
            <sbol:displayId>location1</sbol:displayId>
            <sbol:start>1</sbol:start>
            <sbol:end>1285</sbol:end>
            <sbol:orientation rdf:resource="http://sbols.org/v2#inline"/>
          </sbol:Range>
        </sbol:location>
        <sbol:component rdf:resource="http://www.virtualparts.org/part/pIKE_Toggle_1/pIKELeftCassette_1"/>
      </sbol:SequenceAnnotation>
    </sbol:sequenceAnnotation>
  </sbol:ComponentDefinition>
  <sbol:ComponentDefinition rdf:about="http://www.partsregistry.org/BBa_J61130">
    <sbol:persistentIdentity rdf:resource="http://www.partsregistry.org/BBa_J61130"/>
    <sbol:displayId>BBa_J61130</sbol:displayId>
    <dcterms:title>BBa_J61101 RBS</dcterms:title>
    <dcterms:description>RBS2</dcterms:description>
    <sbol:type rdf:resource="http://www.biopax.org/release/biopax-level3.owl#DnaRegion"/>
    <sbol:role rdf:resource="http://identifiers.org/so/SO:0000139"/>
    <sbol:sequence rdf:resource="http://www.virtualparts.org/part/BBa_J61130"/>
  </sbol:ComponentDefinition>
  <sbol:ComponentDefinition rdf:about="http://www.partsregistry.org/BBa_C0012">
    <sbol:persistentIdentity rdf:resource="http://www.partsregistry.org/BBa_C0012"/>
    <sbol:displayId>BBa_C0012</sbol:displayId>
    <dcterms:title>lacI</dcterms:title>
    <dcterms:description>lacI coding sequence</dcterms:description>
    <sbol:type rdf:resource="http://www.biopax.org/release/biopax-level3.owl#DnaRegion"/>
    <sbol:role rdf:resource="http://identifiers.org/so/SO:0000316"/>
    <sbol:sequence rdf:resource="http://www.virtualparts.org/part/BBa_C0012"/>
  </sbol:ComponentDefinition>
  <sbol:ComponentDefinition rdf:about="http://www.partsregistry.org/ECK120033736">
    <sbol:persistentIdentity rdf:resource="http://www.partsregistry.org/ECK120033736"/>
    <sbol:displayId>ECK120033736</sbol:displayId>
    <dcterms:title>ECK120033736</dcterms:title>
    <dcterms:description>Terminator2</dcterms:description>
    <sbol:type rdf:resource="http://www.biopax.org/release/biopax-level3.owl#DnaRegion"/>
    <sbol:role rdf:resource="http://identifiers.org/so/SO:0000141"/>
    <sbol:sequence rdf:resource="http://www.virtualparts.org/part/ECK120033736"/>
  </sbol:ComponentDefinition>
  <sbol:ComponentDefinition rdf:about="http://www.partsregistry.org/BBa_R0040">
    <sbol:persistentIdentity rdf:resource="http://www.partsregistry.org/BBa_R0040"/>
    <sbol:displayId>BBa_R0040</sbol:displayId>
    <dcterms:title>pTetR</dcterms:title>
    <dcterms:description>pTet promoter</dcterms:description>
    <sbol:type rdf:resource="http://www.biopax.org/release/biopax-level3.owl#DnaRegion"/>
    <sbol:role rdf:resource="http://identifiers.org/so/SO:0000167"/>
    <sbol:sequence rdf:resource="http://www.virtualparts.org/part/BBa_R0040"/>
  </sbol:ComponentDefinition>
  <sbol:ComponentDefinition rdf:about="http://identifiers.org/uniprot/Q6QR72">
    <sbol:persistentIdentity rdf:resource="http://identifiers.org/uniprot/Q6QR72"/>
    <sbol:displayId>Q6QR72</sbol:displayId>
    <dcterms:title>TetR</dcterms:title>
    <dcterms:description>TetR protein</dcterms:description>
    <sbol:type rdf:resource="http://www.biopax.org/release/biopax-level3.owl#Protein"/>
    <sbol:role rdf:resource="http://identifiers.org/biomodels.sbo/SBO:0000020"/>
  </sbol:ComponentDefinition>
  <sbol:ComponentDefinition rdf:about="http://identifiers.org/uniprot/P03023">
    <sbol:persistentIdentity rdf:resource="http://identifiers.org/uniprot/P03023"/>
    <sbol:displayId>P03023</sbol:displayId>
    <dcterms:title>LacI</dcterms:title>
    <dcterms:description>LacI protein</dcterms:description>
    <sbol:type rdf:resource="http://www.biopax.org/release/biopax-level3.owl#Protein"/>
    <sbol:role rdf:resource="http://identifiers.org/biomodels.sbo/SBO:0000020"/>
  </sbol:ComponentDefinition>
  <sbol:ComponentDefinition rdf:about="http://www.partsregistry.org/BBa_J61120">
```

```
    <sbol:persistentIdentity rdf:resource="http://www.partsregistry.org/BBa_J61120"/>
    <sbol:displayId>BBa_J61120</sbol:displayId>
    <dcterms:title>BBa_J61101 RBS</dcterms:title>
    <dcterms:description>RBS2</dcterms:description>
    <sbol:type rdf:resource="http://www.biopax.org/release/biopax-level3.owl#DnaRegion"/>
    <sbol:role rdf:resource="http://identifiers.org/so/SO:0000139"/>
    <sbol:sequence rdf:resource="http://www.virtualparts.org/part/BBa_J61120"/>
  </sbol:ComponentDefinition>
  <sbol:ComponentDefinition rdf:about="http://www.partsregistry.org/BBa_E0040">
    <sbol:persistentIdentity rdf:resource="http://www.partsregistry.org/BBa_E0040"/>
    <sbol:displayId>BBa_E0040</sbol:displayId>
    <dcterms:title>gfp</dcterms:title>
    <dcterms:description>gfp coding sequence</dcterms:description>
    <sbol:type rdf:resource="http://www.biopax.org/release/biopax-level3.owl#DnaRegion"/>
    <sbol:role rdf:resource="http://identifiers.org/so/SO:0000316"/>
    <sbol:sequence rdf:resource="http://www.virtualparts.org/part/BBa_E0040"/>
  </sbol:ComponentDefinition>
  <sbol:ComponentDefinition rdf:about="http://www.partsregistry.org/ECK120029600">
    <sbol:persistentIdentity rdf:resource="http://www.partsregistry.org/ECK120029600"/>
    <sbol:displayId>ECK120029600</sbol:displayId>
    <dcterms:title>ECK120029600</dcterms:title>
    <dcterms:description>Terminator1</dcterms:description>
    <sbol:type rdf:resource="http://www.biopax.org/release/biopax-level3.owl#DnaRegion"/>
    <sbol:role rdf:resource="http://identifiers.org/so/SO:0000141"/>
    <sbol:sequence rdf:resource="http://www.virtualparts.org/part/ECK120029600"/>
  </sbol:ComponentDefinition>
  <sbol:ComponentDefinition rdf:about="http://www.virtualparts.org/part/pIKELeftCassette_1">
    <sbol:persistentIdentity rdf:resource="http://www.virtualparts.org/part/pIKELeftCassette_1"/>
    <sbol:displayId>pIKELeftCassette_1</sbol:displayId>
    <dcterms:title>TetR Inverter</dcterms:title>
    <dcterms:description>TetR Inverter</dcterms:description>
    <sbol:type rdf:resource="http://www.biopax.org/release/biopax-level3.owl#DnaRegion"/>
    <sbol:role rdf:resource="http://identifiers.org/so/SO:0000280"/>
    <sbol:component>
      <sbol:Component rdf:about="http://www.virtualparts.org/part/pIKELeftCassette_1/ECK120029600">
        <sbol:persistentIdentity rdf:resource="http://www.virtualparts.org/part/pIKELeftCassette_1/ECK120029600"/>
        <sbol:displayId>ECK120029600</sbol:displayId>
        <sbol:access rdf:resource="http://sbols.org/v2#public"/>
        <sbol:definition rdf:resource="http://www.partsregistry.org/ECK120029600"/>
      </sbol:Component>
    </sbol:component>
    <sbol:component>
      <sbol:Component rdf:about="http://www.virtualparts.org/part/pIKELeftCassette_1/BBa_R0040">
        <sbol:persistentIdentity rdf:resource="http://www.virtualparts.org/part/pIKELeftCassette_1/BBa_R0040"/>
        <sbol:displayId>BBa_R0040</sbol:displayId>
        <sbol:access rdf:resource="http://sbols.org/v2#public"/>
        <sbol:definition rdf:resource="http://www.partsregistry.org/BBa_R0040"/>
      </sbol:Component>
    </sbol:component>
    <sbol:component>
      <sbol:Component rdf:about="http://www.virtualparts.org/part/pIKELeftCassette_1/BBa_C0012">
        <sbol:persistentIdentity rdf:resource="http://www.virtualparts.org/part/pIKELeftCassette_1/BBa_C0012"/>
        <sbol:displayId>BBa_C0012</sbol:displayId>
        <sbol:access rdf:resource="http://sbols.org/v2#public"/>
        <sbol:definition rdf:resource="http://www.partsregistry.org/BBa_C0012"/>
      </sbol:Component>
    </sbol:component>
    <sbol:component>
      <sbol:Component rdf:about="http://www.virtualparts.org/part/pIKELeftCassette_1/BBa_J61101">
        <sbol:persistentIdentity rdf:resource="http://www.virtualparts.org/part/pIKELeftCassette_1/BBa_J61101"/>
        <sbol:displayId>BBa_J61101</sbol:displayId>
        <sbol:access rdf:resource="http://sbols.org/v2#public"/>
        <sbol:definition rdf:resource="http://www.partsregistry.org/BBa_J61101"/>
      </sbol:Component>
    </sbol:component>
    <sbol:sequenceAnnotation>
      <sbol:SequenceAnnotation rdf:about="http://www.virtualparts.org/part/pIKELeftCassette_1/anno4">
        <sbol:persistentIdentity rdf:resource="http://www.virtualparts.org/part/pIKELeftCassette_1/anno4"/>
        <sbol:displayId>anno4</sbol:displayId>
```

```
        <sbol:location>
          <sbol:Range rdf:about="http://www.virtualparts.org/part/pIKELeftCassette_1/anno4/location4">
            <sbol:persistentIdentity rdf:resource="http://www.virtualparts.org/part/pIKELeftCassette_1/anno4/location4"/>
            <sbol:displayId>location4</sbol:displayId>
            <sbol:start>1198</sbol:start>
            <sbol:end>1288</sbol:end>
            <sbol:orientation rdf:resource="http://sbols.org/v2#inline"/>
          </sbol:Range>
        </sbol:location>
        <sbol:component rdf:resource="http://www.virtualparts.org/part/pIKELeftCassette_1/ECK120029600"/>
      </sbol:SequenceAnnotation>
    </sbol:sequenceAnnotation>
    <sbol:sequenceAnnotation>
      <sbol:SequenceAnnotation rdf:about="http://www.virtualparts.org/part/pIKELeftCassette_1/anno2">
        <sbol:persistentIdentity rdf:resource="http://www.virtualparts.org/part/pIKELeftCassette_1/anno2"/>
        <sbol:displayId>anno2</sbol:displayId>
        <sbol:location>
          <sbol:Range rdf:about="http://www.virtualparts.org/part/pIKELeftCassette_1/anno2/location2">
            <sbol:persistentIdentity rdf:resource="http://www.virtualparts.org/part/pIKELeftCassette_1/anno2/location2"/>
            <sbol:displayId>location2</sbol:displayId>
            <sbol:start>56</sbol:start>
            <sbol:end>68</sbol:end>
            <sbol:orientation rdf:resource="http://sbols.org/v2#inline"/>
          </sbol:Range>
        </sbol:location>
        <sbol:component rdf:resource="http://www.virtualparts.org/part/pIKELeftCassette_1/BBa_J61101"/>
      </sbol:SequenceAnnotation>
    </sbol:sequenceAnnotation>
    <sbol:sequenceAnnotation>
      <sbol:SequenceAnnotation rdf:about="http://www.virtualparts.org/part/pIKELeftCassette_1/anno1">
        <sbol:persistentIdentity rdf:resource="http://www.virtualparts.org/part/pIKELeftCassette_1/anno1"/>
        <sbol:displayId>anno1</sbol:displayId>
        <sbol:location>
          <sbol:Range rdf:about="http://www.virtualparts.org/part/pIKELeftCassette_1/anno1/location1">
            <sbol:persistentIdentity rdf:resource="http://www.virtualparts.org/part/pIKELeftCassette_1/anno1/location1"/>
            <sbol:displayId>location1</sbol:displayId>
            <sbol:start>1</sbol:start>
            <sbol:end>55</sbol:end>
            <sbol:orientation rdf:resource="http://sbols.org/v2#inline"/>
          </sbol:Range>
        </sbol:location>
        <sbol:component rdf:resource="http://www.virtualparts.org/part/pIKELeftCassette_1/BBa_R0040"/>
      </sbol:SequenceAnnotation>
    </sbol:sequenceAnnotation>
    <sbol:sequenceAnnotation>
      <sbol:SequenceAnnotation rdf:about="http://www.virtualparts.org/part/pIKELeftCassette_1/anno3">
        <sbol:persistentIdentity rdf:resource="http://www.virtualparts.org/part/pIKELeftCassette_1/anno3"/>
        <sbol:displayId>anno3</sbol:displayId>
        <sbol:location>
          <sbol:Range rdf:about="http://www.virtualparts.org/part/pIKELeftCassette_1/anno3/location3">
            <sbol:persistentIdentity rdf:resource="http://www.virtualparts.org/part/pIKELeftCassette_1/anno3/location3"/>
            <sbol:displayId>location3</sbol:displayId>
            <sbol:start>69</sbol:start>
            <sbol:end>1197</sbol:end>
            <sbol:orientation rdf:resource="http://sbols.org/v2#inline"/>
          </sbol:Range>
        </sbol:location>
        <sbol:component rdf:resource="http://www.virtualparts.org/part/pIKELeftCassette_1/BBa_C0012"/>
      </sbol:SequenceAnnotation>
    </sbol:sequenceAnnotation>
  </sbol:ComponentDefinition>
  <sbol:ComponentDefinition rdf:about="http://www.partsregistry.org/BBa_J61101">
    <sbol:persistentIdentity rdf:resource="http://www.partsregistry.org/BBa_J61101"/>
    <sbol:displayId>BBa_J61101</sbol:displayId>
    <dcterms:title>BBa_J61101 RBS</dcterms:title>
    <dcterms:description>RBS1</dcterms:description>
    <sbol:type rdf:resource="http://www.biopax.org/release/biopax-level3.owl#DnaRegion"/>
    <sbol:role rdf:resource="http://identifiers.org/so/SO:0000139"/>
    <sbol:sequence rdf:resource="http://www.virtualparts.org/part/BBa_J61101"/>
```

```xml
</sbol:ComponentDefinition>
<sbol:ComponentDefinition rdf:about="http://www.partsregistry.org/BBa_R0010">
  <sbol:persistentIdentity rdf:resource="http://www.partsregistry.org/BBa_R0010"/>
  <sbol:displayId>BBa_R0010</sbol:displayId>
  <dcterms:title>pLacI</dcterms:title>
  <dcterms:description>pLacI promoter</dcterms:description>
  <sbol:type rdf:resource="http://www.biopax.org/release/biopax-level3.owl#DnaRegion"/>
  <sbol:role rdf:resource="http://identifiers.org/so/SO:0000167"/>
  <sbol:sequence rdf:resource="http://www.virtualparts.org/part/BBa_R0010"/>
</sbol:ComponentDefinition>
<sbol:ComponentDefinition rdf:about="http://identifiers.org/uniprot/P42212">
  <sbol:persistentIdentity rdf:resource="http://identifiers.org/uniprot/P42212"/>
  <sbol:displayId>P42212</sbol:displayId>
  <dcterms:title>GFP</dcterms:title>
  <dcterms:description>GFP protein</dcterms:description>
  <sbol:type rdf:resource="http://www.biopax.org/release/biopax-level3.owl#Protein"/>
  <sbol:role rdf:resource="http://identifiers.org/biomodels.sbo/SBO:0000011"/>
</sbol:ComponentDefinition>
<sbol:ComponentDefinition rdf:about="http://www.virtualparts.org/part/pIKERightCassette_1">
  <sbol:persistentIdentity rdf:resource="http://www.virtualparts.org/part/pIKERightCassette_1"/>
  <sbol:displayId>pIKERightCassette_1</sbol:displayId>
  <dcterms:title>LacI Inverter</dcterms:title>
  <dcterms:description>LacI Inverter</dcterms:description>
  <sbol:type rdf:resource="http://www.biopax.org/release/biopax-level3.owl#DnaRegion"/>
  <sbol:role rdf:resource="http://identifiers.org/so/SO:0000280"/>
  <sbol:component>
    <sbol:Component rdf:about="http://www.virtualparts.org/part/pIKERightCassette_1/BBa_R0010">
      <sbol:persistentIdentity rdf:resource="http://www.virtualparts.org/part/pIKERightCassette_1/BBa_R0010"/>
      <sbol:displayId>BBa_R0010</sbol:displayId>
      <sbol:access rdf:resource="http://sbols.org/v2#public"/>
      <sbol:definition rdf:resource="http://www.partsregistry.org/BBa_R0010"/>
    </sbol:Component>
  </sbol:component>
  <sbol:component>
    <sbol:Component rdf:about="http://www.virtualparts.org/part/pIKERightCassette_1/BBa_C0040">
      <sbol:persistentIdentity rdf:resource="http://www.virtualparts.org/part/pIKERightCassette_1/BBa_C0040"/>
      <sbol:displayId>BBa_C0040</sbol:displayId>
      <sbol:access rdf:resource="http://sbols.org/v2#public"/>
      <sbol:definition rdf:resource="http://www.partsregistry.org/BBa_C0040"/>
    </sbol:Component>
  </sbol:component>
  <sbol:component>
    <sbol:Component rdf:about="http://www.virtualparts.org/part/pIKERightCassette_1/BBa_J61130">
      <sbol:persistentIdentity rdf:resource="http://www.virtualparts.org/part/pIKERightCassette_1/BBa_J61130"/>
      <sbol:displayId>BBa_J61130</sbol:displayId>
      <sbol:access rdf:resource="http://sbols.org/v2#public"/>
      <sbol:definition rdf:resource="http://www.partsregistry.org/BBa_J61130"/>
    </sbol:Component>
  </sbol:component>
  <sbol:component>
    <sbol:Component rdf:about="http://www.virtualparts.org/part/pIKERightCassette_1/BBa_E0040">
      <sbol:persistentIdentity rdf:resource="http://www.virtualparts.org/part/pIKERightCassette_1/BBa_E0040"/>
      <sbol:displayId>BBa_E0040</sbol:displayId>
      <sbol:access rdf:resource="http://sbols.org/v2#public"/>
      <sbol:definition rdf:resource="http://www.partsregistry.org/BBa_E0040"/>
    </sbol:Component>
  </sbol:component>
  <sbol:component>
    <sbol:Component rdf:about="http://www.virtualparts.org/part/pIKERightCassette_1/ECK120033736">
      <sbol:persistentIdentity rdf:resource="http://www.virtualparts.org/part/pIKERightCassette_1/ECK120033736"/>
      <sbol:displayId>ECK120033736</sbol:displayId>
      <sbol:access rdf:resource="http://sbols.org/v2#public"/>
      <sbol:definition rdf:resource="http://www.partsregistry.org/ECK120033736"/>
    </sbol:Component>
  </sbol:component>
  <sbol:component>
    <sbol:Component rdf:about="http://www.virtualparts.org/part/pIKERightCassette_1/BBa_J61120">
      <sbol:persistentIdentity rdf:resource="http://www.virtualparts.org/part/pIKERightCassette_1/BBa_J61120"/>
      <sbol:displayId>BBa_J61120</sbol:displayId>
```

```
        <sbol:access rdf:resource="http://sbols.org/v2#public"/>
        <sbol:definition rdf:resource="http://www.partsregistry.org/BBa_J61120"/>
      </sbol:Component>
    </sbol:component>
    <sbol:sequenceAnnotation>
      <sbol:SequenceAnnotation rdf:about="http://www.virtualparts.org/part/pIKERightCassette_1/anno1">
        <sbol:persistentIdentity rdf:resource="http://www.virtualparts.org/part/pIKERightCassette_1/anno1"/>
        <sbol:displayId>anno1</sbol:displayId>
        <sbol:location>
          <sbol:Range rdf:about="http://www.virtualparts.org/part/pIKERightCassette_1/anno1/location1">
            <sbol:persistentIdentity rdf:resource="http://www.virtualparts.org/part/pIKERightCassette_1/anno1/location1"/>
            <sbol:displayId>location1</sbol:displayId>
            <sbol:start>1</sbol:start>
            <sbol:end>55</sbol:end>
            <sbol:orientation rdf:resource="http://sbols.org/v2#inline"/>
          </sbol:Range>
        </sbol:location>
        <sbol:component rdf:resource="http://www.virtualparts.org/part/pIKERightCassette_1/BBa_R0010"/>
      </sbol:SequenceAnnotation>
    </sbol:sequenceAnnotation>
    <sbol:sequenceAnnotation>
      <sbol:SequenceAnnotation rdf:about="http://www.virtualparts.org/part/pIKERightCassette_1/anno5">
        <sbol:persistentIdentity rdf:resource="http://www.virtualparts.org/part/pIKERightCassette_1/anno5"/>
        <sbol:displayId>anno5</sbol:displayId>
        <sbol:location>
          <sbol:Range rdf:about="http://www.virtualparts.org/part/pIKERightCassette_1/anno5/location5">
            <sbol:persistentIdentity rdf:resource="http://www.virtualparts.org/part/pIKERightCassette_1/anno5/location5"/>
            <sbol:displayId>location5</sbol:displayId>
            <sbol:start>743</sbol:start>
            <sbol:end>1463</sbol:end>
            <sbol:orientation rdf:resource="http://sbols.org/v2#inline"/>
          </sbol:Range>
        </sbol:location>
        <sbol:component rdf:resource="http://www.virtualparts.org/part/pIKERightCassette_1/BBa_E0040"/>
      </sbol:SequenceAnnotation>
    </sbol:sequenceAnnotation>
    <sbol:sequenceAnnotation>
      <sbol:SequenceAnnotation rdf:about="http://www.virtualparts.org/part/pIKERightCassette_1/anno6">
        <sbol:persistentIdentity rdf:resource="http://www.virtualparts.org/part/pIKERightCassette_1/anno6"/>
        <sbol:displayId>anno6</sbol:displayId>
        <sbol:location>
          <sbol:Range rdf:about="http://www.virtualparts.org/part/pIKERightCassette_1/anno6/location6">
            <sbol:persistentIdentity rdf:resource="http://www.virtualparts.org/part/pIKERightCassette_1/anno6/location6"/>
            <sbol:displayId>location6</sbol:displayId>
            <sbol:start>1464</sbol:start>
            <sbol:end>1554</sbol:end>
            <sbol:orientation rdf:resource="http://sbols.org/v2#inline"/>
          </sbol:Range>
        </sbol:location>
        <sbol:component rdf:resource="http://www.virtualparts.org/part/pIKERightCassette_1/ECK120033736"/>
      </sbol:SequenceAnnotation>
    </sbol:sequenceAnnotation>
    <sbol:sequenceAnnotation>
      <sbol:SequenceAnnotation rdf:about="http://www.virtualparts.org/part/pIKERightCassette_1/anno3">
        <sbol:persistentIdentity rdf:resource="http://www.virtualparts.org/part/pIKERightCassette_1/anno3"/>
        <sbol:displayId>anno3</sbol:displayId>
        <sbol:location>
          <sbol:Range rdf:about="http://www.virtualparts.org/part/pIKERightCassette_1/anno3/location3">
            <sbol:persistentIdentity rdf:resource="http://www.virtualparts.org/part/pIKERightCassette_1/anno3/location3"/>
            <sbol:displayId>location3</sbol:displayId>
            <sbol:start>69</sbol:start>
            <sbol:end>729</sbol:end>
            <sbol:orientation rdf:resource="http://sbols.org/v2#inline"/>
          </sbol:Range>
        </sbol:location>
        <sbol:component rdf:resource="http://www.virtualparts.org/part/pIKERightCassette_1/BBa_C0040"/>
      </sbol:SequenceAnnotation>
    </sbol:sequenceAnnotation>
    <sbol:sequenceAnnotation>
```

```
      <sbol:SequenceAnnotation rdf:about="http://www.virtualparts.org/part/pIKERightCassette_1/anno4">
        <sbol:persistentIdentity rdf:resource="http://www.virtualparts.org/part/pIKERightCassette_1/anno4"/>
        <sbol:displayId>anno4</sbol:displayId>
        <sbol:location>
          <sbol:Range rdf:about="http://www.virtualparts.org/part/pIKERightCassette_1/anno4/location4">
            <sbol:persistentIdentity rdf:resource="http://www.virtualparts.org/part/pIKERightCassette_1/anno4/location4"/>
            <sbol:displayId>location4</sbol:displayId>
            <sbol:start>730</sbol:start>
            <sbol:end>742</sbol:end>
            <sbol:orientation rdf:resource="http://sbols.org/v2#inline"/>
          </sbol:Range>
        </sbol:location>
        <sbol:component rdf:resource="http://www.virtualparts.org/part/pIKERightCassette_1/BBa_J61130"/>
      </sbol:SequenceAnnotation>
    </sbol:sequenceAnnotation>
    <sbol:sequenceAnnotation>
      <sbol:SequenceAnnotation rdf:about="http://www.virtualparts.org/part/pIKERightCassette_1/anno2">
        <sbol:persistentIdentity rdf:resource="http://www.virtualparts.org/part/pIKERightCassette_1/anno2"/>
        <sbol:displayId>anno2</sbol:displayId>
        <sbol:location>
          <sbol:Range rdf:about="http://www.virtualparts.org/part/pIKERightCassette_1/anno2/location2">
            <sbol:persistentIdentity rdf:resource="http://www.virtualparts.org/part/pIKERightCassette_1/anno2/location2"/>
            <sbol:displayId>location2</sbol:displayId>
            <sbol:start>56</sbol:start>
            <sbol:end>68</sbol:end>
            <sbol:orientation rdf:resource="http://sbols.org/v2#inline"/>
          </sbol:Range>
        </sbol:location>
        <sbol:component rdf:resource="http://www.virtualparts.org/part/pIKERightCassette_1/BBa_J61120"/>
      </sbol:SequenceAnnotation>
    </sbol:sequenceAnnotation>
  </sbol:ComponentDefinition>
  <sbol:ComponentDefinition rdf:about="http://www.partsregistry.org/BBa_C0040">
    <sbol:persistentIdentity rdf:resource="http://www.partsregistry.org/BBa_C0040"/>
    <sbol:displayId>BBa_C0040</sbol:displayId>
    <dcterms:title>tetR</dcterms:title>
    <dcterms:description>tetR coding sequence</dcterms:description>
    <sbol:type rdf:resource="http://www.biopax.org/release/biopax-level3.owl#DnaRegion"/>
    <sbol:role rdf:resource="http://identifiers.org/so/SO:0000316"/>
    <sbol:sequence rdf:resource="http://www.virtualparts.org/part/BBa_C0040"/>
  </sbol:ComponentDefinition>
  <sbol:Sequence rdf:about="http://www.virtualparts.org/part/BBa_J61101">
    <sbol:persistentIdentity rdf:resource="http://www.virtualparts.org/part/BBa_J61101"/>
    <sbol:displayId>BBa_J61101</sbol:displayId>
    <sbol:elements>aaagacaggacc</sbol:elements>
    <sbol:encoding rdf:resource="http://www.chem.qmul.ac.uk/iubmb/misc/naseq.html"/>
  </sbol:Sequence>
  <sbol:Sequence rdf:about="http://www.virtualparts.org/part/BBa_J61120">
    <sbol:persistentIdentity rdf:resource="http://www.virtualparts.org/part/BBa_J61120"/>
    <sbol:displayId>BBa_J61120</sbol:displayId>
    <sbol:elements>aaagacaggacc</sbol:elements>
    <sbol:encoding rdf:resource="http://www.chem.qmul.ac.uk/iubmb/misc/naseq.html"/>
  </sbol:Sequence>
  <sbol:Sequence rdf:about="http://www.virtualparts.org/part/BBa_E0040">
    <sbol:persistentIdentity rdf:resource="http://www.virtualparts.org/part/BBa_E0040"/>
    <sbol:displayId>BBa_E0040</sbol:displayId>
    <sbol:elements>atgcgtaaaggagaagaacttttcactggagttgtcccaattcttgttgaattagatggtgatgttaatgggcac
    aaattttctgtcagtggagagggtgaaggtgatgcaacatacggaaaacttacccttaaatttatttgcactactggaaaactacctgtt
    ccatggccaacacttgtcactactttcggttatggtgttcaatgctttgcgagatacccagatcatatgaaacagcatgactttttcaag
    agtgccatgcccgaaggttatgtacaggaaagaactatatttttcaaagatgacgggaactacaagacacgtgctgaagtcaagtttgaa
    ggtgatacccttgttaatagaatcgagttaaaaggtattgattttaaagaagatggaaacattcttggacacaaattggaatacaactat
    aactcacacaatgtatacatcatggcagacaaacaaaagaatggaatcaaagttaacttcaaaattagacacaacattgaagatggaagc
    gttcaactagcagaccattatcaacaaaatactccaattggcgatggccctgtccttttaccagacaaccattacctgtccacacaatct
    gccctttcgaaagatcccaacgaaaagagagaccacatggtccttcttgagtttgtaacagctgctgggattacacatggcatggatgaa
    ctatacaaataataa</sbol:elements>
    <sbol:encoding rdf:resource="http://www.chem.qmul.ac.uk/iubmb/misc/naseq.html"/>
  </sbol:Sequence>
  <sbol:Sequence rdf:about="http://www.virtualparts.org/part/ECK120033736">
    <sbol:persistentIdentity rdf:resource="http://www.virtualparts.org/part/ECK120033736"/>
```

```xml
    <sbol:displayId>ECK120033736</sbol:displayId>
    <sbol:elements>ttcagccaaaaaacttaagaccgccggtcttgtccactaccttgcagtaatgcggtggacaggatcggcggtttt
    cttttctcttctcaa</sbol:elements>
    <sbol:encoding rdf:resource="http://www.chem.qmul.ac.uk/iubmb/misc/naseq.html"/>
  </sbol:Sequence>
  <sbol:Sequence rdf:about="http://www.virtualparts.org/part/BBa_R0010">
    <sbol:persistentIdentity rdf:resource="http://www.virtualparts.org/part/BBa_R0010"/>
    <sbol:displayId>BBa_R0010</sbol:displayId>
    <sbol:elements>tccctatcagtgatagagattgacatccctatcagtgatagagatactgagcac</sbol:elements>
    <sbol:encoding rdf:resource="http://www.chem.qmul.ac.uk/iubmb/misc/naseq.html"/>
  </sbol:Sequence>
  <sbol:Sequence rdf:about="http://www.virtualparts.org/part/BBa_R0040">
    <sbol:persistentIdentity rdf:resource="http://www.virtualparts.org/part/BBa_R0040"/>
    <sbol:displayId>BBa_R0040</sbol:displayId>
    <sbol:elements>tccctatcagtgatagagattgacatccctatcagtgatagagatactgagcac</sbol:elements>
    <sbol:encoding rdf:resource="http://www.chem.qmul.ac.uk/iubmb/misc/naseq.html"/>
  </sbol:Sequence>
  <sbol:Sequence rdf:about="http://www.virtualparts.org/part/BBa_J61130">
    <sbol:persistentIdentity rdf:resource="http://www.virtualparts.org/part/BBa_J61130"/>
    <sbol:displayId>BBa_J61130</sbol:displayId>
    <sbol:elements>aaagaaacgaca</sbol:elements>
    <sbol:encoding rdf:resource="http://www.chem.qmul.ac.uk/iubmb/misc/naseq.html"/>
  </sbol:Sequence>
  <sbol:Sequence rdf:about="http://www.virtualparts.org/part/BBa_C0040">
    <sbol:persistentIdentity rdf:resource="http://www.virtualparts.org/part/BBa_C0040"/>
    <sbol:displayId>BBa_C0040</sbol:displayId>
    <sbol:elements>atgtccagattagataaaagtaaagtgattaacagcgcattagagctgcttaatgaggtcggaatcgaaggttta
    acaacccgtaaactcgcccagaagctaggtgtagagcagcctacattgtattggcatgtaaaaaataagcgggctttgctcgacgcctta
    gccattgagatgttagataggcaccatactcacttttgccctttagaaggggaaagctggcaagatttttttacgtaataacgctaaaagt
    tttagatgtgctttactaagtcatcgcgatggagcaaaagtacatttaggtacacggcctacagaaaaacagtatgaaactctcgaaaat
    caattagcctttttatgccaacaaggtttttcactagagaatgcattatatgcactcagcgctgtggggcattttactttaggttgcgta
    ttggaagatcaagagcatcaagtcgctaaagaagaaagggaaacacctactactgatagtatgccgccattattacgacaagctatcgaa
    ttatttgatcaccaaggtgcagagccagccttcttattcggccttgaattgatcatatgcggattagaaaaacaacttaaatgtgaaagt
    gggtccgctgcaaacgacgaaaactacgctttagtagcttaataa</sbol:elements>
    <sbol:encoding rdf:resource="http://www.chem.qmul.ac.uk/iubmb/misc/naseq.html"/>
  </sbol:Sequence>
  <sbol:Sequence rdf:about="http://www.virtualparts.org/part/BBa_C0012">
    <sbol:persistentIdentity rdf:resource="http://www.virtualparts.org/part/BBa_C0012"/>
    <sbol:displayId>BBa_C0012</sbol:displayId>
    <sbol:elements>atggtgaatgtgaaaccagtaacgttatacgatgtcgcagagtatgccggtgtctcttatcagaccgtttcccgc
    gtggtgaaccaggccagccacgtttctgcgaaaacgcgggaaaaagtggaagcggcgatggcggagctgaattacattcccaaccgcgtg
    gcacaacaactggcgggcaaacagtcgttgctgattggcgttgccacctccagtctggccctgcacgcgccgtcgcaaattgtcgcggcg
    attaaatctcgcgccgatcaactgggtgccagcgtggtggtgtcgatggtagaacgaagcggcgtcgaagcctgtaaagcggcggtgcac
    aatcttctcgcgcaacgcgtcagtgggctgatcattaactatccgctggatgaccaggatgccattgctgtggaagctgcctgcactaat
    gttccggcgttatttcttgatgtctctgaccagacacccatcaacagtattattttctcccatgaagacggtacgcgactgggcgtggag
    catctggtcgcattgggtcaccagcaaatcgcgctgttagcgggcccattaagttctgtctcggcgcgtctgcgtctggctggctggcat
    aaatatctcactcgcaatcaaattcagccgatagcggaacgggaaggcgactggagtgccatgtccggttttcaacaaaccatgcaaatg
    ctgaatgagggcatcgttcccactgcgatgctggttgccaacgatcagatggcgctgggcgcaatgcgcgccattaccgagtccgggctg
    cgcgttggtgcggatatctcggtagtgggatacgacgataccgaagacagctcatgttatatcccgccgttaaccaccatcaaacaggat
    tttcgcctgctggggcaaaccagcgtggaccgcttgctgcaactctctcagggccaggcggtgaagggcaatcagctgttgcccgtctca
    ctggtgaaaagaaaaaccaccctggcgcccaatacgcaaaccgcctctccccgcgcgttggccgattcattaatgcagctggcacgacag
    gtttcccgactggaaagcgggcaggctgcaaacgacgaaaactacgctttagtagcttaataa</sbol:elements>
    <sbol:encoding rdf:resource="http://www.chem.qmul.ac.uk/iubmb/misc/naseq.html"/>
  </sbol:Sequence>
  <sbol:Sequence rdf:about="http://www.virtualparts.org/part/ECK120029600">
    <sbol:persistentIdentity rdf:resource="http://www.virtualparts.org/part/ECK120029600"/>
    <sbol:displayId>ECK120029600</sbol:displayId>
    <sbol:elements>ttcagccaaaaaacttaagaccgccggtcttgtccactaccttgcagtaatgcggtggacaggatcggcggtttt
    cttttctcttctcaa</sbol:elements>
    <sbol:encoding rdf:resource="http://www.chem.qmul.ac.uk/iubmb/misc/naseq.html"/>
  </sbol:Sequence>
</rdf:RDF>
```
